# XIX International Botanical Congress, Shenzhen: report of the Nomenclature Section, 17 ^th^ to 21 ^st^ July 2017

**DOI:** 10.3897/phytokeys.150.50687

**Published:** 2020-06-08

**Authors:** Heather L. Lindon, Helen Hartley, Sandra Knapp, Anna M. Monro, Nicholas J. Turland

**Affiliations:** 1 Royal Botanic Gardens, Kew, Richmond, Surrey TW9 3AE, UK Royal Botanic Gardens Kew Richmond United Kingdom; 2 The Natural History Museum, Cromwell Road, London SW7 5BD, UK The Natural History Museum London United Kingdom; 3 Centre for Australian National Biodiversity Research, GPO Box 1700, Canberra ACT 2601, Australia Centre for Australian National Biodiversity Research Canberra Australia; 4 Botanischer Garten und Botanisches Museum Berlin, Freie Universität Berlin, Königin-Luise-Str. 6–8, 14195, Berlin, Germany Freie Universität Berlin Berlin Germany

## Preface

This is the official report on the discussions and decisions of the ten sessions of the Nomenclature Section of the XIX International Botanical Congress held in Shenzhen, China, in July 2017. The sessions of the Section took place in Lecture Hall 502, 5^th^ Floor, Peking University HSBC Business School, University Town, Nanshan District, Shenzhen 518055, Guangdong, China, from Monday, 17^th^ July 2017 to Friday, 21^st^ July 2017, inclusive, prior to the main programme of the Congress (23^rd^ to 29^th^ July). The sessions began at 08:00 and finished at 18:00 each day (with 30-minute breaks in the morning and afternoon and a 90-minute break for lunch), except on Friday, when the final session concluded at 17:00. Technical facilities included full electronic audio recording of all discussion spoken into the microphones delivered to the members by an energetic team of volunteers, video recording by a fixed camera facing the front of the auditorium where the Bureau of Nomenclature and two projection screens were located, and a second video camera focusing on general members of the Section as they spoke. Text of all proposals to amend the *Code* was displayed on one screen, while the relevant text of the *Melbourne Code* (McNeill & al. in Regnum Veg. 154. 2012 https://www.iapt-taxon.org/melbourne/main.php) was displayed on a second screen allowing suggested amendments to be updated as appropriate. The local organizers ensured that the entire complicated proceeding ran smoothly and comfortably.

The Section had the honour of being welcomed by Prof. De-Yuan Hong, Academician of the Chinese Academy of Sciences, Institute of Botany, Chinese Academy of Sciences, Beijing.

There was a strong female presence in leadership positions, although the ratio of registered members was tipped toward the male side, more so than in Melbourne (c. 26% of the registered members in Shenzhen were women, compared with c. 33% in Melbourne).

Four of us (HLL, HH, AMM, and NJT) recognize Sandra (Sandy) Knapp for exemplary service as President of the Section, reprising the role she first performed in Melbourne six years previously. Her deft handling of the procedures, debates, and personalities contributed to a cheerful and positive atmosphere, often amusing us, but never straying from the task at hand. She ensured the integrity of the audio recordings by strictly controlling members’ use of microphones, admonishing those who attempted to speak without a microphone, or who held it at the wrong angle, with a friendly but firm dose of humour.

A preliminary report of Congress action on the decisions and appointments of the Nomenclature Section was published on 14 August, 16 days after the closing ceremony of the Congress (Turland & al. in Taxon 66: 1234–1245. 2017 https://doi.org/10.12705/665.16). It includes a tabulation of the published proposals, specifying how the Section acted on each and detailing amendments and new proposals approved upon motions from the floor. It also includes the membership and full report of the Nominating Committee, approved by the Section and thereby electing members of the Permanent Nomenclature Committees for the period 2017–2023, the Rapporteur-général and Secretary for the next International Botanical and Mycological Congresses, respectively, as well as the Congress resolution ratifying the Section’s decisions, none of which are reproduced here. The main result of the Section’s discussions and decisions is the *Shenzhen Code*, which was published in print as *Regnum Vegetabile* 159, on 26 June 2018 (Turland & al. in Regnum Veg. 159. 2018). It was also published online, on 27 June 2018 (https://doi.org/10.12705/Code.2018). The Appendices of the *Code* are published as a continuously updated, online database, hosted by the Smithsonian National Museum of Natural History (https://naturalhistory2.si.edu/botany/codes-proposals/).

We believe that the present full report of the Shenzhen Nomenclature Section conveys a true and lively picture of the event, retaining the atmosphere of goodwill and humour that infused the meeting. It is primarily based on the electronic audio and video recordings and the transcript that was prepared from them (see below). Where necessary, in case of doubt, these sources were supplemented by the comment slips submitted by almost all of the speakers and scanned into PDF (portable document format) files. All proposals, amendments, motions, and voting results were checked against the published preliminary report of the Section, which itself was based on detailed records made by Anna Monro and notes made by Nicholas Turland. Whenever there was any doubt, the original audio or video recordings were consulted. We are therefore confident that the record published here is accurate and complete.

Before it was edited into its present form, this report went through a succession of stages. The audio recordings were professionally transcribed by Pacific Transcription, Indooroopilly, Australia and supplemented, cross-checked, and edited by Anna Monro. The edited version of the transcript was then heavily re-edited by Heather Lindon and Helen Hartley, to convert it into a report format. At the same time some portions were rearranged to ensure that the report reflects the sequence of relevant provisions in the *Code* even when the order of the debates differed. Deviations from the chronology of events are indicated in the text by italicized bracketed notes. The resulting report was then further edited by Sandra Knapp, Anna Monro, and Nicholas Turland.

As in the case of previous nomenclature reports, which the present one follows in style and general layout, the spoken comments had to be condensed and at least partly reworded, while at the same time carefully retaining the evidently intended meaning of the speakers. Indirect speech has been used consistently. Additions by the authors of this report are placed between square brackets; they include explanatory or rectifying notes, records of reactions of the audience, and reports on procedural actions. As in previous reports, the index to speakers has been integrated with the list of registered members of the Section, as Appendix C.

There were 155 registered members of the Section representing 30 countries, of whom 71 carried 427 institutional votes from 166 institutions in 41 countries, making a total of 582 possible votes representing 44 countries. This was a 22% and 24% smaller attendance compared with Vienna in 2005 and Melbourne in 2011, respectively, but the number of institutional votes carried was 6.2% and 7.8% higher, respectively, making the total number of votes only 3.0% less. There were eight card votes, in which the proportion of members voting ranged between 70.3% and 79.4%. For further details, see the preliminary report of Congress action mentioned above. For a full list of the institutions entitled to vote in Shenzhen, see Appendix B.

Three of the most significant decisions made by the Nomenclature Section in Shenzhen were the establishment of a framework for the future registration of algal and plant names (see Art. 42) including a permanent Registration Committee, provisions for improved clarity in the governance of the *Code* (the new, much enlarged Div. III), and the extension of governance of nomenclature that solely relates to fungi to the International Mycological Congress. A detailed account of the changes made to the *Code* in Shenzhen can be found in the Preface of the *Shenzhen Code* itself.

We would like to dedicate this report to Vicki A. Funk (1947–2019), a powerhouse in the field of plant systematics and a notable contributor during the Shenzhen Nomenclature Section. A Senior Research Botanist and Curator at the Smithsonian National Museum of Natural History, Vicki was passionate about the fundamental role of collections-based research and an advocate for improving gender and geographical representation in science. Combining an extensive research career specializing in *Asteraceae* with the mentorship of students and tireless service in international professional societies, Vicki’s contributions to our discipline will continue to have a lasting impact.

## Acknowledgements

We thank Pensoft Publishers for agreeing to publish this report as an issue of *PhytoKeys*, and for kindly waiving the open-access fee, as was done for the reports of the previous two Congresses (see notes below). Our thanks also go to the International Association for Plant Taxonomy (IAPT) for financially supporting the transcription of the recordings of the Nomenclature Section. We are grateful to Eva Kráľovičová (née Senková) and Matúš Kempa (IAPT Central Office, Bratislava) for notification and distribution of institutional votes and for providing the relevant statistics; also to Mung Seng Chua (Fairy Lake Botanical Garden, Shenzhen) for expertly organizing the audio and video recordings of the Section and for kindly providing the Bureau of Nomenclature with copies of the digital files. We also thank Konstanze Bensch (Botanische Staatssammlung München and Westerdijk Fungal Biodiversity Institute), Vicki Funk (deceased, Smithsonian National Museum of Natural History), Pierre-André Loizeau and Michelle Price (Conservatoire et Jardin botaniques de la Ville de Genève), and Li Zhang (Fairy Lake Botanical Garden) for their help in various ways; as well as the multitude of local helpers in Shenzhen, who helped with microphones, recording, projection and comment slips. Finally, we thank Patrick Herendeen (Chicago Botanic Garden), John Wiersema (Smithsonian National Museum of Natural History) and Karen Wilson (Royal Botanic Gardens and Domain Trust, Sydney) for their helpful reviews of the manuscript of this report.

**Kew, London, Canberra & Berlin, 23^rd^ March 2020**

Heather L. Lindon, Helen Hartley, Sandra Knapp, Anna M. Monro & Nicholas J. Turland

## Notes

The figures given in parentheses for each proposal in this report correspond to the result of the preliminary mail vote (Yes: No: Editorial Committee: Special[-purpose] Committee).

The reports of the Nomenclature Sections of the previous two International Botanical Congresses (IBC) were published as follows:

XVII IBC, Vienna, 2005: Flann & al. in PhytoKeys 45: 1–341. 2015 https://doi.org/10.3897/phytokeys.45.9138

XVIII IBC, Melbourne, 2011: Flann & al. in PhytoKeys 41: 1–289. 2014 https://doi.org/10.3897/phytokeys.41.8398

A list of previous International Botanical Congresses and consequent editions of the *Code*, with references, was published by Turland, The Code Decoded: Chapter 14, Table 11. 2019 https://doi.org/10.3897/ab.e38075

## XIX International Botanical Congress, Shenzhen, 2017 – Nomenclature Section

### Bureau of Nomenclature

*President*: Sandra Knapp

*Vice-presidents*: Renée H. Fortunato, Werner Greuter, De-Zhu Li, John McNeill, Gideon F. Smith, Karen L. Wilson

*Rapporteur-général*: Nicholas J. Turland

*Vice-rapporteur*: John H. Wiersema

*Recorders*: Yun-Fei Deng, Li Zhang

*Recorders’ Assistant*: Anna M. Monro

### Tellers

Heather L. Lindon, Melanie Schori, Gustavo Shimizu, Yi-Hua Tong

### Nominating Committee

Vicki A. Funk (Secretary), Alina Freire-Fierro, Dmitry V. Geltman, David L. Hawksworth, Regina Y. Hirai, Jin-Shuang Ma, David J. Middleton, Gideon F. Smith, Kevin R. Thiele

**Figure 1.**
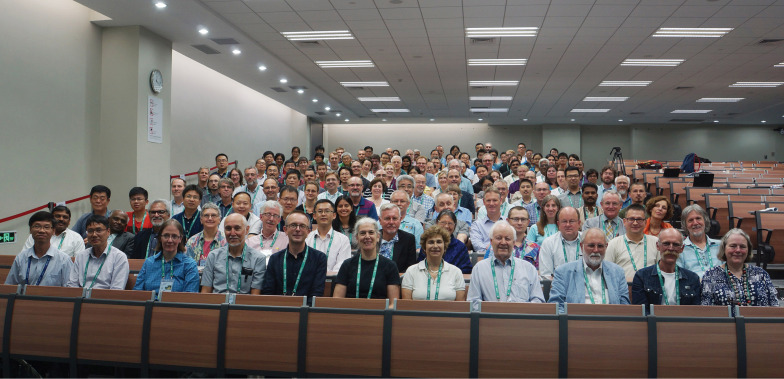
The Nomenclature Section of the XIX International Botanical Congress, Shenzhen, China, 20 July 2017. The Bureau of Nomenclature is seated on the front row (left to right): Li Zhang, Yun-Fei Deng (Recorders), Anna M. Monro (Recorders’ Assistant), John H. Wiersema (Vice-rapporteur), Nicholas J. Turland (Rapporteur-général), Sandra Knapp (President), Renée H. Fortunato, John McNeill, Werner Greuter, Gideon F. Smith, and Karen L. Wilson (Vice-presidents). – Reproduced by permission of the photographer, Li Zhang, Fairy Lake Botanical Garden.

### Monday, 17^th^ July 2017, Morning Session

#### Welcoming comments

**Knapp** thanked all present for coming, acknowledging the difficulties many people had in getting to the conference with delays and cancelled flights. She expressed her appreciation for the effort that people from all over the world had made to come to the session. She introduced herself as the President of the Bureau of Nomenclature and welcomed everyone to the Nomenclature Section of the XIX International Botanical Congress (IBC). She noted that Shenzhen was a vibrant city, and that this Botanical Congress, including the Nomenclature Section, was a really exciting time for Shenzhen.

Knapp reminded delegates that 2017 was the 150^th^ anniversary of the very first *Lois de la Nomenclature Botanique*, written by Alphonse de Candolle, which marked the start of the modern *Codes*. She likened this Section to a birthday party for the *Code.* “We are following de Candolle’s long tradition of modernizing the *Code* and making it fit to facilitate the science that it is supposed to support. What we are doing here during this week is more than just arguing about some small changes. We are facilitating science and making de Candolle’s vision of having rules that govern the naming of plants, algae and fungi become real and good.”

Knapp then introduced Professor De-Yuan Hong, Academician of the Chinese Academy of Sciences, Institute of Botany, Chinese Academy of Sciences, Beijing, and an Honorary President of the International Botanical Congress.

**Hong** thanked Knapp and warmly welcomed everyone to the XIX IBC on behalf of the Organizing Committee. His 80^th^ birthday was last December or January and he had been a plant taxonomist for over 50 years, so he had gained a lot of experience.

“The *International Code of Botanical Nomenclature* is like a cornerstone of a building. It’s very important. You know, we people have nationalities. We cross the border; we should have a visa. But algae, fungi, plants, they don’t need a visa. They can cross borders freely, so many fungi, algae and plants are very widespread. For example, *Pinus sylvestris* is distributed from Europe to East Asia. They cross many nations. In the world we have thousands of nationalities and thousands of local languages. If we don’t have the *International Code of Botanical Nomenclature*, how can we communicate about plants, algae and fungi? The *Code* is very important.”

Hong noted that the Section was an important gathering of world-famous experts working on algae, fungi, and plants who would discuss, debate and improve the *Code*. It was fortunate to have young taxonomists in attendance, as the Congress provided young taxonomists with a wonderful opportunity to take part in discussion and debate and this was good for the future of taxonomy.

He thanked the Peking University Business School (PHBS) and the many volunteers. Finally, he expressed his wish that everyone should have a wonderful time in Shenzhen, and a very happy time in discussion and debate resulting in an improved and more concise *Code* that was more easily understood and used.

**Knapp** thanked Professor Hong and reiterated her thanks to the PHBS. Knapp also thanked the local organizers Li Zhang, Yun-Fei Deng, Su-Zhou Zhang, Shi-Xiu Feng, Shan Li, Mung Seng Chua, Hui Dong, and Qin Zuo. She thanked the runners with the microphones for the first session, Scarlett Lee and Chan Huang, stressing that prompt microphone returns would keep the Section on track.

Knapp provided the e-mail address for sending comments to ensure that they would be properly recorded. She went through the daily schedule, noting that an 8 o’clock start was quite early for many people, but she promised a later start in the second half of the week if the Section was very efficient. There would be coffee/tea breaks in the morning and afternoon, and lunch in the middle of the day, providing an opportunity for people to meet and chat. Knapp stressed that if groups of people wanted to get together and have a meeting to discuss a new proposal to the *Code* there were breakout meeting rooms for them to use.

Knapp noted that she was based at the Natural History Museum in London, UK, which dealt with algae, fungi, and plants. She introduced the Rapporteur-général Nicholas Turland and the Vice-rapporteur John Wiersema. The Recorders were introduced: Li Zhang and Yun-Fei Deng. Anna Monro was introduced as the Recorders’ Assistant.

Knapp went on to introduce the Vice-presidents she had appointed in case she should be incapacitated:

Renée Fortunato, Werner Greuter, De-Zhu Li, John McNeill, Gideon Smith, and Karen Wilson.

Knapp explained that another one of the big jobs in a Nomenclature Section was being a Teller and collecting and counting card votes, especially when the votes were very close. The Tellers for this Section were Heather Lindon, Melanie Schori, Gustavo Shimizu, and Yi-Hua Tong.

The President went on to mention notable absences at this Nomenclature Section, including Lorelei Norvell, the past Secretary of the Committee for Fungi. Knapp asked everyone to join her in wishing Lorelei the best of health. Also absent was Vincent Demoulin, on the Committee for Fungi and the Editorial Committee of the last seven *Codes* since the Leningrad Congress [1975]. Judy Skog, the past Secretary of the Committee on Fossils, and Sebsebe Demissew, Chair [Convener] of the Special Committee on Institutional Votes, were also unable to attend.

Knapp noted that Larry Dorr (Smithsonian, USA) had kept an *in memoriam* list of botanists who had passed away between Congresses. She asked that the attendees check the list and add any missing individuals, adding their place of work and what they worked on. There was a special mention for the passing of Professor Zheng-Yi Wu, the “father of botany in China” and the driving force for the *Flora of China*. Dan Nicolson, who had served for many years as Secretary of the General Committee and had been President of the Nomenclature Section at the Vienna Congress [2005], had also passed away. Knapp noted that if Nicolson had told her how difficult the President’s job was, she would have never taken it on!

Other notable deaths were: Dick Brummitt, with many years of service as the Secretary to the Committee for Spermatophyta, which later became the Committee for Vascular Plants; Paul Silva, who had many years of service as the Chair of the Committee for Algae and who was the driving force for the Nomenclator for Algae, *Index Nominum Algarum*; Pierre Compère, who had many years of service as the Secretary to the Committee for Algae; Ed Voss, who was the Rapporteur-général at the Sydney Congress [1981] and the Vice-rapporteur at Seattle [1969] and Leningrad [1975]; Walter Gams, the past Secretary of the Committee of Fungi; and Bill Chaloner, who had served for many years on the Committee on Fossils. Gill Perry, who was on the Committee for Spermatophyta, later Vascular Plants, had passed away suddenly on the way home from the Melbourne Congress [2011]. Finally, Jim Reveal, a specialist on suprageneric names, had passed away more recently.

The President stated that there were 397 proposals to change the *Code* to be discussed, the largest number of proposals since the Stockholm Congress of 1950, where there had been 550 proposals, largely because nobody had really changed it before. She stressed the importance of being brief and to the point, so that the Section could keep its wheels going around for five days.

The President noted that the Section would be recorded for transcription, as had been done in the last several Nomenclature Sections. For this reason, Knapp informed the Section of the importance of being very clear in speaking, and for those who wished to speak to ensure they waited for the microphone to get to them. In addition, every time anyone spoke, they should say their name and their home base, because that helped the people who were transcribing the recordings to know who had commented. Knapp reiterated the importance of being brief, concise and sticking to the point, noting that any deviation would be shut down. She reminded the Section that the aim was to facilitate the nomenclature of algae, fungi, and plants, as mentioned by Prof. Hong in his opening speech.

Knapp asked that all speakers also submit their comments in writing in case there were problems understanding people, or problems with the recording. Knapp noted that Greuter had once said, “the difference sometimes between what people say and what they write down is quite interesting” and those differences always provided great amusement later. In filling out the comment slip, Knapp asked that speakers write their family name in capital letters and their given name in small letters. In addition to the slips, Knapp repeated the e-mail address to which comments could be sent, noting that any comments sent by e-mail should cite the speaker’s name, the fact that the e-mail contained a comment, and the Article to which that comment referred.

**Turland** noted, however, that all complaints were banned!

**Knapp** agreed that absolutely no complaints should be sent to the e-mail address provided. She went on to quote a translation from de Candolle’s *Lois de la Nomenclature Botanique*, proposing it as the motto for the next five days. “Meanwhile, let us perfect the system introduced by Linnaeus. Let us try to adapt it by the continual and necessary changes of our science. Let us attack abuses and negligence and come to an understanding on debated points if possible. We shall thus have paved the way for the practice of science for many years to come.” She encouraged delegates to keep de Candolle in the back of their minds during the week so that the Section would have a good week and get a lot done.

#### Introductory business

**Turland** wished to talk about voting at the Nomenclature Section, stating that this would be done by a show of hands. When the President asked, “all those in favour?”, members should raise their hand if they supported the proposal. The President, Rapporteurs, Recorders and Recorders’ Assistant would ascertain the majority’s support or otherwise. The President would then say, “all those against?” and those voting for the proposal should take their hand down and those against the proposal should raise their hand. Turland emphasized that it was perfectly acceptable to oppose proposals and that members should vote according to how they felt about the proposed changes. He reminded members that they also had the option to abstain. He cautioned members against raising voting cards, as had been the previous custom, because from the front of the auditorium it appeared as a great ocean of coloured cards hiding the people behind them and making it difficult to count votes. If a required majority could not be reached by other means, a card vote would be held. This would usually be called for by the President. The Tellers would go around the room with boxes and those delegates who wished to vote should deposit one of the small cards from the sheets provided into the relevant coloured box: the red box for “no” votes and the green box for “yes” votes. Each attendee could cast an anonymous personal vote, using numbered cards detached from the white sheets with “P” on them. If someone was carrying any institutional votes, they could also deposit one of the small cards from each of the institutional voting sheets.

Once all cards had been deposited, the Tellers would take the boxes into the room behind the screen. A spreadsheet had been prepared to enable the vote, for or against the proposal, to be quickly calculated. The Section would carry on with business while the Tellers were counting and, once that item of business was finished, the Tellers would come back and the results would be announced. Turland noted that because card votes were time-consuming, they should only be used when necessary, usually when it was too difficult to tell if there was a sufficient majority. Card votes would generally be used for proposals where there was a lot of significance resting on the result.

Turland explained that the voting procedures for this Section were in accordance with what was done at the Melbourne Congress in 2011. Any proposal to amend the *Code* that received 75% or more “no” votes in the preliminary guiding mail vote was automatically ruled as rejected, unless a proposal to discuss it was moved by a member of the Section and supported or “seconded” by another five members. The preliminary guiding mail vote had been conducted in the earlier part of the year and the results were in the information pack provided to delegates [Turland & al. in Taxon 66: 995–1000. 2017 https://doi.org/10.12705/664.25].

Any proposal to amend the *Code* that concerned only Examples, excluding voted Examples, or the Glossary would automatically be sent to the Editorial Committee unless any delegate wished to make a proposal from the floor to discuss it and was supported by five other delegates. In the case of Examples, the Editorial Committee would review them and decide whether or not they were correct, check them for accuracy and decide whether they improved the *Code*. The proposal from the Bureau was to refer these changes to the Editorial Committee automatically.

A simple majority, more than 50% of votes cast, would be required for all decisions except the following: a qualified majority, at least 60%, would be required for accepting a proposal to amend the *Code* or accepting a motion to end discussion and proceed to a vote. The latter, often referred to as “calling the question”, arose when a discussion had been going on for some time and somebody in the Section felt that everything that needed to be said had already been said and delegates should just vote.

A qualified majority would also be required for a new proposal to amend the *Code*, in other words, a proposal that had not previously been published in *Taxon* and did not appear in the synopsis of proposals, or an amendment to an existing proposal introduced at the Nomenclature Section by a member of the Section. The former was called a “proposal from the floor” of the Section. This may be done only when supported by five other members. Proposals from the floor would be dealt with on the last day of the Section after all the published proposals had been dealt with.

Turland then moved that these procedures be adopted for the Nomenclature Section at the Shenzhen Congress.

[The **proposal** was supported by five **seconders**.]

The proposed procedures were **accepted** unanimously.

**Knapp** confirmed that these would be the procedures by which delegates would vote at the Shenzhen Congress. She advised that these voting procedures could be found in the [proposed] revised Division III, which was in the set of documents available to the Section. The President reminded the Section that proposals from the floor to change the *Code* had to be presented in advance to the Bureau of Nomenclature, i.e. to either of the two Rapporteurs, the two Recorders or the President herself. The changes had to be submitted in writing, either neatly written out on a piece of paper or by e-mail, by no later than the end of business on Thursday. Knapp emphasized that the Thursday deadline was a hard-and-fast rule. Knapp informed the Section that any member could propose a friendly amendment to any proposal under discussion. If accepted by the original proposer as a friendly amendment it would not be necessary to vote on it, nor would support be required from other members. However, if an amendment was not accepted as friendly [i.e. it was considered unfriendly], seconders and a vote would be required. The President gave the following example, “if John has made a proposal and you want to make a friendly amendment that he should use ‘the’ instead of ‘a’, as the definite versus the indefinite article, then you can say, ‘I have an amendment; would this be considered friendly?’ and John can say either ‘yes’ or ‘no’.”

**Turland** then asked the Recorders’ Assistant to show the list of names of members of the proposed Nominating Committee on screen. He informed the Section that the Nominating Committee was proposed by the President of the Nomenclature Section in consultation with the other members of the Bureau of Nomenclature. The role of the Committee was to work with the Secretaries of the seven Permanent Nomenclature Committees who had already prepared lists of potential members for the next six-year period between this Congress and the next IBC in 2023.

These lists would include existing members of those committees who were able and willing to continue to serve, and in some cases new members when existing members were retiring. The Nominating Committee’s job was to review and to scrutinize the lists and ensure that there was geographical balance of membership on them and to find additional members if the Secretaries of the Permanent Nomenclature Committees were not able to fill all the vacancies.

The seven Permanent Nomenclature Committees were listed as the Committee for Algae, the Committee for Bryophytes, the Committee on Fossils, the Committee for Fungi, the Committee for Vascular Plants, the Editorial Committee and the General Committee. Turland reminded the Section that there were two proposals to this Nomenclature Section to add two additional Permanent Nomenclature Committees: the Registration Committee and the Committee on Institutional Votes. As it was not known whether these proposals would be accepted or rejected, the Nominating Committee would also produce a list of proposed members for those two committees. Acceptance of those lists would then be subject to the proposals being passed.

The Rapporteur-général pointed out that more information about these Permanent Nomenclature Committees could be found under Div. III Prop. B, the proposal for a new Division III from the Special Committee on By-laws for the Nomenclature Section, in which Provision 7 provided some detail regarding the roles of the Permanent Nomenclature Committees.

Turland closed by advising that anybody who wished to serve on a Permanent Nomenclature Committee should talk to the Secretary of the Nominating Committee and tell her that they were able and willing to serve. He asked Funk to stand up so that attendees would know whom to approach.

The proposed members of the Nominating Committee were: Vicki A. Funk (Secretary), Alina Freire-Fierro, Dmitry V. Geltman, David L. Hawksworth, Regina Y. Hirai, Jin-Shuang Ma, David J. Middleton, Gideon F. Smith, and Kevin R. Thiele.

**Knapp** proposed, both as the President and the person who came up with the slate for the Nominating Committee, that the Nominating Committee be accepted as proposed.

[The **proposal** was supported by **five seconders**.]

The **Nominating Committee** was **accepted**.

**Knapp** went on to urge anyone, particularly younger scientists, to approach Funk over the course of the meeting to join a committee as a way of getting involved in the community.

**Turland** moved that the Section ratify the *Melbourne Code* [2012], including its Appendices. He pointed out that once the *Melbourne Code* was accepted by the Section, all the amendments that resulted from the Melbourne Congress [2011] became fixed and would form the basis of discussion. Further amendment of the *Code* was then only possible through the Nomenclature Section of the Shenzhen Congress.

The **ratification** of the ***Melbourne Code*** was **accepted**.

**Knapp** summarized the work ahead for the Section: out of the 397 proposals, 87 were ruled as rejected in the preliminary guiding mail vote and would not be discussed unless reintroduced from the floor with five seconders. A further 65 proposals concerned only Examples, or the Glossary, and were referred automatically to the Editorial Committee. This left 245 proposals for discussion, which the President still considered a large number and she reminded the Section of the need for efficiency.

Knapp proposed that 30 minutes be set aside for a general discussion on Tuesday morning on the governance of nomenclature, especially the governance of the nomenclature of fungi, because many of the attending vascular plant taxonomists may not have investigated the matter in detail. Knapp referred to the paper from *IMA Fungus* provided in the attendees’ introductory packs [Miller & al. in IMA Fungus 8: (9)–(11). 2017 https://doi.org/10.1007/BF03449429]. The Committee for Fungi had strongly supported the proposed changes in the governance of fungal nomenclature. Knapp urged delegates to read the governance documents so that an informed discussion could take place on Tuesday morning; a similar approach had been taken in Melbourne for discussion of the *Acacia* situation. The proposers of the changes would answer questions at the Tuesday discussion, but the vote on these issues would be held later. Knapp felt it was important to have the time to discuss and then think about the proposals as a community in a collective way, even for those who were not associated with fungal nomenclature.

**Turland** continued with the introductory business by explaining the text that would be projected on the screen during the proceedings. On the right-hand side would be the *Code* itself, in the form of a Word document extracted from the published *Melbourne Code*. The left-hand side of the screen would show the relevant position in the running order of proposals, extracted from the synopsis of proposals published by the Rapporteurs [Turland & Wiersema in Taxon 66: 217–274. 2017 https://doi.org/10.12705/661.36]. The Recorders and Anna Monro would keep those two documents current and with Track Changes activated in the proposals text, so that amendments could be added. In this way the Section would be able to see exactly what was being discussed and it would be clear what was being voted on. Similarly, the *Code* could be amended if proposals were made from the floor.

The order for discussion was adjusted by the Rapporteurs with relevant proposals grouped together. In some cases, if one proposal passed, other proposals might be dependent upon it. In other cases, if one proposal was defeated, others would automatically be defeated. Therefore, it made sense to discuss them together. Occasionally an article would appear out of sequence; this reordering was deliberate on the part of the Rapporteurs.

In general, for about 90% of the published proposals, the current *Code* would be quoted verbatim with additions to the wording in boldface and deletions in strikethrough. In about 10% of cases the proposal would not quote the wording of the *Code* but merely refer to an article or would be a General Proposal that said, “throughout the *Code* use the following terminology.” In those cases, the relevant article in the *Code* itself would be displayed on the screen.

Finally, the Rapporteur-général reminded the Section that the Editorial Committee was mandated to adjust the wording of accepted proposals. They may also put an accepted proposal into the *Code* in the most appropriate place. Although the exact numbering and wording of an accepted proposal may not be identical to what was passed at the Section, the meaning would not be changed by the Editorial Committee. Changes would only be editorial, e.g. wording, punctuation, grammar and syntax, to ensure an accepted proposal was consistent with the rest of the *Code*.

**Knapp** wished to recognize Werner Greuter, one of her Vice-presidents, who was not in attendance when she first introduced him. She asked him to stand and be recognized, which he did to general applause.

**Redhead** queried a point of order regarding proposals rejected by 75% of the mail vote. He wanted to know at what point they could be resurrected for discussion, i.e. during their article running order or on the last day of the Nomenclature Session.

**Knapp** explained that those proposals would be included in the running order. If a delegate could get five seconders, they could bring them back to the floor for discussion.

**Turland** emphasized that such proposals would not be skipped over, they would be mentioned when they came up in the running order.

#### General proposals

**General proposals, Prop. A** (16: 45: 3: 3), **Prop. B** (11: 51: 2: 3), **Prop. C** (8: 54: 2: 3), **Prop. D** (8: 54: 2: 3), **Prop. E** (11: 51: 2: 3), **Prop. F** (14: 48: 2: 3), **Prop. G** (15: 46: 2: 3), **Prop. H** (16: 46: 2: 3) and **Prop. I** (12: 50: 2: 3)

**Turland** introduced this group of proposals to replace different words of terminology in the *Code*, noting that it was not a matter of simply replacing these terms in the *Code*, sometimes they had other meanings. For example, the word “deposited” and the word “listed” were used in different senses in the *Code*, so the specific sense of the word being replaced had to be clear. In addition, some of the terms did not appear in the *Code*, for example “name and type”.

**Hawksworth** provided some background, saying the point of these proposals was to reflect decisions already made and published by the International Committee on Bionomenclature to facilitate communication across biology. He added that having spent a week trying to teach a course on nomenclature in Beijing some of these proposals were especially important, and he was pleased to see that at least some of them had got past the first gate. He took this opportunity to raise the issue of the small number of people who had responded to the mail vote, particularly for proposals relating to groups like mycologists.

**Knapp** asked Hawksworth to stick to the general proposals under discussion and asked if anyone else wanted to speak on this set of proposals.

**Barrie** pointed out that these proposals would change terminology that had been consistently applied in botany for decades and that it would be very difficult for people to change. He argued that people’s thought processes would have to change in mid-course and result in a timeline where things split. He argued for consistency over time, stating that he was opposed to these changes in the *Code*.

**Knapp** reminded Barrie to fill in the comment form. As there were no other objections to voting on this series of proposals she suggested proceeding to a vote and checked with Turland if these would be voted on in order.

**Turland** thought they should be voted on individually.

**Knapp** agreed to vote individually on those that were not rejected in the mail vote.

**General proposals, Prop. A** was **rejected**.

**General proposals, Prop. B, Prop. C, Prop. D** and **Prop. E** were **rejected** based on the **mail vote**.

**General proposals, Prop. F, Prop. G, Prop. H** and **Prop. I** were **rejected**.

**General proposals, Prop. J** (63: 3: 1: 0)

**Turland** noted that Prop. J was essentially editorial. The Rapporteurs had mentioned in their comments that this was something the Editorial Committee had considered doing editorially in the *Melbourne Code*, but they had felt that a mandate from the Section was required. Accepting the proposal would provide that mandate. The proposal was to replace “based on a generic name” with “formed from a generic name” in the articles listed in the proposal. The proposed change was to distinguish between this sense of “based on” and the sense of based on a basionym or replaced synonym, and it was not the intention of the proposal to change the latter sense. In this context, based on a generic name meant formed from a generic name. For example, *Asteraceae* was formed from *Aster*, it was not based on the genus name *Aster*.

**Prop. J** was sent to the **Editorial Committee**.

**General proposals, Prop. K** (8: 56: 3: 0) and **Prop. L** (3: 62: 2: 0) were **rejected** based on the **mail vote**.

#### Preamble

**Preamble, Prop. A** (3: 63: 1: 0) was **rejected** based on the **mail vote**.

#### Article 4 and Recommendation 4A (new)

**Art. 4, Prop. A** (6: 57: 3: 0), **Prop. B** (6: 57: 3: 0), **Prop. C** (9: 54: 3: 0) and **Prop. D** (6: 57: 3: 0), **Rec. 4A (new), Prop. A** (6: 57: 3: 0) and **Prop. B** (9: 53: 4: 0) were **rejected** based on the **mail vote**.

#### Article 5

**Art. 5, Prop. A** (3: 63: 0: 1) and **Prop. B** (1: 62: 3: 1) were **rejected** based on the **mail vote**.

#### Article 6

**Art. 6, Prop. A** (18: 1: *48: 0)

**Turland** explained that for Art. 6 Prop. A, the Rapporteurs’ comments indicated that an Editorial Committee vote [marked with an asterisk above] in the mail ballot would have a special meaning. He asked the proposer, Greuter, if the Rapporteurs’ changes could be considered as a friendly amendment.

**Greuter** said that as far as he recalled, the **amendment** was **friendly**, so he **accepted**.

**Turland** went on to paraphrase the Rapporteurs’ comments, saying Prop. A would add a clause to Art. 6.1 specifying that only material that was effectively published could be taken into account for the purposes of the *Code*. This mostly reflected current practice and the requirement of effective publication was explicitly mentioned in some provisions but not in others. This proposal would place a general provision in Art. 6.1 that would make the explicit provisions in other parts of the *Code* superfluous, and was essentially an editorial improvement simplifying the *Code*.

However, the Rapporteurs were concerned that placing the new provision in Art. 6 would make it apply throughout the *Code* and could prevent specimens, which were not effectively published material, from being taken into account. If specimens could not be included, there could be no types. This concern could be removed by replacing “material” with “text and illustrations” but there also remained a worry that there could be other unwanted consequences.

Turland suggested that an alternative would be to place the new rule following Art. 32.1, which would therefore explicitly limit it to Art. 32–45 on valid publication. This would be worded in Art. 32.1*bis*, “For the purposes of Art. 32–45 only material [or: text and illustrations] that is [are] effectively published is [are] taken into account.”

**Greuter** reiterated that he accepted this as a friendly amendment and clarified that what was being discussed was the amended version of the proposal with “text” instead of “material”. As a comment to the general statement of the Rapporteur-général, he suggested that this be accepted and thereby sent to the Editorial Committee to ensure that no negative consequences were thereby effected.

**Turland** asked for clarification if “material” should be crossed out and “text and illustrations” be accepted.

**Knapp** confirmed this and suggested that the Article be moved to Art. 32.1*bis*.

**Greuter** pointed out that moving the Article to Art. 32 would be considered an **unfriendly amendment**.

**Knapp** agreed that the friendly amendment was to cross out the words “material” and replace that with “text and illustrations” but that this would be placed at the end of Art. 6.1. She asked if the Rapporteurs were proposing a second amendment.

**Turland** noted that the Rapporteurs had made two suggestions. One was to replace “material” with “text and illustrations”, which the proposer has accepted as a friendly amendment. They had made a second, alternative suggestion, in the synopsis in the Rapporteurs’ comments, which was to move it to Art. 32. He told the President that now only the first suggestion was under discussion.

**McNeill** emphasized Greuter’s additional point, the proviso that the Editorial Committee had to ensure that this had no negative effects elsewhere in the *Code*. The Editorial Committee was not totally bound by the outcome, it had the option to fiddle with it.

**Hawksworth** expressed concern about including illustrations because there were cases in fungi where unpublished drawings made by the authors were with collection material in herbaria and had been used for typification in the past. They were part of the original material and they were illustrations, but they were not published. He felt the amendment to specify text was fine but did not want illustrations included.

**Knapp** asked if he was proposing an amendment.

**Hawksworth** proposed the amendment to leave “illustrations” out of the text.

**Knapp** asked Greuter if the amendment to strike out the word “illustrations” and just have “text” was accepted as friendly.

**Greuter** stated that while there were cases in which Hawksworth was right, there were others in which he was not. Though illustrations could serve in place of a description, it had never been accepted that these could be unpublished illustrations. To serve as a surrogate for a validating description, an illustration must be effectively published. He added that if the Section transferred this to the Editorial Committee, the Editorial Committee would have more time, leisure and skill than the Section to examine the consequences and come up with the best possible solution, including placement. [The **amendment** was considered **unfriendly**.]

**Levin** asked if there was a seconder for the amendment to strike “illustrations”.

**Knapp** explained that, as it was not accepted as a friendly amendment, it was still under discussion.

**Turland** addressed the proposer regarding the wording “text and illustrations”. He noted that there was an implication that unpublished illustrations which served as types would not be taken into account if the wording was changed to “For the purposes of this *Code*, save specified exceptions, only text and illustrations that are effectively published are taken into account”.

**Greuter** responded that it should not imply that and, if this proposal was accepted, the Editorial Committee had the mandate to ensure that it did not.

**Turland** summarized Greuter’s explanation by saying that when there existed “text and illustrations” those should only be taken into account when they were effectively published and that it was clear that types which were unpublished illustrations should still be able to serve as types under the *Code*. The Editorial Committee would ensure that no such implication to the contrary was included in the *Code*.

**Redhead** reminded the Section that no one had seconded the proposal to take out the word “illustrations”.

**Knapp** asked if there were any seconders to take out the word “illustrations”. [The **amendment** was supported by **five seconders**.] Knapp then explained that there would be a vote on the unfriendly amendment of striking the word “illustrations” from the amended proposal, saying that it was not a change to the *Code*, but rather a change to an amendment which required a 50% majority to be accepted. [The **amendment** was **rejected**.] Knapp noted that what was now under discussion was the amended Art. 6 Prop. A, which read: “Effective publication is publication in accordance with Art. 29–31. For the purposes of this *Code*, save specified exceptions, only text and illustrations that are effectively published are taken into account.”

**Applequist** asked the proposer whether he accepted the Rapporteurs’ restriction of this proposal to the purposes of Art. 32–45 and, if so, why was there an objection to putting it in that section?

**Greuter** explained that there were several places where this proposed amendment would work and not all were about valid publication. He suggested that the Editorial Committee, in its infinite wisdom, should be given the opportunity to examine all those places and look at the alternatives of either putting the amended text into Art. 32 as the Rapporteur-général had suggested, or else to add “unpublished” or “not effectively published illustrations” in the other places where it might be relevant.

**Knapp** clarified for the Section that it would now vote on the addition of the words, “For the purposes of this *Code* save specified exceptions, only text and illustrations that are effectively published are taken into account.” The friendly amendment was that, should the proposal be accepted, it would be sent to the Editorial Committee who would have latitude not to include this wording in the *Code* if it were to be seen as disruptive.

**Funk** asked if the Section was just voting to send this proposal to the Editorial Committee.

**Knapp** explained that the Section was voting on accepting it in principle and sending it to the Editorial Committee. She sought confirmation from the proposer [Greuter agreed] and pointed out that this was one of the most complicated proposals and it was unfair to have to discuss it right at the beginning.

**Turland** noted that it was a baptism of fire!

**Knapp** agreed and reminded the Section to ask questions if delegates did not understand what was being voted on.

**Art. 6, Prop. A** was **accepted as amended**.

**Art. 6, Prop. B** (32: 2: 30: 0)

**Turland** explained this was to add cross-references to three Articles, suggesting that it could be sent to the Editorial Committee as it did not change the meaning of the *Code*.

**Art. 6, Prop. B** was sent to the **Editorial Committee**.

**Art. 6, Prop. C** (26: 23: 15: 0)

**Turland** noted that this proposal was more than editorial because it changed the definition of an isonym. If this proposal was accepted, an isonym would refer only to the later usages of a name. Currently two names that were spelled the same and had the same type were both isonyms. Under the new proposed definition, the first validly published of those isonyms would not be an isonym, and only the later usages would be referred to as isonyms. In other words, the later published name spelled exactly alike with the same type would be the isonym. Otherwise the proposal was an editorial adjustment of Art. 6 Note 2.

**Wiersema** suggested that Art. 14 Note 1 would need to be adjusted if Prop. C were accepted.

**Turland** agreed, explaining that Art. 14 Note 1 regarded the earliest and later usages of a name as validly published and as isonyms.

**Gereau** thought the rewording was unclear, less intuitive and harder to understand. He thought the current usage of “later isonym” was parallel to the usage of “later homonym”, making the whole issue much easier to understand as it was. He emphasized that the proposal was de-clarifying and considered it undesirable.

**Soreng** said the advantage of the proposal was that you could declare all isonyms not validly published. The first one was effectively published and valid, therefore it was not an isonym.

**Turland** pointed out that the *Code* already said that later isonyms could be disregarded so one could argue that it was already clear in the *Code*.

**Art. 6, Prop. C** was **rejected**.

**Art. 6, Prop. D** (3: 14: 49: 0) was **automatically** sent to the **Editorial Committee**.

**Art. 6, Prop. E** (18: 34: 15: 0)

**Turland** explained that this related to Art. 53 Prop. A and Prop. B on distinguishing between homonyms and isonyms, converting Art. 6 Note 2 to an Article and broadening the definition of isonym in that Note. Some later usages of a name without exclusion of its type would represent isonyms, whereas the current Note 2 required the later usage to be based on the same type. However, it was not possible to determine this when a name had not been typified. The Rapporteurs were concerned that this could be a difficult rule to apply.

**Greuter** wished to support the proposal because although the Rapporteurs said that the rephrased provision would be difficult to apply, the current provision was also difficult to apply. For instance, in works in which no author citations were used by the author, the same names were reused from Linnaeus and earlier authors and had often been misinterpreted as later homonyms. Those later homonyms might be replaced by replacement names but if in fact they were isonyms, they should not be replaced. Sennikov’s proposal was, in his opinion, a clear improvement on the *status quo* because it authorized us not to multiply later homonyms unduly.

**Applequist** foresaw a difficulty with this proposal because of the thousands of homonyms from the pre-type era and suggested that maybe many thousands of names that were now considered to be later homonyms would be converted to isonyms. Even if the taxa were from different continents, if the author did not mention the older name it would be ruled an isonym because there would be no way to exclude the type otherwise. She was concerned that this proposed change was a rather broad redefinition of isonym.

**Gereau** commented that the addition of the phrase “without exclusion of the type”, when applied to names in an era where types frequently did not exist, made the application of this definition much more subjective than the original definition of isonym and should be rejected.

**Govaerts** expressed concern that with this wording genuine homonyms could be considered isonyms if the author, in eras where there were no types, had not explicitly excluded it.

**Art. 6, Prop. E** was **rejected**.

**Art. 6, Prop. F** (12: 28: 26: 1) was **automatically** sent to the **Editorial Committee**.

**Art. 6, Prop. G** (44: 3: 18: 0)

**Turland** suggested that this proposal could be sent to the Editorial Committee since it merely rephrased the second sentence of Art. 6.4. It concerned the illegitimacy of the name of a family or a subdivision of a family. The Rapporteurs considered that the proposed rewording appeared clearer and more precise, eliminated redundancy and was editorial since it did not change the meaning. It could be sent to the Editorial Committee.

**Art. 6, Prop. G** was sent to the **Editorial Committee**.

[*The Section broke for morning tea*.]

**Knapp** thanked the International Association for Plant Taxonomy (IAPT) who helped support nomenclature, both at the Nomenclature Section and between Congresses with the work of the Permanent Nomenclature Committees and publishing all the proposals to change the *Code*, proposals to conserve and reject, and binding decisions in its journal *Taxon*. She invited those interested in learning more about IAPT to look at the table outside with some copies of *Taxon*. She reminded students in the audience that membership dues in the IAPT were only the cost of two slices of pizza and a beer, [*Laughter*] which she considered really good value. She informed the Section that members of the IAPT could participate in the preliminary guiding mail vote, which itself was part of participation in the grand community event of nomenclature.

**Art. 6, Prop. H** (56: 2: 9: 0)

**Wiersema**, as the principal author on this proposal wished to speak about it. As it was his first opportunity to speak, he first welcomed everyone to the Section. He went on to describe the proposal as adding a phrase to Art. 6.4, explaining that it related to the tradition of conserving the basionyms of names in order to protect other names. This was often done because the basionym was illegitimate, so conservation overcoming illegitimacy would allow the basionym to be used. However, it was not the basionym that was of concern, but rather the name that would otherwise have been based on that name. Such a proposed new combination, because it did not have a legitimate basionym [when it was published], could also be illegitimate by reason of superfluity. A significant number of names in the Appendices were listed as cross-referenced to their basionyms assuming that they were protected because their basionyms were conserved. However, Art. 6.4 states that a name, once illegitimate, remains illegitimate. To overcome that problem, he proposed that a phrase could be added and those names that were incorrectly thought to have been protected by having their basionyms conserved would now, in fact, be protected and would become legitimate. He finished by noting that these names cross all different groups including fungal, bryophyte and vascular plant names.

**Barrie** reiterated that this change conserved the current practice that most people assumed was going on, and if passed would legitimize what was already being done. The proposal was not changing anything, just clarifying and putting foundation into the *Code*.

**McNeill** added that, in fact, the preface to one of the Appendices said that this was the case, contrary to the actual Article, therefore it was extremely important to pass this and do so now.

**Art. 6, Prop. H** was **accepted**.

**Art. 6, Prop. I** (9: 55: 2: 0) was **rejected** based on the **mail vote**.

**Art. 6, Prop. J** (33: 18: 15: 0)

**Turland** noted that there were two linked proposals, Prop. J and Prop. K, which were parallel and should be considered together. They aimed at an improved definition of the terms “basionym” and “replaced synonym” by specifying that a basionym or a replaced synonym did not itself have a basionym.

**Gereau** asked the proposer what problem was being addressed by this proposal. Were there people who thought that a basionym had a basionym or a replaced synonym had one? He stated that he had never, in all the nomenclatural editing he had done, found an author who seemed to be convinced of this.

**Greuter** thought it was perhaps unnecessary because he thought the question had been answered in the proposal itself. The proposal sought to draw a line between what was a replacement name and what was the name of a new taxon. This used to be generally understood, in the old times, when there was a kind of backdoor definition of a replacement name being said to be an avowed substitute. He explained that to be avowed it must be intentional and must be visibly intentional as a replacement name. It was most important when the conditions for valid publication were fulfilled for either category, and made a lot of difference in many cases. He cited the case where in type designation a replacement name could not have a type designated, but a new taxon must have a stated or designated type. He believed it was quite an important distinction to be made and that the *Code* was not currently very helpful in this.

**Lindon** queried what would happen if a name being cited as a replaced synonym was itself a new combination and asked if those replacement names would not be validly published if the new combination, and not the basionym, were cited. She pointed out that this might affect names that had already been published.

**McNeill** thought the proposal would make no change to the situation Lindon had referred to. If an author cited something that was not in fact the real basionym it was not validly published unless it fell under the provisions for new combinations that were exceptions in Art. 41. He failed to see that this would make any difference to the situation regarding publishing a new combination based on something that was not the basionym.

**Govaerts** pointed out that the comment was not about making new combinations but about publishing a replacement name. In those cases, a replaced synonym must be cited and now people may cite a replaced synonym that was already a new combination and not necessarily cite the original basionym. He agreed that if the rules were tightened up so that the replaced synonym must be cited from the original basionym, it would render not validly published all these replacement names where the basionym was not cited, which was not uncommon.

**Turland** suggested this would depend on the date of the replacement name.

**McNeill** said he was only referring to recently published names, [in or] after 1953.

**Turland** agreed that before 1953 it was possible to have an indirect reference to a basionym or a replaced synonym, so in that case it would not be a problem.

**Govaerts** stated that even today people published replacement names citing a replaced synonym that was a new combination, and that this was valid.

[The vote on the proposal by show of hands did not clearly reach the 60% majority required.]

**Kirk** called for a **card vote**.

**Middleton** noted that the institutional votes might make a difference.

This being the first card vote, **Knapp** explained that if the issue was contentious and someone in the Section felt that institutional votes should come into play, then a card vote could be called. The Tellers would get the boxes, the cards would be deployed, and the Tellers would then go away and count the votes.

**Art. 6, Prop. J** was **accepted** based on the **card vote** (332 yes: 172 no; 65.9% yes).

[*The Section voted to discuss Prop. K after the card vote for Prop. J had been counted. The following discussion on Prop. K occurred after that of Prop. L and Art. 16 Prop. B*.]

**Art. 6, Prop. K** (41: 16: 9: 0)

**Turland** noted that this proposal was similar to Prop. J. It did not really change anything in the *Code* but merely made what was current practice more explicit. He reiterated that this did not include the pre-1953 situation, where a reference to a replaced synonym could be indirect, but only 1 January 1953 onwards where for example for a replacement name, you cited what you thought was the replaced synonym but it actually turned out to have a basionym and it was not really a replaced synonym at all. He said that would not be considered validly published. However, he suggested that looking in the *Code* it was difficult to come to that conclusion, and that the proposed wording made this explicit.

**Govaerts** clarified that the comments that had been made earlier [by Lindon and Govaerts] were about Prop. K, who were under the impression that Prop. J and Prop. K were being discussed together. He did not agree with the assessment made by Turland. A replaced synonym could often itself be a new combination. It said in the *Code* that if you published a replacement name you must cite the replaced synonym. In that case, the replaced synonym might be a new combination that was illegitimate, and then authors cited that as the replaced synonym. The *Code* did not say that a replaced synonym could not be a new combination and if the Section were to tighten up the wording then all such names would become not validly published.

**Turland** asked what the situation for such names was in the International Plant Names Index (IPNI).

**Govaerts** replied that in IPNI the replaced synonym was recorded as cited by the author and could be a new combination.

**Turland** wondered if the replacement name in such a case would be considered validly published.

**Govaerts** replied that it would.

**Turland** asked if this was still the case even though the name cited as a replaced synonym was in fact a new combination and not a basionym.

**Govaerts** responded that it would be considered validly published by IPNI because such cases were not uncommon.

**Applequist** said that she found the wording in Prop. K inappropriate compared to Prop. J where it said the basionym did not itself have a basionym. She pointed out that although it was kind of circular, it made sense, but when stating that the replaced synonym did not itself have a basionym, the word “basionym” had not appeared before in 6.11 and this phrase was confusing.

**McNeill** referred to his earlier comments on the two proposals: that they did not change anything. He now wished to correct that so far as Art. 6 Prop. K was concerned. He agreed with Govaerts that in a situation in which a name with a basionym turned out to be a later homonym it may be necessary to produce a replacement name, and the replacement name would have a replaced synonym that was a new combination. It would be ridiculous to expect the ultimate basionym, which itself was legitimate, to be cited as the replaced synonym. He had not realized such cases were as common as Govaerts had explained and agreed that the proposal, as it was worded, would in fact make names that were currently validly published now not validly published. He thought the proposal needed to be reworded as to how one might make clear what a replaced synonym was. However, as currently worded it would be undesirable, so he was now against it.

**Sennikov** felt there was confusion about this proposal because there were two issues involved. One issue was how the authors published a certain nomenclatural action and what they believed they were doing, how they assessed their names and whether they were treated as replaced synonyms or basionyms by the original authors. The second matter was how the names were currently treated. When more data became available, the names were reassessed. This proposal and this Article were about how the cases were dealt with currently, not how the original authors were dealing with them. In his opinion it was perfectly valid.

**Govaerts** suggested that since the consequences would be retroactive, he offered a friendly amendment to add a date in the future for the proposal to take effect.

**Greuter** considered the **amendment unfriendly**.

[The **amendment** was not supported by **five seconders**.]

**Turland** asked McNeill to clarify his previous comment to explain the scenario in which this proposal would be disruptive.

**McNeill** explained that there might be a basionym, in other words a legitimate name, and that somebody published a new combination based on it. That new combination could have been a later homonym for which a replacement name was required. That replacement name would be replacing the later homonym, which itself had a basionym. He believed this was the situation that Govaerts was describing. [**Govaerts** nodded in agreement.]

**Xiang-Yun Zhu** suggested the two cases could be treated separately, that a proposal could provide a clear definition for replaced synonyms that did have basionyms, and a definition for replaced synonyms that did not.

**Xia** added that he thought that most replaced synonyms did have a basionym, which was why a replacement name was required.

**Thiele** offered that while the proposal was intended as clarification, the conversation had been about a change in behaviour. He asked for a statement from the proposer or others that if the proposal were rejected would the Section allow a poor practice to persist? In other words, if there were no change would there be a problem if authors continued to publish as they were?

**Greuter** explained that had he not arrived at five o’clock that morning he would feel confident to give a competent answer, but for now he would leave it as it was. He did add that in one of the cases raised, the creation of a later homonym that was a name at a new rank, such as a variety raised to species, the author usually kept the epithet in the replacement name. In that case, the varietal name should be considered as the basionym and the species name should not be considered a replaced synonym.

**Art. 6, Prop. K** was **rejected**.

**Art. 6, Prop. L** (32: 16: 17: 0) and **Art. 16, Prop. B** (31: 14: 20: 0)

**Turland** noted that Art. 6 Prop. L was grouped with Art. 16 Prop. B and was an editorial cross-reference. He questioned whether the note was at all useful but suggested that if the Section determined that it was useful, both proposals could be sent to the Editorial Committee.

**Nakada**, the proposer, explained that the interpretation of Art. 16 was not unambiguous for him. Art. 16 seemed to him to relate to the spelling of the descriptive names, not their validity. However, in discussion with the editor of the [*Melbourne*] *Code*, he understood that this was not about the spelling but rather the valid status of the name, thus he thought clarification was necessary.

**Gereau** felt that on the surface, the proposal seemed relatively obvious, but its virtue was that it absolutely clarified that the authorship of descriptive names did not change. He suggested that this might prevent frivolous changing of the status of descriptive names just to have one’s name as an author.

**Greuter** said that he was speaking in favour of the proposal but had difficulty with one point: its placement. It clearly referred to suprageneric names, but there were descriptive names at lower ranks for which this would be inappropriate. He suggested, as a friendly amendment, that the qualification “suprageneric descriptive names” be added. [The **amendment** was accepted as **friendly**.]

**Turland** asked Greuter to explain his suggestion. He pointed out that a descriptive name was one defined in Art. 16.1(b) and would always in that context be a name above the rank of family. He asked what other descriptive names there might be lower than the rank of genus.

**Greuter** said that there were generic and species names, or at least their epithets, which were descriptive, and he wished to avoid this ambiguity.

**Turland** did not think the amendment necessary given the definition of a descriptive name.

**McNeill** agreed with the Rapporteur-général, suggesting simply adding, “a descriptive name (Art. 16.1(b))”.

**Barrie** suggested the proposal could be sent to the Editorial Committee, noting that minor details could be taken care of at that point.

**Redhead** recalled some order-level names that were published before any constituent family names and these were determined to be valid descriptive names. He saw this proposal as potentially affecting such names, although he could not recall which names were involved.

**Turland** thought that in such a case, the descriptive name would still be the first valid publication of the descriptive name, so it would not be a name at new rank. He felt the current proposal offered nothing new, just a note to clarify something that was implicit or explicit elsewhere in the rules of the *Code* but was not necessarily obvious.

**Wilson** suggested that the definition of “descriptive names” in the Glossary could be looked at. More detail could be added there rather than referring people to the Articles.

**Turland** thought that was a good suggestion, adding that elsewhere in the synopsis of proposals the Rapporteurs made a similar comment that “descriptive name” could be defined more fully in the Glossary.

**Xiang-Yun Zhu** asked what the difference was between a descriptive name and validly published name, suggesting that this may be more related to Art. 23.6 dealing with defining a Latin name.

**Turland** explained that species-level names were not under discussion and that Art. 23.6 concerned designations that were not to be regarded as species names. Descriptive names as defined in Art. 16.1 were above the rank of family and were not automatically typified. They were not formed from a generic name (like *Asterales* for example), but were names like *Centrospermae* or *Gymnospermae*.

**Xiang-Yun Zhu** asked that the distinction be made clearer in the Glossary under the entry for descriptive name.

**Redhead** spoke up for mycology, noting that there were certain order-level names, possibly listed in *Index Fungorum*, for which there was no appropriate family because the authors had jumped from a genus to an order while using molecular phylogenetic studies, thus coming down from above rather than building up the classification. He felt apprehensive about the whole process.

**Turland** asked for an example of such a name.

**Redhead** responded that he would have to look it up.

**Turland** asked if it was a descriptive name or a name automatically typified.

**Redhead** said it was not intended to be a descriptive name but had to be interpreted as one because there was no family upon which to base it, only a genus.

**Turland** asked if being formed from a generic name made it an automatically typified name.

**McNeill** remembered the case Redhead was referring to, but not the exact example. A name was based on a generic name as far as the word structure was concerned, but a name of an order must be based on a legitimate family name and there was no family name involved. That name would not be validly published unless treated as a descriptive name. He imagined that this was an exceptional circumstance that could be resolved by conservation.

**Turland** pointed out that this example was based on a former wording of Art. 16 and with the current wording it would not be a problem.

**Funk** wanted to know if the vote was to refer the proposal to the Editorial Committee.

**Knapp** respectfully pointed out that everything went to the Editorial Committee if it was approved.

**Funk** reminded the President of the option to vote to refer proposals to the Editorial Committee.

**McNeill** explained the difference between a proposal concerning an Article being approved and a proposal concerning a Note being sent to the Editorial Committee. In the first case, the Editorial Committee was bound to include it, making sure that it was consistent with the rest of the *Code*. However, if a Note was sent to the Editorial Committee, it was an instruction to the Editorial Committee to consider making this explanation, but it was not mandatory because it was within the jurisdiction of the Editorial Committee to not include Notes.

**Redhead** wished to withdraw his objection because it was based on the earlier *Code*.

**Art. 6, Prop. L** and **Art. 16, Prop. B** were sent to the **Editorial Committee**.

[*The sequence of events now reverts to its chronological order*.]

**Art. 6, Prop. M** (8: 40: 17: 0)

**Turland** explained that while this proposal was for the Editorial Committee to delete an Example, it was also proposed to delete the second clause of Art. 6 Note 4. He informed the Section that it could either be sent to the Editorial Committee to do what the Editorial Committee considered best, or it could be rejected. He believed this wording was inserted by the Editorial Committee of the *Melbourne Code*. It pointed out that a nomenclatural novelty with a basionym could in rare cases be neither a new combination nor a name at new rank.

**McNeill** announced that he would vote against it.

**Art. 6, Prop. M** was **rejected**.

**Art. 6, Prop. N** (55: 1: 11: 0)

**Turland** suggested that if the Section were to accept this proposal, the Example offered would be sent to the Editorial Committee. The proposal sought a more precise definition of “replacement name” in Art. 6.11, not merely citing “avowed substitute” as an alternative term, which was currently in the *Code*, but clarifying that a replacement name was published as an avowed substitute for an older name. An avowed substitute meant explicitly proposing a name as a substitute for an earlier name. The implication was that this substitution was not accidental. This was implicit in earlier editions of the *Code* but when changes were made in the *Vienna Code* [2006], “avowed substitute” was simply cited as an alternative term for “replacement name” or “*nomen novum*”. This proposal made the definition more precise.

**Wiersema** added that Prop. N was related to some other later proposals. If a replacement name must be avowed, there were other situations where it was not avowed and those would be addressed in other proposals Greuter had put forward.

**Turland** explained this did not mean that a replacement name that was not avowed could not be a replacement name. Other proposals specified when it would be a replacement name and when it would not. Criteria were provided to allow the *Code* user to determine which was the case.

**Applequist** wondered if this proposal was connected to Art. 6 Prop. P. She was concerned that many names now considered to be replacement names, because a legitimate name was cited as a synonym, would suddenly be thrown up in the air. These names could be replacement names or names of new taxa and may have to be given status when they had previously been considered illegitimate. She suggested that the wording “as an avowed substitute for” perhaps narrowed the definition of what it took to be an illegitimate name because something else was cited in synonymy and maybe that no longer suffices.

**Greuter** acknowledged that the question was well taken, pointing out that the distinction between replacement names and names of new taxa had never been very clear in the *Code*. Often, under a strict interpretation of the *Code*, what had been published as names of new taxa had been considered replacement names. There was an interest in making this clearer for reasons of typification, which could only be done for names of new taxa, not for replacement names. Making this distinction clear would threaten to have negative effects with respect to traditional procedure and therefore necessitate the addition of the flexibility clauses [Art. 6 Prop. O and P]. This would give leeway to still consider as *nomina nova* names that were traditionally considered as *nomina nova.* Allowing this flexibility was, he thought, the only way to have both clarity and stability.

**Art. 6, Prop. N** was **accepted**.

**Art. 6, Prop. O** (56: 2: 7: 0)

**Turland** explained that Prop. O led on from Art. 6 Prop. N and covered the situation where a name was not avowedly proposed as a substitute for an earlier name. The name was not an avowed substitute but could be a replacement name if it was validated solely by reference to that earlier name. It could also be a replacement name under the provisions of Art. 7.5, which concerned names that were illegitimate because they were nomenclaturally superfluous when published. This proposal would allow a name that under current practice would be treated as a replacement name to still be treated as a replacement name, despite what was accepted in Prop. N.

**Art. 6, Prop. O** was **accepted**.

**Art. 6, Prop. P** (50: 12: 2: 0)

**Turland** reminded the Section that Greuter had spoken to this proposal already regarding situations where a name could be an avowed substitute under Prop. N and where, under Prop. O, names were considered replacement names when Art. 7.5 applied. There would still be situations where there was ambiguity about whether a name should be treated as the name of a new taxon or a replacement name. Prop. P was intended to provide a mechanism where users of the *Code* could make a decision and provide some flexibility in how that decision was made. It was based on preponderant usage and effected by means of an apposite type designation. The Example in the proposal would be sent to the Editorial Committee if the proposal was accepted. Turland noted that he and his colleagues had discussed this proposal in Berlin in April 2017, adding that his notes indicated he had a suggestion for a friendly amendment. The amendment was to change the beginning from “a name not avowedly proposed” to “a legitimate name not avowedly proposed”.

**Greuter** indicated that this would be an **unfriendly amendment** because it would restrict the application of the Article to cases of illegitimate replaced names, which he thought would be undesirable.

**Turland** agreed with Greuter’s explanation and **withdrew** the **amendment**.

**Applequist** pointed out that the *Code* provided a simple mechanism to save the current use of *Astragalus penduliflorus* – conservation with a conserved type. As it stood, if *Phaca alpina* was in synonymy we could know how that name was supposed to be applied. If preponderant usage could change the meaning of the name regardless of what was cited in the protologue, there would be endless disputes and many names would be thrown into doubt. She was concerned that the Committee for Vascular Plants, on which she served, would be getting hundreds of requests to determine the meaning of these names.

**Gandhi** spoke in favour of the proposal. He had encountered problems dealing with nomenclature for IPNI as well as the *Flora of North America* when the cited synonym and the intended new name had the same type. Unlike the Example cited in the proposal, where both names have different types, if the two names were to be identified with the same element, how should one proceed? He argued that the *Code* did not have an answer. In such cases he used to consult people and would go with the established usage, but if the established usage was evenly split, how could a user of the *Code* decide whether the proposed name was a new [replacement] name or that of a new taxon?

**McNeill** thought the questions that had been raised would be resolved by the final part of the proposal. Decisions would be effected by means of apposite type designation. The typification would establish whether it was a new taxon or a replacement name. Although there was a choice, once the choice was made it was then clear. He saw no reason not to support the proposal.

**Garland** asked for clarification of the term “preponderant usage”

**Turland** suggested in this context it meant “traditional and current,” especially current usage of a name in the literature.

**Sennikov** asked about the situation in which such names were treated as homotypic. He gave the example of *Phaca alpina* and *Astragalus penduliflorus* where the author simultaneously cited a common type for both names without any comment. If later an author removed *P. alpina* from the synonymy, reinterpreting the case and designating a separate type, what should be done in that situation? Would the first action be considered a simultaneous type designation for both names, and in the second case would the type designation be superfluous or not?

He thought there were many such cases when authors placed one name into the synonymy of the other, assuming they were homotypic. They were not strictly speaking homotypic at that time, so there was a certain ambiguity about type designation in such cases. Was the type designation effected? Had it been effected in this case or not? He asked the proposer if this situation would be resolved in such cases.

**Greuter** answered that he was afraid there was an uncertainty in such cases and suggested that the place to solve it was in Art. 7 and Art. 9. He pointed out that the question of what was an effective type designation was the crucial one as that was not always clear and it must be covered in Art. 9 in a general way for all type designations.

**Art. 6, Prop. P** was **accepted**.

**Art. 6, Prop. Q** (57: 3: 4: 0)

**Turland** summarized this as adding a new paragraph after Art. 6.11 explicitly allowing a factually incorrect statement about the status of a name to be treated as a correctable error. This proposal was to prevent such names from being considered not validly published and tied in with the proposals that had just been passed.

**Monro** and **Turland** pointed out that all of Art. 6.9 to Art. 6.11 would not fit on the screen at the same time.

**Knapp** helpfully suggested a slow upward scroll like the introduction to *Star Wars*.

**Turland** explained that the screen showed definitions of “name of a new taxon”, “new combination”, “name at new rank” and “replacement name”. He expounded, saying that if someone published what was in fact a replacement name, but called it a new combination, the statement that it was a new combination was not going to prevent valid publication.

**Wiersema** agreed and gave another example: if an author said it was a new combination and still provided a type and a description it could be treated as the name of a new taxon, if the presumed basionym turned out to be illegitimate.

**Lindon** wanted to draw attention to the use of the word “status” as potentially misleading. When she read it, she thought it referred to validly published vs. not validly published. She suggested either a friendly amendment or for the Editorial Committee to ensure the use of the word “status” was clear.

**Turland** assured her that the Editorial Committee would ensure that the meaning of “status” in this context was clear.

**Gandhi** observed that in early American literature he had seen names published as *species nova* which turned out to be either new combinations or new names. He went on to add that occasionally, even in journals such as *Taxon*, he had seen a name published as a *genus novum* which was only a replacement name because the cited synonym and the proposed new genus had the same type. He suggested that such errors still occur in modern times and occurred quite frequently in the past.

**Art. 6, Prop. Q** was **accepted**.

#### Article 7

**Art. 7, Prop. A** (49: 3: 14: 0) was sent to the **Editorial Committee**.

**Art. 7, Prop. B** (4: 59: 2: 1) was **rejected** based on the **mail vote**.

**Art. 7, Prop. C** (26: 9: 31: 0)

**Turland** told the Section that Art. 7 Prop. C was another proposal from Greuter to reword Art. 7.5. This rewording did not change the rules and he suggested it could be sent to the Editorial Committee. It sought a clearer wording of Art. 7.5, which was once quite simple in the *Code* but was complicated considerably by additions at the Vienna Congress in 2005.

He added that if the proposal was accepted the Rapporteurs thought that perhaps the clause “e.g. by inclusion (Art. 52.2) of the type of the name causing illegitimacy in a subordinate taxon that did not include the intended type of the illegitimate name” could be removed from Art. 7.5 and reformulated as a note.

**Greuter** agreed that the first passage suggested for deletion was not essential to the meaning of the Article. He did think that it was educational, and despite its lengthening an already long article, it made it easier to use. He was happy to leave that decision to the Editorial Committee.

**Turland** explained that if the Section voted to refer Prop. C to the Editorial Committee, the Editorial Committee would also have the latitude to adopt the Rapporteurs’ suggestion if it was considered an improvement, or the clause could be left in if its inclusion was felt to be more instructional.

**Art. 7, Prop. C** was sent to the **Editorial Committee**.

**Art. 7, Prop. D** (28: 19: 19: 0)

**Turland** explained that Prop. D was related to Art. 9 Prop. L and emphasized that Art. 7.7 applied only to names that were validly published solely by reference to a previously published description or diagnosis. He argued that this could be a useful addition to Art. 7.7 regardless of what was decided in the related Art. 9 Prop. L. On the other hand, the proposed additional words in Art. 7.7 might make it less clear.

**Gereau** commented that the rewording of the Article was not explicitly wrong but seemed completely unnecessary because the added text did not change the meaning and it seemed to add no clarity. He saw no advantage in this change and would oppose it.

**Wilson** supported the proposal but thought the Editorial Committee should look at the wording: having “not by the reproduction of, but” made it necessary to read the sentence twice. She suggested either “not by the reproduction of a previously and effectively published description, but solely by reference to it” or some other revised wording.

**McNeill** agreed that if Wilson thought that “not by the reproduction of” meant anything to do with “by reference to”, then it was clear that the wording was extremely muddled. He agreed with Gereau’s suggestion not to accept the proposal.

**Greuter** countered that it was an important addition and not merely editorial because it reflected the policy that was used by the Linnaean Name Typification Project when a former validating statement by Linnaeus was reproduced textually in *Species Plantarum*, the place of valid publication. Material added later and seen later by Linnaeus could be eligible as type material. Otherwise the *Code* could be read to mean that, the reproduction being the same as a reference, only material from, for example, *Hortus Cliffortianus* would be available for type material if the descriptive statement or diagnosis or *nomen specificum legitimum* came from *Hortus Cliffortianus*.

**McNeill** wished to clarify his remarks: he agreed with Greuter but thought the word “solely” already covered that situation, quoting “solely by reference to a previously and effectively [published] description”. He explained that those Linnaean names were not published solely by reference to a previous description because the previous description was reproduced in *Species Plantarum*. For this reason, he agreed with Gereau that the proposal was unnecessary, because “solely” already covered this case. He suggested the alternative of adding a Note, to the effect that the reproduction of a previously published description was not covered by this provision, to explain what “solely” meant in this context. He went on to explain that he made his comments understanding Wilson to have implied that the proposed new wording might mean “not by reference to”, whereas the proposer meant what Greuter had referred to, the inclusion of the wording of a previously published description.

**Sennikov** suggested that such cases had been much debated in the past, and therefore this was an important matter. He suggested that the decision of adding words to the Article, rewording it or adding a Note should be left up to the Editorial Committee.

**Art. 7, Prop. D** was **accepted**.

**Funk** suggested that, as there was discussion on the best way to implement the change, the people who were in favour of the proposal should get together to rewrite it, to propose something specific and present it the next day rather than continuing with this vagueness.

**McNeill** felt the situation was not vague and that the Section had just decided by a clear majority to accept the proposal. He thought perhaps the simplest solution now was for a proposal of an amendment to give authority to the Editorial Committee to include the new material as a Note if it was deemed suitable.

**Turland** summarized that the Section clearly accepted a change to the effect of Art. 7 Prop. D and that there were previous suggestions outstanding to discuss. One was the original proposal criticized by Wilson as having unclear syntax that needed editing. There was another suggestion that instead of amending the Article there would be a Note explaining the same things.

**Schori** requested the Section break for lunch and continue the discussion afterwards.

[*The Section broke for lunch*.]

### Monday, 17^th^ July 2017, Afternoon Session

**Knapp** welcomed everyone back from lunch and outlined some changes to the lunch schedule for the remainder of the week. She went on to announce to the Nominating Committee that its Secretary, Vicki Funk, would like to have a short meeting after the end of the session to plan the next steps.

#### Article 7 (continued)

**Turland** then explained the outcome of what had been discussed before lunch regarding Art. 7 Prop. D, noting that there was a move to have a second vote on the proposal. Several people had pointed out, in agreement with the Rapporteurs, that there was nothing more to do because the Section had accepted the proposal. In any event, the Editorial Committee would have the latitude to express the change with the current wording or different wording, or even as a Note. In this case, nothing new had been introduced to the *Code*, it was simply making the Article clearer and adding some explanation of what was already in the rules.

**Art. 7, Prop. E** (31: 4: 30: 0) was **automatically** sent to the **Editorial Committee**.

**Art. 7, Prop. F** (4: 13: *47: 1)

**Turland** said that this proposal placed a new rule after Art. 7.10 on how reference may be made to a name being lectotypified or neotypified. The *Melbourne Code* did not explicitly rule that when a name was so typified it must be referred to, although he could not imagine how it could be typified otherwise. The proposed methods of referring to the typified name appeared to be precise because they all depended on the same type. He gave the example of an author citing a homotypic name but not its basionym; it would be obvious which name was being typified in this case. The Rapporteurs were concerned about the phrase “an invalidly published designation that was supposed to be that name”, because this depended on supposition rather than typification, and it could therefore be ambiguous.

There was a high Editorial Committee vote in the mail ballot, which the Rapporteurs had said would mean voters supported the proposal but wanted it to be amended. This indicated that the Rapporteurs’ concerns were shared by those voting.

**McNeill** proposed an amendment, that the phrase “or an invalidly published designation that was supposed to be that name” be deleted from the proposal.

[The **amendment** was considered **unfriendly** but was supported by **five seconders**.]

**Knapp** explained that the Section would now vote on whether or not to accept McNeill’s amendment to the proposal.

[The **amendment** was **accepted**.]

**Gereau** said this proposal might be acceptable with an early cut-off date to allow for some existing typifications to be maintained. He added that going forward it did nothing but enshrine bad practice and de-clarify the requirements for lectotypification and neotypification, and as such was entirely undesirable.

**Turland** commented that the proposal as amended would not change current practice. Under the current *Code*, for a typification where the basionym was not mentioned but a new combination based on it was, if the author wrote “lectotype designated here”, it was still obvious what name was being typified. He thought this would function like a Note, making explicit what was implicit elsewhere in the *Code*. He concurred with Gereau that the *Code* should not be encouraging poor practice.

Turland felt that while the Rapporteurs strove to be impartial, in this case he thought the change would not have positive consequences, nor would it change the functioning of the *Code*.

**Art. 7, Prop. F** was **rejected**.

**Art. 7, Prop. G** (3: 13: 49: 0)

**Turland** explained that Prop. G concerned Examples which would have been sent to the Editorial Committee only if Prop. F was accepted.

**Art. 7, Prop. G** was **automatically rejected** [Listed in error as “ed.c.auto.” by Turland & al. in Taxon 66: 1239. 2017].

**Art. 7, Prop. H** (53: 4: 4: 4), **Prop. I** (53: 5: 3: 4) and **Prop. J** (50: 7: 4: 4)

**Turland** explained that these three proposals only affected fungal type designations. The proposals, strongly supported in the mail vote and by the Nomenclature Committee for Fungi, required registering type designations of names of fungi.

**May** spoke in support of the proposals, explaining that it was difficult to trace later typifications, i.e. lectotypes, epitypes and so on. The mycological community already had registration for new names and new combinations relating to fungi with approved repositories for the identifiers, and people were already registering later typifications with those authorities.

**Hawksworth** pointed out that a lot of these proposals, although they bore his name, originated from an International Mycological Congress (IMC). The wording was then worked on by the International Commission on the Taxonomy of Fungi, so they came from a broad group. He added that registering types had already been introduced for some years in the leading mycological journals and did not seem to cause any problems. He hoped all the proposals would be approved almost automatically.

**Wiersema** mentioned that the mycologists at his institution, the USDA Agricultural Research Service, supported all three of the proposals.

**Art. 7, Prop. H, Prop. I** and **Prop. J** were **accepted**.

#### Recommendation 7A

**Rec. 7A, Prop. A** (44: 26: 7: 0)

**Turland** summarized the proposal, saying Prop. A would recommend where type specimens should be deposited. Although it could be argued that scientists should have the option to choose, it was not unreasonable to give advice in the *Code* and make it a Recommendation. If the proposal were passed it could be editorially incorporated into the existing Rec. 7A.1 rather than as an additional Recommendation.

**Dorr** thought it was dangerous for a Code that was ostensibly self-containing to reference things over which it had no control. He cited Brummitt [see Flann & al. in PhytoKeys 45: 108. 2015 https://doi.org/10.3897/phytokeys.45.9138] as introducing the concept of referring to ISSNs [International Standard Serial Numbers], which were not under the control of the *Code*. He warned people to be careful about linking the *Code* to *Index Herbariorum*, which the *Code* also did not control, or to any other organization or publication over which the *Code* had no control.

**Groom** wondered if institutions not yet registered in *Index Herbariorum* could be given time to register before this rule came into place.

**Knapp** pointed out that this would be a Recommendation, not a rule, and would be merely stating what was good practice.

**Turland** added that implicit in the Recommendation was that if an institution or herbarium collection was not listed in *Index Herbariorum* or the *World directory of collections of cultures of microorganisms*, the *Code* was recommending that type specimens should not be deposited in those collections.

**Herendeen** spoke up for palaeobotanical collections, saying that there were many such collections and that most palaeobotanical collections were not listed in *Index Herbariorum*. However, he did support the Recommendation because in palaeobotany authors deposited their type specimens in private collections and this caused a problem. He asked to modify the wording to allow for other official collections. He gave the example of the palaeobotanical collection at the Field Museum (not listed in *Index Herbariorum*).

**Turland** suggested that everyone was trying to say the same thing. The *Code* already recommended that type specimens be deposited in public herbaria, with the intent of recommending that authors did not put specimens in obscure or private herbaria. The proposal under discussion made that more explicit by referencing two specific indices, even though the *Code* generally did not recommend outside sources.

**Hawksworth** gave more information on the *World directory of collections of cultures of microorganisms* because he believed many in the audience might not know about it. He added that, for many culture collections, it was not possible to preserve cultures in a metabolically inactive state, so he thought it would be dangerous to mention that collection because people thought that if they sent a culture there it would be dried and preserved. For that reason, he was opposed to the change and felt the current Recommendation was fine.

**Miller** thought all the concerns expressed could be handled by leaving Rec. 7A as it was written, as a principle, rather than a prescription to use specific depositories.

**Gandhi** noted that while indexing names for IPNI he came across citations of herbaria housing holotypes in the author’s personal herbarium. He found out later on that such a herbarium did not exist. For all practical purposes, the author had met the requirement to validly publish a name, but in reality the herbarium was fictitious. Therefore, he thought the proposal would be useful if it was practised.

**Turland** pointed out that the *Code* already recommended a public herbarium.

**Greuter** announced that he had two misgivings. The first, which had already been voiced, was the reference to concrete registers or indexes of collections that were not all comprehensive for all domains of organisms covered by the *Code*. He suggested this could be circumvented if the current statement was replaced by something like “a relevant international register of collections”, or “biological collections”.

His second concern was the discrimination against private herbaria. Introducing this principle into the *Code* was a declaration that most of the prominent botanists of the early Linnaean period were acting against it, beginning with Linnaeus himself. He did not think it wise in terms of biological politics to discourage the deposition of types in well-kept and sustainably-kept private collections. He said that often they were better cared for than some underfunded and understaffed public collections.

**Nakada** thought this was a similar case to Note 1 in Art. 46 and proposed that this should not be a Recommendation, but rather a Note or Example.

**Rec. 7A, Prop. A** was **rejected**.

#### Article 8 and Recommendation 8C (new)

**Art. 8, Prop. A** (60: 4: 2: 0)

**Wiersema** explained that this proposal sought to provide a clear definition of a gathering, which was a term only mentioned in passing indirectly in a few provisions of the *Code* and was nowhere precisely defined. The proposal required moving around some of the material in the footnote of Art. 8.3, because it became redundant if the definition was made clear. The definition, as stated in the proposal, was that a gathering involved four elements: the same collector, the same place, the same time (i.e. date) and presumably the same taxon. There could be cases where it later turned out that it was a mixture; the *Code* would have to account for that possibility.

**Kirk** wondered if “same time” and “single locality” could be defined more accurately. What did “same” time mean: day, month, year? Same locality: woodland, adjacent woodlands, same country?

**Wiersema** responded that “same time” would be considered as the day because it could not be exactly the same time.

**Kirk** said that he had asked that question of Greuter, who suggested that people studying marine organisms, who throw a trawl net over a ship and drag it for two weeks, could consider that one collecting event covered 14 days of collecting. Kirk felt that the type should be what the person says it is, without any complication of “did I pick it here or here?”, or “was I in the Arctic Circle on 31 December and it was collected one minute before midnight or one minute after midnight?”, suggesting it could be different years for the same gathering, or at the end of the century, which would be a different century. If it was done in 1999, it would be a different millennium. He urged the Section to keep it simple: holotype and nothing else.

**Wiersema** argued that in Art. 40.2 a name was validly published if the type consisted of a single gathering. Since this wording was in the *Code* it was necessary to define a single gathering.

**Paton** wondered if cases of DNA sampling and population sampling should be considered, where a collector could deliberately collect more than one thing, thinking they may be different things, and only one of them later became a type. A collector might do that in the field by using different numbers, but the current wording didn’t have numbers, it just talked about someone collecting species from the same place at the same time. He asked for some wording that would imply that unless the collections were somehow identified as deliberately different, they should be considered the same. He suggested that with the increase in DNA work, population samples might add to the confusion.

**Wiersema** informed the Section that there were a couple of proposals that dealt with the issue of whether the same number needed to be assigned to something to consider it the same taxon or the same type. That was a separate issue, and there were alternative ways that had been proposed for looking at that.

**Redhead** said another complication was for types for fungi where the gathering was made in the laboratory over several days or weeks. Researchers would culture a fungus and grow various stages of it before finally deciding, looking collectively at the whole picture, that it was a new species. They would then put all these things on to a sheet or a box and designate it as type. He felt the mycological community had allowed latitude, and he was concerned that the precision of the wording could end up excluding some of these instances.

**Greuter** had misgivings concerning this proposal. Although he recognized and realized that it was desirable to have concrete definitions of what a gathering was, he hesitated to vote for it if it was a retroactive provision. His reasons were not only a question of unity of time, but also the identity of collectors because of contemporary and historical instances of the same gathering being labelled as being from different collectors. He argued that if these were in a description of a new taxon and cited as isotypes, the name might later be regarded as not validly published if they were no longer considered to be the same gathering.

Greuter thought some cases might be trivial, for example if one label had the collectors listed alphabetically and the other by seniority. In other cases, a “less important” member of a group, for example a student, might be left out on some of the labels and not on the others. For these reasons the identity of collectors could not always be taken for granted in what was considered a single gathering. He said he knew of one case where two botanists collecting together quarrelled about the distribution of the harvest. Each issued his own labels with only his name on the label, but these were part of single gathering as they were done by the same two persons at the same place. He finished by saying that the current *Code* refers to unity of date, but not to unity of collectors, so this proposal would change what is now in the *Code*.

**Thiele** wished to address Kirk’s point, saying he supported this proposal because a good definition of gathering was important. Leaving it undefined was negative, though it was important to leave some of those terms with some latitude for interpretation. Specifying that it must be the same day or within a 10-kilometre radius, or a one-kilometre radius, quickly became *reductio ad absurdum*.

Speaking to Greuter’s point, he said that he had had to deal with possibly the same collectors he spoke about. For dealing with types, this definition would have made that substantially simpler.

**McNeill** also supported the proposal, saying it was essential to have clarification of what a gathering was. For example, if a specimen was cited as an isotype that was collected apparently by someone else, it did not mean the name was not validly published; this was just a technical error. He thought it was more important to have clarity as to what a gathering was and, regarding the technicality of times and place, if a collection stated that it was collected on more than one day then it was not a single gathering. Likewise, if one specimen was collected by one person, and one by another person, it was not a single gathering. If the label data were contradictory (as opposed to one label being more detailed than another), they were different gatherings. If specimens bearing statements contradictory to this definition were considered not to be parts of the same gathering, then the application would work very well. In most cases it would be stabilizing rather than disruptive.

**Paton** sought clarification, asking if a collector collected something on a particular day, of the same species, and labelled them 1A, 1B, 1C, 1D, would these all be considered as one locality or not? He felt the current wording did not mention numbering or references given in the sheets.

**Wiersema** agreed with Paton but pointed out that there were some proposals that would deal with either side of that issue and those could be considered separately. Footnote 2 under Art. 8.3 said “the same collector at the same time”; the only thing missing was place, although if it was made at the same time it would have to be the same place.

**Knapp** suggested time travel.

**Turland** offered quantum mechanics.

**Wiersema** clarified that the proposal was making it direct, whereas how the term was defined was currently indirect.

**Redhead** wished to make an amendment to exclude microorganisms, fungi that might be gathered in a living state at one event, but the types would be generated over a period of time. He added that the mycologists were trying to add an exception to cover laboratory-generated types.

**Turland** thought if the type was from a culture in a laboratory, then the original collection site wouldn’t be the collection site for the type; it would be the laboratory.

**Redhead** explained that sometimes the specimen might have been isolated from the bark from a tree in a certain city. The author may or may not have the original. Thereafter the types would be generated on Petri plates, which would be dried or lyophilized cultures. Although it would be known where the type started, the actual type specimen would have been generated in the laboratory. This would apply particularly to asexual fungi: if a researcher crossed them and then generated a sexual state, the type would be for the sexual state which would consist of two isolates from two different locations originally, but the only place they came together was in Laboratory A on a certain date.

**Wiersema** said there were similar situations when someone would collect seed that was brought into cultivation and collections were taken from the cultivated plant at different times. Some would want this to be considered the same gathering, but the *Code* would currently rule that those were different gatherings because of the difference in time.

**Gandhi** supported this proposal, adding that he had come across situations where dioecious plants were collected in a single gathering and the male and the female plant together constituted a holotype. Quite often botanists got confused when both male and female plants were cited together as holotype; they thought it was a mistake. He believed the proposal would clarify that situation.

**Turland** announced that he and the Vice-rapporteur had decided that from now onward in the deliberations they would abstain [as did the President] from voting by a show of hands because they did not want in any way to influence the way the Section voted. He said they would continue to participate in the card votes.

**Art. 8, Prop. A** was **accepted**.

**Art. 8, Prop. B** (4: 58: 4: 0) was **rejected** based on the **mail vote**.

**Art. 8, Prop. C** (30: 23: 11: 1)

**Turland** explained that Prop. C dealt with the issue of collection numbers and the significance of collection numbers. It would add a new Note with an Example, “field numbers, collection numbers, accession numbers or barcode numbers alone do not necessarily denote different gatherings.” He noted that there was another proposal which had the opposite effect and placed importance on collection numbers.

This Note was a statement of fact rather than an explanation of what was already in the *Code*, and the Example drew a different conclusion to Art. 46 Ex. 21, where *Pancheria humboldtiana* was regarded as not validly published because “no type was indicated”. He suggested that the Note and the Example could be sent to the Editorial Committee because it could simply be interpreted as a statement of fact. On the other hand, if the Section felt that there should be something explicit in the *Code* regarding the importance of collection numbers then it should accept the proposal.

**Gereau** explained that in current and all foreseeable practice, the fundamental identifier for a collection was the field or collection number. He argued that if different collection numbers no longer denoted different gatherings, this was entirely contradictory to good practice and should not even be considered.

**Sennikov** explained that the idea behind this proposal was the practice in some countries or by certain curators to give collection numbers not to gatherings but to individual plants. This meant every plant within a single gathering received its own field number. The field numbers may be discarded when collections were subsequently placed in the herbarium or they may be renumbered later. The number that appeared on the labels may not be given by collectors but rather by curators. This may have happened at different times, and parts of the same gathering in different herbaria may have different numbers given by different people for different purposes. If such numbers were taken at face value and accepted as identifiers pertaining to gatherings, this would be highly misleading.

**Marhold** stressed that there were different practices in different countries. Population sampling had already been mentioned, and when used for morphometrics or molecular studies, each plant must be numbered, and this number should be kept. When it was incorporated into a herbarium it could not be considered a different gathering. For this reason, he supported the proposal.

**Schori** discussed a concern she had about the proposal citing a hypothetical example of a specimen labelled “*Clemens & Clemens*, Mt. Kitanglad, March–April 1933”. In such cases there may be many different numbers with many specimens of the same taxon. If the collection numbers did not mean anything, they could be considered a single gathering. This could potentially lead to considering taxa from different parts of the mountain, collected a month or more apart, as a single gathering, which she did not think should be allowed.

**Barrie** pointed out that extended dates with numerous collections was accounted for by the wording “does not necessarily denote that”. This wording meant one had to look at things in the context of the collections to make a judgement as to whether it was a single gathering or not. There was flexibility in the rules as they existed, and he thought it would be a useful addition.

**Dhabe** suggested that if the definition were applied, any material collected from the same locality and at the same time by the same collector, even though it had received different numbers, could be considered a gathering. If there were different specimens, one flowering and another fruiting, or a vegetative and a reproductive part, they would constitute the gathering. If an author wanted to give additional support and wanted to use another specimen, they may describe it as an epitype, but two specimens should not be described as a holotype.

**Seregin** proposed a friendly amendment, citing the fact that not all herbaria used barcode numbers. He suggested changing “barcode” to “Herbarium ID” or “identification numbers”.

[The **amendment** was accepted as **friendly**.]

**Paton** suggested that, since it was not the herbarium that was being identified but rather the specimen, it should be “specimen identifiers”.

**Knapp** suggested they were straying into wordsmithing the *Code* by a group of 80 people, but that she would accept it from Paton only because he was very nice. [*Laughter*]

[Paton’s **amendment** was accepted as **friendly**.]

**Sennikov** explained that the point was that now in collections there were two numbers that were relevant to physical herbarium sheets. In the past, accession numbers appeared when sheets were stamped with designations of particular herbaria and they bore the numbers on those sheets. Current practice was to add barcodes or other items that allowed machine reading of those numbers. Some herbaria did not use the original accession numbers on those barcodes or whatever was in place of those barcodes. Keeping the numbers was essential; it did not matter what they were called, just that they should be correct in some way.

**Turland** suggested instead of talking about which was the best term to use, maybe a more general term like numbers could be included, such as: field numbers, collection numbers, accession numbers, or barcode numbers, but not an exhaustive list. This conveyed the idea that these kinds of numbers alone did not necessarily denote different gatherings.

**Barrie** reminded the Section that this was a Note, so all this wordsmithing was unnecessary as a Note gave the Editorial Committee much more license to change things. He called the question.

**Knapp** explained, since it had not yet occurred in these proceedings, that “calling the question” forced the Section to vote as to whether they wanted to vote or to continue discussion.

[The Section voted **to vote**.]

**Art. 8, Prop. C** was **accepted as amended**.

**Art. 8, Prop. D** (9: 16: 39: 1) was **automatically** sent to the **Editorial Committee**.

**Art. 8, Prop. E** was discussed under **Art. 40, Prop. A**.

**Art. 8, Prop. F** (8: 52: 6: 0) was **rejected** based on the **mail vote**.

**Art. 8, Prop. G** (10: 4: 54: 0) and **Prop. H** (12: 3: 51: 0) were **automatically** sent to the **Editorial Committee**.

**Art. 8, Prop. I** (51: 9: 5: 1)

**Turland** opened the discussion of Prop. I by saying that the purpose was to avoid unduly restricted typifications, for example when one sheet of a multi-sheet specimen was designated as the type. If the specimen consisted of several sheets, but only one sheet was designated as the type, the others were excluded in the mistaken belief that the sheets were separate specimens, that they were in fact duplicates.

The proposers had cited the herbarium at Geneva where specimen folders contained a single specimen consisting of multiple sheets, which was permitted by Art. 8.3. The problem arose when the sheets were not clearly labelled as being part of a single specimen as stipulated in Art. 8.3, that “all sheets must bear a label stating that they belong to a single specimen”. The proposed amendment was that a single label may apply to all the sheets.

**Alford** offered a grammatical suggestion for a comma inserted between “single” and “original” because it may be unclear if there were copies of a label on different sheets.

**Saarela** asked if it was necessary to define single and original? If there were two labels and one was slightly edited for some reason before it was mounted on a second sheet, would it be a duplicate of the original label because it was not identical?

**Barrie** thought if separate specimens had separate labels, there must be some indication from a curatorial standpoint that those were parts of the same collection or part of the same specimen. If they were marked 1(a) and 1(b) or “one of two” etc. it was clear that they were considered a single specimen. The situation in this proposal was in the spirit of the current wording of the *Code*, but not currently covered, where a folder had one label on the top and the specimens inside were not labelled. That label was considered to apply to all the sheets inside that folder.

**Art. 8, Prop. I** was **accepted**.

**Art. 8, Prop. J** (11: 53: 11: 1), **Prop. K** (12: 56: 7: 1) and **Rec. 9A, Prop. A** (15: 39: 11: 0)

**Turland** noted that Prop. J was the converse of what had just been accepted regarding the significance of collection numbers and that these three proposals could be considered together. Prop. K would rule that duplicates of a gathering must bear the same collection number. The Section had already agreed that this should not be the case. Turland emphasized that if the Section accepted this proposal, it would mean accepting the opposite of what had just been accepted in Prop. I. Turland went on to explain that Rec. 9A Prop. A would add a new paragraph to extend Rec. 9A.2 with 9A.2*bis*, “The possibility of a mixed gathering must always be considered by an author choosing a lectotype, and corresponding caution used”, which cautioned authors about lectotypification and designating specimens as duplicates.

**Art. 8, Prop. J, Prop. K** and **Rec. 9A, Prop. A** were **rejected**.

**Art. 8, Prop. L** (4: 54: 7: 0) was **rejected** based on the **mail vote**.

**Art. 8, Prop. M** (5: 4: 58: 0) was **automatically** sent to the **Editorial Committee**.

**Art. 8, Prop. N** (32: 20: 13: 1)

**Turland** introduced Prop. N, which would replace footnote 1 under Art. 8.1 with a new paragraph and add two Examples. If the proposal was accepted the Examples would go to the Editorial Committee. The proposal would modify the definition of illustration in Art. 8.1 footnote 1, which was new in the *Melbourne Code*, and would promote it to a rule. He suggested that the revised definition would further explain what an illustration may consist of. For example, an illustration that showed flowers and fruits that were only visible at different times could not have been executed at one time. However, as this was an illustration and not a specimen there was nothing in the *Code* that said an illustration had to be drawn on a single day.

**McNeill** offered his support for the proposal, saying he found, when reviewing lectotypifications, he was not sure if something was an illustration when it comprised several figures within one plate. This proposal clarified that so long as the different figures were illustrating the same material it was just one illustration.

**Nakada** argued that this proposal was of concern for those working with microorganisms because many were based on types consisting of several combined figures or photographs. He suggested amending the proposal to “a photograph or photographs”.

**Turland** felt this would be splitting hairs and that it was not necessary to specify a photograph or photographs because the second sentence said, “a single figure or a group of figures, a work of art, or a photograph”.

**Seregin** offered an amendment to use either the word “illustration” or “figure” throughout.

**Sennikov** observed that the first sentence would then say something was an illustration and then say an illustration may consist of a single illustration. He explained that this was why the word “figure” appeared, which he defined as something less inclusive than an illustration: it may be part of illustration or may coincide with it.

[The **amendment** was considered **unfriendly**.]

**Thiele** supported the intent of the proposal but had concerns about the amount of wordsmithing needed for illustrations and figures. Another concern was that the insertion of the term “gathering” at one Nomenclature Section had led to a subsequent Section having to specify what gathering meant. He confessed that now he did not know the meaning of the term “source” in this proposal and was concerned that the Section would have to define that later. He suggested that the proposal should be reworked and reintroduced on a subsequent day.

**Sennikov** did not think “source” needed to be defined. The *Code* could have said “plant” but then the text would have been restricted to macrophytes. The word “source” meant a plant or any other individual organism, including the case when the illustration was derived from that organism, probably in a different season when the artist came and took a picture of first flowers and then fruits.

**Knapp** ruled from the President’s chair that there was too much wordsmithing in this proposal and suggested that the proposer should prepare a new proposal to be introduced from the floor on Friday.

[**Art. 8, Prop. N** was **withdrawn** and reformulated into **Floor Prop. 1–3**.]

[*The Section broke for afternoon tea*.]

**Knapp** made a public service announcement regarding the comment slips. She stated that, together with the audio and video recordings, the comment slips would be used to help write up the proceedings of the Nomenclature Section. Several slips had been handed in without names or times and she reminded the Section to add this information. Knapp went on to emphasize that sometimes the recordings were difficult to manage, and the comment slips were vital for the transcribers to work out who was speaking and what they were saying.

**Turland** noted that the secretary of the Nominating Committee, Funk, had asked him to announce that the secretaries of the Permanent Nomenclature Committees, or their deputies if the secretaries were not present, should send their lists of proposed members to her by Tuesday morning. He added that anybody in the Section who wanted to serve on a Permanent Nomenclature Committee should talk to Funk. Anyone who had already spoken to her about this should confirm that their name was on her list.

**Art. 8, Prop. O** (6: 33: 2: 25), **Rec. 8C (new), Prop. A** (6: 36: 2: 22) and **Art. 9, Prop. A** (15: 38: 7: 18)

**Turland** noted that these proposals concerned DNA sequence data being acceptable as types and how this could be achieved. Art. 9 Prop. A concerned a cross-reference, which would be editorial. The Nomenclature Committee for Fungi did not support these proposals, but eight members of that Committee voted for a Special[-purpose] Committee to look at the matter instead.

**Applequist** acknowledged that environmental DNA studies had shown that there was a lot of unsuspected biodiversity out there. She argued, however, that to use those sequences to give uninformative names to a number of taxa that no one had ever seen was antithetical to traditional taxonomy. She suggested it might lead to a person who found a new mushroom that had never been described before being afraid to publish it as new, lest it had already been named from a random DNA sequence.

**Hawksworth**, the proposer, pointed out that this was something that was already being done. Mycologists were finding groups of fungi, sometimes even separate phyla and classes, which did not have any known representatives. People had eventually been able to isolate one representative of such a group and visualize it in some way using special techniques. Only about 3% of fungi on the planet were known, and this was a major constraint to people working in the field. He noted that people had already proposed and cited DNA sequences as types, trying to get around and apply the current rules, and it was down to the taxonomist to decide how to represent their taxon. He added that if there was going to be a Special-purpose Committee, it would need to work quickly and contain people who were specialists in DNA data. He cited a paper in preparation as a possible guide for a code of practice that could be adopted by the International Commission on the Taxonomy of Fungi. He thought that by the next Congress, there could be hundreds, if not thousands, of these already sequenced and named, regardless of the *Code*.

Hawksworth said when English was proposed as an alternative language to Latin for diagnoses, people were terrified that there might be lots of new taxa proposed, but he did not think this would be the case. He noted there may be a few people that try to do strange things, but there would be guides of good practice available to editors of journals and referees.

**Greuter** was concerned about the amount of time being spent in discussion and the number of proposals concerning Art. 9, which he agreed was unsatisfactory in many details. However, as it was so important, he thought it would not be wise to act on these proposals without advice from a body who had the time and skills to come up with considered opinions. He asked the Rapporteur-général if there was a proposal to set up a Special-purpose Committee to study questions of types and typification and, if so, he would second it. If not, he would propose it himself, to avoid spending too much time in discussion that might lead to rash decisions.

**Turland** was not aware of the Rapporteurs having made a specific proposal. The Special-purpose Committee he referred to earlier was suggested by the Nomenclature Committee for Fungi and the high number of Special[-purpose] Committee votes in the mail vote. The Rapporteurs themselves were not suggesting that there should be a Special-purpose Committee; that would be for the Section to decide.

**Greuter** explained he was referring to a Special-purpose Committee to examine problems of types and typification, ready to receive all proposals that the Section would refer to it. He suggested that in view of that high number of proposals concerning types, there would be no problem in finding suitable membership for such a Committee.

**Turland** summarized Greuter’s proposal, to establish a Special-purpose Committee with a mandate to report back to this Section, or a later Section, to consider problems of typification. This would include everything in Art. 8 except for the last two proposals and all proposals for Art. 9, as Art. 7 had been dealt with. He explained that for Art. 9 there were around 50 proposals, some already rejected in the mail vote and some concerning only Examples. There were plenty left which the Section needed to talk about. Some of them were quite complicated, and there would then be the option to refer them to a Special-purpose Committee, if the Section voted to establish such a Committee.

**Schori** noted that the mycologists at the USDA strongly opposed Prop. O, adding that if only 3% of fungi had been sequenced, how could we know that a DNA sequence was not shared by maybe hundreds of different taxa until everything had been sequenced? How could that serve as the type of an organism? From her experience of DNA barcoding, until you looked at a particular gene region for a particular group, you had no idea whether it would be informative or not. Unless the whole genome was sequenced, you would have no way of knowing whether that sequence was unique to that organism. She did not support this series of proposals.

**Groom** was surprised that it mentioned “no physical specimen being found”, because a physical specimen would have been sequenced, for example in a dried soil specimen, even though you might not know where the organism was in a mixture. You should be able to resequence that physical specimen and find it again, and with new techniques in the future, it should be possible to further extend the sequence. To specify that organism both the physical specimen and the DNA sequence were required.

**Dhabe** spoke against the proposal, saying for the purposes of identification or comparison a type specimen should be a specimen, or at least an illustration. It should not be a sequence of nitrogen bases because this could not serve the purpose of a type specimen. He cited the problems of polymorphism and infraspecific variation.

**Wilson** admitted to not being a mycologist, but was still feeling wary about the proposal because of the special problems with fungi. At the same time, she noted there were moves even within the higher plants to start recognizing species, and presumably even typifying them, on DNA sequences. However, unless there was a full genome for an organism, we could not know whether it was unique, or whether there were another 300 taxa that had the same small sequence. She felt it was analogous to electronic publication: something that was coming and that the nomenclatural community would have to work towards accepting eventually. She agreed that she would like to see a Special-purpose Committee formed to look into it as a general matter.

**May** also agreed that reliance on DNA for taxonomy was coming, that mycologists were engaging with this, and that many branches of the fungal tree of life were known only from sequences. He gave an example of good practice, urging the delegates to look up the genus *Hawksworthiomyces*, in which a new species was described on the basis of sequence data, as an example of the way that it could be done.

He agreed with the need for a Special-purpose Committee to look at the concerns and the suggestions that had been made and come up with specific proposals. He noted that in the International Commission on the Taxonomy of Fungi there was a working group on this issue, but it was split 50:50 in terms of formal proposals to the *Code.* He finished by stating that the *Code* could not specify the regulation of taxonomy, but it could specify that new species of fungi described on the basis of DNA sequences were published in one particular journal.

**Söderström** suggested an amendment to include all groups covered by the *Code* to take advantage of DNA as a tool in taxonomy and consider DNA sequences as good indicators of new species. He did not see any difference between using an illustration as a type or using a phylogenetic tree or published sequence alignments as a type.

[The **amendment** was accepted as **friendly**.]

**Struwe** reiterated that this concerned one DNA fragment. She gave the example from her own work of sequencing green algae to show ancient horizontal gene transfer from fungi into the green algae. Her concern was that if one of those tiny little pieces of DNA were found, it would appear to be from a fungus, but might actually be a green alga. She urged the Section to think more fully about the implications of these changes.

**Gereau** pointed out that taxonomy had always been about the classification of organisms. Molecular sequences were not organisms but rather the coding mechanisms by which organisms are made. He explained that taxonomists had always classified the phenotypes that resulted from those genotypes. He argued against the need for a Special-purpose Committee and felt the Section should not be considering this proposal seriously.

**Thiele** moved that a Special-purpose Committee be set up to consider the question of DNA sequence data and typification and that Art. 8 Prop. O be referred to that Special-purpose Committee.

[The motion was **seconded** and a new **Special-purpose Committee** was established to deal with DNA sequence data and types.]

**Schori** questioned the friendly amendment to the proposal, saying it sounded as though it extended to all organisms, without the stipulation that no physical specimen had been found. She sought confirmation that the intention of the friendly amendment was to restrict it to cases when no physical specimen had been found.

**Knapp** explained that when this proposal and the following two linked proposals were voted on, further proposals on typification that were rejected would be sent to the new Special-purpose Committee where those details could be discussed and ironed out.

**Art. 8, Prop. O** as **amended, Rec. 8C (new), Prop. A** and **Art. 9, Prop. A** were sent to the **Special-purpose Committee for DNA Sequences as Types**.

**Hawksworth** pointed out that a Committee report could not be implemented until 2025, which would be unacceptable to mycologists. He noted they would be producing their own code of practice. He thought that not adopting the proposal would create a huge problem.

**Turland** suggested this was the moment to propose the Special-purpose Committee Greuter had mentioned earlier to examine issues of typification. He explained that such a Committee could deal with problematic proposals in Art. 9.

[The proposal to establish a **Special-purpose Committee on Typification** was supported by **five seconders**.]

**Paton** wanted to clarify that this Special-purpose Committee was separate from the one which would deal with DNA as types.

**Knapp** confirmed that this was indeed a separate Committee.

**Greuter** explained that a Special-purpose Committee was an instrument that permitted the Section to reject proposals that were potentially problematic or for which the consequences had not been completely thought through, but which were supported in principle. His proposal for a Committee allowed proposals defeated on perfectly good grounds to be taken up and re-presented in a more coherent form at the next Congress.

**Turland** further explained that if the Section came to such a proposal, someone would need to move that it be sent to the Special-purpose Committee, should delegates decide to establish that Committee.

**Thiele** raised a point of order: it could arise that a Special-purpose Committee to consider issues of typification was established now, but that no proposals may be referred to it. As an alternative the Section could proceed with the proposals, and only establish the Committee if someone referred a proposal to it.

**Knapp** informed him that since there had been a proposal to establish a Special-purpose Committee, the Section would have a vote on the proposal now. If there was nothing for the Special-purpose Committee to do, they could just go to the Bahamas or something.

**Turland** added that somebody could propose to dissolve the Committee. [*Laughter*]

[The proposal to establish a **Special-purpose Committee on Typification** was **accepted.**]

#### Article 9

**Art. 9, Prop. B** (61: 10: 7: 0), **Prop. F** (53: 3: 11: 0) and **Prop. Y** (43: 7: 15: 0)

**Turland** explained that these three proposals sought to clarify that a holotype could come into existence in two ways. It could either be the one specimen or illustration used by the author or designated by the author as the nomenclatural type. Prop. B was intended to make the distinction clearer, noting that Art. 40 permitted a holotype to be indicated.

**Sennikov** objected to deleting the word “designated”, because holotypes used to be thought of as designated and now this word had gone away. He did not think it was appropriate. The holotype may be either indicated, designated, or may come into existence by having been used by the author. He proposed to add the word “designated” back into the text.

[The **amendment** was considered **unfriendly** but was supported by **five seconders**.]

**Turland** interjected, before the vote, that the word “designated” seemed superfluous. If the type was designated, then it must have been indicated. A type could be indicated without designating it, but not designated without being indicated.

[The amendment was **rejected**.]

**Gereau** noted that Art. 9.1 was one of the most frequently misunderstood articles in the *Code.* He felt that the proposal clarified this misunderstood point, and it would save time for nomenclatural editors trying to get rid of unnecessary lectotypifications.

**Greuter** posed a question to the proposer, asking whether using the word “indicated” would mean that a single specimen cited in the protologue would be regarded as the holotype. He gave an example of an author writing, “I have seen a number of materials” and citing a single specimen, which he would consider an obligate lectotype. He asked if under the proposed amendment the cited specimen would now be a holotype. Additionally, he proposed that if Prop. B was defeated, it should be sent to the Special-purpose Committee for Typification.

**Prado**, one of the proposers, pointed out that Prop. B had been discussed with Turland when it was being prepared. He said they had some doubts about the words, “indicated” or “designated”, as Turland had explained, and chose the wording to be parallel to the other articles in the *Code* that say “indicated” and not “designated”.

**Xiang-Yun Zhu** recommended that, for the purposes of this Article, the meaning of “one specimen” should be clearly defined.

**Dhabe** added that “indicated” meant that the author might have indicated, designated or used a specimen. He considered the literal meanings of these two words and thought “designated” or “used” were better than “indicated”.

**Turland** responded to Greuter’s question regarding the indication of the [holo]type. Under the second part of Art. 40.3, for the name of a new species or infraspecific taxon, mention of a single specimen, or gathering, or illustration, even if that element was not explicitly designated as the type, was acceptable as indication of the type on or after 1 January 1958. He thought only under very specific circumstances would a single specimen cited in the protologue be an indication of the [holo]type under this wording.

**Art. 9, Prop. B** was **accepted**.

**Turland** reminded the Section that since Prop. B was accepted it would be logical to accept Prop. F and Prop. Y. Prop. F reworded Art. 9.2 so that it no longer implied that a holotype was indicated by the author (when instead it might have been used).

**Art. 9, Prop. F** and **Prop. Y** were **accepted**.

**Art. 9, Prop. C** (57: 2: 19: 0)

**Turland** explained that this proposal was a clarification that the word “element” in Art. 9, Note 1 meant a specimen or an illustration. He suggested it was merely editorial and could be sent to the Editorial Committee.

**Art. 9, Prop. C** was **accepted**.

**Art. 9, Prop. D** (10: 52: 5: 0) was **rejected** based on the **mail vote**.

**Art. 9, Prop. E** (8: 2: *56: 1)

**Turland** introduced the proposal by saying it concerned Example 2 under Art. 9.1, which implied that obvious errors in the indication of a holotype were to be corrected. This proposal would avoid the need to republish the name or propose it for conservation. Ex. 2 was added to the *Melbourne Code* by the Editorial Committee, but it did not actually illustrate an actual provision of the *Code*. Turland also felt it should be specified that omissions of required information were not correctable. This change would need to be reformulated as an Article and the Rapporteurs had suggested that those who agreed should vote “ed.c.” in the mail vote. The Rapporteurs were therefore proposing an amendment that the proposed Note should instead be an Article, in which it should be specified that omissions of required information were not correctable.

[The **amendment** was supported by **five seconders**.]

**Sennikov** commented that he had an Example that could be added to this provision, if accepted, when a species was redescribed solely because the collection number was found to be incorrect and the author argued that it was not a holotype designation.

**Turland** asked that Examples be sent to one of the Bureau members for consideration by the Editorial Committee.

[The **amendment** was **accepted**.]

**Art. 9, Prop. E** was **accepted as amended**.

**Art. 9, Prop. F** was discussed under **Art. 9, Prop. B**.

**Art. 9, Prop. G** (8: 2: 56: 0) was **automatically** sent to the **Editorial Committee**.

**Art. 9, Prop. H** (73: 2: 3: 0)

**Turland** thought this proposal addressed an important issue and noted it had a similar aim to Art. 9 Prop. K. However, if Prop. H was accepted, Prop. K would be redundant. He then invited the proposer to comment.

**McNeill** explained that the proposal addressed a situation that virtually everyone had assumed right back to the Leningrad Congress [1975] or earlier: that an illustration included in the protologue was part of the original material. Original material was redefined to refer to specimens or illustrations used by the author for the description of the taxon. However, rarely was the illustration so used by the author; it was often prepared on the instructions of the author by an artist, while the description was prepared by the author using the specimen that had been illustrated. Many types had been designated that were illustrations associated with the protologues, and if this proposal were not accepted, a large number of names would not have been validly published.

**Art. 9, Prop. H** was **accepted**.

**Art. 9, Prop. I** (59: 1: 7: 0)

**Turland** introduced the proposal saying it covered situations where, for example, a protologue cited an illustration from a previous publication. Under the current rules one could argue it was not original material if that illustration had not been the basis of the validating description or diagnosis. The proposal was to change the wording so that if the author associated that illustration or specimen with a taxon, it would be original material. If a specimen was annotated by the author with the name of the new taxon and the author had written “*typus*” or “*holotypus*” then that would satisfy this Article.

**McNeill** expounded on this, saying that the phrase “which it can be shown” required explanation. For example, did it require written evidence, a reference or some indication, or perhaps knowledge of the plants that the author worked on including unpublished material such as specimens annotated by the author? He noted that this was particularly true for Linnaean typification. It had been generally assumed that all material that was studied by the author prior to the publication was original material. This proposal was to try and encapsulate this in simple words.

**Sennikov** thought the proposal changed the definition of “original material” and would affect typifications that had already been carried out. However, he argued that many people had missed the recent change, which stated that original material included uncited specimens and cited and uncited illustrations on which the description or diagnosis was based. He added that his colleagues dealing with cryptogams had asked him to express their concern regarding sketches made depicting some details of their study organisms. Those sketches were mostly unpublished but could be considered drawings and so under this definition could be interpreted as original material. He pointed out that this situation was not restricted to cryptogams but also occurred in macrophytes.

**McNeill** did not think that this would change much because unpublished illustrations were always part of original material. He did admit that he had not considered sketches accompanying a specimen but did not see why, if they were the only remaining material, they should not be eligible for typification.

**Art. 9, Prop. I** was **accepted**.

**Art. 9, Prop. J** (6: 20: *41: 0)

**Turland** summarized the proposal as expanding Art. 9.3 from “description or diagnosis validating the name” to “description, diagnosis or other material validating the name”. The “other material” that the proposer had in mind was an illustration with analysis. The Rapporteurs commented that the proposed wording would not make this clear unless “other material” were replaced with “illustration with analysis” and the reference to Art. 38.1(a) were deleted. Those who agreed had been urged to vote “ed.c.” in the mail vote, so this was a proposed amendment from the Rapporteurs to replace “other material” with “illustration with analysis” and to delete the reference to Art. 38.1(a), which would no longer be needed because it would be made explicit.

[The **amendment** was accepted as **friendly**.]

**Applequist** thought the language was clunky because it now referred to an illustration upon which it could be shown that an illustration with analysis was based.

**Turland** argued that it was implicit from the *Code* that an illustration with analysis for the purposes of validating a name was equivalent to a description or diagnosis, and perhaps it was not necessary to specify. If one were to accept the spirit of the *Code* it was not necessary, but if one were following the letter of the *Code* then perhaps those cases where one wanted to accept illustrations with analysis together with descriptions or diagnosis in this Article needed to be specified.

**Sennikov** pointed out that an Example was supplied along with the proposal.

**Turland** asked the Section to bear in mind the previous two proposals that had been accepted. The current discussion may not be necessary because “upon which it can be shown” from Art. 9.3 was removed by accepting the previous proposals. Therefore, this proposal was no longer relevant because Art. 9.3 had already been amended.

**McNeill** thought an illustration with analysis was the equivalent of a description or diagnosis and fitting it into the wording of Art. 9.3 would be difficult, but that it could be added as a Note. He had heard people suggest that an illustration with analysis was not a description or diagnosis. Although logically it was treated as such in the *Code*, he proposed a friendly amendment for it to be made explicit by the judgement of the Editorial Committee.

[The **amendment** was accepted as **friendly**.]

**Watson** pointed out that Art. 38.7 already stated, “For the purpose of Art. 38.5, prior to 1 January 1908, an illustration with analysis is acceptable in place of a written description or diagnosis”. He suggested a cross-reference to Art. 38.7 instead.

**McNeill** accepted that a cross-reference might be desirable for ease of reference, but it also had to be referred to in Art. 9.

**Knapp** suggested a vote on instructing the Editorial Committee to make clear in Art. 9.3 that a description, a diagnosis, and an illustration with analysis were equivalent.

**Art. 9, Prop. J** was **accepted as amended**.

**Art. 9, Prop. K** (21: 35: 10: 0) was **automatically rejected** because Prop. H and Prop. I had been accepted.

**Art. 9, Prop. L** (11: 43: 13: 0)

**Turland** explained that this proposal added a new paragraph and three Examples. If accepted, the three Examples would automatically go to the Editorial Committee. The Rapporteurs considered that the perceived problem could be more simply solved by Art. 9 Prop. I, which had been accepted, making Prop. L redundant.

**Prop. L** was **automatically rejected**.

**Art. 9, Prop. M** (24: 26: *13: 1) and **Prop. N** (27: 31: 5: 1)

**Turland** explained that Prop. M ruled that an illustration may not be designated as the lectotype of the name of a fungus unless it showed, in the opinion of the typifying author(s), features diagnostic of the taxon. The proposed new rule would take effect from 1 January 2019. The Nomenclature Committee for Fungi supported the proposal. The Rapporteurs suggested that the wording could be amended to “unless the typifying authors include a statement that it shows features diagnostic of the taxon”, otherwise the author’s opinion would have to be discerned and this could be quite difficult. Turland invited the proposer to comment.

**Hawksworth** gave some background, saying this situation arose when original material was an illustration that had to be considered as a lectotype. There were cases when these could not be interpreted, and people had designated epitypes to support these bad lectotypes. The epitypes themselves had not been sufficiently diagnostic, but a new epitype could not be chosen. He thought Turland’s amendment to include a statement made by the authors would be fine.

[The Rapporteurs’ **amendment** was accepted as **friendly**.]

**Barrie** asked what would happen if the only element available was an illustration that was not in conflict with the protologue and therefore could not be rejected, meaning that neotypification was not possible, but that illustration also did *not* show features diagnostic of the taxon in the opinion of the typifying author. In this case the only element that the rules currently say should be used would have to be rejected.

**Hawksworth** stated that this was the whole idea: the proposal would make it possible to reject an original illustration that would otherwise have to be used as a lectotype.

**De Lange** proposed a friendly amendment to include plants and algae.

[This was accepted as a **friendly amendment**.]

**Turland** noted that the proposer had invited the Nomenclature Section to consider if Prop. M and Prop. N should be applied to all organisms treated under the *Code*.

**Gereau** said he did not understand the purpose of the proposal at all. He suggested epitypes already applied if the only available original material did not show the diagnostic characteristics of the taxon. If an already designated epitype did not show the diagnostic characters, it could be superseded.

**Knapp** pointed out that this could only be done by conservation.

**Gereau** agreed it could be superseded by conservation. He asked what would be solved by rejecting lectotype material that could be epitypified.

**Redhead** suggested the change would save time as it was much quicker to not accept the inadequate illustrations or photographs and then allow neotypification. There were many cases for fungi in particular where a black and white picture of a fuzzy colony on a plate or in a tube was an illustration of the organism, which showed almost nothing. It was cases like this that he thought may have precipitated the original proposal.

**McNeill** suggested that as the proposal stood it would need considerable editorial change. It would have to specify that an illustration that did not, in the opinion of a typifying author, show the features diagnostic of the taxon would cease to be original material. However, you could not get around the fact that it was still original material, without some other provision in the *Code* and better wording to ensure that it was not ambiguous.

**Applequist** foresaw two problems: firstly, if the concern was that an unidentifiable lectotype might be epitypified with an unidentifiable epitype and you instead prohibit the lectotypification, the person would instead pick that unidentifiable specimen as the neotype, and this would not be any better.

Secondly, she had thought at first that this proposal was more “creeping MycoCode”, but now that the proposal was being applied to everything, she presumed that Art. 9 Prop. N would get the same friendly amendment. She suggested it would have had a higher “no” vote in the mail ballot if botanists recognized that it might be applied to them as well.

**Price** pointed out that the Section had tabled Art. 8 Prop. N, “*8.3bis*. For the purpose of typification an illustration is a work…”, etc. This had been tabled to be discussed on Friday, 21^st^ July and the use of “illustration” here was linked to the deferred proposal. She asked for clarification on the relationship between the tabled discussion and what was being discussed now.

**Turland** explained that the deferred Art. 8 Prop. N was a definition of what an illustration was. The current proposal, Art. 9 Prop. M, was placing a restriction on designating an illustration as a lectotype.

**Kirk** was also confused. If the authors said the illustration was not diagnostic, would they have to say why? Or was it just a statement that allowed them to ignore the illustration? If the authors thought that the illustration had features that were not diagnostic, would they have to state what those features were and why they were not diagnostic?

**Turland** thought it was the other way around. If an author wished to designate an illustration as the lectotype, then they must include a statement that the illustration did show features diagnostic of the taxon.

**Kirk** agreed that he had it the wrong way around, but asked if an author wanted to use the illustration, would they have to qualify why they thought the features were diagnostic?

**Turland** replied that under the current wording they would just have to include a statement that it showed diagnostic features, but the statement did not necessarily have to be demonstrably true.

**Barrie** was concerned that because the proposal had now been opened up to all types of organisms under the *Code*, it was going to create huge problems as currently written. He asked if there would be a starting point for it to take effect.

**Turland** said it would start on 1 January 2019.

**Barrie** proposed that Art. 9 Prop. M be sent to the Special-purpose Committee on Typification.

[The **proposal** was supported by **five seconders**.]

**Turland** asked whether a “yes” vote meant accepting the proposal and a “no” vote meant referring it to the Special-purpose Committee?

**Knapp** explained the vote would be whether it should be sent to the Special-purpose Committee or not.

**Monro** pointed out that this was contrary to what the Section had done earlier.

**Turland** and **Knapp** agreed that it did not make sense to follow that procedure, but Knapp decided that the Section should be consistent and vote on the proposal as amended. If defeated, the proposal would go to the Special-purpose Committee. If accepted, it would go into the *Code* as amended.

**Turland** offered the choice of voting “no” for those who felt that the current wording was not well thought out and needed work, and for those who agreed with the various issues that had been raised but supported it going to a Special-purpose Committee.

**Knapp** noted that a “yes” vote would mean accepting the proposal going into the *Code*.

**Turland** pointed out that this did not leave the option of rejecting it altogether.

**Knapp** explained that the Section would first vote to send it to a Special-purpose Committee or not. If it was not sent to a Special-purpose Committee there would then be a vote to accept it or reject it. She promised to look up the correct procedure in the rules that evening.

**Malécot** asked if the procedure would be to vote whether to reject the proposal first and then send it to the Special-purpose Committee as was done previously.

**Knapp** explained that a proposal had been made to send the proposal to a Special-purpose Committee. That proposal was seconded by five people and was now under discussion. The Section would now vote on that proposal, and if accepted Prop. M would go to the Special-purpose Committee. If the proposal to send it to the Special-purpose Committee was defeated, then the vote would be whether to accept or reject the proposal.

**McNeill** said if the proposal were rejected, a Special-purpose Committee could always look at it later.

**Knapp** agreed, saying there was no rule that said the Committee could not look at a proposal if it was defeated. She reiterated that the correct procedure in this case was that a proposal had been made from the floor with five seconders, that proposal was under discussion, would now be voted on, and required a greater than 50% majority to pass.

[A show of hands apparently indicated that a simple majority was in favour of sending Prop. M to the Special-purpose Committee on Typification.]

**Hawksworth** called for a **card vote** on sending Prop. M to the **Special-purpose Committee on Typification**.

**Knapp** proposed, dependent upon the results from the card vote for Art. 9 Prop. M, that the Section apply the same result to Art. 9 Prop. N, because they were very intimately linked and dealt with the same procedure.

[The **proposal** to treat Prop. M and Prop. N in the same way was supported by **five seconders**.]

**Knapp** pointed out that some people appeared to be voting with two hands. [*Laughter*] She admonished delegates and pointed out that it was not allowed, but she had not noticed anybody doing it before.

**Paton** asked whether the Section should first decide whether Art. 9 Prop. N applied to plants and fungi, as opposed to only fungi as was currently written.

**Knapp** explained that the proposer had agreed to amend it to all organisms for both Prop. M and Prop. N at the same time.

**Hawksworth** agreed.

**Knapp** pointed out that he would not dare contradict her now. [*Laughter*]

**Dorr** was surprised that the President would ask the Section to accept something that had already been voted on. He pointed out that the vote was being coupled with something that was not discussed before the card votes were cast.

**Knapp** agreed to vote on her proposal now, which was that both proposals be treated the same: if Prop. M went to a Special-purpose Committee, so too would Prop. N. If the vote to refer the proposals to a Special-purpose Committee did not pass, each would then be voted on separately.

[The **proposal** to treat Prop. M and Prop. N in the same way was **accepted.**]

**Knapp** suggested this would be a good place to stop for the day and closed the formal proceedings, while encouraging delegates to remain present until the results of the card vote were announced.

**Art. 9, Prop. M** and **Prop. N** were sent to a **Special-purpose Committee on Typification** based on the **card vote** (277 yes: 250 no; 52.6% yes).

### Tuesday, 18^th^ July 2017, Morning Session

#### Governance of the *Code* discussion

**Knapp** thanked everyone for attending day two and announced that the day would start with a half-hour discussion about some of the governance issues that would come up for votes on Friday. She explained that the Section took proposals to change the *Code* from beginning to end and the last thing the Section would do was to vote on any changes proposed in Division III, which governed both the Nomenclature Section and the *Code*. A half-hour discussion about some of the issues at this point would allow people to get up to speed, so that everyone would be informed about the changes and what they might mean.

Knapp went on to outline that the discussion would be limited to the two major proposals: the proposal to change the whole of Div. III, and the proposal to change issues in the governance of nomenclature of fungi. The purpose of the discussion would be to ask questions, make comments and try to understand what the changes might mean, as many people may not have read them in their entirety. This would not be the time to make amendments or vote on the proposals.

Knapp said she would first invite a member of the Special Committee on By-laws for the Nomenclature Section, Pat Herendeen, to talk about Div. III; then she would invite Tom May, the Convener/Secretary of the Special Subcommittee on Governance of the *Code* with Respect to Fungi, to speak.

**Herendeen** thanked Knapp and noted that the current Div. III of the *Code* was minimal and inadequate in many respects and that many procedural details were not written down or spelled out. In Melbourne [2011] it was decided that something more explicit was required to explain how the process worked. The Special Committee on By-laws was set up to do that. Herendeen noted that a lot of the decisions were based on institutional memory and, judging from the quantity of grey hair around the room, that institutional memory was getting old. There were 18 members on the Committee. Knapp was chair of that Committee, or made it work, and led it with great skill.

The Committee had worked through the whole process of the six-year cycle, writing down all the procedures that had been traditionally followed. They then debated everything, including whether the word “governance” was appropriate. The Committee then condensed the procedures into a sensible plan, debated it all and voted on each section. The more contentious parts were discussed and voted on multiple times. In the end, they came up with Proposal 286, which was accompanied by an article in *Taxon* that explained the process and the thought behind it.

A subcommittee was formed to specifically address procedures related to fungi. Much of the overall Div. III was not controversial and Herendeen did not expect much discussion or debate about it. The few points that were debated at length and took some time to reach conclusions were expected to be more of a focus for discussion. Herendeen assumed that most discussion would revolve around the mycological proposals and, as these would use the proposed Div. III as a starting point, he felt it would be sensible to first discuss the proposed Div. III in general before moving on to discuss the mycological component. He hoped that everyone had read the documents and would be ready to talk about them.

**Knapp** thanked Herendeen and asked if there were any comments or questions that people had thought about with respect to the general Div. III proposals. She asked if anyone else on the Committee wanted to say anything.

**Herendeen** noted that there were a good number of people present who were on the Special Committee and Subcommittee.

**Knapp** suggested that those members present would be available to discuss any concerns in private, should that be preferred.

**Thiele** introduced himself as one of the members of the By-laws Committee. He agreed that much of the work that the Special Committee had done was relatively straightforward and they had done a good job working through, in good detail, the general issues around governance. One contentious issue that Thiele was passionate about, and which was played out at great length, revolved around voting proportions in the Section with respect to General Committee reports. This was prompted by the whole issue around *Acacia* in the period between the Vienna [2005] and Melbourne [2011] Nomenclature Sections. The question of the voting proportions was crucial in that whole argument.

Thiele had raised a minor issue with Turland earlier. Turland had mentioned on day one that the voting mechanism adopted for this, Shenzhen, meeting was the same as that which had been used at Melbourne. This was true except for the voting proportions with respect to the General Committee reports. The voting adopted for this meeting was, in fact, the voting that had been agreed by the By-laws Committee. Thiele noted that he intended to raise the issue of voting proportions when the Section came to debate it, because he felt there was an extremely important issue there that went to the heart of governance, of checks and balances, and of how the relative powers should be distributed between the Section and the Permanent Nomenclature Committees.

Thiele emphasized that there were some important issues to be debated on this subject and encouraged everyone to think about those issues, to read the relevant sections and part of the discussion, and to talk to anyone on the Committee who was involved.

**Herendeen** invited Thiele to provide more detail on his concerns so that people had time to think about them.

**Thiele** described his take on the issue: anyone involved in the Vienna Nomenclature Section would know that a majority of people (he thought 54%) at that meeting voted against the proposal to conserve *Acacia* with an Australian type. But the voting rules in effect at that meeting meant that a supermajority [60%] was required to overturn the decision that the General Committee had made to conserve the name *Acacia* with an Australian type. Hence, a majority of the Vienna Section voted against it, but that majority opinion was defeated, because it had not reached the required supermajority. Looking at the processes that were now set out and formalized in the newly proposed Div. III, the Permanent Nomenclature Committees would require a supermajority to come to a decision on a matter and to promote that decision to the next level.

The Committee for Vascular Plants needed a supermajority to accept a proposal to conserve or reject a name or for all the other matters that came before it. The matter then went to the General Committee, which also needed a supermajority to accept it and to promote that decision to the Section for ratification. The issue came down to what the required proportion of the Section should be to overturn a decision of the General Committee and of the other Permanent Nomenclature Committees.

Thiele believed it was critical that a Nomenclature Section should be able to overturn a decision of the Permanent Nomenclature Committees and the General Committee. However, it should be difficult to do so and there should be a high bar. The question really came down to questions of democracy. There was substantial expertise in the Committees. When matters were sent to them, they considered them at great depth and with an enormous amount of skill, and then two levels of a supermajority were required for an issue to then come to the Nomenclature Section.

In every case before *Acacia*, passing the recommendations of the General Committee had been done by a show of hands and was always unanimous. *Acacia* was the controversy that sparked all of this. Thiele reiterated his view that it should be possible to reject a General Committee recommendation. But it should be difficult to do so, and it should require a supermajority.

**Herendeen** confirmed that this was a simple majority versus supermajority and that the requirement came into play at each level in the decision-making process. He asked if there were any other issues or if people now wanted to turn to the mycological question.

**Knapp** asked if there were any comments from people who were not on the Committee. She reiterated that the merits of different voting percentages would not be debated at this time but urged the delegates to read the report of the Special Committee and to look at the revised Div. III before Friday when it would be debated prior to a vote.

**Funk** wondered what the thinking was behind the changing of one of the roles of the General Committee regarding voting on proposals that came up from the other Permanent Nomenclature Committees.

**Turland** stated that there was, in the proposed new Div. III, a change in the voting procedure from what had previously been standard practice. According to the new proposal if a specialist committee (e.g. the Committee for Algae, the Committee for Bryophytes, the Committee for Vascular Plants) was unable to make a recommendation after voting three times, the Committee would be considered to have recommended against the proposal or against making a binding decision. If the General Committee could not make a recommendation – if the required majority was not achieved – the matter would be referred back to the specialist committee for further consideration.

Turland explained that currently, if a specialist committee could not make a decision it could request that the General Committee make a decision instead. The General Committee had the power to make a decision instead of the specialist committee. Also, the General Committee had the power to overrule or reverse a recommendation of a specialist committee. Under the current procedure, which was not enshrined in the *Code* at all but was followed by “tradition”, the General Committee had more power and it was possible for a name to become conserved or rejected, for example, upon the decision of one Committee, the General Committee, and it could go straight to the Congress for approval.

Under the rules proposed in the new Div. III, however, every decision that came to an IBC for ratification, must have been approved by two committees: a specialist committee and then the General Committee.

**Herendeen** added that if specialist committees could not make a decision on a proposal after three attempts, the proposal should not move forward and should be viewed as rejected. The authors of the proposal could take the feedback from the specialist committee and try again if they wished to, but the original proposal was finished. In terms of the General Committee approving a decision of the specialist committee, the General Committee, according to the new by-laws, could *approve*, but they could not disapprove. In this way there would be a negotiation or a conversation between the specialist committee and the General Committee. The proposal could go back and forth between them until the specialist committee either decided that the proposal should not be recommended or convinced the General Committee that it should be approved. This was the set-up proposed in the new Div. III.

**Funk** asked why the changes had been proposed and wondered if something had not been working.

**Turland** explained that the Special Committee on By-laws had considered the new proposals as a more robust mechanism for reaching a final recommendation that could go before the Nomenclature Section for approval. It was all about checks and balances. For example, if a proposal to conserve a name was to be approved by a specialist committee and then by the General Committee, it would be a robust proposal recommended by two committees. Whereas, if the specialist committee could not decide and was split 50/50 and asked the General Committee to make a decision on their behalf, the proposal to conserve a name would only have General Committee approval. If the proposal concerned an algal name, for example, there were very few phycologists on the General Committee, therefore a much less robust recommendation would come before the Nomenclature Section.

**Herendeen** agreed that it was relying on the taxonomic expertise of those individual specialist committees. He then suggested turning to the mycological aspect of Div. III to see what people had to say about that.

**Knapp** agreed. She noted the importance of two areas in which a considerable amount of debate had occurred in the By-laws Committee. One area dealt with how proposals went from specialist committees to the General Committee and how things came to the Section. The reasoning behind that, as Turland and Herendeen had said, was about broadening participation and making robust decisions that were not dependent on a single fulcrum point. The second area concerned the voting percentages, which were being tried out in Shenzhen. Some of these were also used in Melbourne, but as Thiele had pointed out, those in use in Shenzhen were not exactly the same.

**Turland** added that the most contentious issue that the Committee had discussed was the subject of voting percentages: when a General Committee recommendation came before a Nomenclature Section, should it be approved with a 50% simple majority, or a 60% qualified majority? The Special Committee on By-laws debated this at great length. This subject was saved until last, as they knew it was going to be contentious, and it was discussed in great detail, with many complicated voting mechanisms devised by Knapp to ensure that it was fairly debated and voted upon. The Committee agreed on their decision, which could be seen in Proposal 286. Turland pointed out that Thiele was also a member of the specialist committee.

**Knapp** momentarily stood aside from her role as a completely neutral Chair to clarify that the Special Committee on By-laws had devised a mechanism by which a single General Committee recommendation could be singled out from the General Committee report. That is, a mechanism by which a single decision could be voted on separately from the rest of the report. This was sparked by the controversy over *Acacia* that happened in both Vienna and Melbourne. Knapp pointed out that this controversy should not happen again because the proposed procedures were different. However, the voting percentage was about the General Committee report and the controversy was not so much about the whole of the General Committee report, but rather about a single recommendation therein. That there would now be a mechanism for singling out a particularly contentious recommendation meant that the voting had to be thought about in a slightly different way. Knapp suggested that the delegates go back and look at the two issues so that everyone could think about the proposal before it was amended, debated and discussed later in the week.

Knapp then invited May to come up and talk about his Subcommittee’s work on the governance of fungal nomenclature. This had been very closely and tightly linked to the work that had been done on Div. III and had built upon that in an important way.

**May** explained that he was speaking to the set of proposals that came out of the Special Subcommittee on Governance of the *Code* with Respect to Fungi. He asked all the mycologists in the room to raise their hands and counted 10 delegates. May noted that, as has always been the tradition, there was good representation of mycologists at Nomenclature Sections and there had been very strong engagement with the apparatus of the *Code* from mycologists. Mycologists served on the Nomenclature Committee for Fungi and on the General Committee. They were represented across the different Special Committees. They were active as editors, in various roles, in *Taxon*. Mycologists had always been embedded in the apparatus of nomenclature. May wanted to stress at this point that whatever mention there might be about a “MycoCode”, this was *not* on the table. This was *not* something that had support from mycologists, and he wished to take it off the table straight away.

May stated that, in terms of engagement with mycologists, at the IMCs over the last several decades there had been formally organized Nomenclature sessions with debates, discussions and voting. There was good engagement from mycologists in those sessions, with 100 or more people attending. These sessions included voting and discussion on important modifications that had ended up in the *Code*. The move to Latin/English was initiated by the mycologists, put up as a proposal solely for fungi, but in Melbourne it had been accepted for all organisms. The change to the *Code* title which, in May’s opinion, had been enormously significant in bringing together algae, fungi, and plants under this umbrella, and which he felt was moving out into general parlance, was very influential and had been initiated by mycologists. Registration, another innovation, was brought in by mycologists and was in practice already for mycology.

The big issue that had been engaging mycologists for the last several decades was the move towards one name, one fungus. Most of the discussions on this were carried out at Mycological Congresses and then brought to the Nomenclature Section of the IBC. May was happy to note that when mycologists had wished for changes in the *Code* specifically related to fungi, they had received a good reception from Nomenclature Sections. In general, the wishes of the mycologists had been respected and changes had occurred. However, he thought that a more democratic system was required. The few mycologists attending the Nomenclature Sections carried the wishes of the many. May felt it was important to understand that mycology was a separate discipline now: the great conference [IBC] that many of the audience would be attending the following week would have 6000 botanists and no mycology would be discussed there.

May reminded the Section that mycology was a separate discipline with its own Congress, attended by 1000–2000 people and there was an active interest in nomenclature at that Congress. Rather than having the few mycologists at the present Nomenclature Section, albeit that they were engaged in the apparatus of the *Code* carrying the wishes of the many, what the Special Subcommittee had proposed was that a formal Nomenclature session at a Mycological Congress would enact formal decisions about matters solely relating to fungi.

May stressed that the proposed mycological Nomenclature Session would deal only with matters solely relating to fungi. A very useful amendment, which May regarded as friendly, had been put forward on the suggestion of Knapp and Greuter. The amendment was to assemble the matter in the *Code* that solely related to fungi into a separate section or chapter. Regarding this subject matter, only the starting point date would have to be repeated in both places. The new section or chapter would include sanctioning, a special procedure that solely related to fungi. May pointed out that the only other matters solely related to fungi were: registration, which would now include the registration of later typification acts for fungi; Art. 59 on pleomorphic fungi; and some special provisions in Art. 13, Art. 14 and Art. 56.3 regarding the preparation of lists by international working groups, which had been introduced at the Melbourne Congress. These were the kinds of things that mycologists would like to be able to amend and produce innovations in.

Referring to the extensive and excellent Special Committee on By-laws that had come up with a codification of practice, May wanted to stress that in preparing the formal proposals, the Special Subcommittee had been extremely careful to exactly replicate all of the procedures in the new Div. III and would be quite happy to modify any of them if any shifts occurred as a result of debate about Div. III. In this apparatus there would be a Bureau of Nomenclature, Rapporteurs, a President, a mail vote and a synopsis of proposals. The only detail that would differ would be that they would prefer not to have institutional votes. Otherwise, every aspect of the current, formal Nomenclature Section would be replicated at an IMC to deal with matters solely related to fungi. What those matters were would be decided by the Nomenclature Committee for Fungi in consultation with the General Committee. There were a number of points in the procedures where the General Committee would be involved in consultation. It would be anchored into the existing apparatus of the *Code*. May emphasized that the proposal was not some schism or separation: it would just be taking the procedures that were utilized at the current Nomenclature Section and completely duplicating them at International Mycological Congresses to deal with matters specifically related to fungi.

May stressed that the proposal had extremely strong support from within the mycological community. It was supported by a majority of the Nomenclature Committee for Fungi. It was supported by a majority of the International Commission on the Taxonomy of Fungi (a body of the International Union of Microbiological Societies [IUMS]). Key international mycological bodies strongly supported this; 19 members of the Executive Committee of the International Mycological Association (IMA), who responded to a request for their opinion, were all in favour of this move. So there was strong support, in terms of governance, from international bodies. Of course, the IMA organized the IMC.

In closing, May reiterated that mycologists wanted to stay in the family. Mycologists saw the *Code* as many-layered. There were fundamental parts of the *Code* – the Preamble, typification, orthography – that covered all organisms. Mycologists wanted to stay with that, but there were particularities about fungi. Fungi were a different kingdom, a sister to animals rather than to plants. Phylogenetically, there were a lot of biological differences of fungi that necessitated some nomenclatural differences. The Special Subcommittee thought it would be better for a large group of mycologists to make an informed decision about these issues, but they would be entirely replicating, as far as possible, the procedures that operated at the IBC Nomenclature Section, with all of the checks and balances that existed in that apparatus. May stated that he would be happy to take questions on any issues over the next couple of days and encouraged the delegates to come and talk to him, stating that mycologists were very open to working on how to get this proposal up before the Section.

**Levin** asked if there were any things in the *Code* that pertained only to plants or only to algae.

**Turland** noted that there were a few rules in the *Code* that pertained solely to algae. In terms of plants, apart from starting dates, there was very little.

**Wiersema** replied that some rules pertained only to fossils.

**Levin** explained that the reason he had asked the question was that if a group of people who were responsible for one group of organisms wanted to vote on things that affected them only, it would similarly make sense for them to be the only ones who voted on their proposals.

**May** thought this was a good point and it was possible that delegates might have heard phycologists discussing this. May stressed, however, that with mycology this was the end point of several decades of engagement with the process. In time, if other groups were able to get to the same level of sophistication and organization, that could well be appropriate, remembering that only a very small number of Articles of the *Code* would be involved. May stated that the mycologists wanted to keep this about mycology because they had spent several decades getting to this end point.

**Dorr** asked how frequently the IMC would convene and how they would coincide with the cycle that the IBC had of every six years if things became effective only when the Congress ratified them.

**May** thought this was a good question. The Mycological Congresses were on a four-year cycle. The next one would take place the following year (2018) in Puerto Rico, so they were not aligned with the IBC. The proposal was that changes to matters solely relating to fungi that had been approved by the plenary session of an IMC would go into the *Code* and would go live from that point.

**Redhead** made the point that there were mycologists at the IBC, even though they did not consider themselves botanists, therefore the botanists would be most welcome to attend the IMC.

**May** said this was an excellent point and, in their proposal, part of the formal process was that the Rapporteur-général for the Nomenclature Section of the IBC would be cordially invited to attend the Nomenclature Session of the IMC. They were very pleased that Turland had attended the informal nomenclature session at the IMC in Bangkok [2014]. May stated that they would very much like to have these interconnections.

**Applequist** noted that the proposed fungal chapter already necessarily included one feature – registration of names – that was more of a philosophical issue or governance issue than something mandated by the life history or taxonomic history of the group in question. She asked what assurances there would be that other such philosophical issues, such as the radical redefinition of “type” that was apparently supported by some mycologists, would not also be placed into this chapter.

**May** answered that in terms of the organization of material, if and when registration became more widely applied, then the fungal component of that would be a mere cross-reference in the fungal chapter. In terms of what kinds of things mycologists might consider, May thought it important to remember that these would come through a process exactly the same as that which was operating in the Nomenclature Section and formal proposals would have to be published in advance. There would have to be a Rapporteurs’ synopsis and so on. If mycologists decided on different changes that were appropriate to fungi because of the nature of the organism, that would be okay. But such changes would come not just from some sort of fly-by-night thought process. It would be a long process as happens with changes to the *Code* in general. It would not be possible to give assurances about what may or may not happen, but the apparatus created for the Nomenclature Section worked very well and they had faith that the same apparatus would work for the mycologists.

**Seregin** noted that here at the Shenzhen Congress there were 245 proposals being voted on, and he asked May if he would be happy to accept the results of all these proposals or whether some of the accepted, general proposals might be voted against at the next Mycological Congress.

**May** thought it was clear, from the type of material mycologists had been bringing to Congresses over the years, that there was engagement by mycologists on general issues, but it was envisaged that there would be a small number of proposals for a mycological Nomenclature Session, and these would be specifically related to fungi because that would be the only mandate given. There would not be 250 or 300 proposals; more likely a small number would be discussed. May stressed that there was nothing in the proposal about being able to overturn anything else that was agreed in the IBC Nomenclature Section.

**Seregin** asked if the general proposals would definitely be accepted from the Nomenclature Section.

**May** stated that the name would be Nomenclature Session (rather than Section), and the Rapporteurs would be called Secretaries and so on to keep it separate. The Nomenclature Session of an IMC would only be dealing with proposals published in the lead-up to the IMC that specifically related to fungi. It would not come back and look at what was done at an IBC Nomenclature Section. He explained that it would be an onward process where new proposals were addressed.

**Knapp** wished to clarify the point raised by Seregin: should the Nomenclature Section vote on something generally at an IBC that applied to all the organisms covered by the *Code*, that would be accepted *de facto* by the mycological Nomenclature Session. Issues pertaining only to fungi, for example the Articles about sanctioning that did not apply to vascular plants, would be the kinds of Articles that could be amended in a Nomenclature Session on fungi.

**Dorr** noted that proposals for the Nomenclature Section were published in *Taxon* and asked if the mycological proposals would be published in *Taxon* or in *Mycologia* or another journal.

**May** answered that proposals specifically related to fungi would be published in *IMA Fungus*, the journal of the International Mycological Association.

**Funk** said she was sort of sad because there may be less participation from fungal colleagues at IBC Nomenclature Sections, as they would be deciding on a lot of the things that would be particularly related to fungi in a separate place, and the proposals would be published in a separate place. She thought it would be the crack in the icefield, that everyone would become more and more separated and eventually the connection between the two communities would break.

**May** said he had thought about that a lot, but predicted that at the next IBC Nomenclature Section he would see the same engagement by mycologists because they were deeply embedded in the apparatus of the *Code*. He noted that there were mycologists on the General Committee. There were people editing various parts of *Taxon* and he had started to publish the reports of the Committee for Fungi in both *Taxon* and *IMA Fungus*; it would be appropriate, and they would like to maintain the communication. May speculated that the synopsis of proposals for a fungal Nomenclature Session could be published in *Taxon*. They wanted to keep in touch about it. May reiterated that this was not a schism nor a split, it was just a more democratic way of handling the decisions specifically relating to fungi and he thought that in six years’ time there would be the same representation of mycologists at the IBC.

**Turland** added that about 90% of the *Code* concerned issues relating to all groups: algae, fungi, and plants. The IBC was still going to be important to mycologists because most of the provisions of the *Code* were going to continue to apply to fungi. This was not a schism at all, the two disciplines were still going to be very much linked.

**Greuter** was grateful to May for the way he explained his case because it made one feel that there was real and justified concern behind what was being proposed. He thought that May had explained the rationale of these proposals in a very sympathetic way, which was hard to contradict. Greuter also felt that the proposals were a coherent package. However, he was worried about technicalities. For example, if the proposals should pass, would there be a *Shenzhen Code*, a post-*Shenzhen Code* 1, and a post-*Shenzhen Code* 2 before the next IBC *Code*?

**May** explained that it would not be necessary to have a separate name and *Code*. What they would need to include in the printed version of the *Code* and in the online one, in the separate chapter for fungi, would be a note or warning to mycologists to consult the current online version of the *Code* for any changes specifically related to fungi that might have been inserted at a subsequent mycological conference. These would be the only bits of the *Code* that mycologists would be empowered to change. May thought that most people accessed the *Code* online and that there would be a statement in the printed copy for mycologists to check. Any changes enacted at Puerto Rico would go live into the online *Code*. In support of Turland’s point, 40 or 50 other articles would remain exactly the same and it would only be the chapter on fungi that would have the potential to change between Botanical Congresses.

**Greuter** argued that historically we had not always had a published *Code* at our disposal within one year after the last Congress. When he entered nomenclature, the delay was more likely to be three years and there was no guarantee that this may not happen again in the future. The present very speedy production of *Codes*, largely due to the current Rapporteur-général and the Editorial Committee working under his apt authority, should not be taken for granted. Greuter went on to point out that at a Nomenclature Section, the first action carried out was the approval of the previous *Code* as the basis for discussion. This would not be possible for the next IMC, as it would be unlikely that by that date they would have a *Shenzhen Code* to base themselves upon. The same would probably apply to the next IBC Nomenclature Section, which would not have a post-*Shenzhen Code* 2 already published to discuss and to base their deliberations on.

Greuter, therefore, wondered whether it would be possible, or whether it had been considered, to go a different way: to say that the mycologists’ concerns could be equally well covered by delegating the real responsible decisions on fungal matters to the IMCs, where changes to whatever was proposed from the past would require a supermajority and then, say, that such changes approved by a supermajority at an IMC, when referring only to fungal organisms, would need a supermajority to be overturned by the next IBC.

**Knapp** was concerned that the conversation was now becoming concerned with making proposals to amend and to discuss details.

**Greuter** disagreed and explained that his question was to ask if any such possibility had been considered.

**May** answered in the negative but said that over the next couple of days he would be extremely happy to discuss any options.

**Levin** asked if there was any mechanism for dealing with new solely fungal issues: for example, splitting off things that historically had pertained to algae, fungi, and plants and now saying this would be a new rule that might pertain only to fungi.

**May** stated that this was exactly what they wanted to do. This was the nub of it: what would be discussed at a Nomenclature Session of an IMC would be proposals concerning Articles or Recommendations specifically pertaining to fungi.

**Levin** asked, hyperbolically, if mycologists wanted to say that red was blue, would they feel that they would be empowered to make that kind of decision?

**May** asked everyone to think about the mycological Nomenclature Session as being like the group of people sitting in the room at the Nomenclature Section in Shenzhen. There would be a measured discussion. There would have to have been a proposal beforehand and a synopsis by the Rapporteurs. There would be discussion and so on. Mycologists were not going to try to make red blue.

**Levin** noted that the Section dealt with a proposal the previous day that initially was suggested only for fungi and asked if the mycologists would have been able to deal with that all on their own. If so, there would be a very different process than that seen at the Nomenclature Section.

**May** had not wanted to talk about this particular proposal but, as the subject had been raised, he explained that as far as the “DNA as type” proposal was concerned, it had been raised as a proposal in the same way as other proposals had been raised. The DNA as type had not been accepted by the current Section, nor would it be accepted at this point at an IMC. Just because an idea was floating around and had been proposed by a mycologist did not mean it was going to be accepted. May assured Levin that this proposal would not be accepted at an IMC because of the reasons discussed the previous day. He noted that there were always proposals to amend the *Code* that were a bit “out there”. Any changes in relation to fungi would come after a measured and engaged debate by the mycological community. If, in the end, there were some changes that departed from the way plants were dealt with, it would be because fungi were different. There was a different biology and there was a different need for the nomenclature attached to it.

**Levin** joked that alternatively it might be because mycologists were smarter than the rest of the delegates and they had come up with a great idea that had not occurred to others. [*Laughter*]

**Knapp** thanked May and Herendeen for helping to enlighten the delegates a little bit more about some of the changes proposed, which were going to be important for the whole community because, unlike other communities that governed organisms, this community made changes as a group and did things in a much more participatory way than, for example, the zoological community. Knapp felt that one of the great powers of what she might call “plant-algal-fungal nomenclature” as compared to zoology was that there was community participation, debate and discussion, consideration of different reports and an apparatus. Knapp explained that this apparatus was one of the community’s great powers. One of the impetuses behind looking at governance was thinking about how to strengthen this community aspect that other communities of organisms did not have. Knapp noted that these were her personal feelings on the matter, but now she was going to be the President again and be completely impartial! She suggested the Section start from where it left off the previous day with Art. 9 Prop. O.

#### Article 9 (continued)

**Turland** asked if Knapp would first explain about the Special-purpose Committee mechanisms and how they worked.

**Knapp** thanked Turland for reminding her that there had been some confusion the previous day about voting to send proposals to a Special-purpose Committee and about how that worked. Knapp had since consulted her various sources about how they should do this and declared that the way it would work for the rest of the Section was that if someone moved that a proposal go to a Special-purpose Committee, it would be called a “motion to commit”. A motion to commit would stop debate on the proposal being discussed. Knapp explained, for example, that if Art. 9 Prop. O was being debated and someone proposed that the Article being discussed, perhaps at the end of the day when everyone was tired, should be sent to a Special-purpose Committee, then that motion would then be on the table. The Section would discuss and debate the motion to send the original proposal to a Special-purpose Committee and would then vote on whether to send it to such a Committee. If there was a positive vote to send it, off it would go to the Special-purpose Committee. If the Section voted not to so send it, the Section would go back to debating the original proposal and vote on it yes or no. This procedure would be followed from now on.

Knapp explained that she had got slightly confused the day before because she was told to do something different. She went on to recap what happened at the end of the previous day: Art. 9 Props M and N, regarding the diagnostic features of illustrations, were discussed and there were various proposals and amendments put forward. There was then a motion to send these proposals to a Special-purpose Committee. The Section had voted the previous day to send both of those proposals to the Special-purpose Committee on Typification.

Knapp announced that the Section would now start the day’s business with Art. 9 Prop. O, but mentioned she had forgotten to announce that Funk, as Secretary of the Nominating Committee, would like to meet with either the Chair or the Secretary of each of the Permanent Nomenclature Committees sometime that day to discuss the slates for nominations. Funk also wished to meet anyone who had not been involved to date in any of the specialist committees, but who would be interested in participating. There was no need to be an expert. Knapp explained that expertise came with practice and part of gaining expertise was to serve on committees. She reiterated that anybody who would be interested in participating should talk to Funk and discuss with her what this might entail. Knapp noted that a lot of people were interested but perhaps felt like it was something of a closed shop. She stressed that this was not the case.

**Redhead** wished to ask a question on the procedure that the Section had just been talking about. Two Special-purpose Committees on DNA as Types and on Typification in general had been agreed to, but he wanted to know how and when those Special-purpose Committees would be set up.

**Knapp** replied that this would be done on the last day of the proceedings. In the Melbourne Section [2011] various Special Committees were proposed, including the Special Committee on By-laws for the Nomenclature Section and the Special Subcommittee on Governance of the *Code* with Respect to Fungi. People signed up for the Committees and then, in discussion with the General Committee, a decision was made on who would be the conveners and secretaries of the Committees. The Committees were then assembled afterwards. So, it might not only be people who were present at the Section who would be on those Special-purpose Committees.

**Turland** noted that there would be a General Committee report published after the Congress announcing the Special-purpose Committees.

**Knapp** tried to summarize the point, noting that the Special-purpose Committees would be assembled and worked out on the last day. These would be discussed over some time and there would then be a General Committee report published. As to the Permanent Nomenclature Committees, there would be a proposal put forward for slates for these, as traditionally done by the Nominating Committee, on the last day, so there would be plenty of time to think about it.

**Art. 9, Prop. O** (48: 0: 18: 0)

**Turland** explained that Art. 9 Prop. O was almost editorial. It proposed to add a note to point out that a duplicate specimen of a conserved type could be logically equated with an isotype. Turland stated that it was important to point out that the word “*isotypus*” had been used in this sense in the Appendices of the *Code*, mainly App. IV, since the *Tokyo Code* of 1994. It would be a Note because it would be explaining something that was implicit elsewhere in the rules but may not be immediately obvious.

**Applequist** asked if the proposer would accept a friendly amendment, to say “the term isotype is also used for a duplicate of the type of the conserved name”.

[The **amendment** was accepted as **friendly**.]

**Knapp** asked Gereau if he had a comment, but it was noted that Gereau had wanted to make the same comment as Applequist. Knapp pointed out that Gereau had done what all good delegates should do: if someone had already said what you wanted to say, you do not say it. She awarded a gold star to Gereau!

**Barrie** thought the *Melbourne Code* said that the term isotype was used for a duplicate of the holotype. It was always a specimen. He wondered if it would be simpler just to change Art. 9.4 to say an isotype was used for a duplicate of a holotype or a conserved type?

**Knapp** thought this might be editorial and McNeill agreed.

**Barrie** suggested, therefore, that the Note would not be needed.

**McNeill** thought Barrie’s suggestion was probably a good one and that one would want to look at the actual wording, so this was an editorial matter. If the wording was incorporated into the Article, then the Note would be redundant. The inclusion or exclusion of a Note was editorial, and so the matter could be entirely dealt with if it was sent to the Editorial Committee.

**Art. 9, Prop. O** was **accepted as amended** and sent to the **Editorial Committee**.

**Art. 9, Prop. P** (2: 0: 75: 1) was **automatically** sent to the **Editorial Committee**.

**Art. 9, Prop. Q** (6: 71: 1: 0), **Prop. R** (4: 67: 7: 0) and **Prop. S** (6: 59: 1: 0) were **rejected** based on the **mail vote**.

**Art. 9, Prop. T** (7: 39: 21: 0)

**Turland** noted that Prop. T would add the words “or has been in existence” to Art. 9.7 at the end of the sentence “A neotype is a specimen or illustration selected to serve as nomenclatural type if no original material is extant”. This seemed to be based on a strict understanding of “no original material is extant” to mean that original material had once existed but did not still exist. Taken literally this could preclude a neotype being designated for a name that never had any original material. If the proposal was accepted, the Rapporteurs wondered if the Editorial Committee might replace “is extant” with “exists” throughout Art. 9, including editorial changes in Art. 9.13 and Art. 9.12. Turland suggested this as a friendly amendment.

[The **amendment** was accepted as **friendly**.]

**Knapp** confirmed that the proposal would be amended to say “A neotype is a specimen or illustration selected to serve as a nomenclatural type if no original material exists or as long as it is missing”.

**Gereau** pointed out that with the amendment absolutely nothing had changed. “Extant” meant “exists”. He argued that there was absolutely no change and the proposed example misinterpreted “original material”. So, there was no improvement and no reason to approve this proposal.

**Turland** argued to the contrary. He had checked the Oxford English Dictionary because he had also been confused initially. There was a sense of the word “extant” that implied still in existence, as opposed to merely exists. So, there was a subtle difference in meaning but the proposed amendment got around any possible confusion.

**Greuter** asked if the “Gordian knot” could be cut by sending the proposal to the Editorial Committee, rather than approving it.

**Knapp** stated that, if approved, it would go to the Editorial Committee. She believed Turland was asking for a mandate to send the proposal to the Editorial Committee to make it work, to change the meaning.

**Turland** said the proposal could be approved or sent to the Editorial Committee. If the Section voted to send it to the Editorial Committee, then the Editorial Committee would adjust the wording appropriately if necessary. If it was approved as amended, it would read “exists”, unless the Editorial Committee felt that there was something wrong with that.

**Knapp** stated that to send this to the Editorial Committee someone had to make a proposal. She pointed out that the procedure in this case was that the Section had been voting on a proposal. It was now voting on an amended proposal. No one had proposed to send the proposal to the Editorial Committee. The Section was not voting on sending it to the Editorial Committee, but was voting to approve it, or to not approve it.

**Turland** pointed out that Greuter had just proposed to send it to the Editorial Committee.

[The **proposal** was **seconded**.]

**Knapp** clarified that the Section was now discussing whether to send the proposal to the Editorial Committee. As discussed earlier that morning, if the Section voted to commit this proposal to the Editorial Committee, it would go to the Editorial Committee and it would not be discussed again.

[The **proposal** was **accepted**.]

**Applequist** asked if the Section were to vote on the proposal in this fashion, would it make the *Vriesia* example a voted Example?

**Knapp** replied in the negative and asserted that only Examples that had been proposed as voted Examples would be treated as such. Any Examples accompanying proposals would go to the Editorial Committee for scrutiny. No proposed Examples would automatically go into the *Code*.

She declared that the Section would now vote on Art. 9 Prop. T, to amend Art. 9.7. She confirmed that the Example associated with this change would go to the Editorial Committee in the same way that all other Examples would go to the Editorial Committee.

**Art. 9, Prop. T** was **accepted as amended**.

**Art. 9, Prop. U** (19: 41: 7: 0)

**Turland** explained that Prop. U concerned epitypes in Art. 9.8 and noted that the Rapporteurs had some concerns. The opinion of the epitypifying author that a type could not be critically identified would replace the need to demonstrate its ambiguity, however nebulous in interpretation that current requirement might be, and would perhaps lower the standard for undertaking such an epitypification. Turland cautioned that the permanent nature of epitypification with respect to the interpretation of a name should be considered, therefore, the effect of this proposed change would need to be carefully considered. Turland reminded delegates that once an epitype was designated it could not be overridden, or superseded. The typification of the name could then only be changed through conservation. The Rapporteurs were somewhat concerned about this proposal.

**McNeill** stated that, while he did not want to stop discussion on this proposal, he thought it should be sent to the Special-purpose Committee on Typification: not because of the wording of the proposal, but because of the *lack* of wording. He felt that currently it was unclear what would happen if a type was *not* demonstrably ambiguous. There was no provision by which an epitype could be overturned in such a case. There was no obligation even for an author to state that the type was demonstrably ambiguous. This proposal, he suggested, went a step forward in that respect in requiring, by implication, that the author considered the type to be ambiguous. He agreed with the Rapporteurs opinion that epitypification was such an important process that there should be some provision. In fact, he thought there should be a requirement to indicate ambiguity, and if that was not met the epitypification should not be effective. This would require a new wording of the *Code* which was not the wording set out in the proposal, therefore, he felt it was a good case to go to the Special-purpose Committee on Typification.

**Greuter** proposed to send Prop. U to the Special-purpose Committee.

[The **proposal** was **accepted**.]

**Art. 9, Prop. U** was sent to the **Special-purpose Committee on Typification**.

**Art. 9, Prop. V** (39: 13: 13: 0)

**Wiersema**, as the primary author of this proposal, explained that in Art. 9.8, which discussed establishing epitypes, there was nothing to prevent someone from designating an epitype to replace a conserved type. The article only talked about when the holotype, lectotype, or previously designated neotype were demonstrably ambiguous. The proposal was designed to preclude the possibility of anyone designating an epitype in the case of a conserved type.

**Kirk** corrected Wiersema’s language, noting that an epitype did not replace a primary type, it supported a primary type. Speaking as a mycologist, Kirk explained that most new taxa and existing taxa were now defined based on molecular data. Kirk suggested that most of the conserved types would not be suitable for that process as they may be illustrations or ancient types with poor quality DNA. The option to propose an epitype to support a conserved type was, in his opinion, a good thing to have.

**Sennikov** believed the proposal provided a significant limitation. It did not look like a Note, but was more like a provision and he suggested, as a friendly amendment, that it should be changed from a Note to an Article. [The **amendment** was considered **unfriendly** and was not supported by **five seconders**.]

**McNeill** was also not sure that the proposal should be in the form of a Note. It appeared to him that there was nothing in the *Code* that precluded one from epitypifying the type of a conserved name. As Kirk had just pointed out, the type did not change, it was merely the interpretation of the type. He proposed that the Note should be an Article.

**Wiersema** agreed that the type would not change but changing the interpretation of the type was fairly serious and could change the application of the name. Wiersema asked if delegates would want this for something that had gone through the process of conservation, had been evaluated by committees and had ended up in the *Code*. He suggested that such a decision could be revisited and it was possible to conserve a name with a new type. He asked if delegates wanted a procedure to simply have someone epitypify it, or would it be preferable for it to go back and be re-evaluated in the same procedure by which it was conserved in the first place? This could either be in the form of an Article or a Note.

**McNeill** wished to clarify that he was not in the least opposed to the proposal. He believed that Wiersema had just confirmed that it should be an Article because, as he had just explained, it would be terrible to epitypify the type of a conserved name. If it was not possible to do this under the *Code*, which must be the case if it was a Note, then no one should worry about it. However, McNeill thought that it could be done and warned that it would be bad to do it.

**Turland** explained that it was not expressly forbidden under the current rules, because the *Code* stated that “An epitype is a specimen or illustration selected to serve as an interpretive type when the holotype, lectotype, or previously designated neotype, or all original material associated with a validly published name, is demonstrably ambiguous”. As it did not mention conserved types there, it was not expressly allowed, and was not expressly forbidden.

**Redhead** stated that he supported the idea in principle, but wished to point out that the only way to change an epitype was via conservation, therefore, the process would go round in circles: if someone were to designate an epitype to a conserved type, and people decided that they did not actually match taxonomically, then they would have to go down the conservation route in some way again, for the same name, because that was the only way to get rid of an epitype.

**Barrie** pointed out that there were types in the *Code* that were there but were not formally conserved, even though they were treated as though they were conserved [because they were the types of conserved names]. He did not think that there was anything to worry about as the current wording said nothing about conserved types. He believed that a type that had been formally conserved could not, under the current wording, get an epitype. There might be an argument about types that were simply cited for [conserved] names that had a holotype or previously designated lectotype, that were listed in the Appendices. Even though they could not be changed without conservation, Barrie was not sure that the current wording would protect them. Barrie was, therefore, in favour of McNeill’s proposal that the Note should be an Article.

**Gereau** said that he was in favour of the Note as it was written, and as a Note. He felt it probably avoided disruption of the application of a conserved name. However, as some of the consequences of it seemed uncertain, he thought it should be sent to the Editorial Committee.

**Greuter** stated that he had been listening to deviating interpretations of what the *Code* currently said and the desirability of having it say what was proposed in the new Note. In view of the uncertainty, he proposed that it was a clear case to go to the Special-purpose Committee for further examination.

[The **proposal** was **accepted**.]

**Art. 9, Prop. V** was sent to the **Special-purpose Committee on Typification**.

**Art. 9, Prop. W** (10: 7: 47: 2)

**Knapp** suggested that this proposal be sent straight to the **Editorial Committee**.

**Hawksworth** stated that this Example was particularly important because it was the basis for Art. 9 Prop. U. He felt that the Example would make a change because there was no demonstration of ambiguity in it and proposed that it should be a voted Example.

**Knapp** pointed out that this would be a new proposal and would have to come before the Section on Friday.

**Hawksworth** argued that the proposal was already being discussed.

**Knapp** did not know if the proposer was present to accept Hawksworth’s proposal as a friendly amendment.

**Turland** wished to consult a copy of the synopsis, as there was something relevant to the proposal in there.

**Knapp** stated that if Prop. W related to Prop. U, and Prop. U had been sent to the Special-purpose Committee, then Prop. W should be treated the same way. Knapp confirmed to Turland that the Section was discussing the *Salicornia* Example in Art. 9 Prop. W. She pointed out that this was an Example and that delegates had voted at the beginning of the Section to send proposals which concerned only Examples, except voted Examples, to the Editorial Committee.

**Hawksworth** commented that the proposer had originally wanted this to be a voted Example.

**Turland**, having found the relevant section in the synopsis, agreed that the proposer wanted “the relevant ruling bodies to consider if this should not be entered as a Voted Example”. Turland then read the Rapporteurs’ response: “If this were the case it would not be clear what aspects of nomenclatural practice the Voted Example was intended to govern”. On the one hand “that a lectotype may be demonstrably ambiguous without molecular testing, or that an epitype in that case is to be molecularly tested, or that an epitype is to be from the type locality, or any combination of these. As a regular Example, however, it would illustrate these principles”. Turland explained that a voted Example was intended to govern a particular aspect of the *Code*. If this Example were made a voted Example it would be unclear what it was supposed to be governing.

**Paton** stated that while the Example as proposed might refer to Prop. U, it would also work perfectly well as an example of how to apply the term of an epitype. Given that Prop. U was sent to the Special-purpose Committee, Paton thought that this should also be dealt with as a normal Example and should, therefore, be sent straight to the Editorial Committee.

[The **proposal** to amend Prop. W to become a voted Example was **rejected**.]

**Art. 9, Prop. W** was sent to the **Editorial Committee**.

**Art. 9, Prop. X** (10: 41: 16: 0) was **automatically** sent to the **Editorial Committee**.

**Art. 9, Prop. Y** was discussed under **Art. 9, Prop. B**.

**Art. 9, Prop. Z** is discussed under **Prop. DD**.

**Art. 9, Prop. AA** (10: 52: 2: 1) was **rejected** based on the **mail vote**.

**Art. 9, Prop. BB** (34: 16: 14: 1)

**Turland** explained that this proposal concerned the relative precedence of different kinds of types and original material in lectotype designation. Art. 9.12 of the *Code* implied that a syntype and an isosyntype had equal precedence but did not make this entirely clear. A proposal to the Melbourne Congress [2011] to give a syntype precedence over an isosyntype was defeated in the mail vote. This was a clear message that they should be considered as having equal priority. Prop. BB made this message explicit by saying, “otherwise a syntype or an isosyntype, if such exists”. Turland pointed out that the deletion of, in parentheses, “duplicate of syntype”, was contingent on the acceptance of Rec. 9C Prop. A, which came later. Therefore, the discussion should focus simply on putting “or isosyntype” after “syntype”.

**Art. 9, Prop. BB** was **accepted**.

**Art. 9, Prop. CC** (20: 40: 7: 0)

**Turland** noted that this proposal was to add an extra sentence at the end of Art. 9.14, explaining what should happen in a case where a type contained parts belonging to more than one taxon.

**Seregin** wished to point out that Prop. CC and Prop. DD were, in his opinion, clear alternatives. He asked delegates to bear this in mind when voting on Prop. CC.

**Proćków** stated that this was his proposal. He asked Yun-Fei Deng to show Art. 9.17 on the screen as an excellent example of how a problem was outlined and how instructions were then given to illustrate how the problem should be dealt with. In Art. 9.14, however, the problem was outlined but there were no specific instructions provided to show how the problem should be resolved. Proćków explained that his proposal would strengthen the Article by providing instructions on how the problem should be dealt with, removing the possibility of doing something wrong.

**McNeill** wished to endorse what Seregin had said, that Prop. CC and DD were alternatives. McNeill preferred Prop. DD.

**Art. 9, Prop. CC** was **rejected**.

**Art. 9, Prop. DD** (28: 30: 9: 0) and **Prop. Z** (33: 21: 11: 0)

**Turland** explained that these two proposals were linked. Prop. DD also concerned Art. 9.14 and would provide a different and more detailed procedure to that of Prop. CC that had just been rejected: “an admixture may be disregarded provided that the validating description or diagnosis does not apply to it. Otherwise the type should” – or he thought “may” would be a better word – “be narrowed to a single ‘element’ by subsequent lecto- or neotypification”. Turland added that this would presumably be carried out in the way that best served nomenclatural stability, which was not mentioned but which was implicit in Ex. 11*ter* and 11*quater*, following the Article. Turland went on to note that Prop. Z was connected with Prop. DD and would insert in Art. 9.11 an apposite reference concerning admixtures to the revised Art. 9.14 of Prop. DD. Prop. Z was dependent on whatever was done with Prop. DD. Acceptance of Prop. DD would refer Prop. Z to the Editorial Committee.

**Redhead** was concerned that if a type was demonstrably ambiguous, how would it be possible to designate a neotype when a type existed, as was implied by this change? He stated that if a change was being made which superseded an existing type, it had to be a conserved type.

**McNeill** suggested that he should leave the proposer to answer this point but noted that the proposal included any type and the admixture might be included in a neotype, so [subsequent] neotypification may be needed. He agreed with Redhead that the wording was ambiguous.

**Redhead** stated that the proposal was worded in such an ambiguous way that it did not preclude the idea that a neotype could be designated for an existing type, which did not make sense. He concluded that there was something wrong with the wording.

**Gereau** noted that, by allowing an admixture to be excluded without a separate nomenclatural act, the proposal would allow undesirable ambiguity to remain in the application of a name and it should be rejected.

**Barrie** did not agree with Gereau because if, for example, there was a type of a *Cuscuta*, the host plant could be present but only the *Cuscuta* part would be considered part of the type. The host plant, even though it might take up more volume on the specimen, would be an admixture. The host plant would not be part of the description and excluding it would not create any ambiguity. Barrie stated that he would be supporting the proposal.

**Schori** wondered how portions could be excluded without a separate nomenclatural act and was curious as to what that would look like. She stated that the way to do this currently was to put “p.p.” (*pro parte*) down in a type designation, but she was unsure if that would be sufficient. The proposal did not provide a clear way to do what it was intended to do.

**Knapp** invited the proposer to answer the question posed by Schori.

**Sennikov** stated that the proposal had three Examples, which covered different situations in macrophytes and microphytes. It was especially applicable to cases where a potential type specimen or designated type might be represented by several individuals: vascular plants mounted on a single herbarium sheet; a preparation with several small individuals, or a package with many items inside, some of which belonged to the type and others of which did not. In some cases those items were demonstrably different things…

**Knapp** interrupted Sennikov, noting that he was explaining the rationale behind the proposal, not answering Schori’s question.

**Sennikov** explained that there were Examples.

**Knapp** asked Sennikov if he was suggesting that the Examples answered the question.

**Sennikov** agreed.

**Redhead** voiced his concern over the proposal. He liked the *Code* the way it was. Mycology now required registration for nomenclatural acts of typification, so it would either be accepted or not accepted if it was registered in the future. He also wished to point out that in mycology specimens often contained multiple elements, for example a piece of bark with some lichens, hyphomycetes etc. Mycologists knew that if something existed there that was described, then that part was part of the type and they did not worry about all the extraneous material. However, they did encounter problems when people had described in the protologue elements that contained obvious parts of different organisms or species. Redhead, therefore, concluded that he was still concerned about the wording and preferred to retain the wording from the old *Code*.

**Struwe** wondered how this proposal would affect palaeontology collections when there were many species on the same rock for the same collection, but only one of the organisms was a type. She asked if all such instances would require lectotypification.

**Knapp** sought out a “fossil person” to answer the question. She picked out Herendeen, whom she noted was not actually fossilized yet but was getting close. [*Laughter*]

**Herendeen** introduced himself as Pat Herendeen, Chicago Botanic Garden, fossil person. [*Laughter*] He went on to state that a palaeobotanist would never do such a thing. He explained that they worked with rock specimens, coalball slabs that had many different taxa on them. They would be looking at one particular specimen and designating a type, or whatever, and it would not be an issue because palaeobotanists would not be so stupid as to do that. [*Laughter*]

**Thiele** proposed that the proposal be sent to the Special-purpose Committee on Typification and that delegates go to tea.

**Art. 9, Prop. DD** and linked **Prop. Z** were sent to the **Special-purpose Committee on Typification**.

[*The Section broke for morning tea*.]

**Knapp** welcomed delegates back after the morning break with a threat that if Art. 9 was not finished before lunchtime, no one would have lunch.

**Art. 9, Prop. EE** (32: 23: 11: 0)

**Turland** explained that Art. 9 Prop. EE was contingent on Art. 9 Prop. DD. As Prop. DD had been sent to the Special-purpose Committee on Typification, he proposed that so too should Prop. EE.

**Art. 9, Prop. EE** was sent to the **Special-purpose Committee on Typification**.

**Art. 9, Prop. FF** (2: 2: 60: 1) was **automatically** sent to the **Editorial Committee**.

**Art. 9, Prop. GG** (39: 18: 9: 0)

**Turland** explained that this proposal sought to provide some explicit rules on what may be done when a previously designated neotype had been lost or destroyed. He referred delegates to the Rapporteurs’ comments but added that if a substitute neotype was designated – which was optional – must it be chosen from among the isoneotypes if such existed? If this was the intention, then it was not quite clear from the wording of the proposal. Turland also wished to mention that the proposal assumed that the first neotype was a good specimen that supported current usage; therefore, if it were lost, we would want to have a duplicate of it, which presumably would be the same taxon. There were some potential concerns with the proposal.

**Redhead** stated that he had some concerns about the proposal itself, not the actual wording. He wished to go on record as saying that the example given of *Psilocybe atrobrunnea*, where the neotype had been lost, and which therefore served as a justification for the proposal, had never been explicitly designated as a neotype. It was, as far as Redhead was concerned, an inadvertent neotype; therefore he did not know of an actual existing neotype that had been lost. He would be looking for a real example where this proposal might apply.

**Barrie** noted that what was described in the proposal was something that might be good in practice but, as the Rapporteur-général said, there would be situations where it would not be desirable to be forced to take a duplicate of a neotype. He thought the proposal was too restrictive in its legislation. It may be something that people should be encouraged to do, but people should not be forced to do it because there may be better choices.

**Schori** pointed out that, as currently worded, the Article said, “a substitute for it may be designated from among the isoneotypes if such exists”. This would allow for it, but the Article did not say “must”; therefore she thought Barrie’s comment was not quite accurate.

**Wiersema** noted that the Rapporteurs mentioned this in their comments. The wording was “may be chosen”, not “must”. Thus, a substitute may be designated from among the isoneotypes. The proposal did not say “must”, so it was not mandatory.

**Barrie** made the point that if it was not considered something that people were required to do, then it was a Recommendation and not an Article, assuming it was accepted.

**Knapp** asked if Barrie was making a proposal.

**Barrie** said that he could make a proposal to turn the Article into a Recommendation but, personally, he would rather see the proposal disappear.

**Knapp** suggested, therefore, that Barrie should not propose to turn it into a Recommendation.

**Turland** did not think that new provisions should be introduced into the *Code* for situations that were extremely rare or hypothetical and this, to him, seemed to be one of those situations. He said he would be reluctant to make any changes in this case.

**Miller** made the point that while the first sentence said “may be designated from among the isoneotypes”, which was not mandatory, the second sentence said “if none exists”, implying that only if none existed could another suitable element be chosen. Miller thought this was being more prescriptive, but also thought that it was not well worded. Miller thought that, as Turland had implied, it referred to an extremely rare case. In this situation one would want to be able to choose from any eligible, reasonable best material.

**Freire-Fierro** asked how the first neotype would be distinguished from the second neotype, as it seemed like another term would need to be used for the subsequently designated neotype.

**Sennikov** did not think this Article would apply only to hypothetical situations because specimens, including type specimens, could be destroyed at any time. He was concerned because a neotype was supposed to be a well-chosen specimen. If a neotype was lost and had to be replaced by an isoneotype, that replicate could be quite a bad specimen. He thought that it would be better to have a provision in case the neotype got lost to allow any good specimen to be designated instead.

**Greuter** stated that, knowing herbaria for what they were, the loss of a neotype was not necessarily a very exceptional or rare situation. He was aware of many cases in which herbaria had lost designated types, including neotypes. He also made the point that, being associated with a herbarium that had been destroyed in its entirety in the last World War, which he hoped would not be a situation that repeated itself too often, the loss of neotypes must be considered as a serious possibility. Greuter also wished to make the point that the verb “may” in this case was appropriate because “should” would be a recommendation. He said that “may” was an enabling verb. Greuter noted that there was no obligation under the *Code* to designate a type at all for old names. A name could be left untypified and no one could object. He felt, therefore, that “may” here was enabling or providing the way in which, if wanted, a replacement type may be designated for a destroyed or lost neotype and, as such, it was entirely appropriate, including the choice of verb. In view of the uncertainty that had been demonstrated by some of the comments that had been made, Greuter proposed to send the proposal to the famous Special-purpose Committee, which would be pleased to receive it.

**Knapp** asked for seconders for Greuter’s proposal.

[The **proposal** was supported by **five seconders**.]

**Wiersema** interrupted proceedings, noting that Soreng had a comment to make.

**Knapp** explained that a proposal had been made to send Prop. GG to the Special-purpose Committee, therefore, the Section had to discuss that proposal.

**Gereau** felt strongly that the proposal was excessively restrictive. It would prevent the choice of other suitable material and should simply be voted down and not sent anywhere.

**Soreng** attempted to propose a friendly amendment to the original proposal but Knapp told him he could not do that because the Section was now discussing whether that proposal should be sent to a Special-purpose Committee or not.

**Soreng** argued that Gereau had not talked about that.

**Knapp** disagreed, noting that Gereau had said it should *not* be sent to the Special-purpose Committee.

**Soreng** proposed a friendly amendment to remove the phrase “if such exists” and replace it with “or if none exists another suitable element may be designated”.

**Knapp** noted that this would be an unfriendly amendment as the proposer was not in the room.

**Turland** suggested Greuter could comment as he was a co-author on the proposal but thought Soreng’s amendment was editorial and would not change the meaning.

**Watson** thought perhaps that the situation could be more elegantly dealt with by just inserting brackets, “(or neotype)”, after “designated lectotype” in the existing Art. 9.16.

**Knapp** reminded delegates that the Nomenclature Section was not the place for wordsmithing and reiterated that the present discussion should be focussed on accepting or rejecting the proposal.

**Cantrill** called the question.

[The Section voted **to vote**.]

**Art. 9, Prop. GG** was **rejected**.

**Art. 9, Prop. HH** (30: 24: 11: 1)

**Turland** explained that this proposal was parallel to Prop. GG except that it concerned lectotypes instead of neotypes.

**Knapp** warned that she would only accept comments that were substantially different to those put forward for the previous proposal on neotypes.

**Redhead** said that in his opinion Prop. HH was not exactly parallel to the previous proposal. Whereas Prop. GG said “may” be designated, Prop. HH said “must” be designated, therefore making it a little more concrete. If isotype or isolectotype material was available, but was of poor quality, there was a danger that people would be forced to take that sample as the new lectotype, so there were subtle differences between the two proposals.

**Govaerts** was concerned that this proposal would be retroactive so that if people had already chosen a new type, for example for a holotype destroyed in Berlin, those typifications would now be overruled.

**Schori** believed it would be helpful to have something that specified what to do when a lectotype had been destroyed, as there were no instructions in the *Code* detailing what to do about designating a new lectotype, or in some cases a neotype. She was not sure that the wording of the proposal was the best but thought it would be useful to have guidelines.

**Turland** wished to point out that there was already a provision in the *Code* to say what would happen if a lectotype had been lost or destroyed, in Art. 9.11, “when the holotype or previously designated lectotype has been lost or destroyed…a lectotype or, if permissible, a neotype as a substitute for it may be designated”.

**Applequist** stated that she regarded this proposal as even more undesirable than Prop. GG because syntypes that had actually been seen by the author of the taxon would have to be passed over in favour of isolectotypes that the author had not seen.

**Proćków** stated that if isolectotypes were of very poor quality an epitype could, if necessary, be designated.

**Middleton** suggested that the situation was rather analogous to first- and second-step lectotypifications. If a lectotype was lost, an isolectotype would have to be chosen.

**Greuter** wanted to draw the attention of the Editorial Committee to the problem in the word “previously” in the proposed wording. First, it was not clear immediately what it referred to. Was it previously to loss or destruction, or previously to the designation of the replacement lectotype? He felt that this question was not quite academic because, in many cases, lectotypes had been designated that no longer existed. He thought this would probably best be solved at the discretion of the Editorial Committee by deletion of the word “previously”.

**Knapp** asked if Greuter was making a friendly amendment, but Turland and Greuter agreed that it was editorial.

**Art. 9, Prop. HH** was **rejected**.

**Art. 9, Prop. II** (42: 19: 3: 0)

**Turland** explained that Prop. II would add the concept of two-step epitypification to the *Code*. Two-step lectotypification and two-step neotypification were addressed under Art. 9.17. Turland felt that the proposal appeared to address a hypothetical situation, but the proposers considered it inevitable that, in the future, a designation of an epitype would be found to refer to a single gathering but to more than one specimen. He noted that the proposal would put a provision in the *Code* for a situation that did not currently exist but probably would in the future.

**Redhead** stated that this situation arose in mycology, particularly in going from dual nomenclature to single nomenclature, in that there were original types that were either asexual or sexual. Some people would designate an epitype for whatever sexual state one name applied to and then someone would discover a different sexual state for it and in some cases that was designated as the epitype; the term “teleotype” was coined for such situations. Redhead saw a positive potential here in the proposal, but also saw negative potentials for second-step epitypification. He thought, perhaps, it may be applicable in some way in mycology.

**Art. 9, Prop. II** was **accepted**.

**Art. 9, Prop. JJ** (3: 13: 49: 0)

**Turland** thought this proposal was purely editorial and proposed that it be sent to the Editorial Committee.

**Knapp** said that they could not do that. The proposal could be accepted and would then be sent to the Editorial Committee. Knapp repeated that Turland thought the proposal was purely editorial and a vote to accept would mean that it would go to the Editorial Committee.

**Turland** corrected himself, agreed with Knapp, and repeated that the proposal was purely editorial.

**Gereau** agreed with the Rapporteur-général that the proposal was purely editorial but felt that it provided absolutely no clarification and was not helpful and so should be voted down. It should not go to the Editorial Committee.

**McNeill** stated that, while he did not really like disagreeing with the President, he thought there was a difference between accepting the proposal and sending it to the Editorial Committee. If we accepted it, it would go to the Editorial Committee, but there was an implication that the Section approved it. He, rather like Gereau, was not sure he approved of it, but was happy to let the Editorial Committee have a look at it. If delegates accepted the proposal, he assumed that the Editorial Committee would feel an obligation to do something about it because the Section approved it. McNeill thought there was a distinction here and suggested that maybe the Section should, sometimes, just send things to the Editorial Committee without any implication of approval.

**Knapp** attempted to clarify the concerns raised by McNeill. She noted that there were two points. First, that a proposal would be approved and sent to the Editorial Committee, which meant that the Section accepted that something like it should be added to the *Code*. If, on the other hand, a proposal was sent straight to the Editorial Committee, there was no opportunity for the Section to reject it. Knapp, therefore, decided to take a step back and run through a hypothetical situation: if the Section was happy for something to go into the *Code* for the Editorial Committee to take a look at, then the Section should vote to accept it. If the Section was unhappy about something going into the *Code* and thought it was unnecessary, then it should be rejected. However, if the Section sent something straight to the Editorial Committee, there was no opportunity to reject it.

**Turland** added, “unless people vote not to send it to the Editorial Committee”, in which case the Section had to choose “yes” or “no”.

**Knapp** agreed that this was the correct way forward. The Section would vote on whether to commit a proposal to the Editorial Committee. If the Section did not want to send the proposal to the Editorial Committee, then the proposal itself would be discussed. This would work in the same way as sending a proposal to a Special-purpose Committee. Knapp went on to explain that, in a discussion about the situation over lunch, the manner of how to proceed was not clear because sending things to an Editorial Committee was not what normally happened in debating and parliamentary assemblies. Knapp admitted that it was a somewhat anomalous situation, but she wanted to see how the process would work and suggested that the Section try it out. Regarding Prop. JJ, Knapp confirmed that Turland had made a proposal to send this to the Editorial Committee, so the Section would now vote on that proposal.

**Art. 9, Prop. JJ** was sent to the **Editorial Committee**.

**Knapp** confirmed that this was the procedure that would be followed: someone would have to propose to send something to the Editorial Committee. The Section would then vote on sending it to the Editorial Committee with the proviso that the Editorial Committee could accept it or reject it as they saw fit. A vote counter to that would mean that Section would not want a proposal to go to the Editorial Committee.

**Art. 9, Prop. KK** (8: 53: 4: 1), **Prop. LL** (7: 58: 1: 0) and **Prop. MM** (6: 58: 2: 0) were **rejected** based on the **mail vote**.

**Art. 9, Prop. NN** (50: 11: 5: 1)

**Turland** noted that this proposal sought to improve Art. 9.19, clarifying that both lectotypes and neotypes could be superseded under clause (*b*). It also aimed to allow more latitude when superseding a lectotype. Under the proposed amendment, if there was no non-conflicting element of original material available, the lectotype may be superseded by a neotype. Turland emphasized that this was new and that, under the existing Art. 9.19, the only available options in such a case were either accepting the conflicting lectotype and its nomenclatural consequences or proposing the name for conservation with a conserved type.

**Applequist** noted that McNeill had always told her that a lectotype could not be in conflict with the protologue since it was part of the protologue. The new proposal stated, “non-conflicting element of the original material”. Applequist argued that there could be original material that was not in the protologue and that could be rejected if conflicting. However, if there were other specimens listed in the protologue that conflicted with the standard understanding of the taxon, or with the diagnosis or description, then these would still have to be chosen, according to the rules. Applequist stated that she would love to see that rule go away, but she did not think it should be done half-heartedly in a case where the *Code* would seem to conflict with itself.

**Turland** pointed out that he thought Art. 9 Prop. OO was relevant to this, unless he misunderstood.

**McNeill** said that the thrust of the proposal was to remove a provision that had been in the *Code* since it was introduced in Sydney [1981]. He was one of the people who proposed it at Sydney, but he still did not know why that clause was added. He said that Applequist was perfectly correct, a lectotype could only be in conflict with the protologue if the lectotype was original material that was not cited in the protologue. In other words, it could not be a syntype. That was mentioned in the wording of the proposal. The existing wording said that you could only replace a lectotype that was in serious conflict with the protologue if there was another element available that was not in conflict. He felt that this was an unnecessary restriction and if a lectotype was in conflict with the protologue it should be possible to change it. The thrust of the proposal was simply to allow that to happen. It was not intended to make any other change. Regarding Prop. OO, it was simply pointing out what the *Code* currently said: if an element was cited in the protologue it was part of the protologue and could not be in conflict with it.

**Sennikov** asked McNeill if there was any logical conflict between “any of the original material” and “a non-conflicting element of the original material”.

**Turland** clarified what he believed to be the question: was there any conflict between the existing clause (*a*) and the additions proposed here?

**McNeill** replied that he did not intend there to be any conflict and did not think there was one.

**Turland** agreed because if any of the original material was rediscovered, the superseding lectotype or neotype would be superseded by that.

**Greuter** was uneasy about the meaning of the word “otherwise” in the last line and wondered what it was concretely linked to. It could be linked to “if it is not in serious conflict with the protologue” or “if none exists”.

**McNeill** stated that it was the second option.

**Greuter** said that he would prefer that this be spelled out, so instead of “otherwise” it should say “if none exists”. [This was **accepted** as a **friendly amendment**.]

**Knapp** confirmed that Section was now discussing the proposal as amended.

**Art. 9, Prop. NN** was **accepted as amended**.

**Art. 9, Prop. OO** (48: 6: 10: 1)

**Turland** noted that Prop. OO was independent of Prop. NN and had received a positive mail vote. Prop. OO was editorial because as a Note it was not introducing any new provision into the *Code*, but merely explaining what may not be obvious from other provisions of the *Code*. Because it was editorial, Turland asked if he should dare propose to refer it to the Editorial Committee?

**Art. 9, Prop. OO** was sent to the **Editorial Committee**.

**Art. 9, Prop. PP** (6: 0: 72: 0) was **automatically** sent to the **Editorial Committee**.

**Art. 9, Prop. QQ** (2: 11: *65: 0)

**Turland** noted that this was a proposal to add a Note to Art. 9 to point out that the designation of a lectotype or neotype was not necessarily achieved deliberately, in other words, this covered inadvertent lectotypification or neotypification. Editorially, Turland thought the Note would probably be better placed after Art. 7.10, where it could also apply to epitypes. Some aspects of the wording would need to be changed. The Rapporteurs had pointed out that the use of the word “type” was not correctable under Art. 9.9 because it was not a term defined in Art. 9.1, 9.2 or 9.4–9.8. The specification of the herbarium or institution in which the type was conserved applied only on or after 1 January 1990. Turland therefore reiterated that there were several editorial issues that would need to be fixed. The Rapporteurs had said in their comments that those who wished the Editorial Committee to formulate a suitable Note on inadvertent lecto-, neo- and epitypification under Art. 7.10 should vote “ed.c.”. In the mail vote there were 65 “ed.c.” votes out of 78 votes cast. Turland asked if the proposer would accept this as a friendly amendment.

[The **amendment** was accepted as **friendly**.]

**Turland** stated that the proposal was now for the Editorial Committee to formulate a suitable Note on inadvertent lectotypification, neotypification and epitypification and place it under Art. 7.10.

**Knapp** observed that they were dealing with this proposal in a different way than previous ones. After some hesitation Knapp stated that Art. 9 Prop. QQ sought to instruct the Editorial Committee to compose a suitable Note on inadvertent typification.

**Middleton** was worried that Section was not following the proper procedure. He was also concerned about the inclusion of the word “isotype” in the proposal. He noted that when he saw somebody writing “isotype”, quite often it was deliberate because they thought they had not seen the holotype. If the word “isotype” was to be included as being correctable to lectotype or neotype, he was not sure if that would have been the intention of the author. In many situations, they would have thought that there was a better specimen or a [presumed] holotype, which could be correctable to lectotype, somewhere else.

**Wiersema** noted that there had been some discussion over e-mail about this situation: whether one could correct an isotype to a lectotype or neotype. However, he was not sure how that discussion had been resolved. He asked McNeill and Greuter if they could remember.

**McNeill** did not remember the details that Wiersema alluded to but agreed with Middleton’s assertion that there were people who thought there was a holotype somewhere but had not seen it and so would write “isotype”. His conclusion was that, in this situation, one had to interpret this as correctable to lectotype because, if they thought there was a holotype, but in fact there was not, it would mean that a holotype had never been designated. This would be a situation in which the author had not, in fact, designated a holotype. McNeill went on to say that if somebody cited something as an isotype because they thought there was an implicit holotype, then selecting a specimen from the type gathering and calling it an isotype must be a lectotypification from the holotype gathering. This was why McNeill thought it was correctable to lectotype.

**Dorr** disagreed. He stated that the practice had been that if somebody did not say “type” then it was not considered to be a lectotype. If they said “isotype” then they had not said the magic words. Dorr thought that this was potentially destabilizing if what McNeill said was true.

**Redhead** stated that this sort of situation often arose in mycology and maybe in other areas where people were looking at exsiccatae sets. Because exsiccatae sets were distributed to different herbaria, people looked at parts of a distributed “thing” that they assumed was an isotype, because they assumed that the author’s original herbarium contained the holotype. People talked about the isotype when they looked at one of these exsiccatae packets. Then the mycologists ended up with multiple copies because these things got amalgamated and people hesitated to say that they were looking at the holotype because they were looking at part of a distributed set.

**Knapp** pointed out that the Section was discussing the original wording of the proposal but reminded delegates that it had been amended to be a proposal to instruct the Editorial Committee to formulate something sensible on inadvertent typification.

**Turland** stated that he was aware of how nebulous his proposal sounded. Essentially, he explained, the proposed Note would be the Note that was in the proposal but with the concerns of the Rapporteurs addressed by the Editorial Committee, and it would be under Art. 7.10 rather than Art. 9.

**Knapp** asked Turland to confirm that the friendly amendment was that the Editorial Committee would be instructed to compose a Note taking into account the Rapporteurs’ comments and those things that had been raised by the Section.

**Turland** replied affirmatively and stated that one could propose to refer the proposal to the Editorial Committee, where they could fix it.

**Art. 9, Prop. QQ** was sent to the **Editorial Committee**.

**Art. 9, Prop. RR** (2: 9: 53: 2) and **Prop. SS** (4: 2: 60: 0) were **automatically** sent to the **Editorial Committee**.

**Art. 9, Prop. TT** (7: 57: 2: 0) was **rejected** based on the **mail vote**.

**Proćków** stated that while he respected mail voting, he wanted to draw delegates’ attention to the possibility of having two epitypes because…

**Knapp** interrupted, stating that Proćków could not do this, because the proposal was not being discussed and that he would have to propose to bring the proposal back onto the floor.

[The **proposal** was supported by **five seconders** and was **reintroduced** for discussion.]

**Knapp** opened the floor to discuss Prop. TT to amend the first sentence of Art. 9.20.

**Proćków** wished to look at the possibility of having two epitypes because it was possible to designate a subsequent epitype if the first one was lost or destroyed. However, if the first one was rediscovered, there would be two epitypes. Prop. TT would ensure that the second epitype could be superseded if the original epitype was rediscovered.

**Turland** noted that the Rapporteurs had commented in the synopsis that such a rule would be redundant because Art. 9.20 already required that the author who first designated an epitype must be followed. So, in the case of the first of these two epitypes being rediscovered, the first author would have to be followed.

**Art. 9, Prop. TT** was **rejected**.

**Art. 9, Prop. UU** (24: 33: 9: 0)

**Turland** explained that Prop. UU addressed another hypothetical situation but noted that this situation may well happen in the future. The proposal sought to provide a rule on what may be done when a previously designated epitype had been lost or destroyed. Unlike the neotype situation in Prop. GG, there was no example of a lost or destroyed epitype given. The proposal seemed to be providing a parallel rule to deal with that situation. It would likely be favoured by delegates who appreciated consistency. Turland stated that the Section had discussed a parallel proposal, Prop. HH, concerning lectotypes. Prop. HH had been rejected, as had Prop. GG concerning neotypes. So this proposal was parallel to two rejected proposals.

**Art. 9, Prop. UU** was **rejected**.

**Art. 9, Prop. VV** (56: 5: 4: 0)

**Turland** explained that this proposal added the need to explicitly declare that an epitype had been designated. This would be retroactive to 1 January 2001. The proposer considered that this would not be problematic because identifying a type as an epitype would have been common practice since epitypes first entered the *Code* in the *Tokyo Code* of 1994. It would be unusual for somebody to designate an epitype without using the word “epitypus” or “epitype” or an equivalent. Turland noted that this would reinforce current practice.

**Prop. VV** was **accepted**.

**Art. 9, Prop. WW** (2: 4: 55: 5) was **automatically** sent to the **Editorial Committee**.

#### Recommendation 9A

**Rec. 9A, Prop. A** was discussed under **Art. 8, Prop. J** and **Prop. K**.

**Rec. 9A, Prop. B** (5: 60: 2: 0) was **rejected** based on the **mail vote**.

**Rec. 9A, Prop. C** (15: 42: 10: 0)

**Turland** noted that this proposal recommended against needlessly or senselessly narrowing the choice of a lectotype to a particular part of a specimen unless, for example, the specimen was taxonomically mixed or was suspected to comprise more than one gathering. He quoted the Rapporteurs’ comments, “While the advice seems to be reasonable, the proposed Example could be interpreted as either following or going against the Recommendation”. Turland explained that if the Example was interpreted as following the Recommendation it could set a precedent for narrowed lectotypifications in any instance where parts may have been attached to the specimen at different times. However, he noted that the Example would be considered by the Editorial Committee if the Recommendation were accepted. The Editorial Committee could provide a replacement Example in which a lectotype choice was narrowed on taxonomic grounds.

**Greuter** said that he opposed the proposal on the grounds that it would imply that some type designations, which in his opinion were not effective, could be considered to have been effectively made. If a lectotype was designated that was only part of a specimen, this was not covered by the *Code* because one was not designating a specimen as the lectotype. On the other hand, if there were different taxa on a herbarium sheet, that herbarium sheet was not a specimen. So, the Recommendation was useless or inappropriate because it recommended against something that could not be possible.

**Sennikov** wished to explain the background of the proposal. It seemed to him to be based on the fact that the definition of a specimen was interpreted in different ways by different people. Some people thought that a specimen was a single fragment on a herbarium sheet; therefore, every fragment on a herbarium sheet was a specimen. Other people thought that a specimen was the whole sheet and it did not matter how many fragments it contained. Those who followed the first approach would say that the type must be designated from among those fragments or small individuals, if a species was small enough and was represented by small individuals. The proposal was to discourage the interpretation that a specimen was the same as a fragment on a herbarium sheet.

**Rec. 9A, Prop. C** was **rejected**.

#### Recommendation 9B

**Rec. 9B, Prop. A** (23: 39: 3: 0)

**Turland** noted that this proposal received a strongly negative mail vote. The Rapporteurs had commented on the phrase, “all available evidence”, and noted that this could include post-protologue evidence such as correspondence records or other publications.

**Gereau** said that although this was only a Recommendation, it was a recommendation of bad practice. He noted that people typified as best they could and that taxon names should be typified as well as possible so that the application of names could be fixed. This was the whole purpose of the process and he did not want to discourage it.

**Rec. 9B, Prop. A** was **rejected**.

**Rec. 9B, Prop. B** (61: 9: 7: 0)

**Turland** explained that this was a proposal recommending that authors state why the holotype, lectotype, neotype or original material was ambiguous. When designating an epitype, authors should explain the nature of the ambiguity. The Rapporteurs commented that the phrase, “demonstrably ambiguous”, in Art. 9.8 merely required such demonstration to be possible, not necessarily enacted.

**Dhabe** thought that it was essential for an author, if they designated an epitype, to mention why they wished to use a specimen other than the holotype. For example, it may be a fruiting specimen or maybe a root or any other part of the plant. The author had to support why they were designating an epitype.

**Lindon** also wished to support the proposal noting that, as a content editor for IPNI, she had observed a spate of people designating holotypes and epitypes at the same time in a protologue. She hoped that if this Recommendation were accepted, authors might think twice about doing this, or at least explain to people looking at the protologue why they were doing this at the same time.

**Rec. 9B, Prop. B** was **accepted**.

#### Recommendation 9C

**Rec. 9C, Prop. A** (10: 9: *47: 1)

**Turland** noted that this was a Recommendation explaining that duplicates of lectotypes, neotypes and epitypes were isolectotypes, isoneotypes and isoepitypes, respectively. The proposal sought to convert the Recommendation into a rule and add isosyntype as a duplicate of a syntype. The Rapporteurs, however, questioned whether it should be a rule and thought that it might be better to place the paragraph of Prop. A as a footnote to the word “isosyntype” in Art. 9.3, where it first appeared in the *Code*. Turland quoted the Rapporteurs as saying that otherwise “it might look rather odd for terms used in Art. 9 to be recommended in Rec. 9C”.

**Turland** proposed that the Recommendation be sent to the Editorial Committee to put a footnote…

**Knapp** interrupted, asking for five seconders for Turland’s proposal to refer the Recommendation to the Editorial Committee to be dealt with as a footnote.

[The **proposal** was supported by **five seconders**.]

**Rec. 9C, Prop. A** was sent to the **Editorial Committee**.

#### Recommendation 9D

**Rec. 9D, Prop. A** (32: 37: 7: 0)

**Turland** said that this proposal sought to provide an alternative to citing any available number permanently and unambiguously identifying the lectotype, neotype or epitype specimen when such a number was unavailable. It suggested that the author designating the type should, if possible, annotate the specimen or publish its photograph with a scale.

**Schori** thought the Recommendation for publishing a photograph was quite useful because if something happened to the type specimen there would be an image available. Such an image could also be used to determine whether there was a duplicate specimen in another herbarium if it showed enough detail.

**Dhabe** suggested that if an illustration were to be accepted, a photograph with a scale should also be accepted as a type specimen when no material was available.

**Gereau** thought that the Recommendation provided sound advice but felt that this was really in the province of editors of publications and not the *Code*. He did not think the *Code* should be recommending on these matters of publication.

**Rec. 9D, Prop. A** was **rejected**.

#### Article 10 and Recommendation 10A

**Art. 10, Prop. A** (16: 4: 46: 0) was **automatically** sent to the **Editorial Committee**.

**Art. 10, Prop. B** (42: 10: 13: 1)

**Turland** noted that this was a proposal to delete Art. 10.5, Clause (a), which the proposer had demonstrated in the accompanying text to be completely superfluous. As such it was an editorial matter and Turland proposed that it be sent to the Editorial Committee.

[The **proposal** was supported by **five seconders**.]

**Gereau** noted that in their “in-house” discussions of this proposal, they had read this very carefully and had decided that the clause was not redundant and should not be deleted. He could see no reason, therefore, to have it editorialized. The clause was necessary.

**Turland** asked if the proposer of Art. 10 Prop. B could give delegates a brief explanation of why the clause was redundant.

**McNeill**, the proposer, was unsure if he was allowed to do this when the Section were considering whether or not to refer the proposal to the Editorial Committee.

**Knapp** agreed that there should first be a vote on whether to send the proposal to the Editorial Committee or not.

[The proposal was **rejected**; the Section continued its discussion of Art. 10 Prop. B.]

**McNeill** explained that there were two situations to consider. In the first situation the clause was redundant because any type selected under the first sentence of Art.10.2, the situation in which a type [of a species name] was included in the protologue of a name [of a new genus or subdivision of a genus], any element eligible as type would be in the protologue and therefore would not be in conflict with it. The only situation in which a potential type could be in conflict with the protologue would be if it was “otherwise chosen”. In that case there was already a provision that such a choice could be superseded if the selection was not conspecific with any of the material, or any element, associated with the protologue. In the first case it could not be superseded because it would be in the protologue. In the second case, the case that applied when a new genus was described without any included species and the type of another species was chosen as a type, it would not be conspecific with any of the material, or any element, associated with the protologue. This could be superseded already under another part of Art. 10.2; therefore the clause seemed to him to be totally superfluous. McNeill went on to say that what Applequist had referred to earlier was that many people did not like the fact that if an element was in the protologue it could not be superseded. McNeill felt that this was a different issue. He noted that, in Sydney [1981], when this clause originally appeared covering both names of species and names of genera, it was deliberate that the word “protologue” be retained from previous versions of the provision, and not “description”.

**Art. 10, Prop. B** was **accepted**.

**Art. 10, Prop. C** (57: 5: 3: 0), **Prop. D** (57: 5: 3: 0), **Prop. E** (57: 5: 3: 0), **Prop. F** (50: 11: 4: 0), **Prop. G** (42: 5: 18: 0) **and Rec. 10A, Prop. A** (56: 4: 5: 0)

**Turland** explained that the Section would now discuss a group of proposals made by the Special Committee on Publications Using a Largely Mechanical Method of Selection of Types. He invited McNeill to state, briefly, what the problem was and how this set of proposals sought to address it.

**McNeill** explained that the problem originated in the Seattle Congress [1969], when people realized that the typifications published under the *American Code* [*of Botanical Nomenclature*, 1907] were very frequently at variance with current usage of common generic names. It could have been dealt with differently at the time, but it was decided that people should be allowed to set such names aside. The wording in the new proposals stated that although that typification was acceptable, nevertheless it could be superseded by another choice made later. McNeill went on to say that the question referred to the Special Committee in Melbourne [2011] was: “Which works were published under the *American Code*?” He noted that some were obvious; Britton and Brown’s *Illustrated Flora of the Northeastern United States and Adjacent Canada* was one work that had a large number of types. Others were more problematic. The Special Committee looked at the extent to which typification was largely mechanical and whether this process was restricted entirely to the *American Code*. They looked at the whole situation and came up with what they thought was a practical method of assessing which works should be covered. They also set timelines so that, unless a person specifically said they were using a mechanical method, the appearance of the “Type-basis Code” in 1921 would be the cut-off date. Any implicit use of the *American Code* stopped at that point. As all typifications made by the largely mechanical method only started in about 1890, there was no need for a start date.

McNeill went on to explain that the issue had been that there was now this revision in the *Code*: such names were superseded by the next selection and in many cases these were done by the lists prepared by Hitchcock and Green prior to the Cambridge Congress [1930]. In other areas, the Clements and Shear names for fungi, for example; Clements himself had been a follower of the *American Code* but of course by that time had switched. The Special Committee had tried to identify those categories of people and institutions in which, even though it was not explicitly stated in the work, it was implicit that they were following the *American Code*. The Special Committee had identified, in a way that could be practically applied, which works were involved, and there were only a relatively small number of works in which a large number of typifications occurred.

**Hawksworth** was worried about the word “affirmed” because he thought there may be cases where things had been copied by other people in the interim period. The Editorial Committee would, therefore, have to think about the word “affirmed”, otherwise people may find there were works that had been taken to be typifications now that previously would have been superseded by other ones.

**Gandhi** noted that it was for the *Melbourne Code* that the late Dr James Reveal and himself had made the proposal about the mechanical method of typification. He noted that it was Professor Reed Rollins who was mainly responsible for the introduction of the concept of mechanical method of selection in the *Code*. Reveal and Gandhi proposed that as long as the concept of the *American Code* was not mentioned in a floristic work and a typification was cited, then it should not be treated as a method of mechanical processing. Gandhi gave an example of *The Bahama Flora*, a floristic work published in 1920, in which Britton did not mention anywhere that the *American Code* was practised. Both Reveal and Gandhi argued that any typification mentioned in that work should be accepted as genuine, not as mechanical. Their proposal was sent to the Special Committee. Reveal passed away and then, within the Special Committee, John McNeill revised what they had originally proposed, and the group of proposals under discussion were the outcome.

**Knapp** asked Gandhi if he was speaking in support of the proposals.

**Gandhi** confirmed that, yes, he was on the Committee and supported the proposals.

**Thiele** proposed that delegates should vote on all of the proposals together.

**Knapp** stated that the Section would now vote on whether the proposals would be considered and voted on as a single package.

[The **proposal** was **accepted**.]

**Turland** noted that Prop. G only concerned Examples, so would automatically go to the Editorial Committee.

**Wiersema** remembered that, in the Smithsonian discussions, Soreng had raised a reference that had not been taken into account and needed to be included in the list.

**Soreng** explained that Hitchcock was proposing types in 1918 that were definitely within the timeframe of the *American Code*, but he was rejecting it at that time. In this publication he designated several types, one of which he later did not accept in 1920. Green then voted for Hitchcock’s original lectotype in 1929. Hitchcock had been explicit that he was not following the *American Code* by this time, although some of his earlier typifications were a little “iffier”. In summary, Hitchcock could not be included automatically as having followed the *American Code*.

**McNeill** thought that as Hitchcock was a US Government employee at that time, he would likely fall under it. However, if any work indicated that it was following the “Type-basis Code”, or anything else, then that work would be excluded. McNeill added that if there was no explicit evidence, it might run into difficulty. However, if in 1929 Hitchcock adopted the revised version, this would supersede the earlier version so it probably would not matter. If Hitchcock indicated that he was not following a mechanical method, it would be acceptable.

**Greuter** said that he was aware of the Hitchcock situation and specifically wanted to speak against clause (*e*) of Art. 10 Prop. F, which as formulated did not appear to be appropriate. He was opposed to penalizing the work of someone just because of where he was employed. The fact that he could not support this clause prompted him to vote against all the proposals if they were voted on in one go. In general, he was favourably impressed by the quality of the work of the Committee, but he would not be able to vote for a set of proposals that included this clause.

**Garland** stated that there did not appear to be any wording in the proposal to the effect that an author’s statement that they were not following the *American Code* (or a largely mechanical method of type selection) would be taken into account by this rule. Garland said he was thinking of Prop. F, clauses (*f*) and (*e*).

**McNeill**, speaking for the whole Committee, stated that they would be very happy to include “unless otherwise provided” or some wording of that sort, indicating that if there was a statement from an author that they were not following a largely mechanical method, the proposal would not apply. In addition, he would be happy to amend the proposal to include a provision that made sure that someone like Hitchcock, or anyone else who explicitly was not following the *American Code* in this period, was not covered by the clause. McNeill, however, was not sure why Greuter felt that clause (*e*) was unacceptable because while Britton was director of the New York Botanical Garden there was a clear ruling that the *American Code* would be followed. McNeill stated that there seemed to be no suggestion that anyone working at that Garden, or closely associated with it at that time, was not following the *American Code*, although they did not specify it. He asked Greuter if he might further explain his objection.

**Greuter** stated that his objection was to the implication, in the proposed wording, that employees of the New York Botanical Garden were slavishly following the directives of their director.

**McNeill** said he thought they were. [*Laughter*]

**Levin** commented that he was uncomfortable with the idea that they were entertaining an amendment and felt that there should be a specific proposal. He proposed that at the beginning of Art. 10.5*ter* the words “Unless the author specifically states they are not following a mechanical method of type selection, the following criteria determine…”

[*There followed some wordsmithing and issues with showing the amendments on screen*.]

**Knapp** confirmed that McNeill and the Committee **accepted** Levin’s wording as a **friendly amendment** and asked for further comment on the amended proposal.

**Gandhi** was not sure that the wording was appropriate, as he believed the term “mechanical method” was introduced only in the 1960s or ‘70s. He suggested, as a friendly amendment, replacing “following a mechanical method of type selection” with “following the *American Code*”, as it referred to what happened prior to 1935.

[The **amendment** was considered **unfriendly**.]

**Gandhi** voiced his concern over the wording, reiterating that he thought the concept of the mechanical method came into existence only in the 1960s, so he wanted to know what words could be used to apply to typification carried out before 1935.

**Thiele** called the question.

[The Section voted **to vote**.]

**Art. 10, Prop. C, Prop. D** and **Prop. E** were **accepted**.

**Art. 10, Prop. F** was **accepted as amended** and **Prop. G** was **automatically** sent to the **Editorial Committee**.

**Rec. 10A, Prop. A** was **accepted**.

**Knapp** praised delegates for finishing Art. 10 in time for lunch. She asked that delegates look at the sign on the door on their way out regarding the tea concert that would be held at 8 o’clock that evening.

[*The Section broke for lunch*.]

### Tuesday, 18^th^ July 2017, Afternoon Session

**Knapp** welcomed everyone back after lunch and reminded the Section that proposals from the floor should be delivered by hand or e-mailed to the Bureau by the end of the afternoon session on Thursday (20^th^ July), so that the Bureau would have time to look at them and think about how to order the discussion of them. Members of the Bureau, and other members of the Section, would be available for discussion and advice should anyone require help to put together a sensible proposal that would not be rejected out of hand. The chance of a proposal from the floor being accepted was extremely small; only 11% of proposals from the floor had been accepted at the Melbourne Congress [2011], so they had to be very well constructed.

**Turland** wished to reiterate that he and the Vice-rapporteur would be happy to give an informal opinion on proposals from the floor before they were submitted.

#### Article 11

**Art. 11, Prop. A** (12: 0: *54: 0)

**Turland** explained that this was a proposal for which, in the mail vote, an Editorial Committee vote had special meaning. The Rapporteurs considered the Note to be a useful clarification of how to determine the correct name for a taxon. The Note was relevant to Art. 11.4, which explained how to determine the correct name for a taxon below the rank of genus. Turland referred delegates to the wording of the *Code*, noting that when clause (*b*) applied, the existing *Code* did not specify how a correct name should be determined. The Rapporteurs noted in their comments that because the proposed Note spelled out what the Article did not cover, it would be best if it were incorporated into Art. 11.4. In other words, it needed to be an Article in itself, or part of an Article. Turland also noted that there should be a phrase added stating that, if there was no final epithet of a legitimate name available, a replacement name may be published. Turland went on to note that if the proposal was passed, the Example would go to the Editorial Committee. He supposed that in this case the Rapporteurs were proposing an amendment to the proposal.

[Because the proposers were not present, the Rapporteurs’ **amendment** was considered **unfriendly**; it received **five seconders** and was then **accepted**.]

**Greuter** wondered whether the addition of the replacement name at the end of the proposed Note deliberately left out the option of publishing the name of a new taxon.

**Turland** believed that this was also an option and it had been left out accidentally, not deliberately, so it could be added.

[The **amendment** to the **amendment** was accepted as **friendly**.]

**Gandhi** said it was not clear to him why the name of a new taxon was required, because there was no new taxon in this case.

**Turland** stated that it was not required, but it was a possibility.

**Gandhi** did not think the situation applied to a new taxon. It was about an existing taxon requiring an alternative name.

**Greuter** explained that it was obvious that these two possibilities existed if there was no other, earlier epithet available in a legitimate name. Mentioning only one would appear to discriminate against the other and this was not an impression that should be conveyed by a Note that aimed to be clarifying.

**Knapp** stated that it was no longer a Note; the amendment was to make it part of an Article.

**Greuter** agreed, noting that his comment applied equally.

**Art. 11, Prop. A** was **accepted as amended**.

**Art. 11, Prop. B** (56: 2: 5: 1), **Prop. C** (50: 1: 12: 1), **Prop. D** (50: 1: 12: 1), **Prop. E** (50: 1: 12: 1) and **Prop. F** (50: 1: 11: 2)

**Turland** noted that this set of proposals formed a group submitted by the same proposer. Prop. C to Prop. F concerned Examples and would automatically be sent to the Editorial Committee, but they were contingent on Prop. B being accepted. If Prop. B was rejected, the other proposals would be redundant.

**Wiersema** suggested that Herendeen might wish to comment on the proposal.

**Turland** agreed but wanted first to state that the Nomenclature Committee on Fossils unanimously supported all five proposals and there was a very positive mail vote on all five.

**Herendeen** stated that the proposals were necessary because they dealt with dinoflagellates, which had a dual nomenclature system, depending on whether the cyst or motile phase of the lifecycle was being dealt with. Without these changes to the *Code* there would be problems in the synonymy or selection of names for these dinoflagellates.

**Art. 11, Prop. B** was **accepted**.

**Art. 11, Prop. C, Prop. D, Prop. E** and **Prop. F** were **automatically** sent to the **Editorial Committee**.

#### Article 13

**Art. 13, Prop. A** (5: 56: 2: 3) and **Prop. B** (7: 58: 0: 1) were **rejected** based on the **mail vote**.

#### Article 14 and Recommendation 14A

**Art. 14, Prop. A** (8: 56: 1: 0) and **Prop. B** (10: 55: 1: 0) were **rejected** based on the **mail vote**.

**Art. 14, Prop. C** (57: 3: 2: 0)

**Wiersema** believed it was in Melbourne [2011] that they had added the ability to conserve infrageneric names or infraspecific names if they were the basionym of a genus or a species name. There were some of these in the *Code* and they appeared as though they were conserved, but in fact the conservation was at a higher level, genus or species, and it was not clear that the basionyms of these were also conserved. The provision had been added, and it was now clear that the names were conserved. However, it turned out that there was at least one case in App. IV where there was a replaced synonym of a name that was listed with its replacement name. It appeared as though both of these names were conserved but, in fact, the *Code* had no provision to allow that. In this situation, if the two did not have the same type, and the replaced synonym had to revert back to the type that it had before the conservation proposal, it would be problematic. To solve this and any future situations where this might be undertaken, the proposal would make it possible to conserve the basionym or replaced synonym of a genus or a species name that could not continue to be used in its current sense without conservation. The proposal solved an existing nomenclatural problem that would otherwise require a conservation proposal to fix and would allow this practice in the future.

**Art. 14, Prop. C** was **accepted**.

**Art. 14, Prop. D** (37: 4: 23: 0)

**Turland** explained that this proposal was a reformulation of the third sentence of Art. 14.9 and he proposed to refer it to the Editorial Committee.

[The **proposal** was supported by **five seconders**.]

**Art. 14, Prop. D** was sent to the **Editorial Committee**.

**Knapp** took this opportunity to clarify the procedure for sending proposals to the Editorial Committee. She explained that the issue had been discussed over lunch and she admitted to having been “very confused” about the procedure. Knapp stated that this was entirely her fault and apologized to the Section for the confusion. Knapp went on to state that sending a proposal to the Editorial Committee would work in the same way as sending a proposal to a Special-purpose Committee. She felt that it was important that the Section understood, and that people reading the Proceedings understood, what sending something to the Editorial Committee meant. Sending something to a Special-purpose Committee meant it would be considered by that Special-purpose Committee, not that they would use it, but that it was part of something that was being considered in more depth later in the time between the Congresses. Sending something to the Editorial Committee meant that the Editorial Committee could look at it and may or may not include it in the *Code*. Sending something to the Editorial Committee did not imply that it would automatically become part of the *Shenzhen Code*. Knapp warned that there was an important distinction there, and it was important that people realized that sending something to the Editorial Committee did not mean that it had been accepted: it meant that the Section had given the Editorial Committee latitude to do with it what they wanted. Knapp asked delegates if that was clear and thanked them, explaining that she just wanted to make sure that everyone was on the same page about what it meant, because she felt she had confused the matter.

**Art. 14, Prop. E** (7: 52: 5: 0), **Prop. F** (3: 55: 6: 0) and **Prop. G** (2: 57: 5: 0) were **rejected** based on the **mail vote**.

**Art. 14, Prop. H** (49: 9: 5: 0), **Prop. K** (51: 5: 5: 0) and **Rec. 14A, Prop. A** (54: 7: 3: 1)

**Turland** noted that these three proposals were linked with Art. 14 Prop. I and Prop. J and wondered whether the Section wished to talk about them all together or not. The Nomenclature Committee for Fungi supported all five but had noted that both lichenologists on the Committee for Fungi opposed Prop. J. The Council of the International Association for Lichenology, however, supported Prop. J. Turland suggested that it would be sensible to discuss Art. 14 Prop. H and K together with Rec. 14A Prop. A first, because they all concerned replacing “accepted” with “protected”. The three proposals could form a group, which could be accepted or rejected together, and the second two were contingent on the first.

**May** noted that there were another couple of proposals to discuss in the way Turland had suggested splitting them up. The terms dealt with in Art. 14 Prop. H and K and Rec. 14A Prop. A were also dealt with in Art. 56 Prop. C and F and Rec. 56A Prop. A. Looking ahead, therefore, he wished to alert delegates to the fact that these proposals would be connected back in.

**Wiersema** agreed that it was a similar concept, but he thought the terminology in the second case was different. The Art. 56 proposals dealt with rejected names, whereas the Art. 14 proposals dealt with protected names.

**Turland** explained that in the case of Art. 56 the word “rejected” was being replaced with “suppressed”, whereas in Art. 14 the word “accepted” was being replaced by “protected”. He agreed, therefore, that it was a similar concept, a change in terminology, but still thought they could be treated independently and voted on separately. However, delegates should bear in mind how they had voted on Art. 14 when discussion on Art. 56 began. Turland noted that the Art. 56 proposals were not contingent on what the Section did with Art. 14.

**Hawksworth** pointed out that the proposals had already been discussed extensively by mycologists. The terminology had been discussed with different alternatives at special meetings in the Netherlands and at the last IMC and had been reviewed by the International Commission on the Taxonomy of Fungi. A lot of thought had gone into finding words that were not competing or confusable with other words in the *Code*.

**Knapp** asked Turland whether the proposals would be voted on together or separately.

**Turland** suggested that Prop. H should be voted on first, then the next two would probably be automatic because they would be editorial. However, they could all be voted on individually.

**Knapp** ruled that, for the purposes of procedure, the Section should vote on the proposals individually.

**Art. 14, Prop. H** and **Prop. K**, and **Rec. 14A, Prop. A** were **accepted**.

**Art. 14, Prop. I** (53: 7: 4: 0)

**May** thought this was a useful clarification because they had some difficulty in the Committee for Fungi interpreting what “treated as conserved” meant. This proposal spelled out the meaning, making it clearer and easier to apply.

**Art. 14, Prop. I** was **accepted**.

**Art. 14, Prop. J** (49: 10: 4: 0)

**Turland** stated that this proposal was to remove the exception for lichenicolous fungi or lichen-forming fungi from Art. 14.13.

**Hawksworth** explained that the inclusion of the clause was due to a misunderstanding by a delegate at the last Congress that lichens were pleomorphic, and it was not actually necessary at all. If the proposal was to be rejected there would be cases where families and genera could feature on protected lists for some of the species, and some of the genera would be included but not others. Hawksworth pointed out that at least 40 genera included lichenized and non-lichenized species, so some could be protected, but not others; therefore, it seemed nonsensical to retain this clause in the article.

**Applequist** wondered why, if the clause was nonsensical, many lichenologists apparently supported it.

**Kirk** stated that it was made clear that the International Association for Lichenology supported this change and that it was an un-named delegate in Melbourne [2011] who did not quite understand the proposal that was presented there.

**Knapp** asked Kirk to explain what had not been understood.

**Kirk**, not being a lichenologist, deferred to Hawksworth.

**Hawksworth** stated that there had been concern because Art. 59 gave some precedence to sexually typified morphs. This had the potential to upset lichenology where, for example, a lichen thallus had pycnidia or no sexual production there, but lichen conidia were not actually part of the pleomorphism in the sense that it was understood generally, because these were part of the sexual cycle. The key point in Art. 59 was that the actual structure that made these was the important thing. The sterile lichen was still the structure which would make the apothecia, so it was not relevant to include this exception in the first place.

**Redhead** explained that the history of Art. 14.13 was that the proposal had come up from the floor in Melbourne and was part of the replacement package to a whole series of things concerning pleomorphic fungi. The package was distributed in Melbourne with only a few days’ notice. There was one lichenologist present who was apprehensive and did not fully understand the implications. The phrase was placed in the Article to get Art. 14.13 passed so that they could move on with creating lists of conserved and rejected names. However, the more mycologists looked at it, including the Nomenclature Committee for Fungi and a larger body of lichenologists, the more they realized that it was just introducing complications and was difficult to support. This was why the Nomenclature Committee for Fungi wished just to exclude this series of exceptions and make it homogeneous for the fungi.

**Greuter** commented that he was in favour of bringing some more logic into Art. 14.13 as proposed but, as a friendly amendment, suggested the deletion of the words “for organisms treated as fungi” and to insert “appropriate nomenclature committee” instead of “Nomenclature [Committee] for Fungi”.

[The **amendment** was considered **unfriendly** and was not supported by **five seconders**.]

**Art. 14, Prop. J** was **accepted**.

**Art. 14, Prop. L** (58: 3: 3: 0)

**Turland** stated that this proposal adjusted Art. 14.16 to clarify that it was not just the name that should be retained but the application of the name. In other words, the intent of the conservation proposal. Retention of a name as approved would be authorized subject to the decision of a later IBC, because names may be conserved to preserve a gender, orthography, spelling or type. Therefore, it was not just the name but could be other aspects of the conservation proposal. Turland thought this was not editorial because it was a little bit extra. However, it did accord with current practice and made what had been the practice, and what would still be the practice, explicit in the *Code*.

**Art. 14, Prop. L** was **accepted**.

**Art. 14, Prop. M** (55: 9: 3: 0)

**Wiersema** introduced this proposal by noting that the rules relating to conservation were retroactive, but actions taken under the rules were not. As conservation was one of those actions, he explained that there was a need to know when the conservation would take effect. Various dates could be picked, and he noted that the same concept would come into play with Art. 56 concerning rejection, and other proposals dealing with binding decisions. He noted that Art. 14.16 stated, “When a proposal for the conservation of a name has been approved by the General Committee after study by the committee for the taxonomic group concerned, retention of that name is authorized.” This seemed to be the best date to accept for when conservation would take effect. Wiersema cautioned that this would be subject to the approval of the IBC, but botanists, mycologists or phycologists would be able to create replacement names for names that would otherwise still have been available until such time as they were rejected [under Art. 14], which would have implications on replacement names. This proposal would ensure that once the conservation or rejection had taken place, one would be free to replace the name and not have to worry about such a situation. It would set a date in the case of conserved names. Wiersema pointed out that this conservation had been happening since the Vienna Rules of 1906. For the earliest *Codes*, those decisions were published in the proceedings of the Congress, and for later *Codes* the conservation dates were published in the General Committee report in *Taxon*. These dates would be the effective dates and they were spelled out for the entire history of conservation.

**Applequist** stated that the motivation of wanting to protect the legitimacy of replacement names was valid, but she had a concern about the phrasing of the proposal. The existing Article paid lip service to the decision of the later IBC. She said that delegates were going to be discussing the codification of rules for how a Nomenclature Section might reject a General Committee recommendation. Applequist explained that the *Code* stated that when a name was conserved or rejected it was added to the Appendices. If that happened on the date of effective publication of the General Committee report, since there was no provision for removing a name from the Appendices by any means, it would become completely impossible for the Nomenclature Section to overturn the decision of a General Committee, which would be undemocratic. Applequist wondered whether the proposer would accept a friendly amendment either to make the conservation retroactive to the date of the General Committee report, or to add them to the Appendices only after IBC approval.

**Turland** wished to make the point that there was nothing in the *Code* to say that when the General Committee had approved, for example a conservation proposal, it had to go in the Appendices. It was Art. 14.16 and it said retention of that name was authorized subject to the decision of a later IBC. Turland noted that some of the earlier editions of the *Code* had marked provisional entries with an asterisk, all of which were later approved by a subsequent IBC.

**McNeill** did not think Applequist was correct in saying that it was not possible to remove a name from the Appendices. It was not possible to remove a conserved name from the Appendices, but if the name was not approved by the IBC it would cease to be a conserved name. McNeill understood that it was the Congress that made the final decision. However, in the whole history of the very thick Appendices, there had been only two cases where any of those names had ever been questioned and none had ever been overthrown. McNeill felt like the botanical community were all still living in the shadow of *Acacia* and he understood the concern. However, he did not think it would be nomenclaturally disruptive if it turned out that what had been decided to have been the effective date had to be changed. It seemed to him that the sooner one could allow a name that had been approved for conservation to take effect, the better. He thought the proposal to time it on the publication of the General Committee report was an excellent one.

**Marhold** wished to make a comment on a technicality, asking if the link to the database of proposals would be stable over six years. He suggested that the link should be replaced with a more general reference.

[This was **accepted** as a **friendly amendment**.]

**Gereau** referred to the previous question from Applequist and whether this was considered a friendly amendment, as there had not been an answer.

**Knapp** explained that Applequist’s proposal had not been accepted.

**Wiersema** said he did not remember saying anything on that.

**Applequist** repeated that her amendment would be either to make the conservation take effect after IBC approval, retroactively to the date of the General Committee report, or to say that it took effect on the date of publication of the report, but the name would not be entered into the Appendices until after the Nomenclature Section approved it.

**Wiersema** stated that names would never go into the Appendices before the resolution was passed at the Congress.

**Knapp** thought that this was what McNeill had been explaining and asked him to clarify further.

**McNeill** stated that John Wiersema was the expert on the Appendices, so he would hesitate to suggest otherwise. It had only been in the very recent years, starting with Greuter who managed to get the *Code* out within a year, and successive Rapporteurs who had also succeeded in doing this. In all previous times there was a time lag between the IBC and the publication of the Appendices. This was time enough for the General Committee to have considered several conservation proposals and to have them included in the Appendices with asterisks. McNeill expected that this time, despite the fact that he thought the Rapporteur-général would have the *Code* out very rapidly given the speed of electronic publication, it would be perfectly possible that some proposals for conservation would be approved by the General Committee before the Appendices were published. McNeill went on to suggest that if the Appendices were, in fact, entirely online, should they not be continually updated once a decision had been approved by the General Committee? He felt that the suggestion by Applequist would be completely destructive in terms of rapidly ensuring that the effects of conservation could be utilized.

**Wiersema** agreed and considered **Applequist’s amendment** as **unfriendly**.

**Knapp** asked Applequist if she was prepared to make a proposal for the amendment.

**Applequist** declined.

**Barrie** pointed out why the proposal was necessary: authors of such proposals tended to act on them as soon as the General Committee had published their decisions. They did not wait for IBC approval. This proposal was, therefore, legitimizing current practice. If the Section were to set this up so that the names did not become officially conserved until they were approved by the Congress, there could be a lot of disruptive nomenclatural issues.

**Art. 14, Prop. M** was **accepted as amended**.

**Rec. 14A, Prop. A** was discussed under **Art. 14, Prop. H** and **Prop. K**.

#### Article 15

**Art. 15, Prop. A** (5: 58: 3: 0) was **rejected** based on the **mail vot**e.

#### Article 16

**Art. 16, Prop. A** (43: 7: 16: 0)

**Turland** explained that this was a proposal to replace “the name of an included genus” by “a generic name”. The proposer had demonstrated that this was superfluous wording from a previous edition of the *Code* and was editorial. The Rapporteurs agreed that it was editorial. Turland proposed that it be sent to the Editorial Committee.

**Art. 16, Prop. A** was sent to the **Editorial Committee**.

**Art. 16, Prop. B** was discussed under **Art. 6, Prop. L**.

**Art. 16, Prop. C** (57: 2: 5: 1)

**Turland** stated that this concerned the terminations in names of algae in Art. 16.3. The Nomenclature Committee for Algae supported the proposal. He asked if there was someone from the Committee who would like to comment.

**Nakada** said that he was a phycologist and as far as he knew only a limited number of phycologists used the termination of -*phycota* after the Melbourne Congress [2011]; therefore, he strongly supported the proposal.

**Art. 16, Prop. C** was **accepted**.

#### Article 18

**Art. 18, Prop. A** (30: 17: 19: 0) and **Prop. B** (24: 15: 27: 0)

**Turland** suggested that these two proposals be considered together. Prop. A was to restore Art. 18.3 to its pre-Melbourne phrasing and Prop. B added a Note. The proposer pointed out that the current wording of Art. 18.3 contained an internal contradiction: if an illegitimate generic name was conserved it was not illegitimate, hence restoring the old wording and explaining it with a Note. The proposals were editorial and could be sent to the Editorial Committee, which would also address the details noted by the Rapporteurs in their comments. Turland pointed out that Art. 19 Prop. B and Prop. C were parallel to these proposals. As the Rapporteurs’ comments were quite short he read them out: “Prop. A and B concern illegitimacy of the name of a family under Art. 18.3 and belong to a set… If accepted, the Note of Prop. B would need to be editorially adjusted ([because] Art. 18.3 applies to a name of a family, not to a genus, and if a name of a family were a later homonym it would remain illegitimate)”. Turland noted that there were some minor issues that, if passed, the Editorial Committee would take care of. He proposed that the proposals be sent to the Editorial Committee.

**Art. 18, Prop. A** and **Prop. B** were sent to the **Editorial Committee**.

#### Recommendation 18A (new)

**Rec. 18A (new), Prop. A** (45: 17: 4: 0)

**Turland** explained that this would add good advice in a new Recommendation after Art. 18.

**Gereau** felt that while the Recommendation provided good advice, it was overly prescriptive as a Recommendation. However, he would not support converting it to an Article as it would be too prescriptive there too. Therefore, he thought it should just “go away”.

[The vote on the proposal by show of hands did not clearly reach the 60% majority required and a **card vote** was requested.]

[*The card vote was followed by a tea break and discussion of the next proposal, which was interrupted by the receipt of the results of the card vote. The following discussion of the results of the card vote is added here for clarity.*]

**Knapp**, on receipt of the card vote results, asked delegates to pay attention to which one of the little cards they tore off for a card vote. She noted that there were cards with the numbers 12, 23 and 13 in the boxes.

**Monro** asked if there were any 3s [*3 being the number that was supposed to have been used*].

**Knapp** noted that there were lots of 3s, so most people had behaved beautifully. However, she repeated that delegates had to pay close attention to what card they used, because 12, 23 and 13 could no longer be used in a card vote. If this continued, delegates would limit the number of card votes there could be and when it came to something delegates thought was very important, they would not be able to have a card vote on it.

**Redhead** suggested that if there were cards in the box with the wrong number on them, it was not possible to know whether people threw in two ballots, and so the card vote should be done again. [*Groans and noes*]

**Schori** (as one of the Tellers) pointed out that it would not be significant.

**Turland** added that it was not necessary because the cards had been put into bags and numbered, and only one vote was done at a time.

**Redhead** argued that technically someone could have thrown two cards in.

**Schori** repeated her point that there were not enough [cards with the wrong numbers] to affect the vote, there were only one or two of each of the wrong numbers, so it was not enough to affect the vote in any significant way.

**Knapp** read out the results (below) confirming that the inclusion, or not, of the wrong numbered cards would not have made any difference to the result. Knapp suggested that the next time this happened, cards with the wrong numbers on them should be excluded from the vote. She asked for a show of hands and delegates agreed that this should be the case. Knapp thanked the Section, stating: “No hanging chads! Americans will all get that, but nobody else will”. Knapp thanked her Tellers who had been the fastest she had ever seen at any IBC. She asked delegates to give them a round of applause. [*Applause*]

**Rec. 18A (new), Prop. A** was **rejected** based on the **card vote** (264 yes: 271 no; 49.3% yes)

[*The sequence of events now reverts to its chronological order*.]

#### Article 19

**Art. 19, Prop. A** (61: 2: 3: 0)

**Wiersema** stated that this proposal would clarify that a subdivision of a family that included a type of the adopted legitimate name of the family, but was not formed from the generic name equivalent to that type, was incorrect but may still be validly published and could become correct later. There was a proposed Example that could be submitted to the Editorial Committee for consideration. Wiersema thought that it was back in the Berlin *Code* [1988] that there were autonyms at this level but, subsequent to that, the autonyms at these subdivisions of families no longer existed and the *Code* had glossed over what to do or what the status of those names were.

**Greuter** stated that he thought it was the Sydney Congress [1981] that introduced the new autonym concept in which the “typical subdivisions of a family” did not bear autonyms any longer. The reason for this was that these were univerbal names, or not combinations with the family names as part of them. There was no reason and no technical way to declare them autonyms. They were still sometimes considered as if they were autonyms, but they were not. They had to be treated, when published, as names of new taxa, for instance; they were not formed automatically. Greuter noted that at lower ranks, the subdivision of a genus that included the type of the name of the genus must be an autonym, and above the rank of genus this was no longer so. Below the rank of genus, when the subdivision of the genus that included the type of the adopted name of the genus could not be an autonym, or have the form of an autonym, then it would not be validly published. However, the situation for the names above the rank of genus had been glossed over in past editions of the *Code*, and this proposal provided a possible answer.

**Gandhi** asked if the incorrect name in this case would be superfluous and legitimate or just incorrect. He explained that incorrect names could also be superfluous but could continue to be legitimate, so he wondered what the situation would be according to this proposal.

**Wiersema** asked Gandhi if he was referring to Art. 52.3 and confirmed that he thought this would apply because the name was included that ought to have been adopted. However, in this case it could later be excluded and be correct.

**Art. 19, Prop. A** was **accepted**.

**Art. 19, Prop. B** (34: 15: 17: 0) and **Prop. C** (25: 13: 28: 0)

**Turland** stated that Art. 19 Prop. B and C went together and that they were parallel to the recently discussed Art. 18 Prop. A and B. They were editorial and could be sent to the Editorial Committee. The Rapporteurs had some minor concerns which could be addressed and fixed by the Editorial Committee. Turland therefore proposed that they be sent to the Editorial Committee.

[The **proposal** was supported by **five seconders**.]

**Alford** stated that he was “a little leery” about committing this to the Editorial Committee because, if this were to happen, it may or may not show up in the next *Code*, whereas if delegates voted yes or no, which he thought they should do, then at least the principle of the matter would be taken care of in the next *Code*.

**Knapp** clarified that these proposals were just rephrasing of Articles that were already in the *Code*.

**Art. 19, Prop. B** and **Prop. C** were sent to the **Editorial Committee**.

#### Article 20

**Art. 20, Prop. A** (29: 33: 3: 1)

**Turland** noted that out of all the 397 proposals, this one was the longest in terms of explanatory text for a single proposal. It explained why Art. 20.2 should never have been part of the *Code*. The proposers considered the rule unnecessary and considered the term “morphology” subject to a broad range of interpretations. They noted that deleting the Article outright was impractical, because designations that had long been considered not validly published would then become validly published names and cause untold disruption. Instead, they proposed a retroactive ending date in line with that of the requirement for a Latin description or diagnosis, 31 December 2011. This would also permit two recently published generic names of lichen and fungi, “*Caeruleum*” and “*Carbonicola*”, to be validly published. The proposers were not aware of any similar cases in botanical or mycological nomenclature since what was now Art. 20.2 first came into effect in the Montreal *Code* of 1961. Turland clarified that the problem was the term “morphology”. It was subject to a broad range of interpretations and Art. 20.2 did not really define what was meant by it. The proposers wanted to “switch off” the Article, slightly retroactively, rather than delete it.

**Schori** stated that she was not in favour of the proposal. It seemed to her that the authors wanted to change something that had been in place for quite a while just because they wanted to be able to keep two names that they had published. She did not think that it was particularly helpful to go changing the *Code* just because someone had a couple of pet projects that they wanted to protect.

**Barrie** recollected that the reason this came to the authors’ attention was because the two generic names they published were extremely obscure terms and it was controversial whether or not they were actually morphological terms. The terms were extremely specialized, and Barrie thought they were used in some small group other than botany. He explained that this was a problem that people could run into: entirely innocently coming up with a generic name and then all of a sudden it was discovered to be some obscure morphological term. Barrie therefore supported the proposal because he thought it would simplify people’s lives.

**Gereau** agreed with Barrie. He stated that the Article had been an utterly unworkable rule from its inception, not only the decision of what was morphology, but “morphology of what?” Gereau explained that he had recently been asked to give an opinion on a genus, which he would not name, where the objection was raised that it was the plural of a term currently in use for beetles. The Article did not specify plant morphology, so it could be a Latin term in morphology of anything, and the term in question was ambiguous because it was a neo-Latin form of a Greek word. Gereau asked how these sorts of names were to be judged, repeating that the Article had never been workable. He went on, robustly, to state that the proposal gave a 100-year window from January 1912 to December 2011 for this rule to be in effect, and it was “time for this turkey to die!” [*Laughter*]

**Gandhi** said that about a week earlier he had been asked for an opinion about a recently published algal genus called *Setacea*. This was a descriptive adjectival term and someone, without checking Stearn’s *Botanical Latin*, used that adjectival term as a genus name. Based on Art. 20.2, he had given his opinion that it had not been validly published.

**Levin** asked if anyone remembered what the justification was for the rule in the first place.

**McNeill** drew attention to the existing Ex. 2 and Ex. 3 under Art. 20.2, stating that they were nouns that were technical terms. In 19^th^ century literature it was sometimes difficult to know whether a person was really describing a new genus or whether in fact they were just talking about a morphological feature. He believed this to be the origin of the Article but did not know for sure. He continued by saying that the rule presumably came in at the Brussels Congress [1910] but that its time had gone, and that he was totally in agreement with Gereau.

**Wilson** expressed her concern regarding the ending date set out in the proposal because that was convenient for the authors of the proposal. She was not aware of any case in the *Code* where they had set a finishing date that was retroactive. She thought that the usual thing would be to have an ending date after the date of the IBC, not before the date of the IBC.

**Sennikov** also questioned the end date of the rule. He stated that if the rule was really so ridiculous and unworkable, although he knew of examples which were quite unambiguous and which fell under this rule, then two things should be added to the proposal. First, if the proposal passed, then the names listed in the current Examples would need to go into the list of rejected names; second, it would be reasonable to remove the rule totally and remove the finishing date, the year 2011, in order to roll it back as if the rule had never existed. If some names were an obstacle, they could be added to the list of rejected names. He proposed the extreme solution of formally deleting the Article without any limitation.

**Knapp** asked Sennikov if he was proposing an amendment.

**Sennikov** said, “Yes, apparently unfriendly.”

[The **amendment** was supported by **five seconders**.]

**Turland** wanted to say that this would be quite a bold move and it would be difficult for the Rapporteurs to say how much nomenclatural disruption might be caused by names that would become validly published again. He warned that there was a significant potential for unforeseen and unwanted consequences if the Article was deleted. Regarding Sennikov’s suggestion that the unwanted consequences, if they existed, could be dealt with by conservation or rejection, he wished to point out that this could mean a lot of work for Committees if there were a lot of cases to deal with.

**May** stated that if the Section was going to go down this track, a list of all the names that were involved would be required so that delegates could see the consequences. He proposed, therefore, just changing the end date to 2011.

**Knapp** reminded May that the Section first had to make a decision on Sennikov’s amendment and asked for further comments on deleting the Article.

**Greuter** wished to propose that in order to minimize or reduce the negative effects of deleting the Article, the current Ex. 4 and Ex. 6 of Art. 20 could be voted Examples and could be maintained, so that *Lanceolatus, Lobata, Caulis, Folium, Radix* and *Spina* would no longer have to be dealt with as validly published generic names.

[The **amendment** to Sennikov’s **amendment** was considered **friendly**.]

**Knapp** moved the discussion to making all of the Examples voted Examples to minimize disruption.

**Schori** asked how there could be voted Examples for an Article if the Article was deleted.

**Knapp** noted that the Section would be voting on deleting the Article entirely and changing all four of the Examples into voted Examples.

**Monro** pointed out that Greuter had only mentioned Ex. 4 and Ex. 6.

**Turland** stated that Schori had a point. He went on to quote the definition of a voted Example from the Glossary, “An Example denoted by an asterisk in the *Code*, accepted by an IBC in order to govern nomenclatural practice when the corresponding Article is open to divergent interpretation or does not adequately cover the matter. A voted Example is therefore comparable to a rule, as contrasted with other Examples provided by the Editorial Committee solely for illustrative purposes”. Turland noted, therefore, that in this case there would be no corresponding Article.

**Sennikov** wished to propose an amendment to the amendment that was proposed by Greuter. He suggested replacing the present Article and making a provision out of the Examples, to rule that words such as those listed in the Examples could not be validly published as generic names. This would incorporate the text of what was already in the *Code* without change.

**Turland** suggested that Sennikov wanted, therefore, to convert the Examples not into voted Examples but into an Article.

**Sennikov** explained that words listed in other Examples could be incorporated into Ex. 6 and converted into the rule in place of the present Art. 20.2.

[The **amendment** to Greuter’s **amendment** to Sennikov’s **amendment** was considered **friendly**.]

**Barrie** asked what was covered by the “etc.” in Ex. 6?

**Turland** wished to make the point that if the Section eventually accepted the “nested amendments” they could be editorially incorporated into Art. 20.4, “The following are not to be regarded as generic names”.

**Gereau** stated that all of this now very complicated reasoning ignored the fact that this had been a troublesome Article in the first place. He said it would be tremendously disruptive to get rid of it all together and then deal with all the unknown consequences. Gereau, therefore, suggested that the Section could change the end date if someone found that desirable, but to take the original proposal in the spirit in which it was offered, reject the amendment, give it a reasonable end date and get rid of it.

**Redhead** said that he was now so confused he could not possibly support all the amendments.

**Knapp** clarified what delegates would be voting on. She stated that the vote would be on whether to have an Article containing the following: “Words such as *lanceolatus, lobata, caulis, folium, radix, spina* etc., cannot be validly published as generic names”.

**Turland** added that if this was rejected, then the Section would go back to the original proposal.

**Cantrill** thought that all the amendments had been friendly, so wondered if the vote should be to delete Art. 20 and all the amendments that were there.

**Knapp** disagreed, noting that Sennikov had amended his first amendment to delete Art. 20.

**McNeill** questioned whether Art. 20 would still be deleted.

[*There followed some discussion of the procedure*.]

**Knapp** stated that a vote to approve this amendment would result in deleting Art. 20 and replacing it with something like the wording she had given previously.

**Turland** said that there would be a new proposal that would have this wording.

**Funk** called the question.

**Knapp** agreed that the discussion was tedious but was not prepared to move to a vote until everyone understood what they were voting on. If something appeared in the *Code* later, she did not want people to say it was not what they voted for.

**Gandhi** said it was still not clear to him if the Section was rejecting the name “*Lanceolatus*”, because if someone asked him to define *lanceolatus*…

**Knapp** interrupted, stating that the present discussion was not about the names, it was about the amendment to reject Art. 20.2. Knapp then called for a vote on whether to reject Art. 20.2, which was an unfriendly amendment to Art. 20 Prop. A.

[The **amendment** was **rejected**.]

**May** wished to propose an amendment to the original proposal to change the date of 2011 to “something suitable” like 2018 or 2019.

**Turland** noted that these dates were normally set as 1 January the year after the *Code* was published. He suggested that, unless the Editorial Committee “went under a bus or something”, the new *Code* should be published by the middle of 2018. He recommended, therefore, that the date should be 31 December 2018.

**Knapp** pointed out that this would be an unfriendly amendment because the proposer was not in the room.

[The **amendment** was supported by **five seconders**.]

**Saarela** did not understand why it was a problem to add a date that had already occurred, when the *Code* provisions were retroactive once they were passed. He did not see an issue with using 31 December 2011.

**Barrie** was also in favour of keeping the original date. He made the point that anyone who was familiar with Robert Lücking’s methods of working would know that he was extremely thorough in checking names. Barrie did not want to think that delegates would be punishing Lücking because he was trying to save a couple of his own names. Barrie thought that 31 December 2011 was a decent date to use and he could see no reason for changing it to 2018.

**Knapp** asked the Rapporteurs to confirm if they had said in their introductory comments that the date that had been chosen coincided with the date at which Latin was no longer required for the diagnosis.

**Turland** confirmed that he had, indeed, said this.

**Knapp** speculated that this was probably part of the reason that the date was chosen.

**Dorr** believed the date was chosen because the names in question were published in 2012 and 2013 and he thought it was merely an effort to save two names, referring to this as “silliness”.

**Turland** noted that the Section had already passed a retroactive date, 1 January 2001, when adding the requirement to use the word “*epitypus*” to Art. 9.23, so that “*epitypus*” was required to be cited in an epitypification; adding a retroactive date would not be unprecedented.

**Wiersema** confirmed that this was in Art. 9 Prop. VV, which had been dealt with earlier in the day.

**Knapp** stated that the Section was still discussing the difference in date between 31 December 2018 and 31 December 2011 and asked if there were any more questions.

**Redhead** saw nothing wrong with 31 December 2011 and thought there was a logic to it. He noted that the Section had almost got rid of the “entire thing”, so limiting it by accepting the proposal and the dates that were put forward by the proposers made perfect sense to him.

**Cantrill** called the question.

[The Section voted **to vote**.]

[The **amendment** to change the date was **rejected**.]

**Seregin** strongly opposed the proposal. He explained that we lived in the era of databasing, where words meant more than they meant some time ago because we used search engines. He wished to encourage all botanists to leave this Article as it was and not to invent new words which had ambiguous meanings.

**Thiele** pointed out that the proposal as it stood was logically inconsistent: if, after the date of 31 December 2011 the technical terms were allowed, then Ex. 6 would no longer apply and words such as *caulis*, *folium*, etc. would be applicable and perfectly valid as generic names.

**McNeill** stated that if the Section decided to delete an Article, the Editorial Committee would automatically delete any Examples associated with it, so this was not a problem.

**Knapp** interjected, noting that the discussion was not about deleting the Article but about changing it.

**McNeill** corrected himself commenting that if the Article was amended, the Editorial Committee would automatically remove any Examples that were no longer relevant.

**Thiele** accepted McNeill’s comment but wanted to point out that all the terms would become perfectly valid [as potential names of genera]. He agreed that the Article was problematic but thought that the alternative, of all those terms being allowable, was equally problematic. The community would not be dealing with obscure terms that would be rendered not validly published names, but with very common terms that would be rendered validly published names.

[The vote by show of hands apparently indicated a majority against the proposal, but nevertheless a **card vote** was requested.]

**Art. 20, Prop. A** was **rejected** based on the **card vote** (173 yes: 348 no; 33.1% yes).

[*The Section broke for afternoon tea.*]

**Knapp** welcomed delegates back noting that there was a lot of work to do and warning that she would not let anyone leave at the end of the day until it was finished!

**Art. 20, Prop. B** was deferred for discussion under **Art. 60, Prop. F, Prop. G** and **Prop. H**.

**Art. 20, Prop. C** (2: 0: 64: 0) was **automatically** sent to the **Editorial Committee**.

#### Article 21 and Recommendation 21B

**Art. 21, Prop. A** (29: 18: 20: 0), **Rec. 21B, Prop. A** (27: 22: 17: 0) and **Rec. 21B, Prop. B** (24: 22: 20: 0)

**Turland** stated that these three proposals could be considered as a set and would therefore be discussed together. Art. 32 Prop. B was linked to these proposals but could stand on its own, so would be discussed separately. The core of Art. 21 Prop. A was an editorial change: instead of saying “a plural adjective” it was saying “an adjective in the plural” and, while this was completely editorial, it also added “nominative plural” whereas the grammatical case was not specified in the existing article. The proposal also stated, “or participle used as such”. Turland believed some people would argue that a participle and an adjective were essentially the same thing in this context. In summary, he stated that these proposals were very nearly editorial and that the Rapporteurs wondered somewhat why these changes should be made.

**Gereau** proposed that all three proposals be sent to the Editorial Committee, with the recommendation that the phrase “or participle used as such” be deleted by the Editorial Committee.

[The **proposal** was supported by **five seconders**.]

**Art. 21, Prop. A, Rec. 21B, Prop. A** and **Rec. 21B, Prop. B** were sent to the **Editorial Committee**.

[*Note: Art. 21 Prop. A, Rec. 21B Prop. A and Rec. 21B Prop. B were subsequently ruled as****rejected****by the President owing to their being parallel to Art. 23 Prop. A, which was rejected by the Section – see discussion of Art. 23 Prop. A*.]

**Art. 21, Prop. B** (3: 10: 54: 0) and **Prop. C** (2: 0: 64: 0) were **automatically** sent to the **Editorial Committee**.

**Art. 21, Prop. D** (2: 0: 63: 1) and **Art. 24, Prop. C** (2: 0: 64: 0)

**Turland** noted that these two proposals were editorial and offered clearer wording of Art. 21.4 for subdivisional epithets and Art. 24.4 for specific epithets. He proposed that they be sent to the Editorial Committee.

[The **proposal** was supported by **five seconders**.]

**Art. 21, Prop. D** and **Art. 24, Prop. C** were sent to the **Editorial Committee**.

**Rec. 21B, Prop. A** and **Prop. B** were discussed under **Art. 21 Prop. A**.

#### Article 23

**Art. 23, Prop. A** (23: 27: 14: 0)

**Turland** believed that this was very similar to the proposals that had been sent to the Editorial Committee on a proposal from Gereau; therefore, he proposed it too be sent to the Editorial Committee.

[The **proposal** was supported by **five seconders**.]

**Greuter** wanted to make sure that the Rapporteur-général was confident that these were purely editorial changes. If they were sent to the Editorial Committee and turned out not to be simply editorial, he asked that they not be considered as being accepted changes to the meaning of the *Code*.

**Turland** checked the Rapporteurs’ comments for the proposal, noting that whereas the Rapporteurs could think of no adjectival or subdivisional epithets that were not nominative, they did think of *Wollemia nobilis* where the adjective could be a genitive adjective. In this case the nominative form was also *nobilis* so it would accord with the revised Art. 23.1. This was not really a concrete case of something that would be disrupted by the proposed change. Turland then suggested that the proposal was not purely editorial because it specified the nominative for the adjective, which was not currently specified. He therefore decided that it was safer to **withdraw** his **proposal** to send it to the Editorial Committee.

**Sennikov** pointed out that if the Section voted on this particular case then the previous set of proposals (Art. 21 Prop. A, Rec. 21B Prop. A and B) would have to be further discussed as they were totally parallel.

**Schori** pointed out that as most people no longer studied Latin, and fewer places offered to teach it, she thought it was helpful to provide this clarification, especially for people who were new to describing names.

**Garland**, who used to write Latin descriptions as a side business, explained that in Latin there were different cases for different usages of words in a sentence. If someone was using a word that would normally be a name, in a case other than the nominative, would that mean that they were not using a name anymore? He wondered what the status of a species name would be in a Latin description, as the only name would be in the nominative. For instance, would a name that was published in the 1800s, which was not in the nominative case, still be a name?

**Turland** believed this would be correctable because Art. 32.2 allowed an incorrect Latin termination to be corrected without change to place of valid publication and date.

**Garland** argued that in Latin it could be completely correct, it would just be used for instance in the ablative case and that would not be an incorrect termination in the sentence structure. However, it would not be considered to be a name.

**Turland** noted that Art. 32 Prop. B addressed this by proposing to put a note into Art. 32, “Improper terminations of otherwise correctly formed names or epithets may result from the use of an inflectional form other than that required by Art. 32.2.” This addressed the issue, for example, where a name appeared in the accusative or ablative in a sentence. Turland noted that this would be discussed later.

**Lindon** referred to an example of a German text that included the name *Victoria Amazonum*. As this was not in the nominative case, she asked if it would have to be corrected to the nominative or accepted as it was spelled.

**Greuter** was worried by the addition of the specification “in the nominative”. He believed that in the *Code* there was an example, *Gloeosporium balsameae*, which was an adjective in the genitive. He believed it was too restrictive to say that an adjective used in an epithet must be in the nominative. He also thought that adding “participle” as separate from “adjective” did not make much sense, as participles were grammatically adjectives.

**Turland** agreed that the addition of the word “nominative” might be restrictive. In most cases the adjectival epithets would be nominative but there may be a few cases where they were not, and it was not known how many there were. It could potentially, and unnecessarily, cause problems.

**Sennikov** had checked the example of *Gloeosporium* in the *Code*, under Art. 23 Ex. 6, and explained that it referred to the case when an adjective was treated as a noun. Therefore, it was not relevant for this particular provision: this discussion was about plant name epithets that were real adjectives, not adjectives treated as nouns.

**Art. 23, Prop. A** was **rejected**.

**Knapp** then used her Chair’s prerogative to rule that **Art. 21 Prop. A, Rec. 21B Prop. A and Rec. 21B Prop. B**, which had been sent to the Editorial Committee with similar, parallel wording, were also now to be rejected.

**Applequist** raised a point of order that while these proposals were not particularly useful, she did not think that Knapp could reject them and overrule the Section’s vote.

**Knapp** disagreed, stating that the Chair’s prerogative allowed her to go back and reject proposals if they were equivalent to any proposal that had just been discussed and rejected.

**Art. 21, Prop. A, Rec. 21B, Prop. A** and **Rec. 21B, Prop. B** were ruled as **rejected** [*see above*].

**Art. 23, Prop. B** (13: 45: 6: 0)

**Turland** explained that this was a rephrasing of Art. 23.1 adding the words “the epithet be written with an initial lower-case letter”, which would become a rule.

**Gereau** remembered the Rapporteurs’ comment that the intention was good. It would be good to have all specific epithets written in lower case. However, as written, it presented the danger of making numerous already published names not validly published. He could see nothing in the proposal to alleviate that danger, so he strongly opposed the proposal.

**Art. 23, Prop. B** was **rejected**.

**Art. 23, Prop. C** (10: 36: 18: 0) was contingent on **Art. 23, Prop. B** and was therefore **automatically rejected**.

**Art. 23, Prop. D** (51: 1: 24: 0)

**Wiersema** noted that this proposal, of which he was an author, was put together with the idea of achieving some standardization in the treatment of the terminations of transcribed Greek epithets. In forming the proposal, they restricted their survey to just Linnaean epithets based on Greek stems. Usage was mixed in the original combinations as well as in combinations where the specific epithet had been transferred to another genus. However, the majority had preserved the Greek termination, which is why they opted to make the choice they did, and then allow that to be preserved when it was published and when it was transferred to another genus. There were a set of proposals to achieve this, not only with specific epithets but also infraspecific epithets. It was written into Art. 32.2 and then carried out in the Articles dealing with specific epithets (Art. 23) and infraspecific epithets (Art. 24). In summary, there was mixed usage and this proposal was an effort to achieve standardization in usage.

**Gereau** applauded the authors for finally making explicit what had been implicit in the *Code*. It had been very difficult to justify in terms of Art. 60.1, as it was not in the checklist of things that could be changed, so implicitly one would have to leave Greek epithets Greek. This proposal made it quite explicit. It was a technical term that not many people would care about, but it was greatly in need of standardization. Gereau stated that he was tremendously in favour of it. He proposed a friendly amendment that a sentence could be added in Art. 23.5 to simply say explicitly that epithets with transcribed Greek adjectival terminations were to remain Greek when transferred to another genus. It did not quite say so in so many words, and this was such unfamiliar territory to many users that it might be useful to make an explicit sentence to that effect. Otherwise he had no comment other than “congratulations”.

**Wiersema** had no problem with Gereau’s suggestion.

**Turland** did not think it was necessary and felt it was a little overly prescriptive. He thought that the Examples made it clear and an explicit sentence would not need to be added to the rule.

**Gereau** was perfectly happy to leave it to the discretion of the Editorial Committee, so withdrew his amendment.

**Garland** was curious as to how the proposal related to Principle V in Div. I where it said, “Scientific names of taxonomic groups are treated as Latin regardless of their derivation”. It seemed to him people had treated scientific names as Latin or Greek regardless of their derivation.

**Wiersema** noted that there were several references in the *Code* to the use of transcribed Greek words, and Rec. 60G.1(a), which was enforced by Art. 60.8, prescribed using the Greek connecting vowel or the transcribed Greek connecting vowel -*o*- in certain situations when dealing with Greek words. It had been acknowledged in other places in the *Code* that transcribed Greek words were involved.

**Garland** noted that he supported the amendment, but it seemed inconsistent with the general principles.

**Gandhi** mentioned that quite a long time ago Dan Nicolson had suggested that he preferred Greek terms for generic names and Latin terms for epithets, but Gandhi admitted that such a practice was not universally acknowledged. For maintaining consistency within IPNI and for *Flora of North America* and for *Flora of India*, Gandhi had been practising the same principle that was shown on the screen. When Wiersema came up with this idea, Gandhi was willing to be a co-author.

**Art. 23, Prop. D** was **accepted**.

**Art. 32, Prop. A** (54: 1: 10: 0) [*Discussed out of sequence*.]

**Wiersema** noted that this proposal provided the basis for the changes in Art. 23. The current Art. 32.2 referred to these other Articles, but because some did not apply to names at specific rank and below, they could not just add the species part into the Article, so they split it into two parts. In other words, to make it clear which other Articles were relevant to names at particular ranks, they split the Article into two parts.

**Redhead** noted that there were French-ending “names” and he wondered if they were not correctable if they were considered to be Latinized, for example Art. 18 Ex. 10.

**Wiersema** pointed out that names published with French endings were not names at all because they were not validly published. However, this proposal was dealing with names that were defined in the *Code* as being validly published. Those not validly published would not be names in the sense of the *Code*.

**Greuter** wanted to draw the attention of the Editorial Committee to the fact that, in his opinion at least, epithets had no rank: they were “names” or “epithets in names” above the rank of species.

**Art. 32, Prop. A** was **accepted**.

**Art. 23, Prop. E** (21: 1: 56: 0) was **automatically** sent to the **Editorial Committee**.

**Art. 23, Prop. F** (13: 46: 4: 0), **Prop. G** (19: 37: 6: 1) and **Prop. H** (16: 36: 11: 0)

**Turland** noted that these three proposals formed a group. If Prop. F were to be rejected, the Note of Prop. G would be redundant. Prop. H would go straight to the Editorial Committee as an Example but would be contingent on Prop. F being accepted.

**Hawksworth** stated that it would be very unsatisfactory for mycologists to reverse the decision over the -*icola* ending that was widely used in mycology and had been corrected repeatedly.

**Art. 23, Prop. F** was **rejected** and **Prop. G** and **Prop. H** were **automatically rejected**.

**Art. 23, Prop. I** (25: 31: 8: 0)

**Turland** had made a note to the effect that if the clause “or participle used as such” was excluded from the previous proposals in Art. 21 and Art. 23, then this proposal should also be rejected. As this was the case, he suggested that this proposal should be automatically rejected.

**Art. 23, Prop. I** was **automatically rejected**.

**Art. 23, Prop. J** (52: 6: 5: 0)

**McNeill** explained that this had arisen from the fact that many works long recognized as not using Linnaean binomials, not adopting Linnaeus’s *nomina triviale*, instead used *nomina specifica legitima*. Those were the phrase names that were universal pre-Linnaeus, and which continued in use extensively after 1753. From time to time these works reduced their phrase names to two words: the generic name and one adjective. These names had originally been ruled out by a clause in the *Code* [see Art. 23.6(c) of the Berlin *Code*, 1988], but this clause had been changed at the Tokyo Congress [1993]. It had not been intended that these names should suddenly become legitimate and validly published. The current proposal would determine what works did not adopt the binomial system of Linnaean nomenclature by looking at the extent of the predominant usage in them. McNeill felt that the wording was clear as were the Examples.

**Mabberley** commented that he had spent quite a lot of time going through *The Gardeners Dictionary* […*abridged*, ed. 4. 1754]. Most of the “accidental binomials” in this work were generally not coined by Miller, but were binomials taken from pre-Linnaean works.

**Art. 23, Prop. J** was **accepted**.

**Art. 23, Prop. K** (39: 6: 18: 0)

**Turland** noted that this proposal was almost editorial if Prop. J was accepted, although he hesitated to say that something “almost editorial” be sent to the Editorial Committee. He felt that the Section should probably vote on it, but it was logical to accept it as Prop. J had just been accepted.

**Art. 23, Prop. K** was **accepted**.

#### Recommendation 23A

**Rec. 23A, Prop. A** (13: 30: 22: 0)

**Turland** explained that this Recommendation proposed a change in terminology, replacing “attributing” with “crediting”, because the proposers preferred to reserve the term “attribution” for the authorship that was treated as correct under the rules for a name. They preferred to use “crediting” when this was not the case. Turland supposed this would depend on whether the Section felt there should be such a distinction in terminology in the *Code*.

**McNeill** asked Sennikov why he adopted “crediting” rather than “ascribing”, which was what was used in Art. 46 for a situation that was not the correct attribution.

**Sennikov** explained that this was only part of a large package of several proposals and he consistently tried to make a distinction between when a certain name was attributed to somebody in the protologue and when it was attributed to somebody after the protologue. In the first case he thought it would be appropriate to say that somebody was credited with this name and then attribution would be reserved for other cases. He had tried to make a distinction in terminology concerning the modern interpretation of authorship and the interpretation of the original author.

**Knapp** explained to Sennikov that the question was why he had not used the term “ascribe”, as used in Art. 46.

**Sennikov** replied that he was not quite ready to say at that point.

**Wilson** noted that Art. 6 Prop. F had already been sent to the Editorial Committee and wondered if this proposal should also be referred.

**Turland** said that Art. 6 Prop. F concerned an Example, so was automatically sent to the Editorial Committee, and the Section did not actually discuss it. He said that whatever the Section decided on the present proposal would impact the earlier Example. If the present proposal was rejected, then that earlier Example would not go to the Editorial Committee. If the present proposal was accepted, the earlier Example would go to the Editorial Committee.

**McNeill** proposed that, as Sennikov was not yet able to answer, the best solution would be to refer the proposal to the Editorial Committee.

[The **proposal** was supported by **five seconders**.]

**Rec. 23A, Prop. A** was sent to the **Editorial Committee**.

#### Article 24

**Art. 24, Prop. A** (56: 1: 21: 0)

**Wiersema** noted that this proposal dealt with intraspecific epithets, in harmony with what the Section had already accepted for specific epithets with Greek terminations. It was a cross-reference to the provision dealing with specific epithets.

**Schori** pointed out that cross-references tended to be editorial, therefore she proposed to refer this to the Editorial Committee.

[The **proposal** was supported by **five seconders**.]

**Art. 24, Prop. A** was sent to the **Editorial Committee**.

**Art. 24, Prop. B** (55: 2: 6: 0)

**Turland** drew the Section’s attention to the positive mail vote for this proposal. Two Examples were included which, if the proposal were to be accepted, would automatically be sent to the Editorial Committee.

**Applequist** wished to propose a friendly amendment to delete the word “when”. She thought that the present wording simply declared that all such epithets purported to indicate the taxon containing the type of the species and that they were not validly published. The inclusion of “when” grammatically meant that unless the publication explicitly stated that it contained the type then it was validly published. Many of these names were published before the type method existed.

**Knapp** asked if Applequist’s amendment was to change “when purporting” to “that purport”?

**Applequist** replied in the negative, because all these epithets were treated as purporting to contain the type even though they were published before the type method existed. She said that if just the word “when” was deleted it would be okay.

**Greuter** considered the **amendment unfriendly** because there were clear cases in which use of *originalis* etc. was not purporting to indicate the taxon containing the type of the name of the next higher rank taxon.

**Turland** agreed with Greuter and remembered when they were editing the proposal that the inclusion of “when” was quite deliberate.

[The **amendment** was not supported by **five seconders**.]

**Art. 24, Prop. B** was **accepted**.

**Art. 24, Prop. C** was discussed under **Art. 21, Prop. D**.

#### Article 28

**Art. 28, Prop. A** (48: 7: 11: 0) and **Prop. B** (9: 2: 53: 1)

**Turland** pointed out that this Article sought to bring Art. 28 Note 4 into accord with the *International Code of Nomenclature for Cultivated Plants* (*ICNCP*) so that it read: “An epithet in a name published in conformity with this *Code* may be retained in a name for that taxon under the rules of the *ICNCP* when it is considered appropriate to treat the taxon concerned under that *Code*.” Turland proposed that this be sent to the Editorial Committee to check that the proposed new wording was indeed in accord with the *ICNCP*.

**Knapp** commented that in a discussion between the Royal Botanic Gardens Kew, the Natural History Museum (London) and the Royal Horticultural Society (RHS), the people at the RHS were extremely concerned about this proposal.

**Govaerts** agreed that the RHS were very concerned with the Example, which was incorrect and should not be adopted under any circumstances, but they were very happy to have that wording of the Note.

**Knapp** confirmed for the Section that it was the Example that the RHS objected to, not the Note.

**Turland** therefore proposed that Art. 28 Prop. A be sent to the Editorial Committee, which would ensure that Note 4 was indeed brought into accord with the latest edition of the *ICNCP*.

**McNeill** asked if there was any business of this proposal being in “our *Code*” at all. It seemed to him to be advising people what to do under the *ICNCP* and he did not think it should be included in the *Code*.

**Wiersema** noted that the Note had been in the *Code* for quite some time.

**Turland** agreed that the proposal was not relevant to the *Code* and asked McNeill if he was proposing to delete the Note as an amendment to the proposal.

**Knapp** asked Turland if this meant he was withdrawing his proposal to send it to the Editorial Committee.

**Turland** stated that the Editorial Committee could determine whether or not the Note was irrelevant to the *Code* and simply delete it, because it was a Note. A Note was supposed to make an implicit or explicit provision elsewhere in the *Code* more obvious. McNeill’s point was that it did not actually refer to anything else in this *Code*.

**McNeill** agreed but had less objection to the existing wording than he had to the proposed wording. The existing wording was making a statement of fact, whereas the wording in the proposal had an element of almost recommendation that some of “our epithets” should be used and it did not seem to be relevant.

**Turland** confirmed that he was withdrawing the proposal to refer this to the Editorial Committee and suggested the Section vote on the original proposal.

**Greuter** stated that he seconded the proposal by McNeill to delete the current Note 4.

**Knapp** stated that the Section would now debate whether or not to accept the proposal to delete Note 4 in Art. 28.

**Thiele** was confused because the Section were now being asked to accept an amendment which utterly reversed the proposal. He thought that the Section should just vote on the proposal.

**Knapp** agreed that this made more sense and noted that McNeill was now withdrawing his proposal to delete Note 4.

**Malécot** pointed out that the wording of Art. 28 Prop. A and B was the same as that in the current edition of the *ICNCP*.

**Knapp** thanked Malécot and invited Govaerts to speak, warning him not to discuss the Example. [*Laughter*]

**Govaerts** stated that he very much supported the proposal, because it brought the wording into conformity with the *ICNCP*. He also thought it was very important to have the Note there, because there was often great confusion on whether to use a scientific name or a cultivar name, or whether to use a scientific name for a cultivar.

**Art. 28, Prop. A** was **rejected** and **Prop. B** was **automatically** sent to the **Editorial Committee**.

#### Article 29

**Art. 29, Prop. A** (35: 28: 2: 0).

**Turland** noted that the Section had now crossed over into effective publication. Prop. A would introduce a new requirement for effective publications starting from 1 January 2019. The publication must have an ISSN (International Standard Serial Number) or an ISBN (an International Standard Book Number). Turland had heard some concern that this might be problematic in some countries, where issuing ISBNs or ISSNs was strictly controlled by the government, so that the scientists who were publishing names did not have any control over whether or not they could have an ISBN for their publication. However, the thrust of the proposal was to exclude a lot of ephemeral or so-called grey publications.

**Seregin** had a question for Turland regarding how to deal with the case of false or incomplete ISSNs or ISBNs. He had come across a provisional ISSN in a book with “empty digits” at the end of this code. Undoubtedly there was an ISSN, but it was incomplete.

**Turland** replied that if a publication had an incomplete ISSN or ISBN it might simply be due to a typographical error, but it might still have been registered correctly. If an ISSN or ISBN was demonstrably false, however, then it would not have an ISSN or ISBN and would therefore not be effectively published.

**Seregin** asked if this would be after the date of 1 January 2019.

**Turland** confirmed that it would be after the date and noted that it already applied to electronic publications in the *Code* and that it was not an entirely new concept.

**Gereau** thought that like some other proposals, this one was overly prescriptive of specific mechanisms over which the *Code* had no control. It might be desirable but was not appropriate.

**Sennikov** said that he knew of cases where publishers provided false ISBNs, but that these were not demonstrably false. Such numbers might refer to a book published a year earlier by the same publisher who just recycled the number. The authors who published with such a publisher would be penalized for something that was out of their control. He added that this practice was embraced in some countries with a very low level of control in the publishing business.

**Groom** stated that ISSN and ISBN were international standards and although the *Code* did not control them, they were a very solid base from which to start. He argued that other standards were accepted within the *Code*, so he did not have a problem about using these numbers. He believed they were good standards that were internationally controlled and could be relied upon in the future.

**Thiele** understood that the intent of this proposal was to bring consistency between electronic publication and paper publication. Currently, electronic publication required an ISSN or ISBN, but paper publication did not. Many of the objections that had been raised to ISSNs and ISBNs for paper publication also applied to electronic publication. He supported the consistency that this proposal was attempting to bring and thought if delegates objected to it, the Section would need to deal with the same issues under electronic publication unless these issues applied only to paper publication, which he was not convinced about.

**Applequist** replied that the issue of electronic publication was that when a “fly-by-night” electronic publication disappeared, all trace of it was gone, whereas books did not evaporate off the shelves. The Section had heard that for people in some parts of the world, it could be very difficult to get an ISBN, or they may think they had one when they did not. She thought that delegates from privileged parts of the world ought to take that objection seriously.

**Freire-Fierro** wondered how easy it would be in Latin American countries, for example, to obtain an ISSN or ISBN.

**Groom** said it basically came down to whether or not the Section wanted people publishing in journals that nobody could get hold of. They might be very small print runs and they may disappear off the shelves in the future. That was why this clause was added in the first place.

**Marhold** stated that people should be strongly discouraged from publishing in grey literature.

**Greuter** had two comments. The first was a technical one, that there were two dates proposed as starting dates for proposals: after 31 December 2018, which the Section had already accepted, and now on or after 1 January 2019. He urged that these dates be consistent within the *Code*. Secondly, he believed that distinct numbers were required for electronic and printed publications. He wondered what would happen if an electronic publication, for lack of attention to those rules, used “abusively” the ISSN or ISBN of the printed publication, which was already attributed to a printed book. Would these then be false ISBNs and ISSNs?

**Hawksworth** did not think delegates should underestimate the difficulty that some authors might have in obtaining an ISSN. He noted that in the previous week a senior Chinese mycologist, who already published a journal, had asked him for help to start a new journal, primarily because it would be relatively easy for Hawksworth to get an ISSN and it would be incredibly difficult in China. Hawksworth was, therefore, against the proposal.

**Kirk** thought that delegates were conflating two issues: the availability of the numbers, and the permanence of electronic publications or other ephemeral ink-on-paper publications. In defence of electronic publications, he stated that he was involved with one that went into a digital archive, which, short of World War III, would still survive. Hawksworth had alluded to a scenario where, if in countries where these numbers were difficult to obtain, it would be possible to have a partner in a country where they were easy to obtain, a bit like the rich people who have offshore bank accounts.

**Knapp** asked if Kirk had one of those.

**Kirk** refused to answer the question: “I’ll take the Fifth Amendment.” [*Laughter*]

**Art. 29, Prop. A** was **rejected**.

#### Recommendation 29A

**Rec. 29A, Prop. A** (57: 14: 1: 0)

**Turland** stated that this proposal, made by the President and himself, sought deletion of what they considered to be an unrealistic Recommendation. They considered that the *Code* should recommend realistically and that it was questionable whether libraries would curate what were essentially reprints. Authors would still be free to deposit printed copies in libraries if they wished. They felt that this was something the *Code* should not be recommending, and they proposed its deletion.

**Fortunato** noted that for electronic data, four repositories were recommended and noted, for example, that the genetic sequence data in GenBank were stored in four repositories.

**Rec. 29A, Prop. A** was **accepted**.

#### Article 30

**Art. 30, Prop. A** (10: 54: 1: 0) was **rejected** based on the **mail vote**.

**Turland** noted that a number of proposals on Art. 30 had been automatically rejected in the mail vote, including Art. 30 Prop. A and Prop. B.

**Redhead** however, wished to reintroduce Art. 30 Prop. A so that one of the issues in it could be discussed.

[The **proposal** was supported by **five seconders** and was **reintroduced** for discussion.]

**Sennikov** had written to a friend in Lund telling him that this proposal had failed in the mail vote and that now they would be in great trouble. Numerous exchange catalogues published in Lund in Sweden had on the title page a statement “Printed as manuscript” in the Swedish language. They had always considered that these manuscripts were not effectively published. These manuscripts all had authorships of scientific names and many new combinations. If the proposal was reintroduced Sennikov would be extremely happy to skip this sort of literature.

**Redhead** wished to amend the proposals because the Rapporteurs had noted that the Examples were somewhat nebulous. He wanted to change the wording of the first part [of Art. 30.1*bis*] to “Distribution of printed matter does not constitute effective publication if there is evidence within the work that…”, then replace the rest of the wording with “…the printed matter was not intended to be formally published when distributed”. He also wished to replace the Example with “Raithelhuber in 1981 distributed typeset sample pages of a proposed book, *Die Gattung Clitocybe*, that included several taxonomic novelties. The book was never published and therefore none of the proposed ‘names’ were effectively published”.

[The **amendment** was accepted as **friendly**.]

**Struwe** wished to point out that “Printed as manuscript” in Swedish did not necessarily mean that it was printed for publication in Swedish. She disagreed with the suggestion that people would have to go to Lund universities and look for every work containing “Printed as manuscript”. From a Swedish point of view, it did not necessarily mean at all that it was printed or published.

**Geltman** wished to clarify the situation with the proposed Example because it was a practice used in Russia for dissertations. A summary of the dissertation may be issued, but there was a clear indication on the title page that it had been printed “On the right of manuscript”. He thought that in such cases another provision of the *Code* could be applied to rule that such issues were not effectively published.

**Wilson** asked if Art. 30.8 already covered this situation, at least for a thesis.

**Wiersema** noted that Art. 30.8 had a date, and asked whether these cases were before 1953.

**Wilson** admitted to forgetting the date issue but noted that Redhead’s Example did not concern a thesis and would not be covered by Art. 30.8. If this proposal were to be accepted, she wondered if it should be placed around Art. 30.8.

**Turland** said he was having a hard time discerning the difference in meaning between “not intended for effective publication” and “not intended to be formally published when distributed”. He wondered what “formally published” meant and whether it was different from “effectively published”. He was worried that if this wording were to be added to the *Code* it was not necessarily defined. He asked Redhead what he meant by “formally published”.

**Redhead** explained that he had worded it that way because he did not think the printer or even the author was thinking about effectively publishing it. He was thinking more about a formal publication of a taxonomic work. In his Example, the pages were supposed to demonstrate what was in the books, and he did not think there was ever an intent, in the distribution of advertising samples, to formally publish new taxonomic entities in them: the intent was for them to appear in the book.

**Levin** felt that there was an important issue here and the Section was agonizing a bit about the exact way to express it. He hoped that delegates would vote on the proposal and, if passed, the Editorial Committee could continue the agonizing in order to figure out how to exactly express what the Section was trying to get at.

**McNeill** wished to pose a question to people who knew more than he did about distribution of pre-publication proofs and sample pages in the 19^th^ century. He seemed to remember that from time to time in advertising books, people would distribute some pages that would include names of new taxa. He believed these had been accepted and wondered if there was a view as to whether this could be disruptive for some earlier works, quite apart from the Example that the Section was talking about.

**Schori** answered that this would not just be disruptive for earlier works. There were some Swedish works that contained a synopsis of papers that were produced from a dissertation. There would be a circular citation where it would say “on page such and such of this paper, which is going to be published, this name appears”, but the combination was made there. It would affect some more recent names, in terms of the date and place of publication.

**Knapp** asked if any of her American colleagues knew about the publications of Harold St. John, all of which were produced in manuscript form and distributed individually to libraries.

**Funk** said that everybody accepted the manuscripts [as being effectively published] and had been using them for years. She noted that a lot of them were just “junk”, but people still dealt with them.

**Mabberley** pointed out that there was a striking example in the 19^th^ century that would be affected if this proposal were to be accepted: Lewis Dillwyn’s *A Review of the References to the Hortus Malabaricus of Henry van Rheede van Draakenstein* [1839], which provided lots of names for Indian plants, because Dillwyn stated that this work was for private distribution.

**Knapp** drew attention to the time and suggested that the proposal be put aside until first thing the following morning. [A show of hands demonstrated that everyone agreed.] She stated that the meeting was closed for the day, with the exception of an announcement that the Permanent Nomenclature Committee Secretaries or Chairs should see Funk on their way out. Knapp then read out instructions for delegates regarding the evening’s entertainment, which included a tea concert at the conference venue.

### Wednesday, 19^th^ July 2017, Morning Session

**Knapp** began the session with some announcements, reminding attendees to fill in comment slips or to send them by e-mail with “Comment” in the subject line to distinguish them from suggestions. She also announced that Funk and Greuter were thinking about an *ad hoc* committee to address the issues around Art. 20 Prop. A: generic names coinciding with terms used in morphology. Anyone wishing to participate should see either of those people during the lunch break.

She then began the official proceedings by reopening discussion on Art. 30 Prop. A, which had been postponed in the previous session.

#### Article 30 (continued)

**Art. 30, Prop. A** (continued)

**Redhead** informed the Section that after a sober second thought and having heard concerns at the end of the previous day, he wished to amend his emendation slightly and after “the printed matter”, add in “on or after 1 January 1953”. That change put the wording in parallel with Art. 30.4, Art. 30.6, Art. 30.7 and Art. 30.8, all of which had that date. He hoped this might alleviate some of the concerns expressed.

**Knapp** suggested that since this was an amendment to Redhead’s own amendment, it should be counted as friendly.

**Sennikov** pointed out that in the rush of the previous evening there had been some misunderstandings. When Redhead had proposed his correction, Sennikov was not aware of the Example Redhead had in mind, and it was totally against the intention of Sennikov’s proposal. The change now proposed by Redhead was a different matter and so should be treated as an **unfriendly amendment**.

**McNeill** stated that if Sennikov was now saying that the amendment was no longer friendly, Sennikov’s original proposal need not be discussed because it had received more than 75% “no” votes. Secondly, McNeill thought this was an example of a bad practice of changing the *Code* to deal with one special case. If the publication by Raithelhuber was a problem, then there was a provision for suppression of individual works. Even if the date was changed to 1953, which removed some of the works that he was concerned about, other situations could arise after that. The general principle in the *Code* was that no matter what a person said, if the work was effectively published, it was effectively published.

**Redhead withdrew** both of his **amendments** to Art. 30 Prop. A.

**Knapp** pointed out that the Example, if passed, would be dealt with by the Editorial Committee.

**Sennikov** apologized for the confusion. He added that the proposal was not about a rare case. It related to works printed by a commercial publisher with a disclaimer that they were not intended for effective publication. The works met all criteria for effective publication but, because of this disclaimer, people had taken them as not effectively published without any nomenclatural effect. This was common practice in Russia, Finland and Sweden. The phrase “Printed as manuscript”, was a translation from Swedish, but the Russian version had the same effect. If the proposal was accepted, it would fix the status quo for both nomenclatural and bibliographic purposes. However, if the proposal failed, it would bring such printed matter under consideration for nomenclatural novelties.

**Gereau** raised a point of order that the proposal had already been ruled as rejected in the mail vote and could not be discussed further without a proposal for it to be considered with corresponding seconders.

**Knapp** pointed out that Redhead had resurrected this proposal with five seconders, and once the proposal was resurrected it was on the floor again. Now that Redhead had withdrawn his amendments the original proposal was under discussion again.

**Middleton** asked Sennikov if he had applied to suppress these works and if not, why not?

**Sennikov** answered that there were hundreds of publications in Russian and some dozens in Finland and Sweden. The titles were easy to trace from libraries in Russia, but although they were less numerous in Sweden and Finland, they were harder to trace because they were scarce.

**De Lange** called the question.

[The Section voted **to vote**.]

**Art. 30, Prop. A** was **rejected**.

**Art. 30, Prop. B** (10: 54: 0: 2) and **Prop. C** (8: 56: 0: 2) were **rejected** based on the **mail vote**.

**Art. 30, Prop. D** (59: 8: 4: 1) and **Prop. I** (52: 10: 7: 1)

**Turland** moved to Art. 30 Prop. D, which sought to clearly establish that the content of an electronic publication must not be preliminary for that publication to be effective. “Content” was defined later, in Art. 30 Prop. I, so Turland suggested if the Section were to accept Prop. I, then Prop. D could be logically accepted, but if Prop. I was rejected it would not make much sense to accept Prop. D. Therefore, he suggested discussing Art. 30 Prop. I first, because it established what was meant by “content”.

[The **motion** to discuss **Prop. I** first was supported by **five seconders**.]

**Turland** introduced the proposal to convert the current Art. 30 Note 2 into an Article to define what was content and what was not, to avoid some of the uncertainty regarding “online-first” or “fast track” or “issue in progress”: electronic articles which were published ahead of an electronic issue of a journal. Although final versions, they sometimes had preliminary paginations. The final pagination was added when the issue of the journal was compiled and when the publishers wanted to arrange the articles in a particular order. Page numbers were covered by a Note in the *Code* but this proposal would make it explicit by converting the Note into an Article.

**Herendeen** thought the Article would make it clear that online early publications were effective, even ones that later came out in print. Sometimes there were months between a paper appearing online and in print.

**Kusber** thought it important to exclude linked material because it was important for editors and reviewers not to put nomenclatural acts in supplemental material which had no ID.

**McNeill** thought the Article was important and he supported it, but wondered why the word “watermark”, which appeared in the previous Note, was not repeated.

**Turland** could not remember a specific reason for removing the word, and thought it could go back in. He thought the proposers did not feel it was important.

**Malécot** wondered if there was a problem with the full and direct reference in Art. 41.5: when making a combination you must cite the basionym with a full and direct reference including page number. If the in-press version lacked a page number, but the printed or the final version online had both a volume and page number, how should a basionym be cited?

**Turland** pointed out that this was covered in the *Code* and was no different to print publications that lacked pagination. One would either cite the preliminary page number or, if it were not paginated, one could put a page number in square brackets, or say that it was not paginated.

**Malécot** responded that someone may consider later that the combination was not validly published because the full and direct reference was ambiguous and contained two page numbers, one in the in-press version and one in the final one. If the in-press version was not available in the future, there would be no possibility of checking the original.

**Turland** asked if this would be a correctable error under Art. 41.6.

**Kirk** confirmed that it was a correctable error, and that there were instances of effective publications that did not have page numbers. He pointed out that the Rapporteur-général was correct to say you could place the calculated page number in square brackets, like an editorial comment.

**Gandhi** explained that he was frequently asked whether a publication was effective if there were no volume or page numbers. This proposal solved such problems.

**Wilson** remembered something in the *Melbourne Code* regarding pagination for online publications that had no page numbers, saying that you should just count the number of pages and put this in square brackets. She could only now find Ex. 1 under Rec. 30A where a Kartesz publication had [1] for the page number.

**Lindon** said that Emma Williams had submitted a proposal for this Section to address that very issue – calculating pages in square brackets to make it absolutely clear that this was an acceptable way to cite them.

**Tong** suggested a friendly amendment to include the word “may”, as in “but it may exclude volume, issue and page numbers; it may also exclude external sources”, to expand its usefulness and cover cases where a work might have page numbers.

**Turland** said including the word “may” implied that the content in some cases did and in some cases did not exclude page numbers and this would dilute the intent of the proposal. It would then be possible to argue that page numbers were part of the content and that online-first articles with preliminary pagination were not effectively published. He did not regard this as a friendly amendment.

**Knapp** asked if there were five seconders of the amendment to insert the word “may”.

[The **amendment** was not supported by **five seconders**.]

**Groom** made an editorial comment that “hyperlink or URL” could be replaced by “URI” [Uniform Resource Identifier], as it covered both.

**Saarela** wished to make a friendly amendment to say “but it excludes volume, issue, article numbers and page numbers”, because some electronic journals only provided numbers for their articles.

[This was **accepted** as a **friendly amendment**.]

**Soreng** suggested that the content should include the DOI in order to ensure that preliminary content could always be accessed.

**Knapp** argued that a DOI was an external link and, therefore, she did not consider the amendment to be friendly.

**Turland** stated that a DOI in this case would be equivalent to volume, issue, article, and page numbers, but as it was also an external link, he would prefer not to include it. He did not consider the amendment to be friendly.

**Knapp** asked if there were five seconders for Soreng’s amendment.

[The **amendment** was not supported by **five seconders**].

**Levin** wished to know why this proposal pertained only to electronic publications, noting that similar issues regarding changes in page numbers occurred in print publications. He thought the proposal could apply more broadly.

**Turland** agreed but noted that extending the proposal to include print publications may lead to unforeseen and unwanted consequences.

**Art. 30, Prop. I** was **accepted as amended**.

**Turland** moved the discussion back to **Art. 30 Prop. D**, to establish that it was the content, as defined in Prop. I, of an electronic publication that must not be preliminary for the publication to be effective. Art. 30.2 in the *Melbourne Code* talked about preliminary versions, but this had now been narrowed down to the content being preliminary.

**Greuter** was concerned that this might throw into question the current *Dracula* Example. Although “content” was now defined to mean something in particular, the issue with the *Dracula* paper was that the format of the content was not in the definitive form. If the *Code* now just said “content” and thereby meant the substance of the article, this change would unduly widen the coverage. He suggested this could be taken care of by a friendly amendment, changing “content” to “content and layout” or “content and format”, whatever the Editorial Committee considered best.

**Turland** considered it **friendly**, although he noted that “layout” would also need to be defined.

**Wilson** argued for keeping “format” because the *Dracula* Example under Art. 30 Note 1 [Ex. 5] used the term “format” as an argument for not accepting that paper.

**Dorr** was concerned with the choice of words for “evidence within or associated with the publication”, as elsewhere only “internal evidence” was used. He wondered at the parameters of “associated with the publication”.

**Turland** explained that the wording was added at the Melbourne Congress [2011]. In this context, “associated with” meant information on the website that was serving the paper, not actually within the publication or in the PDF. For example, the table of contents or the list of articles in that issue.

**Knapp** pointed out that this was already in the *Code* and was not part of the change proposed.

**Thiele** worried about the term “format” because it was increasingly used to refer to publications in electronic format. He thought it was gaining a broader meaning than merely page layout and this might become confusing in future as the meaning solidified.

**Knapp** noted that the proposal as amended was to use the word that the Editorial Committee felt was best, and she was sure that they would investigate that: “heaven forbid that the *Code* would keep up with modern English usage.”

**Garland** commented that “content” should be replaced wherever it appeared with “content and format”.

**Turland** stated that would be editorial.

**Art. 30, Prop. D** was **accepted as amended**.

**Art. 30, Prop. E** (53: 13: 5: 1)

**Turland** introduced this as a new Note to clarify that page numbers were not part of the content of a publication and were therefore irrelevant in deciding whether a publication was preliminary or final.

**Hawksworth** suggested the Editorial Committee should check that the wording was consistent with what had just been passed. For example, the word “article” would need to be added [i.e. in “volume, issue, article, and page numbers”].

**Art. 30, Prop. E** was **accepted**.

**Art. 30, Prop. F** (3: 10: 52: 1)

**Turland** noted that this **proposal** concerned only an Example and was therefore **automatically** sent to the **Editorial Committee**.

**Redhead** asked that the Editorial Committee look into the word “watermark” in the Example because there was an earlier question about it.

**Knapp** assured him the Committee would investigate.

**Art. 30, Prop. G** (15: 1: 55: 0)

**Turland** stated that this proposal was editorial and came about because Note 1 and Ex. 5 really belonged in Art. 29 and not in Art. 30. The proposers explained that Ex. 5 did not actually illustrate Note 1. He proposed it be sent to the Editorial Committee.

[The **proposal** was supported by **five seconders**.]

**Art. 30, Prop. G** was sent to the **Editorial Committee**.

**Art. 30, Prop. H** (14: 43: 4: 2)

**Turland** explained that Prop. H was contingent on Art. 30 Prop. B and C, which were ruled as rejected in the mail vote. He suggested Prop. H should also be rejected, although the mail vote was only 68% “no”, not quite the 75% required for rejection.

**Knapp** suggested a vote because the proposal did not refer to the other proposals.

**Turland** stated that it wouldn’t make any sense to accept Prop. H because the related proposals had been rejected in the mail vote.

**Wilson** added that if one looked at the accepted Prop. I, there would be no point in looking at Prop. H.

**Knapp** and **Turland** agreed that the vote was merely a formality.

**Art. 30, Prop. H** was **rejected**.

**Art. 30, Prop. I** was discussed under **Art. 30, Prop. D**.

**Art. 30, Prop. J** (4: 39: *22: 1)

**Turland** noted that Prop. J sought to clarify that electronic supplementary material could be treated as part of the online publication to which it was linked. However, he pointed out that the first clause of Note 2 in the *Melbourne Code* suggested that supplementary material was not part of the publication. Because the Section had just amended Note 2 in Prop. I, the Section should look at that amended wording rather than Note 2 as worded in the *Melbourne Code*.

**Wiersema** explained that what had been excluded was “external sources accessed via hyperlink”.

**Turland** cited the wording of the new proposal, “Electronic supplements and appendices issued separately in Portable Document Format and linked to an online publication that complies with the provisions of Art. 29.1 are treated as part of that publication”. He pointed out that the Rapporteurs felt that changing the wording “issued separately” to “issued separately and simultaneously” might be better, as the current wording of the new Note suggested that one could link a PDF file not necessarily published at the same time to effectively published electronic material, thereby creating a supplement or appendix that was part of that publication. The Rapporteurs had suggested that those favouring amendments should vote “ed.c.”, but there had been a strongly negative mail vote. The Rapporteurs now thought the suggested amendment could be potentially disruptive and urged the Section to proceed with caution.

**Struwe** wondered about supplements and appendices that could only be published in electronic format. The publisher did not allow authors to include them in the publication. For this reason there were links, and this might include original material for the description of new species, figures of type specimens, Excel files [spreadsheets] and many types of information that might be crucial to understand the species concepts and the description of and exclusion of other species from a new species.

**Schori** agreed with the point but offered her experience in trying and failing to track down supplemental information. She described having to go back to a publisher’s website to find a link that may or may not have been active or trying to go to a database where there was supposed to be a PDF and not finding a record. She hesitated to accept this amendment, because you would have the original publication at hand, but anything linked to it was not always going to be available.

**Kirk** opined that he would tend to keep things simple. He pointed out an inconsistency between the recently passed Prop. I, which included the words “excludes external resources”, and this proposal, which was about external resources. He suggested that, if the preferred journal operated this policy, the authors should publish their new nomenclature in another journal.

**Seregin** thought the proposal covered a situation when something was in a PDF with an ISSN or ISBN, but there was a clear reference that it was an electronic supplement. Under this proposal, such a PDF may be used without any doubt about whether it was supplemental to or the main body of a publication.

**Herendeen** suggested an author could put a link in a paper to something hosted somewhere other than the publisher’s website. It could be a link to their own webpage with material that would then be considered part of the publication, and he thought this was risky.

**Greuter** said he thought initially that the Note was potentially disruptive because PDFs are already effectively published under Art. 29.1. Having reread the question, he thought the Note would better be placed under Art. 29.1, saying an electronic supplement with an ISBN or ISSN was acceptable if the ISBN or ISSN was associated with that PDF.

**Gereau** pointed out that the Note also conflicted with the redefinition of “content” already accepted by the Section. By not guaranteeing that all material was simultaneously published, the priority of the publication would be obscured, and therefore the proposal should be rejected.

**Struwe** wondered if supplements and appendices referred to the ones formally published and listed in the publication as “appendix 1”, “supplement this” etc. They could be considered a part of the formal publication, but links in the materials and methods to an external supplement somewhere should not. She suggested amending the proposal to say formally linked supplements and appendices within the publication were effectively published.

**Turland** understood that was already the intention of the proposer but wished to draw attention to conflicts with the recently passed Prop. I and the difficulties establishing the date of these publications. An electronic supplement linked to another article may not have any indication of date and could have been added to the article later. He agreed that the proposal should be rejected.

**Knapp** noted this was “very strong” for a Rapporteur-général.

**Turland** agreed it was probably the strongest thing the Rapporteur had said so far.

**Art. 30, Prop. J** was **rejected**.

**Art. 30, Prop. K** (5: 58: 0: 2) and **Prop. L** (10: 53: 1: 1) were **rejected** based on the **mail vote**.

#### Recommendation 30A

**Rec. 30A, Prop. A** (60: 7: 5: 0)

**Turland** noted that this proposal was intended to encourage the use of the phrase “Version of Record” commonly used by publishers to indicate the final version of an electronic publication. The proposal was also intended to discourage the misuse of this phrase in a preliminary version of a publication.

**Schori** added that while she was, in principle, in favour of the proposal, she noted that this was something publishers did, and the *Code* has no authority over publishers. She wondered whether it was appropriate to incorporate it into the *Code*.

**Turland** responded that it was not only publishers that did this, but also editors. He added that, for example, the Rapporteur-général was also an author, an editor, and a publisher, so it was not purely aimed at publishers who did not read the *Code*.

**Rec. 30A, Prop. A** was **accepted**.

**Rec. 30A, Prop. B** (61: 5: 4: 2)

**Turland** explained that this proposal recommended that final versions of journal articles issued online in advance of completion i.e. fast-track, online-first, pre-publication or issue-in-progress articles, should be citation-ready and should contain final pagination. The Recommendation was aimed at publishers, who should already encourage citation-ready articles with final publication details when they first appear.

**Saarela** asked if it contradicted the previous proposal that page numbers were not part of the content.

**Knapp** noted that this was only a Recommendation.

**Turland** felt that it did not contradict, but merely recommended good practice. It would always be better to have final pagination because it was much easier to cite if articles were published initially with final pagination. It also encouraged authors to publish in journals that had a policy of publishing online-first articles that were citation-ready.

**Rec. 30A, Prop. B** was **accepted**.

**Rec. 30A, Prop. C** (50: 11: 4: 1)

**Turland** explained that this proposal urged the inclusion of page numbers on the actual pages of publications in order to facilitate citation.

**Schori** added that she spent a year and a half doing freelance editing for different journals, asking authors and editors to include page numbers when the journal may have its own policies. She thought in theory it was good, but she did not think putting something in the *Code* to tell a separate body, such as the editorial board of many different journals, what to do was appropriate.

**Wilson** agreed that Schori had a point about journals, but noted that there were also books where authors had more control over what went into a freestanding monograph rather than in a journal.

**Rec. 30A, Prop. C** was **accepted**.

**Rec. 30A, Prop. D** (5: 15: 46: 0)

**Turland** noted that the proposal was essentially editorial and would make the *Code* consistent in its predominant use of the word “paper” instead of “article” in the sense of a paper or article in a journal. He proposed that it be sent to the Editorial Committee.

[The **proposal** was supported by **five seconders**.]

**Gereau** thought that because fewer publications were on paper, this would be an undesirable step backwards and he thought it should be rejected. The Editorial Committee should consider the other uses of “paper” in the *Code*, rather than simply submitting one improper suggestion to the Editorial Committee.

**Hawksworth** suggested using “taxonomic works”.

**Knapp** reminded Hawksworth that the Section was not amending but rather discussing sending the proposal to the Editorial Committee.

**Rec. 30A, Prop. D** was sent to the **Editorial Committee**.

**Rec. 30A, Prop. E** (3: 61: 1: 1) was **rejected** based on the **mail vote**.

**Rec. 30A, Prop. F** (21: 52: 2: 1)

**Turland** said that this proposal recommended that if authors published electronically, they should give preference to open-access journals. He added that the Rapporteurs had concerns that, as worded, it could imply that electronic publication was also preferred. It was debatable whether the *Code* should recommend on such matters. The Rapporteurs felt that authors may have other factors to consider, such as the cost of open-access publishing or the journal impact factor.

**Paton** noted that novelties were also mentioned in books, which were less likely to be open access than papers, so this could lead to an interpretation that people should not publish things in books and only publish journals. For this reason, he would vote against.

**Kirk** agreed, saying he had discussed copyright issues with numerous lawyers across Europe and that it was a minefield. He believed content was not copyrightable, but the layout was. Therefore, a simple process to convert the content to plain text would bypass all the restriction problems with publishing in commercial journals.

**Knapp** suggested that the Section should not enter into that discussion.

**Rec. 30A, Prop. F** was **rejected**.

#### Recommendation 31B

**Rec. 31B, Prop. A** (61: 8: 2: 1)

**Turland** explained that this proposal sought to bring Rec. 31B.1 up to date because the wording still dated from the time before electronic publication. It was worded with print-only publication in mind, and the proposed new wording covered both print and electronic publication and avoided mentioning who should indicate the date of effective publication. The proposal stressed the importance of the date being indicated in the content of the publication.

**Schori** thought the only people who had control over the effective date of publication were publishers themselves and did not think it appropriate to put something in the *Code* telling publishers what they should do.

**Knapp**, answering as a botanist (not as President), said it was important that botanists influenced publishers. Recommendations in the *Code* helped influence publishers to change their behaviour.

**Thiele** felt it was important to note that many botanists were publishers, and that in-house journals should work to this Recommendation.

**Gandhi** supported the proposal because the priority of names published was determined by the precise publication date.

**Greuter** pointed out that the Section had just accepted a clear definition of “content” for electronic publications only. Now the same term and the same meaning appeared in a Recommendation that concerned printed publications and traditionally only concerned those. He thought this was misleading as it could be interpreted that the same definition of content should be used for paper publications as for electronic publications. He wished to strike out the words “as part of the content”.

**Turland** agreed with Greuter that the Section had defined “content” for electronic publication, but not in the context of printed matter. If “as part of the content” were removed, it would weaken the Recommendation because the intention was that the date should be within the publication and not, for example, within the table of contents. Nevertheless, Turland decided that “within a publication” was enough because page numbers and volume numbers were not part of the content [of an electronic publication] and it could be argued that a publication date was comparable with page and volume numbers. In that case he would accept this as a friendly amendment. He asked if the President, as co-proposer, would also accept it as friendly?

**Knapp** concurred: as it was only a Recommendation, it could be accepted as friendly and the words “as part of the content” could be removed.

**Rec. 31B, Prop. A** was **accepted as amended**.

**Rec. 31B, Prop. B** (11: 46: 8: 1) and **Prop. C** (7: 49: 8: 2)

**Turland** noted that these were two connected proposals, which were redundant because Prop. A had just been accepted.

**Wilson** explained that the comment in both proposals about last-published parts of a multi-part publication was still useful information and suggested that this information could be incorporated in the appropriate place by the Editorial Committee.

**Turland** said that Prop. B added “publishers or editors” and Prop. C added “editors”. He thought because Rec. 31B.1 had already been changed substantially by Prop. A, the words Wilson had just mentioned were no longer part of the Recommendation. For that reason, the words “publishers or editors”, or just “editors”, should be reconsidered. Prop. A was deliberately worded to avoid mentioning authors, publishers or editors, so he thought that Prop. B and Prop. C had been implicitly rejected.

**Rec. 31B, Prop. B** and **Prop. C** were **rejected**.

**Rec. 31B, Prop. D** (7: 41: 17: 1)

**Turland** noted that Prop. D concerned the date of publication and was therefore misplaced in Rec. 30A [as originally proposed]. This proposal was also redundant because Prop. A had been accepted.

**Rec. 31B, Prop. D** was **rejected**.

**Rec. 31B, Prop. E** (46: 3: 16: 1)

**Turland** explained that this proposal suggested that precise dates (year, month and day) of effective publication should be included in electronic material. He suggested that, if accepted, it could either stand alone as a separate Recommendation or it could be editorially incorporated into Rec. 31B.1, regardless of whether the previous proposals in this Recommendation were accepted.

**Thiele** moved that it be sent to the **Editorial Committee**.

[The **motion** was supported by **five seconders**.]

**Seregin** said he was happy that the proposers of Prop. A were members of the Editorial Committee, and that Prop. A covered that situation as it clearly stated that this was the date of effective publication.

**Knapp** was concerned the Section was getting off the topic of sending this to the Editorial Committee and warned she was going to be stricter about keeping to the topic today.

**Rec. 31B, Prop. E** was sent to the **Editorial Committee**.

#### Article 32

**Art. 32, Prop. A** was discussed after **Art. 23, Prop. D**.

**Art. 32, Prop. B** (26: 9: 31: 0)

**Turland** explained that this proposal contained both a Note and an Example. If passed, the Example would go to the Editorial Committee. The Note and Example would clarify that a name or epithet may be correctable under Art. 32.2 because it had a termination that was not in accordance with the *Code* even though it was grammatically correct in its context. This was clear from the text supporting the proposal but not in the Note itself. He further explained that when a name was mentioned in a grammatical case that was not the nominative or the case that it should be for that name, such as in the example of Senecio sect. Synotii in the accusative [“*Synotios*”], the rule would allow this name to be validly published and correctable.

**Gereau** thought the Note covered an obscure point that would confuse many readers without sufficient knowledge of Latin grammar. He felt that, although the content was correct, it needed some editorial attention. A “yes” vote would give the Editorial Committee the mandate to fix the wording and make sure that it was fully understandable.

**Govaerts** stated for the record that a good example would be *Victoria amazonica*, the famous giant water lily.

**Garland** spoke in support of the proposal, and commented that the first word, “improper”, may need to be looked at because they were not improper terminations, as they had been used correctly in the original context. They were just different from the nominative form.

**Art. 32, Prop. B** was **accepted** and the Example sent to the **Editorial Committee**.

#### Article 34

**Art. 34, Prop. A** (34: 25: 5: 0)

**Turland** explained that this proposal sought to extend the effect of Art. 34.1, which concerned the *opera utique oppressa* in which names at specified ranks are ruled as not validly published. It sought to extend this to any other nomenclatural acts associated with those names, such as typifications. Turland warned that there was an issue that if the names in these works were not validly published, there could not be any nomenclatural acts associated with them. The intention was to suppress the nomenclatural acts in the suppressed work that were associated with names previously validly published. He added that the Rapporteurs had communicated with McNeill on the matter and he invited McNeill to speak about the proposal.

**McNeill** thought the intent of the proposal was good. He wondered if it could be approved with the proviso that as worded, “associated with any name of the specified ranks” was not acceptable.

**Turland** read out the final suggested wording that McNeill had offered: “and no nomenclatural act associated with any name in the specified ranks is effective”.

**Hawksworth**, the proposer, accepted the amendment as **friendly**, adding that if he had thought about it at the time, it would have been in the original proposal.

**Sennikov** asked about adding some flexibility to the proposal by allowing a separate stipulation about those nomenclatural acts. Those who proposed to suppress works may specify that only names in specific ranks were suppressed, or only other nomenclatural acts like typifications. In this way, if a work was listed as suppressed, then not all other nomenclatural acts were automatically nullified in that work.

**Knapp** asked if the amendment to change “and” to “or” was accepted by the proposer.

**Hawksworth** considered the **amendment unfriendly**.

**Redhead** pointed out that using the word “name” was not appropriate as, if they were not validly published, then they could not be a name according to the definition of that term in the *Code*.

**McNeill** explained that it referred to those names previously validly published.

**Redhead** said that “names” was used twice in the wording. He also asked if this was limited to the things within these suppressed works, or anything to do with them later. The current wording of the proposal did not seem to specify that it was the other nomenclatural acts within the specified works.

**McNeill** clarified why “no nomenclatural act associated with any name in the specified work is effective” was meaningful. Those works would not only include publications of new names, which of course were not names because the work was suppressed, but they could also include typification of previously published names, and these *were* names. For this reason, he felt the use of “name” was in order.

**Redhead** pointed out that the proposal said, “specified ranks” not “works”.

**McNeill** pointed out that works were not suppressed totally, and every suppressed work was only suppressed with respect to particular ranks. Some were for all ranks, but many were only for genera, others were only for species or infraspecific taxa. It was only for those ranks that any suppression was relevant.

**Gereau** noted that the proposal as originally written and as amended extended the scope of App. VI with unpredictable consequences. Its purpose purported to save the work of undoing the improperly-done nomenclatural acts, such as lectotypifications for non-suppressed names. However, it could just as easily cause as much or more additional work redoing the acts that were properly done. He felt it was neither predictable nor desirable.

**Schori** wondered if, as currently worded, it could be interpreted to mean that nomenclatural acts outside of the suppressed work, for names that were validly published before they were included in the suppressed works, would not be considered effective.

**McNeill** concurred that there might be a need to adjust the wording to make clear that the final clause also related only to names that appeared within the publication, but this would be entirely editorial.

**Turland** asked if something like “and no nomenclatural act within the work”, or words to that effect, would help. He asked the Recorders’ Assistant to write it down so the Editorial Committee would not forget to include those words. He added that the Editorial Committee would not likely forget such a thing.

**Knapp** agreed that the Editorial Committee was an elephant and thus unlikely to forget.

**Greuter** wanted to stress the point raised by Gereau that the proposal looked fine. In fact, he thought it looked appetizing. [*Laughter*] However, when voting on a retroactive change in an Article concerning the valid publication of names, he was wary of possible consequences. He wondered if the consequences of accepting the proposal had been assessed and found to be irrelevant or minor?

**Hawksworth** said that most of the works concerned were very old, as the Rapporteurs pointed out. The proposal mainly concerned one work where there were a huge number of inappropriate lectotypifications. He thought it would take a lot of work to propose all these separately for changes and felt a change to the rules was the easiest option for dealing with it. He had not seen any indication that this would cause any problems with the other works because they were all published pre-lectotypification.

**Art. 34, Prop. A** was **accepted as amended**.

**Art. 34, Prop. B** (3: 58: 3: 0) was **rejected** based on the **mail vote**.

**Art. 34, Prop. C** (55: 8: 3: 0)

**Wiersema** explained this proposal concerned an accompanying Article of the one just discussed, which established when this suppression would take effect. Early in the *Code* it said that the rules were retroactive unless expressly limited. This proposal made it clear that the suppression of these publications took retroactive effect.

**Thiele** sought clarification on when the decision came into effect: at the time of the decision or at the point at which it would be ratified at a later IBC? He proposed including “is authorized” and adding the word “and” before “takes retroactive effect”.

**Wiersema** accepted the **amendment** as **friendly**.

**Wilson** thought that even as amended it didn’t clearly specify what the date was for approval by the General Committee.

**Knapp** noted that the date was fixed in Art. 14 Prop. M, which was passed the previous day.

**Art. 34, Prop. C** was **accepted as amended**.

**Knapp** then suggested that the Section move on to “Article Tea” for a 30-minute break, while **Funk** asked the Nominating Committee to meet after they got their tea.

[*The Section broke for morning tea*.]

#### Article 36

**Art. 36, Prop. A** (61: 2: 3: 0)

**Turland** noted that this proposal sought a clearer formulation of Art. 36.1. It was almost editorial.

**McNeill** echoed the Rapporteur-général: the proposal did not seek to change the meaning of the Article but to make it much clearer. Art. 36.1 was difficult to apply without knowing what was meant by “not being accepted” because of the obligation in the 19^th^ century for people, particularly non-professionals or those at a lower rank, to be very cautious, polite and tentative in proposing new names, even if they accepted them. McNeill thought the first clause was the core of the Article itself. What was important was whether the author accepted the name. The remainder of the Article gave examples of ways in which it was seen that the author was not accepting the taxon. McNeill’s proposal made the first clause, (*a*), the core of the Article and the next two clauses examples of how (*a*) could apply. The next clause, the statement that a name was not validly published by mere mention of the subordinate taxa had nothing to do with whether it was being accepted by the author or not, so he thought that this was better moved to a separate paragraph.

**Art. 36, Prop. A** was **accepted**.

**Art. 36, Prop. B** (7: 40: 17: 0)

**Turland** stated that the Rapporteurs had some concerns about Prop. B. It aimed to increase the accuracy of Art. 36.1(a), which the Section had just modified, by specifying that “author” meant the author of the name, who was not necessarily the author of the publication. This would be consistent with Ex. 3 under Art. 36. The Rapporteurs thought the wording was less than ideal, because a name not validly published was not really a name in the sense of the *Code*, and the reference to Art. 46 was questionable because the *Code* already used the word “author” throughout, without referring to Art. 46.

**Greuter** added that, apart from the purely editorial cross-references, the purpose of the proposal would be achieved if in the currently accepted Prop. A “by the author” were changed into “by its author”, making it clear that it meant the author of the name and not the publication.

**Turland** asked if this could be considered an amendment to the proposal currently being discussed.

**Knapp** pointed out that the previous proposal had just been approved and it seemed to her that change would be editorial.

**Turland** agreed that it would be editorial and that the Section should continue with the current proposal, but he made a note for the Editorial Committee.

**Schori** proposed that the proposal be sent to the Editorial Committee.

[The proposal was **seconded** by **five** people.]

**Turland** pointed out that this referral would be on the understanding that if the Editorial Committee considered that no change was necessary, it would make no change.

**Art. 36, Prop. B** was sent to the **Editorial Committee**.

**Art. 36, Prop. C** (13: 27: 23: 0)

**Turland** explained that this proposal also concerned Art. 36.1(a), suggesting the words “original publication”, which remained in Art. 36.1 as amended by Prop. A, might imply the place of valid publication. The clause in question concerned designations in the sense of names not validly published. One might question whether “publication itself” unambiguously meant the publication in which the name appeared, but the Editorial Committee could ensure that this was clear if the proposal was accepted. He added that “in the original publication” was added editorially in the Edinburgh *Code* of 1966, so it went back just over half a century.

**Alford** thought there could be downstream effects of this change. He outlined an example, *Opera Varia*, a pirated publication of Linnaeus’s works, where names accepted in the original publication were not accepted in the pirated version. If the language of the Article was changed, there may be other cases where names would then not be validly published. He had made a proposal to suppress that work, but it had failed.

**Art. 36, Prop. C** was **rejected**.

**Art. 36, Prop. D** (32: 26: 6: 0) and **Glossary, Prop. A** (15: 18: 42: 0)

**Turland** explained that the Glossary proposal would be automatically sent to the Editorial Committee if Art. 36 Prop. D was accepted. The Rapporteurs had commented that the proposed addition to Art. 36 Ex. 11 seemed to be beside the point because it concerned names published before 1953 and therefore Art. 36.2 did not apply. If Prop. D was accepted, the Editorial Committee should leave that Example unchanged and find a new one to illustrate the current proposal.

Prop. D addressed an issue concerning alternative names. If two or more alternative names were published under Art. 36.2 and only one of them was accepted, none of them was validly published. The proposed addition would prevent the rule applying to this case, allowing the accepted name to be validly published, whereas the non-accepted name or names would fail to satisfy Art. 36.1(a).

**McNeill** disagreed with the Rapporteur-général that this only affected the period from 1953 onward. He thought the main role of this Article was in the reverse situation: what, in fact, were acceptable as alternative names. Although the Article began, “When, or after 1 January 1953…”, its real use was for names published before then and that therefore could be accepted as alternative names. By making it clear that the author was pointing out that they were alternatives, this would allow the rule to be more readily applied. McNeill thought the addition to the Example was a good one, because the reason these were alternative names was that Ducke indicated he was proposing one name under the *International Rules* [Brussels *Règles*, 1912] and another name for the alternative *American Code* [1907].

**Redhead** was concerned about the definition of the word “taxon” and had looked at Art. 1.1 to see what it was. He pointed out that people doing phylogenetic studies may discover that a certain species was “way off in left base” [far separated from others] and then they simultaneously publish, sometimes with the same description, an order, a family, or even a class. As there was only one species, there would be one taxon with different alternative names, even though they were at different ranks.

**McNeill** suggested to the President that Dr Redhead’s point, although interesting and important, had nothing to do with the proposal but rather the original wording of the Article.

**Geltman** gave an example from his work in dealing with the alternative names *Euphorbia* and *Tithymalus*. He strongly supported this proposal and said he could supply the Editorial Committee with other good examples.

**Gandhi** stated that in the given Example it was not clear to him why the addition was necessary, because they were alternative names published before 1953. Regardless of whether it followed the *American Code* or not, they were validly published.

**Knapp** responded that the Editorial Committee would deal with the Example.

**Sennikov** believed that the addition did not change anything in the definition, only added more, superfluous words. He wondered if “simultaneously” was necessary when it was stated that it was in the same publication? Secondly, he thought the effect of this provision would be covered if “accepted” was moved to the place of “proposed”, and “proposed” was eliminated.

**Knapp** stated that unless Sennikov was making amendments to change the proposal the Section would not be wordsmithing. She made clear that the Section would not be changing the wording of Articles and that the changes mentioned were purely editorial.

**Schori** asked about the implications of “and accepted as alternatives”. If there was a work published after 1953, in which someone proposed a new name and had a footnote saying: “If this genus is treated as something different, the combination would be…”, currently neither of those names would be validly published. If this proposal was accepted then one might say, “well, they are not accepting the one in the footnote”, which would mean the other one, which is currently considered not validly published would become validly published. This would potentially change authors and dates of a number of names.

**McNeill** felt that this was part of the intent. If an author merely said, “Those who wish to treat this differently would have to use a different name” and mentioned that name but did not accept it, then these were not alternative names. It was quite clear that the author accepted one name that he referred to. In some groups, however, there was so much controversy regarding generic delimitation that authors indicated alternative names that they did not accept. The intent of the proposal was to make clear that if an author was not accepting those alternatives, his accepted name could be validly published.

**Greuter**, on first reading the proposal, had the impression, which turned out to be erroneous, that it aimed to widen the concept of alternative names beyond what was currently in the *Code* to a concept that was usually held when people read and used this Article. The current Ex. 12 under Art. 36 was not covered by the *Code*, for the simple reason that “*Euphorbia jaroslavii*” and “*Tithymalus jaroslavii*” were not alternative names because, as defined here, alternative names were names proposed implicitly as new by the author. In the case at hand, one of the names already existed, but both were accepted alternative nomenclatures for these taxa. He finished by saying that he knew the President would contradict him now and warned that he would protest.

**Knapp** told him she was ready! [*Laughter*]

**Greuter** said this would be achieved by substituting in Art. 36.2 and Art. 36.3 the word “proposed” by “used”, which he proposed as an amendment.

[The **amendment** was accepted as **friendly**.]

**Redhead** brought up a recent series of publications by two lichenologists who agreed that they had new species but disagreed on the phylogenetic arrangement of them. They simultaneously, in a co-authored publication, published alternative names with different authorship. He wondered if the Section agreed how that would be interpreted. He had corresponded with McNeill on the topic and asked him to comment.

**McNeill** said he had concluded that as the authorship was different, the fact they were in the same paper was irrelevant. The wording said that an alternative name was accepted at the same time by the same author. The new addition emphasized that point by using the word “author” again. Under the previous wording of this Article they were validly published and, under the change, if accepted, they would also be validly published.

**Garland** admitted that he was confused by the proposed amendment, specifically the word “used”. He asked if, in a flora, one author might use two different names for the same taxon, even though that author was not the author of those names. He felt the change was unclear.

**McNeill** pointed out that this Article only applied where there was a newly published name involved. There were not many new names in floras but he gave Ex. 12 as a case in which they were not two new names: one was an existing name and only the other one was being proposed as new.

**Turland** reassured the group that the Editorial Committee would ensure that the proposal, if accepted, was made clear.

**Sennikov** did not agree with this interpretation. With the change of “proposed” to “used” it became clear that the provision also covered the cases when one author used a name already published by somebody else, or by himself, and added an alternative to that name in some later publication in which he then accepted both names. In the later publication, only one name would be new, but they were alternatives in the classification of that author. If the proposer agreed that this provision would also cover such cases that would be fine.

**Greuter** agreed that this was the intent of the friendly amendment and such cases would be covered now.

**Govaerts** accepted that this was the intent, but then both of those names would become not validly published. He wondered if the previously published name would then become not validly published.

**Turland** did not think so and said the Editorial Committee would have to ensure that “none” was expressed as something like “the newly proposed name or names”.

**Geltman** stressed that the significance of this Article was more for situations before 1953, to qualify what was alternative and what was not alternative.

**Gandhi** said that until now, for two alternative names used after 1953, if one of them already existed prior to 1953 and one was proposed after 1953, he treated the newly proposed name as not validly published. With the change in the wording, now it could become validly published and he thought it may become disruptive for past decisions.

**Greuter** clarified that the amendment he proposed was to replace “proposed” by “used” in Art. 36.2 and Art. 36.3.

**Thiele** noted that several people had said that this amendment, or Article, had more implications for the period before 1 January 1953 but he did not see that. He was confused by that comment and wanted clarification as to how the Article and its amendment affected the situation before that date.

**Geltman** answered by giving the example of *Flora USSR*, which was published in 1949. In that work, when a taxon was described in *Euphorbia*, it was also mentioned as “*Tithymalus*, nom. alt.”.

**Paton** thought the Section was spending more time talking about words not covered by the proposal itself. Although something genuinely may need to be done, he suggested that would have to be a new proposal to avoid adding to the confusion.

**Redhead** said he was still concerned about the word “used” because Art. 36 was about valid publication. The word “proposed” implied that this was the place of valid publication. When it was changed to “used” the meaning broadened and it introduced ambiguity. He proposed changing it back to “proposed”.

**McNeill** thought now that the proposal had become controversial it would be unwise to accept it. He **retracted** his acceptance of the **amendment** as **friendly** and suggested it could be discussed later with Greuter, and a separate proposal could be prepared. For the moment he thought the Section ought to stick to what was originally proposed by Mosyakin.

**Knapp** ruled that the Section would **revert** to discussing the **original proposal** without the amendment.

**Schori** asked if the proposers had any idea how many names would be affected? She knew of at least one but thought it could disrupt hundreds of names.

**Knapp** (licking her finger and making a tally mark in the air) asked Greuter to come up with a vague figure, suggesting just an order of magnitude for reference.

**McNeill** pointed out that this was not his proposal so he did not have the details. There were a number of Examples given which would clarify what should be done in a particular situation.

**Watson** called the question.

[The Section voted **to vote**.]

**Art. 36, Prop. D** was **accepted** and **Glossary, Prop. A** was **automatically** sent to the **Editorial Committee**.

**Art. 36, Prop. E** (11: 49: 3: 1) was **rejected** based on the **mail vote**.

**Sennikov** proposed that this be resurrected from the floor for discussion.

[The **proposal** was supported by **five seconders** and was **reintroduced** for discussion.]

**Turland** explained that the proposal, with seven Examples, sought to rule that names published in dictionaries, stand-alone indices, or certain kinds of reviews were not accepted by any author and were therefore not validly published. The intention was to avoid accepting names such as those published by Martinov in his *Tekhno-Botanicheskiy Slovar* of 1820. An example of this was the *Aizoaceae* and eleven other conserved family names in Appendix IIB of the *Code*. The authors claimed that they were not introducing a new provision, merely stating what was implied by the present Art. 33.1 and Art. 36.1. They also claimed that the nomenclatural disturbance would be minimal because these publications had only recently been interpreted as sources of validly published names. The Rapporteurs were not certain that there would be no unwanted consequences and were concerned that this proposal could have broader implications beyond the rather narrow case intended.

**Gandhi** thought the proposal might become disruptive, using the example of *Prodromus Florae Peninsulae Indiae Orientalis* by Wight and Arnott, published in 1834. In that work, many of the new combinations listed in the index were not included in the text but had been treated as validly published. In many works of Rafinesque, a particular name may be at the rank of subgenus or section in the text but was clearly at subgeneric rank in the index.

**Gereau** saw no possibility of an objective definition of what constituted a dictionary, a stand-alone index, or a review, and no possibility of objective application of this proposal.

**Greuter** added that of all proposals before this Section, he thought this was the most disruptive. For example, it would outlaw new combinations currently universally cited from *Index Kewensis*. The *Index Kewensis*, in its earlier editions and supplements, clearly distinguished between names: accepted names and synonyms. Therefore, these new combinations were validly published. For instance, combinations that were incorrectly attributed to Bentham and Hooker in *Genera Plantarum* were later made, for the first known time, in *Index Kewensis*.

**Mabberley** sympathized with Greuter’s view but pointed out that a number of names in *Index Kewensis* were proposed at the wrong ranks. He wondered what should be done with those because they seemed to be inadvertent changes of rank.

**Wilson** pointed out that the proposal referred only to hardcopy publications. She wondered about online databases and indexes, including the *Australian Plant Name Index* (APNI) and *Australian Plant Census* (APC), where details of publications were given. There was thus a real possibility of inadvertently making a new combination. This was also a concern in the zoological world. She did not think that all the consequences had been considered, including for electronic publications.

**Art. 36, Prop. E** was **rejected**.

#### Article 37

**Art. 37, Prop. A** (48: 10: 6: 0)

**Turland** explained that Prop. A was almost editorial, and he invited the proposer to speak.

**Nakada** stated that he had proposed this Note because the termination of an algal phylum had been changed in the *Melbourne Code*. For example, the use of -*mycota* for an algal phylum or division would be inappropriate. Based on the change made previously by the Section, he proposed an amendment in the Note of “-*phyta*” to “-*mycota*”.

**Turland** reminded the Section that a proposal had been approved the previous day to remove the special terminations of algal names at these higher ranks: -*phycota* and -*phycotina* [replacing them with -*phyta* and -*phytina*].

**Alford** thought it best to reject the proposal to avoid any confusion, asking for clarification of what algae really were. He asked if oomycetes, which were close relatives of algae, were also algae and therefore would become *Oomycota*, so even this example would be imperfect.

**Art. 37, Prop. A** was **rejected**.

**Art. 37, Prop. B** (20: 41: 3: 0)

**Turland** outlined that this proposal, which added another Note to Art. 37, claimed that Art. 37.2 applied to descriptive names as well as to automatically typified names. Therefore, one of the terminations specified in Art. 16.3 etc. could indicate the rank of a descriptive name. However, Art. 16.3 and Art. 17.1 explicitly applied only to automatically typified names and the other Articles applied to names at the rank of family or subdivision of a family, whereas descriptive names applied to taxa above the rank of family. So the proposed new Note was an incorrect statement.

**Art. 37, Prop. B** was **rejected**.

**Art. 37, Prop. C** (3: 48: 12: 0) was **rejected** based on the **mail vote**.

**Hawksworth** asked that the last sentence of this proposal go to the Editorial Committee because it caused confusion among plant pathologists. The names of special forms did not compete with names in the rank of form.

**Turland** pointed out that the proposal had to be resurrected from the floor in order to be discussed.

**Hawksworth** said he was happy for the Editorial Committee to take that on board.

**Art. 37, Prop. D** (7: 51: 6: 0) was **rejected** based on the **mail vote**.

**Art. 37, Prop. E** (19: 21: 23: 0)

**Turland** introduced the proposal by saying it made a small amendment to Art. 37.8, pointing out that the phrase “same rank-denoting term” would not cover division and phylum because these were different terms that did not denote the same rank but were “treated as referring to one and the same rank” [Art. 16 Note 1]. The Rapporteurs had questioned whether what was meant by “equivalent” was clear in the proposed new wording, asking if it could be misinterpreted with unwanted consequences. He pointed out that Art. 16 Note 1 already explicitly covered this situation and the Editorial Committee could add a reference to it.

**McNeill** proposed that it be sent to the **Editorial Committee**.

[The **proposal** was supported by **five seconders**.]

**Art. 37, Prop. E** was sent to the **Editorial Committee**.

#### Article 38

**Art. 38, Prop. A** (2: 9: 53: 0)

**Turland** stated that Prop. A sought to delete clause (*b*) of Art. 38.1. The Rapporteurs had commented that the proposal claimed that the phrase in Art. 38.1(b), “comply with the relevant provisions of Art. 32–45”, was redundant. All provisions of Art. 32–45, on valid publication of names, stipulated “In order to be validly published…” or “…is not validly published unless…”. In the *Vienna Code* [2006], this clause was at the end of Art. 32.1 and applied to all names, but when Chapter 5 of the *Code* was editorially revised for the *Melbourne Code* it was extensively rearranged and reformatted and the clause was moved to Art. 38.1, which concerned only names of new taxa.

The Rapporteurs could not trace a reason for this change in position and the proposer may well have been correct that the clause was superfluous. However, they were concerned that deleting it could bring about unwanted consequences, and the Editorial Committee might consider moving it back to Art. 32.1. Turland recommended that the Section trust the Editorial Committee to do the right thing and proposed it be sent to the Editorial Committee.

**Art. 38, Prop. A** was sent to the **Editorial Committee**.

**Art. 38, Prop. B** (50: 9: 5: 0)

**Turland** explained that this proposal sought to add a new note to Art. 38.2 to clarify the status of a description relative to a diagnosis. It highlighted that a description did not have to be diagnostic because there seemed to be some confusion about this. A validating description could not be rejected on account of it not being diagnostic.

**Applequist** spoke on behalf of the Committee for Vascular Plants saying that they generally did not investigate if there was one species potentially published in a work that had a very meagre description and was not being compared to anything else. Nobody asked whether those few words would distinguish it from any other previously published species. On the other hand, if there was a work in which multiple species were given the same one or two words of description, the Committee did not want to be forced to accept all those as validating descriptions. She thought the proposal ought to be amended in some fashion to clarify that the same validating description could not be used for multiple species in the same work.

**McNeill** reminded the Section that a proposal made to this effect in Melbourne in 2011 was defeated. The situation with Art. 38 Ex. 3, a voted Example, was that it did not reflect any provision in the *Code*. Art. 38.2 was the only part of Art. 38 in which the opinion of the author came into consideration, and Ex. 3 could only be extrapolated to other works in which names appeared in a tabular form, such as in Sweet’s *Hortus Britannicus*. If there was a tabular form in which the flower colour and the time of flowering and a few features of that sort appeared, this voted Example had the effect of a rule. In any such work, those names with similar or the same descriptors were not validly published. The rule could not be applied more generally. He reiterated that a proposal had been made at the Melbourne Congress seeking to rule that, in works in which taxa were described with the same set of characters within a single higher-ranked group, none of the names of those taxa was validly published. That proposal had not been accepted. He thought the present wording for the new Note was the best option and reinforced the fact that there was no definition of a description in the *Code*, which was why it had become a matter for Committees to make binding decisions.

**Applequist** offered as an unfriendly amendment that “a validating description need not be diagnostic as long as the identical description is not used in the same work.”

**McNeill**, as the proposer, thought such a statement might be an interesting addition, but it should be a totally separate Article and was considered an **unfriendly amendment** to the current proposal.

[The **amendment** was supported by **five seconders**.]

**Barrie** felt this could create some problems for works where the same description was used for new species in different genera, or families.

**Schori** asked whether changing “multiple taxa” to “multiple species” (or equivalent) “in the same genus or higher rank”, especially since for subspecies or varieties within a species they may all say “flowers white”, might address that concern.

**Applequist** replied that when two taxa were described as having “white flowers” and were put in different families, there was an intimation of other characters separating them.

**McNeill** suggested using the words used in Melbourne: “so long as an identical description is not used for multiple taxa under the next higher taxon”.

[This **amendment** to Applequist’s **amendment** was accepted as **friendly**].

**Sennikov** proposed a further amendment to convert the Note to an Article.

**Knapp** rejected Sennikov’s proposal and reminded members of the Section to confine discussion to the amendment as amended.

**Redhead** thought the amendment to the amendment was confusing and thought it should not be changed as it confused the whole issue.

**McNeill**, though he liked the wording that had been added, felt it was essential to defeat the amendment, because otherwise, as Sennikov had pointed out, the Note would be lost. He recommended the amendment be defeated and perhaps the content of that amendment be brought up in a separate Article as a later motion from the floor.

**Gandhi** said he had come across situations wherein multiple new species within a genus were described as “shrub three foot tall”, “shrub four foot tall”, “shrub five foot tall” and was asked for his opinion on whether they were validly published names. In these cases the descriptive part was not identical, but beyond that there was nothing else.

**Thiele** called the question.

[The Section voted **to vote**.]

[The **amendment** was **rejected** and the discussion resumed on the unamended Prop. B.]

**Gereau** said that as written, and without the amendment, the proposal clarified current practice and got rid of discussions about old descriptions that may be inadequate but were descriptions.

**Art. 38, Prop. B** was **accepted**.

**Art. 38, Prop. C** (14: 32: 19: 0) was **automatically** sent to the **Editorial Committee**.

**Art. 38, Prop. D** (60: 4: 2: 0)

**Wiersema** noted that Prop. D was related to the other proposals that added dates or indicated that nomenclatural acts would be retroactive. It was similar to the one for suppressed works, which the Section had already approved, and dealt with a binding decision on valid publication of a name.

**Art. 38, Prop. D** was **accepted**.

**Art. 38, Prop. E** (3: 59: 2: 0) was **rejected** based on the **mail vote**.

**Art. 38, Prop. F** (37: 24: 3: 0) and **Prop. G** (37: 25: 2: 0)

**Turland** noted that these two proposals were linked and they extended the application of the *descriptio generico-specifica* to the names of subdivisions of genera. The Rapporteurs had envisioned a small problem, which could be resolved editorially, in the definition of a monotypic subdivision of a genus in Prop. G. Presumably this meant that the author of the name included in the subdivision of the genus only one species, the name of which was validly published under the same generic name, even though the author may have indicated that species otherwise named were attributable to that subdivision of a genus.

**Redhead** noted there was a certain logic to having a *descriptio generico-specifica*, in that if you discovered a new species, and it was in a new genus, you had to simultaneously publish them and there was little reason to separate the description of the genus from that of the species, and you could not publish a binomial without a genus. He did not see the same parallel situation here. He felt it was opening the door to a different taxonomic rank as an unnecessary complication of the *Code*.

**Wiersema** recalled that it was precipitated by an article in *Taxon* where, because of this situation, the name *Hedyotis merguensis* was considered not validly published and a new name was required. The authors wanted to preserve the name, which was apparently in use. They found a few other examples, but he did not think there were many cases like this.

**Söderström** thought there were many cases in bryophytes where this would have an effect, so he thought that either there should be a starting date added, or it should not be approved.

**Turland** summarized by saying it seemed that the proposal was conceived in order to address a few cases and he thought that introducing new provisions in the *Code* to address a few situations or isolated cases should be avoided.

**Greuter** proposed adding a new starting date, “on and after 1 January 2019” to make it clear that no negative effects could be entailed by adoption of this proposal.

**Turland** pointed out that such a change would then apply to generic names as well.

**Wiersema** noted that the proposal would not then be able to solve the case that the author was trying to preserve as it was an older case.

[The **amendment** was considered **unfriendly**, but was supported by **five seconders**.]

**Levin** suggested that moving the amendment to before “subdivision of a genus” would make the date specific only to the case of a subdivision of a genus. He gave the Editorial Committee leave to find the best placement to limit this to subdivision of a genus.

**Greuter** accepted as a **friendly amendment** that the Editorial Committee look into the amended proposal, if passed, so that it did not affect generic names.

[The **amendment** with a starting date was **accepted.**]

**Gereau** thought the amendment removed the possible destabilizing effect but did not get at the primary problem, which was that already the *descriptio generico-specifica* was poorly understood, poorly applied, and a nightmare for editors.

**Redhead** pointed out that for fungi, names must be registered, and there were problems with people using a single descriptor for a genus and a species when they were published with a *descriptio generico-specifica*. He foresaw additional confusion because each taxon name required a separate identifier.

**Art. 38, Prop. F** was **rejected**; therefore **Prop. G** was **automatically rejected**.

**Art. 38, Prop. H** (2: 53: 9: 0), **Prop. I** (1: 52: 11: 0) and **Prop. J** (2: 53: 9: 0) were **rejected** based on the **mail vote**.

**Art. 38, Prop. K** (3: 22: 39: 1) was **automatically** sent to the **Editorial Committee**.

#### Recommendation 38B

**Rec. 38B, Prop. A** (46: 18: 1: 0) and **Prop. B** (49: 15: 2: 0)

**Turland** noted that these two proposals were linked and had received a positive mail vote. He stated that the rewording proposed in Prop. B would be especially desirable if Prop. A were adopted and accepted a suggestion from the Chair to vote on the proposals together.

**Rec. 38B, Prop. A** and **Prop. B** were **accepted**.

#### Article 40

**Art. 40, Prop. A** (8: 53: 2: 0) was **rejected** based on the **mail vote**.

**Art. 8, Prop. E** (24: 33: 6: 2) [*Deferred*]

**Turland** explained that the two linked proposals, Art. 8 Prop. E and Prop. F, had been deferred earlier. Prop. F had been rejected in the mail vote, but Prop. E could still be considered. The proposal concerned types and gatherings and would add a Note that “herbarium specimens prepared from cultivated stock derived from a wild gathering are not parts of that wild gathering.”

**Barrie** pointed out that there were two types of herbarium specimens prepared from cultivated stock from wild gatherings. The first kind was where specimens were made from the wild material and subsequent material was grown in the greenhouse and a second set of specimens was made from that. This was clearly two gatherings. However, the second situation was where people brought live, wild material into the greenhouse, grew it for a certain period and then made the specimens. He considered that this was a single gathering, because it was the same material and it was only preserved once. Although collectors sometimes gave the material a field number and also a greenhouse number, he felt this was still the same gathering because they had not made specimens from the wild material. He felt this proposed change de-legitimized the second type, which he did not want to happen.

**Wiersema** pointed out that in the second case there was no wild gathering if specimens had not been made at that time.

**Barrie** agreed but pointed out that some people regarded the wild material as separate to the material collected and preserved from the greenhouse. Having given the material a field collection number and then changing the number when making a type specimen from the greenhouse material, they often included both numbers in type citations. His view was that this was all the same single gathering.

**Wiersema** stated that the initial wild collection in such cases was not the gathering. When the specimen was made from the greenhouse, that was the gathering.

**Redhead**, from a mycological perspective, looked at this differently. Even though the discussion was about plants and cultivated stock there were implications for microorganisms, which could be gathered and cultivated so that things were generated in a laboratory, as the Section had discussed earlier. Even for plants you could produce a hybrid in the greenhouse between something and then collect a specimen from that. He felt there were nuances that were not fully appreciated in the discussion.

**Paton** stated that gatherings had to be collected at the same time to be the same. By using “gathering” as the Section had defined it, you automatically distinguish between the collection in the field and the collection made on day one in horticulture or on day two once the label had been changed. They were all different gatherings because they were collected at different times.

**Schori** proposed that this be reworded to distinguish specimens prepared from cultivated stock from any herbarium specimens that were collected in the wild.

**Govaerts** thought of orchids, where often collectors found the plant, made a specimen and collected the live plant. Later the plant flowered in cultivation and the collector took a couple of flowers and put them on the original herbarium specimen and said it was the type. He thought the Note was quite good to point out that you then have a mixed gathering [rather, a type consisting of more than one gathering] and the name would not be validly published.

**Malécot** thought the distinction between wild or cultivated material was in the way they had been propagated. Plants and fungi could be vegetatively propagated or grown from seed but then it was cultivated stock.

**Middleton** thought the Note unnecessary because “gathering” had already been defined. He thought it had more to do with how editors handled this issue when papers were submitted to journals, which was not relevant to the *Code*.

**Miller** supported Middleton’s statement.

**Wilson** disagreed because she felt it was necessary to specify what was and what was not one gathering: for example, material could be taken at several different times from a cultivated plant, but was often considered as part of one type.

**Turland** interjected at this point, saying it had become clear to the Section that the proposed Note, as currently worded, was unsatisfactory and unhelpful and would need to be completely rewritten. He suggested that this should be a proposal from the floor later in the proceedings, and that the vote now would be on the proposal as currently written. He called the question.

[The Section voted **to vote**.]

**Art. 8, Prop. E** was **rejected**.

**Knapp** suggested that those who were interested in the topic addressed by Art. 8 Prop. E should get together and think about how they could add something to help people from the various communities who used wild and cultivated gatherings in apparently quite different ways.

[*The Section broke for lunch*.]

### Wednesday, 19^th^ July 2017, Afternoon Session

**Knapp** welcomed the Section back from lunch and noted that the members should ensure they had read the General Committee reports before Friday, 21^st^ July, as that was when they would be voted upon. Any proposals from the floor for Friday would need five seconders and should either be written in copperplate handwriting and handed to her or sent by e-mail before end of play on Thursday, 20^th^ July.

#### Article 40 (continued)

**Art. 40, Prop. B** (4: 54: 6: 0) was **rejected** based on the **mail vote**.

**Art. 40, Prop. C** (21: 12: 31: 0)

**Turland** pointed out that this proposal was almost editorial and could be sent to the Editorial Committee. Acceptance of the proposal would make it explicit that Art. 40.3 should not be applied to pre-1958 names. The Rapporteurs, in their comments, had mentioned that all of the provisions in Art. 40 applied on or after 1 January 1958. The Article at the beginning [Art. 40.1] used that date and then later Articles either included a date that was later than 1958 or stated, “For the purposes of Article 40…”; therefore, they themselves apply on or after 1 January 1958. Strangely, Art. 40.3 did not. The Rapporteurs looked at the history of the Article and saw that “For the purposes of Article 40…”, was added editorially to a number of the other Articles. It seemed clear from the history that Art. 40.3 also applied on or after 1 January 1958 and it was an editorial oversight that this was not explicitly mentioned in the Article.

**Art. 40, Prop. C** was **accepted**.

**Art. 40, Prop. D** (6: 9: 52: 0) was **automatically** sent to the **Editorial Committee**.

**Art. 40, Prop. E** (7: 4: *53: 0)

**Turland** introduced this proposal, stating that it would provide in Note 2 some additional items of information that would constitute mention of a single specimen or gathering, namely “herbarium, or unique herbarium barcode or accession number”. He cautioned that the implication was that any one of these items alone would suffice, whereas herbarium alone would not. If this proposal was passed, the Editorial Committee would ensure that such an implication was avoided and noted that an “ed.c.” vote in the mail vote would be so interpreted. He proposed it go to the Editorial Committee in accordance with the majority of the mail votes.

**Art. 40, Prop. E** was sent to the **Editorial Committee**.

**Art. 40, Prop. F** (1: 31: *34: 0) was **automatically** sent to the **Editorial Committee**.

**Turland** asked any native Chinese speakers present to let the Rapporteurs know if there were any errors in the translation of the Chinese characters in the proposed Example as, apart from a few basics, the Vice-rapporteur and himself did not know any Chinese.

**Art. 40, Prop. G** (10: 48: 6: 0) was **rejected** based on the **mail vote**.

**Kirk** proposed to resurrect this proposal from the floor.

[The **proposal** was supported by **five seconders** and was **reintroduced** for discussion.]

**Kirk** then read from Principle II of the *Code*, “The application of names of taxonomic groups is determined by means of nomenclatural types.” He emphasized that the *Code* was type-based, not circumscription-based. However, there was an anomaly with respect to the publication of new generic names. He stated that he could publish a new generic name, but that genus would not require a Code-compliant type because of the unique wording in Art. 10.1, which said, “The type of a name of a genus or any subdivision of a genus is the type of a name of a species… For purposes of designation or citation of a type, the species name alone suffices, i.e. it is considered as the full equivalent of its type.” Kirk pointed out that learned colleagues had said that technically such a type need not exist, but how it could it be the full equivalent of something that did not exist? This was what prompted the proposal to change the *Code*: when a new genus is published, that new generic name should have a Code-compliant type. Kirk was open to suggestions that he was misinterpreting the *Code*.

**McNeill** said he had been rather sceptical about this initially, in conversations with the proposer, but had come to the view that this was worth putting into the *Code* because it concerned the publication of new generic names. It was not unreasonable in publishing a new generic name, that the name chosen for the type should itself be typified. He thought this was taxonomically desirable and not nomenclaturally restrictive.

**Hawksworth** also supported the proposal, saying it was not uncommon for people to assume they knew what a particular species was, indicate that it was the type of the generic name and never check the type of the species name, thus resulting in misapplied names.

**Sennikov** thought it was an overly strict interpretation of the rules and would lead to undesirable consequences. If a name had to be typified first, someone might typify it by “whatever means”, picking the first specimen that was somehow appropriate and designating it, without thinking of the consequences or how it was relevant to the protologue of the generic name. He felt this provision would lead to careless lectotypifications of old names, which could be problematic and lead to trouble in the future.

**Kirk** asked how un-typified names would have their application fixed? He pointed out that a name could not be typified on a string of characters, especially in mycology where almost all new taxa were defined molecularly. Regarding “hasty type choices”, he thought this quote from the Rapporteurs was slightly derogatory as it sounded like they did not trust the community to carry out good taxonomy. Conservation was there to fix any errors. He concluded by saying that the fundamental thing was that new names at the rank of genus should not be published without having *Code*-compliant types, otherwise those names could not be applied.

**Greuter** opposed the proposal because it could lead to an inordinate number of non-validly published new generic names that would not conform to this new provision. He thought the intent behind the proposal was excellent but that it was better phrased as a Recommendation rather than as a rule.

**Redhead** said he was sympathetic to the proposal but agreed with Greuter’s suggestion that it should be a Recommendation. Otherwise it would slow to a snail’s pace modern revisions of genera, where some of the species that were now shown to belong in totally different places were ancient names that no-one had ever gone back and typified and were only known from concepts. Some of the names went back to Fries, Persoon and several other authors, and locating the exact types would be exceedingly difficult.

**Hawksworth** proposed a friendly amendment to insert “of fungi” after “genus” because he thought it was important for mycology particularly. [The amendment was **accepted** as **friendly.**]

**Turland** added that the new Article would require citation of the type of the species name for the generic name to be validly published. The Rapporteurs asked what would happen if a type was cited but this turned out to be an error and something other than the type was cited, perhaps inadvertently. Would that name be validly published?

**Kirk** [the proposer] shook his head, no.

**Thiele** spoke to the amendment, saying that it was an excellent idea to have Articles that applied only to fungi because they were fungi. However, he thought in this case there was nothing special about fungi that required restriction of the Article.

**Redhead** agreed that as a mycologist he did not particularly like fungi being singled out in this case.

**Kirk** accepted as **friendly** Greuter’s amendment that the proposal would be better phrased as a Recommendation rather than a rule.

**Barrie** suggested that if the proposal was now for a Recommendation, both “of fungi” and the starting date should be deleted.

**Kirk** also **accepted** this amendment as **friendly**.

**Knapp** clarified what all the amendments to the proposal were: that this would be a Recommendation to follow Art. 40 that would apply to all groups covered by the *Code* and would not have a starting date.

**Turland** commented that the Rapporteurs’ concerns were now no longer concerns.

**Art. 40, Prop. G** was **accepted as amended**.

**Art. 40, Prop. H** (62: 1: 2: 0)

**Turland** explained that Prop. H would make it clear that a type culture of an algal or fungal name could not be the nomenclatural type unless its metabolically inactive state was specified in the protologue, thereby avoiding uncertainty as to the validity of some algal or fungal names for which the type citation included a culture in addition to a normally preserved type specimen.

**Nakada** suggested that, although probably an editorial matter, he thought this should be associated with the deletion of Rec. 8B.3.

**McNeill** agreed both that deletion of the Rec. 8B.3 would be necessary and that it was editorial.

**Art. 40, Prop. H** was **accepted**.

#### Recommendation 40A

**Rec. 40A, Prop. A** (20: 39: 7: 0) was **withdrawn**.

**Knapp** noted that this was the first proposal to be withdrawn and thanked Kirk, saying that it was always nice to be the first to do something. [*Laughter*]

**Rec. 40A, Prop. B** (43: 17: 5: 0)

**Thiele** thought this proposal was more generic – “forgive the pun” – than the one just accepted as a Recommendation. This covered families, subdivisions of families, genera and subdivisions of genera and thus might supersede the proposal just accepted.

**Knapp** suggested that, if passed, the Editorial Committee would sort out the redundancy.

**Turland** agreed that the Editorial Committee could either merge them into one Recommendation or have two separate Recommendations for the different ranks.

**Applequist** thought “species name on which the name of a genus is based” did not really make a lot of sense.

**Rec. 40A, Prop. B** was **accepted**.

**Rec. 40A, Prop. C** (33: 3: 30: 0)

**Turland** said the proposal would change the sole occurrences of “Roman script” and “Roman letters” in the *Code* to “Latin script” in both cases, because “Roman” could be interpreted as either Latin or as non-italic, upright typeface. He proposed that this be sent to the Editorial Committee.

[The **proposal** was supported by **five seconders**.]

**Gereau** urged the Editorial Committee to use “in the Latin alphabet”, which would be clearer than “Latin script”, as “alphabet” was more familiar to many readers than “script”.

**Rec. 40A, Prop. C** was sent to the **Editorial Committee**.

**Rec. 40A, Prop. D** (42: 30: 4: 1)

**Turland** noted that this proposal addressed a weakness in Art. 40.7, which required the herbarium where the holotype was conserved to be specified in the protologue. However, it was not easy to confirm that the holotype was actually deposited in that herbarium. While the Rapporteurs wished this to be the case, it would be a difficult rule to apply. Rec. 40A.3 already helped by recommending the citation of any number that permanently identified the specimen, and this proposal would be added to that Recommendation.

**Barrie** questioned what “actually deposited” meant. Did it mean after the specimen had been sent to the herbarium or after it was formally accessioned? A specimen may be sent to a herbarium but not formally accessioned for years due to backlogs. This proposal would put the herbarium and the authors in a difficult situation.

**Miller** added that specimens moved around for all sorts of reasons and such a requirement was unenforceable. A specimen deposited one day may be gone the next week.

**Middleton** pointed out that this was a Recommendation not a rule, therefore enforceability was not an issue.

**Freire-Fierro** agreed with the Recommendation, as it would force the herbarium to mount the specimen and have it available.

**Paton** suggested the removal of “and until”, given that this was a Recommendation, to remove some of the concerns while at the same time ensuring that the thought was in the *Code*.

[The proposer was not present, so Paton’s amendment was considered **unfriendly**; the **amendment** was supported by **five seconders** and was **accepted**.]

**Alford** did not support the proposal because he felt it was speaking down to authors, as if they could not be trusted to be honest.

**Dhabe** pointed out that while it was essential for the author to deposit the specimen, the author might not want to deposit it until the name was published. Good practice may be to deposit the specimen in a herbarium along with the published paper.

**Rec. 40A, Prop. D** was **rejected**.

**Rec. 40A, Prop. E** (6: 7: *65: 0)

**Turland** explained that this would add a new Recommendation, “Citation of the herbarium or collection or institution of deposition should be in full, with the location, when no abbreviated form is given by one of the standards mentioned in Art. 40 Note 4”; for example, an *Index Herbariorum* herbarium code. The Rapporteurs suggested that rather than forming a new Recommendation, this could be editorially combined with Rec. 40A.4 and that an “ed.c.” vote in the mail vote would be so interpreted.

**Groom** felt it was encouraging people not to put their specimens in a well-established herbarium and gave them a way out.

**Gereau** thought entirely the contrary. He said there were many situations in which one was required to deposit specimens in a project herbarium. For example, if the foundation that sponsored one’s research had its own unregistered herbarium, one of the conditions might be that duplicates had to be deposited there. If this was an obligation, there should be a means of referring to that herbarium.

**Rec. 40A, Prop. E was accepted**.

**Rec. 40A, Prop. F** (34: 32: 9: 0)

**Turland** summarized this proposal as providing an alternative to citing any available number permanently identifying the holotype specimen when such a number was not available. The Rapporteurs noted that Rec. 9D Prop. A was parallel, and that proposal had been rejected by the Section. Logically the Section should reject this too, otherwise it would be inconsistent.

**Knapp** pointed out [with some sarcasm] that such a thing had never happened in the *Code*.

**Barkworth** proposed amending “in the absence of a number” by replacing it with “a unique identifier”.

[The **amendment** was supported by **five seconders**.]

**Barrie** thought “unique identifier” should be moved so that it said, “in [the] absence of a permanently unique identifier”, and delete “identifying”.

**Groom** did not think that a number was a unique identifier because a number was not unique.

**Saarela** noted that this could cause problems because there was no guarantee that any unique identifier from a herbarium would be globally unique.

**Barrie** pointed out that what was written was not what he had suggested. He clarified that it should have read, “in [the] absence of a permanently unique identifier”, and then delete “permanently” after identifier.

**Paton** suggested that the wording meant it would have to be permanently unique, and wondered how one could be sure that it would remain permanently unique.

**Thiele** called the question. [*Laughter*] He asked if he could call the question for both the amendment and the proposal?

**Knapp** said there would be no stacking up of calling the questions.

[The Section voted **to vote** on the amendment, and the **amendment** was **rejected**.]

**De Lange** then called the question. [*Laughter*]

**Knapp** said that, technically, she was not supposed to allow this, but given that the proposal concerned a Recommendation and it had been discussed for quite a long time, she would allow it.

[The Section voted **to vote**.]

**Rec. 40A, Prop. F** was **rejected**.

#### Article 41

**Art. 41, Prop. A** (3: 56: 5: 1) was **rejected** based on the **mail vote**.

**Art. 41, Prop. B** (3: 2: 62: 0)

**Turland** noted that this proposal was editorial: it would move the reference “see Art. 6.10 and 6.11” so that it did not seem restricted to new combination, name at new rank, or replacement name, but could also refer to basionym or replaced synonym. The Rapporteurs felt that in the new position it could be interpreted as being restricted to basionym or replaced synonym. The added reference to Art. 58.1 was intended to cover cases where an apparent new combination referred to an apparent basionym that was in fact illegitimate, so that, as described in Art. 58.1, a replacement name was published instead. The Rapporteurs felt that the Editorial Committee could determine the best places for these references in order to achieve the desired effect of the proposer.

**Art. 41, Prop. B** was sent to the **Editorial Committee**.

**Art. 41, Prop. C** (3: 49: 12: 0), **Prop. D** (1: 60: 3: 0), **Prop. E** (3: 55: 6: 1) and **Prop. F** (5: 52: 7: 1) were **rejected** based on the **mail vote**.

**Art. 41, Prop. G** (1: 12: 65: 0) was **automatically** sent to the **Editorial Committee**.

**Art. 41, Prop. H** (33: 27: 4: 0)

**Turland** stated that this proposal would amend Art. 41.4 so that certain failed new combinations or names at new rank may be validly published as such, rather than as the names of new taxa or replacement names, which could be undesirable and result in two different names with the same epithet, with priority from different dates, and with the same type or with different types. The latter could even be illegitimate and block the desired transfer, resulting in further change.

**Gereau** thought by removing the limiting date, this proposal allowed for continued bad practice to accommodate what were very few cases and it was therefore undesirable.

**Art. 41, Prop. H** was **rejected**.

**Art. 41, Prop. I** (39: 18: 7: 0)

**Turland** explained that Prop. I would place a change of emphasis in Art. 41.4 to make it easier to apply. Instead of evidence of the author’s intent to publish a new combination or name at new rank, it would require evidence that the author’s intent was different.

**Gereau** said this proposal lowered the criterion for application of Art. 41.4 to no apparent benefit, and he did not support it.

[The vote on the proposal by show of hands did not clearly reach the 60% majority required.]

**Hawksworth** called for a **card vote**.

**Knapp** chided the Section about their sloppy card voting, saying that even though she had harangued the members the day before about using the right card, there were still two misplaced cards in the previous card vote. She reminded everyone this time to only use the card that had the number “5” on it for personal votes and institutional votes. People who did not put the correct number in this time would be in big trouble!

**Funk** assured the voters that they would look for DNA and fingerprints if there were misplaced cards.

**Turland** suggested a SWAT team could deal with misplaced cards.

**Art. 41 Prop. I** was **rejected** based on the **card vote** (249 yes: 283 no; 46.8% yes).

**Art. 41, Prop. J** (3: 8: 55: 0), **Prop. K** (3: 0: 62: 1), **Prop. L** (4: 0: 62: 0) and **Prop. M** (4: 5: 57: 0) were **automatically** sent to the **Editorial Committee**.

**Art. 41, Prop. N** (4: 52: 9: 0) was **rejected** based on the **mail vote**.

**Art. 41, Prop. O** (32: 28: 4: 0)

**Turland** cheerfully announced that Prop. O was a rare opportunity to simplify the *Code*.

**Knapp** cheered.

**Turland** explained that this proposal would rid Art. 41.5 of one of the important dates in the *Code*, associated with what the Rapporteurs described as a useless provision. On or after 1 January 2007 the basionym or replaced synonym must be cited. However, the *Code* already stated that it must be clearly indicated, and full and direct reference given to its author and place of valid publication with page reference or date. Moreover, if it was cited incorrectly would it be correctable under Art. 41.6 or would it fail to satisfy Art. 41.5? The proposal would delete this phrase and the consequences would be minimal or negligible.

**Knapp** suggested the consequences would be 28 fewer words in the *Code*!

**Govaerts** thought this was put into the *Code* because there were some inadvertent new combinations when authors thought something was a subspecies, but in fact it had been published as a variety. The name at new rank would be validly published because they had *indicated* the basionym, having given its author and full bibliographic reference, but they had not actually *cited* the basionym because instead they had cited their name at new rank. The wording was inserted to prevent this happening.

**McNeill** added that while he had no particular opinion, he could confirm what Govaerts had just said. The proposal came from Katherine Challis and it was as a result of a particular IPNI problem, with a citation of a place of publication, a full and direct citation, but the actual basionym itself was not cited. He thought it was a very rare case and was not convinced it was needed.

**Gandhi** recalled that based on the current wording in the *Code* a few new combinations were treated as not validly published. If the words were removed, some names that were currently considered not validly published would become validly published.

**Govaerts** estimated he came across such situations about once a month, mostly in floras, where people assumed something was published as a variety, but it was not and they would inadvertently make a new combination. Authors could get very upset if they made a combination that they did not intend to make and it was entered in IPNI. He would prefer the wording to remain.

**Greuter** noted that restrictions in the *Code* were usually made for a good reason: to avoid something undesirable. The examples that had been given, in which this provision proposed for deletion would work, were those in which a new name, or a name at new rank in this case, was needed in the author’s opinion. If the author failed to see that, in order to have that name, he must publish a nomenclatural novelty but unintentionally publishes it anyway, there was nothing wrong with it. On the other hand, there were situations in which, according to much classical precedent, an author indicated a basionym and it was perfectly clear which basionym was meant, but the basionym itself was not concretely cited and the new name would then not be validly published. He saw no reason and nothing to be gained by such a restriction.

**Xiang-Yun Zhu** suggested that the modified Art. 41.5 should be cross-referenced to Art. 33.1.

**Turland** said that, though the Rapporteurs were not immediately agreeing, the Editorial Committee would look into it and would add a cross-reference if necessary.

**Knapp** announced that because the vote by show of hands was 50%, even-steven, it did not receive the required qualified majority and the proposal was not accepted.

**Turland** called for a **card vote**.

**Knapp** pointed out, holding up a fan of four card voting slips, that there had been four cards that had the number “4” [instead of “5”] on them in the last card vote. She reminded the Section that this was card vote number six.

[*The Section broke for afternoon tea.*]

**Knapp** welcomed everyone back, and announced the results of card vote number six, for which she said there were quite a few nines used, “Only kidding”. [*Laughter*]

**Art. 41, Prop. O** was **rejected** based on the **card vote** (237 yes: 267 no; 47.0% yes).

**Art. 41, Prop. P** (4: 56: 6: 1) was **rejected** based on the **mail vote**.

**Art. 41, Prop. Q** (37: 19: 10: 0) and **Rec. 41A, Prop. A** (36: 21: 8: 0)

**Turland** said that Prop. Q would add a new Note with two new Examples. The Examples would go to the Editorial Committee. Rec. 41A Prop. A went with Prop. Q and they could be discussed together. Prop. Q provided a clarification of what would constitute a full and direct reference under Art. 41.5 for journals with differing editorial styles. Rec. 41A Prop. A would adjust Rec. 41A.1 in the event of acceptance of Prop. Q.

**Wiersema** added that Rec. 41A said that the place of publication of the basionym or replaced synonym should immediately follow a proposed new combination. The proposal sought to split part of that out and have it in the bibliographic portion of a document, rather than immediately following.

**Sennikov** explained that the idea of these proposals came from a debate around journal styles. One was the traditional style, which was in the *Code* and in many publications. The second was the style represented by the journal *Phytotaxa*, which was remarkably different from the style used previously. Some people were unsure if the new style satisfied the conditions for valid publication. Because there could be more than one way of presenting the nomenclatural information in taxonomic publications, the proposal aimed to clear that up.

**Gereau** thought these two proposals encouraged undesirable practice. He said the plant taxonomic and nomenclatural community spent decades developing a full in-text bibliographic citation style that was fully informative, fully self-contained, did not require a reference to the bibliography at the end, made the text fully readable, and was very well established. He thought it one of the great strengths of the plant nomenclatural format and it should not be abandoned.

**Gandhi** mentioned that for the purpose of indexing newly published names, having to find a cited reference in the literature section of a publication added more time to what was already a time-consuming job.

**Kirk** noted that this also applied to fungal nomenclature and opened the option for people to make mistakes, specifically regarding page numbers. For example, author and year might be given in the text, but the references at the end of the article might have the full pagination and would result in a name not being validly published.

**Greuter** agreed with Gereau and Kirk about the *Phytotaxa* style, which he thought was undesirable and unpalatable. It was also against a published Recommendation in the current *Code*, and he thought it appalling that a taxonomic journal would force authors to go against the Recommendation in the *Code.* For this reason, he had abandoned his role as a section editor of *Phytotaxa*.

**Saarela** thought that making it clear in the *Code* that this format was allowed was a good thing. He argued that, throughout the history of botany, the community had done botanists a disservice by not providing full bibliographic information about where the names of new taxa were published. It was very difficult to find papers in old literature when they were only in microcitation format.

**Lindon** pointed out that microcitations in the text prevented taxonomic papers from getting higher impact factors. The in-text citations were not counted the same way as bibliographic ones. She thought it would help boost the visibility of taxonomic papers if they were used and cited in the way that all the other papers were.

**McNeill** followed up Lindon’s remark by saying that this had been one of the arguments for *Phytotaxa* and other journals doing it in that way, but it seemed to him that it could be covered both ways. An author could provide the concise, traditional botanical reference and, in parentheses, put the bibliographic reference, which would refer to the full reference appearing at the end of the article. That bibliographic reference need not then have the page numbers and so forth, as they would already have been provided.

**Sennikov** wanted to say that the citations in *Phytotaxa* were full and complete, more so than traditional citations, because those were in a condensed style.

**Knapp** corrected the proposer, saying that this was not necessarily true and that the point he had just made had already been made by Saarela.

**Art. 41, Prop. Q** was **rejected**.

**Turland** noted that the previous proposal would have adjusted Rec. 41A.1 in the event of its acceptance, but it had just been rejected.

**Knapp** thought the two proposals (Art. 41 Prop. Q and Rec. 41A Prop. A) were different and required a separate vote.

**Rec. 41A, Prop. A** was **rejected**.

**Art. 41, Prop. R** (11: 49: 3: 0) and **Prop. S** (10: 50: 3: 0) were **rejected** based on the **mail vote**.

**Art. 41, Prop. T** (7: 47: 10: 0) was contingent on **Art. 41, Prop. R** and **Prop. S** and was therefore **automatically rejected**.

**Art. 41, Prop. U** (8: 49: 7: 0) and **Prop. V** (3: 56: 5: 1) were **rejected** based on the **mail vote**.

**Art. 41, Prop. W** (54: 7: 4: 0)

**Turland** explained that this proposal addressed situations that Art. 41.8(a) failed to cover: when “the name cited as the basionym or replaced synonym” was not the actual basionym or replaced synonym. That is, the cited name and the actual name represented different combinations for the same taxon placed in different genera or species or at different ranks.

**Sennikov** addressed the proposer [Greuter], asking about the meaning of “isonym” in this wording, because he interpreted it as including reuse.

**Greuter** asked if amending it to “a later isonym” would make it clear.

**Knapp** said that **amendment** would be, by definition, **friendly**.

**Sennikov** reiterated that he was confused about what should be called an isonym, and what should not be called isonym, and the difference between “later isonym” and “reuse”.

**McNeill** suggested the disagreement, if there was one, between Greuter and Sennikov was totally editorial and should be dealt with in that way.

**Art. 41, Prop. W** was **accepted as amended**.

**Art. 41, Prop. X** (45: 13: 6: 0)

**Turland** introduced Prop. X by saying it would add a phrase to close a gap in Art. 41.8(a), in which the phrase “reference to the actual place of valid publication” could be taken to mean a reference in the general references of a work and not in the context of the “name cited as the basionym or replaced synonym”.

**Gereau** thought the proposal did, indeed, prevent a possible misunderstanding, but it seemed so improbable that it would be legislating for something that was highly hypothetical and, on that basis, he opposed it.

**McNeill** assured Gereau that he did not go in for hypothetical proposals and this one had come about because he had a conservation proposal in which this situation had arisen. The reference was in the bibliography and related to a totally different part of the publication, but following the present wording of Art. 41.8(a) literally, the name would have been validly published.

**Art. 41, Prop. X** was **accepted**.

**Art. 41, Prop. Y** (39: 9: 15: 0)

**Turland** said Prop. Y slightly adjusted the wording of Art. 41.8(c) and Art. 41.8(d), so that it was neutral as to the actual intent of the publishing author, who may appear to have published a new name, either intentionally or unknowingly. The proposal demonstrated that the current Art. 41 Ex. 24 was incorrect and should be deleted. If Prop. Y was accepted, the Editorial Committee would consider whether the offered new Example was too complex.

**Art. 41, Prop. Y** was **accepted**.

**Art. 41, Prop. Z** (3: 56: 4: 2) was **rejected** based on the **mail vote**.

#### Recommendation 41A

**Rec. 41A, Prop. A** was discussed under **Art. 41, Prop. Q**.

**Rec. 41A, Prop. B** (52: 8: 6: 0)

**Turland** said Prop. B was linked to Rec. 30A Prop. C, which strongly recommended publishers include page numbers on the actual pages of publications. This had been discussed and accepted earlier. The current proposal recommended that, in line with the style of the *Code* on how to cite page numbers in unpaginated electronic publications, pages should be referenced with square brackets. An Example of *Crocus antalyensioides* was provided.

**Mabberley** asked why this was not applicable to unpaginated printed matter from the last century?

**Turland** said that this point had been in the Rapporteurs’ notes as well, but they were going to wait and see whether somebody else brought it up. [*Laughter*]

**Lindon** explained that Emma Williams, who had written this proposal, was on maternity leave but had authorized Lindon to speak on her behalf. The IPNI team supported the proposal in order to ensure that unpaginated electronic publications were treated in the same way as paper publications. Elsewhere in the *Code*, electronic publications were sometimes treated differently or had slightly more ambiguous pagination, and this would clarify the situation.

**Govaerts** replied to Mabberley’s question: the proposal did not address all publications because for some older publications there was a tradition on how to cite the pages. If the *Code* started recommending doing something different it might be disruptive.

**Groom** suggested getting rid of “electronic” so that the Recommendation would apply to all publications. He thought, regardless of traditional usage, putting page numbers in square brackets gave more information to people trying to find the page.

**Lindon** accepted the **amendment** as **friendly** on behalf of the proposer.

**McNeill** said he would be horrified to have to cite Miller’s *Gardener’s Dictionary* by counting the pages for the square brackets. He reiterated Govaerts’s comment, that there was a traditional way of doing that, by referring to the genus and then using square brackets. He thought it could not be taken as a general application, given the diversity of unpaginated works, particularly in the 18^th^ and 19^th^ centuries.

**Garland** asked if this was a common format that was already being used. It was confusing and looked like a single page was being cited. He suggested adding “*pp.*” after the “6” in the Example, to indicate that the total number of pages in the publication was being cited.

**Knapp** pointed out it was a page number that was being cited, not the total number of pages.

**Lindon**, speaking for Williams, said she thought it would be better to say, “in the absence of established tradition”, rather than “may”. That would get around concerns about publications that were already cited in a particular way. She gave the Editorial Committee license to find the correct wording, in line with the rest of the *Code*.

**Wilson** suggested, in line with Garland’s question, that since it was on the sixth page of the PDF, it should be cited as six in square brackets, just to make it clear.

**Redhead** said he would still like to change “should” to “may”, because this was a Recommendation.

**Turland** felt that “should” was perfectly okay for a Recommendation, although he supposed “should” was a little stronger than “may”, because “should” was telling people what they should do, whereas “may” was saying you may do it if you wanted to.

**Barrie** called the question.

[The Section voted **to vote**.]

**Rec. 41A, Prop. B** was **accepted as amended**.

#### Recommendation 41B (new)

**Rec. 41B (new), Prop. A** (21: 35: 9: 1)

**Turland** said that this proposal explicitly recommended adopting in bibliographic citations the standardized abbreviations given in the second editions of *Taxonomic Literature* (TL-2) and *Botanico-Periodicum-Huntianum* (BPH-2). He noted that this was already done in many botanical journals and in the *Code* itself, although it did not mention another standard, the IPNI publications database. He thought it may promote greater consistency in botanical citations. One thing to consider was that the *Code* did not explicitly recommend standards, but rather mentioned their existence, for example *Index Herbariorum* for herbarium codes.

**Wiersema** pointed out that the same was done with the author standard, Brummitt & Powell. It was mentioned as existing without recommending that it be followed.

**Dorr** said one of the problems with *Taxonomic Literature* was that it only covered a certain period in time, between Linnaeus and 1940, and was not currently an ongoing project.

**Knapp** quipped that nothing had been written since then. [*Laughter*]

**Dorr** agreed that nothing of importance had been written. He thought the proposal was helpful but did not provide the community with an ongoing guide that would get updated. He suggested that IPNI and other organizations were doing a better job of keeping the standards updated.

**Söderström** noted that another problem with recommending standards like this, including IPNI, was that they were mainly for vascular plants. Other groups are poorly represented in many of those indices.

**Nakada** proposed an **unfriendly amendment** to change this Recommendation to an Example, which would then go automatically to the Editorial Committee.

[The **proposal** was supported by **five seconders**.]

**Thiele** objected, asking how it could be an Example, because it didn’t look like any other Example he had ever seen in the *Code*.

**Knapp** suggested it might be an example of lists.

**Turland** agreed that Thiele had a point because this was a new Recommendation. If it was converted to an Example it was not actually an example of anything, it would have to be linked to a Recommendation or an Article to be an Example.

**Greuter** proposed an amendment to the amendment: to refer the proposed new Recommendation to the Editorial Committee, with the intent to transform it in a more neutral way, such as, “In the references formed” etc., “the titles of books should be abbreviated in conformity with existing international standards”, and list what was now there as examples of such standards. He would like to add to the listed examples of the standards, *Taxonomic Literature* in particular, the qualification “as updated through the IPNI”.

[The **amendment** to the **amendment** (sending the Example to **Editorial Committee**) was supported by **five seconders**.]

**Funk** was opposed to giving the Editorial Committee the task of rewriting the proposal without any good instructions for it.

**Saarela**, having just checked BPH-2 on the web, noted that current popular journals were not updated in their list. For example, the recent journals *PeerJ*, *PLoS ONE* and *Phytotaxa* were not included.

**Knapp** pointed out that the discussion was on whether to refer this to the Editorial Committee which, she reminded the Section, did not mean that it went into the *Code*, because the Editorial Committee may decide that it was not very useful.

[The vote to refer the proposal to the Editorial Committee achieved less than the 60% majority required. The Section therefore reverted to discussing the proposal.]

**Levin** called the question.

**Greuter** raised a point of order, saying the President did not ask the proposer [Sennikov] whether the amendment he moved to send the proposal to the Editorial Committee was a friendly one.

**Knapp** said that Sennikov had nodded his head when she asked him, but that the discussion had gone beyond that phase.

[The Section voted **to vote**.]

**Rec. 41B (new), Prop. A** was **rejected**.

#### Chapter V Section 4 Article n (new)

**Chapter V Section 4 Art. *n* (new), Prop. A** (13: 37: 1: 13)

**Turland** explained that this proposal sought to limit the principle of priority by preventing the acceptance of overlooked or unrecorded names. To achieve this goal the proposers conceived the concept of the “IPNI 2020 List”. While an actual list could, in theory, be generated by IPNI on 1 January 2020, the proposed provision was designed to avoid placing any additional burden on the staff of IPNI or its successor, if any, so that the list was in fact a virtual one. *Code* users would have to be able to interpret the notes in the IPNI record history correctly: IPNI had a record history which showed how the record had been edited over the years, and this would be used to determine the date on which a name was added to IPNI. Guidance might be provided on the website. There was also a rule for those managing the IPNI data: names may not be added to or deleted from the list.

**Smith** said that this was not a new matter in any respect. The proposal tried to address the tension between priority on the one hand and stability on the other. For the first time since the 1930s, when the first efforts were made to have *Index Kewensis* serve as what was being called “IPNI 2020” here, we had the benefit of an electronic dataset. Over 50 years ago, the comment had been made that perhaps by the year 2000 sufficient work would have been done to enact something like this. The effort here was probably sparked when Jim Reveal used some informative bibliographic datasets and started unearthing thousands of names that had been overlooked. This meant that names competing with ones that were currently in use and widely accepted must have their validity checked, or names would have to be proposed for conservation or rejection. This could be exceedingly time-consuming.

Smith stated that this would obviously be very contentious and for that reason there was a fairly comprehensive indication in the supporting paper. However, he wanted to assess the mood and sentiment from the floor.

**Schori** said she had some serious concerns about this proposal. In her job, she dealt with a lot of horticultural names and, because of all the online databases that were available, it was possible to find places of valid publication of names that were treated as unpublished but used in horticulture. For example, she had recently found a place of valid publication of a name and sent it to Gandhi, who entered it into IPNI. Another concern was that there was a lot of literature, particularly from the former Soviet Union, that was not easily accessible. These names might only pertain to taxa that were endemic to those countries, but if they were not indexed, then potentially many names would not be considered validly published and she did not find that acceptable.

**Geltman** wanted to support the previous speaker with regard to the evident lack in IPNI of “Conspectus of Plants of Middle Asia” and “Flora of West Siberia” [presumably the Russian-language *Opredelitel’ Rastenij Srednej Azii* and *Flora Zapadnoĭ Sibiri*]. He felt it was premature to incorporate such provisions.

**Marhold** said that while he also appreciated efforts to improve IPNI, his recent experience with central Europe and species names of the genus *Alyssum* was that there were still many errors, and there were several names currently in use that were missing from IPNI. He thought the idea itself was not bad, but 2020 was a very early date.

**Greuter** was sympathetic to the proposal and to the reasons that led to it: the continued and increasing discovery through the web, via text recognition, of scanned old remote literature, especially from horticultural quarters. These discoveries were a threat to nomenclatural stability and nomenclatural work in general, and he predicted it would increase rapidly and dramatically.

On the other hand, he was also aware of some shortcomings of IPNI, an instrument that was invaluable, and he emphasized that he was not criticizing it. He and Professor Hilger in Berlin did a search on *Cynoglossum* and came up with concrete figures of names lacking in IPNI, which were not shattering, but there were names that were used in the literature, not remote and unknown names, but names that were generally used and known, which for some reason were not in IPNI.

Another difficulty with the proposal was the concern that if a name was not found in IPNI by searching, it would be considered not validly published. However, that name may just have been misspelled in IPNI such that it did not appear in a search. Greuter moved to refer the proposal to a newly established Special-purpose Committee on a “List of Available Names”, which is what the zoological community called these kinds of things.

[The **proposal** was supported by **five seconders**.]

**Barrie** was in favour of the principle because he thought there were considerable problems with names emerging from the depths of obscure literature. However, he thought it was premature to do it now, therefore sending the proposal to a Special-purpose Committee was exactly the right way to make sure that if something was put into the *Code* it could be relied on, would be stable in the future, and would be something that everyone would have confidence in.

[The **proposal** to establish a Special-purpose Committee only required a simple majority, and the **Special-purpose Committee on “Lists of Available Names” was established**.]

**McNeill** asked about the remit of the Committee. The proposal dealt only with vascular plants, but he wondered if the remit was to be restricted to vascular plants or whether it should consider names in other groups falling under the *Code*?

**Knapp** asked if he wanted to make a proposal to that effect for the Section to vote on.

**McNeill** said he did not, he was just hoping that the Section would get it solved. He thought the proposer might have a view.

**Greuter** thought fungi could safely be excluded as they already had their own mechanisms in place to deal with the problem, but he did not want to limit it to vascular plants. He supposed the remit of the Committee could be so defined as taking care of the rationale for the current proposal under consideration.

**Knapp** asked the Secretary of the Nomenclature Committee on Fossils [Herendeen] to say whether he would like fossils to be part of this exercise.

**Herendeen** explained that the compilation of names of fossils was woefully inadequate, so while it might be useful to participate in the discussion, there would not be anything for fossil names by 2020.

**Knapp** pointed out this would not be 2020, it would be a Special-purpose Committee to bring something to the next Nomenclature Section in 2023.

**Herendeen** agreed that it would be useful to have someone from the Committee on Fossils.

**Price** said four people from the Nomenclature Committee for Bryophytes were there, and if the idea was going to be opened up to all plants, bryophytes would need to be part of the discussion. However, it was acceptable to just treat the vascular plants for the moment. She added that bryophytes did not yet have a centralized indexing system.

**Knapp** summarized the discussion by saying that the Special-purpose Committee would have a remit to look at establishing these lists for vascular plants. There would be participation from other communities in the Special-purpose Committee with a view to looking at how it might be achieved across the *Code*.

**Barkworth** asked if the Special-purpose Committee could look at the costs of achieving this.

**Knapp** noted that Special-purpose Committees did not usually have specialized remits, and that the Section was just trying to establish whether to include in the Special-purpose Committee the communities across the *International Code of Nomenclature for algae, fungi, and plants*.

**Kusber** spoke on behalf of the algae community, pointing out that while it was too early to have lists for algae, it was urgent to have lists for cyanophytes or cyanobacteria because of cross-domain problems with the *International Code of Nomenclature of Prokaryotes*.

**Knapp** reminded the Section that there had been Special Committees established to deal with these names in the last two Congresses and nothing had happened. Special[-purpose] Committees did not always come to the Nomenclature Section with proposals to do things. She used her President’s prerogative to say that she thought there was a feeling that this was a problem. It was a problem that the community recognized, but it seemed too soon to be doing it. Doing it through an instrument like IPNI would be difficult, because to base it on something that was run out of a single institution, or a few institutions, put a very big burden on those institutions. She urged everyone in the community to think of ways to come up with solutions to this problem. The mycologists had come up with solutions to this in abandoning dual nomenclature, and the Section could benefit from their experience. Extending participation across the *Code* would probably lead to a better solution than would be achieved by limiting it very narrowly.

**Chapter V Section 4 Art. *n* (new), Prop. A** was sent to the newly established **Special-purpose Committee on “Lists of Available Names**”.

#### Article 42

**Art. 42, Prop. A** (13: 45: 4: 3)

**Turland** relayed the Rapporteurs’ ideas, saying that the proposal addressed a semantic point: the words “name” and “names”, as used in Art. 42.1 and Art. 42.2, may not in fact be names as defined in the *Code* in Art. 6.3 if they were not yet validly published. The statement, “neither identifier nor name can be changed” did not seem to be explicit or implicit elsewhere in the *Code* and would therefore be better as an Article instead of a Note, using a verb other than “can”. Similarly, the phrase “authors should” would be changed to “authors must”, but these were relatively minor editorial issues.

The Nomenclature Committee for Fungi [NCF] did not support Prop. A; instead the Committee supported an alternative approach that would treat errors in citation of identifiers as correctable. He had been informed by Tom May, the Secretary of the Committee, that this would be the subject of a proposal to be moved from the floor of the Nomenclature Section.

**May** said the fungal taxonomists had been discussing this and explained that the proposal contained three different things: one was the semantics; the second was dealing with the possibility of change between the name as registered and the name as published; thirdly, there was the issue of mis-citation of the identifier. The fungal group intended to bring up proposals from the floor, but an *ad hoc* committee comprising representatives of the repositories and the NCF had been working on this over the last couple of days. They would continue to work on it and publish a proposal that they hoped would be dealt with at the Nomenclature Session of an IMC. He moved a **friendly amendment** to this proposal to remove the material that did not relate to the semantics and consequently ask that the semantics be sent to the Editorial Committee.

**Kirk** replied that the cunning plan proposed by “Baldrick” was **accepted** as a **friendly** amendment. [*Laughter*]

**Knapp** explained that those who were not English would not have understood Kirk’s cunning reference and offered to explain over dinner. [*Laughter*] She said that the proposed change would be left until later. The first part, regarding semantics, had been proposed for referral to the Editorial Committee, while the rest of the proposal would be dealt with by mycologists generally.

[*Kirk’s cunning reference: Baldrick, a character in the British television pseudo-historical comedy “Blackadder” notorious for his cunning plans to overcome adversity: https://en.wikipedia.org/wiki/Blackadder*]

[The **proposal** was supported by **five seconders**.]

**Art. 42, Prop. A** as **amended** was sent to the **Editorial Committee**.]

**Knapp** then used her President’s prerogative to postpone the discussion on the proposals that were put forward by the Special Committee on Registration, Art. 42 Prop. B, Prop. C, Prop. D and Div. III Prop. A, until the next morning when everyone would be a little bit less tired and the Section would be likely to finish something in a whole session. She asked if there was anybody to second that?

[*Lots of seconders and laughter; the discussion of these proposals was postponed until the beginning of the Thursday morning session.*]

#### Article 45

**Art. 45, Prop. A** (3: 4: 59: 0)

**Turland** noted that Prop. A was editorial and it sought to make the footnote of Art. 45 Ex. 1 more accurate, because the term “available” was used in more than one sense in the zoological *Code*. The Rapporteurs suggested it could be sent to the Editorial Committee, who would look closely at it and, if necessary, seek advice from the zoological community. He proposed that it be sent to the Editorial Committee.

[The **proposal** was supported by **five seconders**.]

**Art. 45, Prop. A** was sent to the **Editorial Committee**.

**Art. 45, Prop. B** (3: 55: 6: 0) was **rejected** based on the **mail vote**.

#### Article 46

**Art. 46, Prop. A** (48: 22: 8: 0)

**Turland** introduced the proposal with the Rapporteurs’ comments, saying it might make Art. 46 slightly less austere by implying, almost at the beginning, that in most cases a name was attributed to the author of the publication in which it appeared. He allowed that it would still be necessary to read all the other provisions to determine if they ruled otherwise, but at least one could embark on one’s journey not utterly clueless.

**Knapp** chimed in with “Like me.”

**Turland** thought the proposed Note helped understand a case that was not explicitly covered elsewhere in Art. 46: when a name of a new taxon appeared in a publication by Author A and was ascribed to Author A and the validating description or diagnosis was ascribed to Author B.

**Greuter** said he had a question rather than a comment. Usually when a new Note was proposed it stated where it would go in the *Code*, but he did not see an obvious placement for this. He thought it could hardly be at the head of the Article.

**Knapp** and **Turland** thought it would be following Art. 46.1.

**Greuter** pointed out that this referred to the Example, not the Note.

**Knapp** said that the proposal read “Add a Note”.

**Gereau**, upon reading this through and pondering it, found that the Note added more confusion than clarity. His major fear was that it could be over-applied by people who did not want to think about things carefully. He saw potential for creating error and confusion and did not support it.

**Wilson** supported the proposal but felt that the second half of the Note, starting from “when” was hard to read. She thought it could be better reworded as “unless one or more of the following provisions rules otherwise” and proposed this as a friendly amendment.

[McNeill **accepted** the **friendly amendment**.]

**Art. 46, Prop. A** was **accepted as amended**.

**Art. 46, Prop. B** (7: 40: 18: 0)

**Turland** said Prop. B would add a new Note under Art. 46.1. The change would be essentially editorial, because Notes explain something that may not at first be readily apparent but is covered explicitly or implicitly elsewhere in the *Code*. The proposed Note summarized how an author citation may function and this was already explicit in the relevant rules of Art. 46. Those who felt that this was not at first readily apparent might consider the Note to be useful.

**Gereau** stated that upon reading this he understood the Article less clearly than he had before.

**Art. 46, Prop. B** was **rejected**.

**Art. 46, Prop. C** (40: 16: 11: 0)

**Turland** said that the Rapporteurs had commented that Prop. C made [the second sentence of] Art. 46.2 more precise by replacing the rather vague words “some way” with “a relevant way”. There was an adjustment to Ex. 7 to help make this point. Further to the Rapporteurs’ comments, the Section would need to be able to discern between relevant and irrelevant contributions, otherwise Art. 46.2 would become more difficult to apply.

**Gereau** thought that if the author of a publication ascribed a taxon name to another author and stated explicitly that the other author contributed to the publication, it may be assumed that the contribution was relevant, whether or not the nature of the contribution was explained. He thought it unlikely that an author would ascribe a name to someone else if they did not think that the contribution was relevant.

**Schori** explained that there were cases where there were acknowledgements that people had contributed to the manuscript in one way or another. In one case, someone was thanked for assistance with the proofs and that person was currently listed as an author because of ambiguity in the work. She was in favour of the proposal, because in some cases there was an indication that a contribution was not relevant, but someone was nonetheless listed as an author.

**Applequist** wondered if this was a common issue. She asked what the definition of “relevant” was and who was going to decide that. She wondered how many names now considered validly published might be rendered not validly published .

**Kirk** did not think this related to valid publication as it only concerned authorship.

**Turland** agreed.

**Kirk** related a recent case in mycology where the author citation of a name of a new taxon included an author who did not contribute anything to the publication and was unaware that he was going to be included in authorship. There was no relevant indication [elsewhere] in the manuscript that he was an author. This clarification would allow the removal of that author’s name.

**McNeill** addressed a question to Kirk, asking if this author was recognized or acknowledged in some way in the work.

**Kirk** said he could not recall the details, but there was a little storm in a teacup within various e-mails.

**May** clarified that if any of the authors of that name were included in the authors of the paper, the author name could not be removed.

**Gandhi** suggested that the terms “relevant” or “in some way” were sometimes debatable. For example, in *Flora of British India*, for seven volumes J. D. Hooker was the editor. Some of the new combinations were ascribed to him, but his name was not mentioned as author of the relevant flora treatments. There was debate about whether J. D. Hooker should be the author of the new combinations, or whether he should be an “ex” author. Gandhi argued that since Hooker was listed as the editor, he had contributed in some way to the publication of the treatments. Some people agreed and others disagreed with this interpretation. Gandhi agreed with Applequist that unless the ascribed author had done significant work, there was no reason for the author of the article to ascribe that name to him. He noted that Schori’s comment regarding the proofreading referred to a very famous botanist, [James] Dandy. The name was ascribed to Dandy, and Dandy had read the proofs, so it was not any Tom, Dick, or Harry reading the proofs: it was a very famous botanist who was likely to have made a significant contribution to the publication of the article. He was therefore against the current proposal.

**De Lange** called the question.

[The Section voted **to vote**.]

**Art. 46, Prop. C** was **rejected**.

**Art. 46, Prop. D** (6: 45: 14: 0) concerned an Example contingent on **Art. 36, Prop. E** and was therefore **automatically rejected**.

**Art. 46, Prop. E** (7: 8: 51: 0) was **automatically** sent to the **Editorial Committee**.

**Art. 46, Prop. F** (34: 9: 22: 0) and **Prop. G** (15: 8: 42: 0)

**Turland** explained that these proposals were linked and that Prop. G, as an Example, would be sent to the Editorial Committee, which might also expand the Glossary entry for “descriptive name”, as it had been mentioned to him before that the Glossary definition of that term could be improved.

Prop. F was purely a Note, and for those who preferred to cite the authors of descriptive names above the rank of family, this might be a useful explanation and could be sent to the Editorial Committee. These proposals would make clear that the authorship of descriptive names did not change when they were used at different ranks, because they were not thereby names at new ranks. He noted that if Prop. F was accepted, the new Note would be better placed at the end of Art. 49.

**Barrie** stated that descriptive names were used above the rank of family, at which priority did not apply. His understanding was they could be redescribed and the authorship could change every time they were used, because there was no priority. Anyone could use the name again and ascribe it to themselves.

**Gereau** said Prop. F followed immediately on from the idea behind Art. 6 Prop. L, which the Section had declared desirable, because the authorship of a descriptive name would not thereby change. The Section had already established the principle, and Prop. F was entirely consistent with Art. 6 Prop. L, which had already been approved.

**Turland** said it was true that it was not a name at new rank. Barrie’s comment made him wonder whether the Rapporteurs had erred in their comments.

**Wiersema** wondered if those names actually had authors.

**Turland** queried whether every time a descriptive name was used, did it count? Was it the very first author ever to use a descriptive name or was it effectively the name of a new taxon? He asked if there were any instructions in the *Code*.

**Wiersema** did not think so, and asked if there was something in the *Code* that said such names did not even have authors?

**Turland** noted that Art. 6 Prop. L had in fact been sent to the Editorial Committee.

**Funk** suggested it might be time to call it a day.

**Gereau** said he retracted his statement about the acceptance of Art. 6 Prop. L and recommended that Prop. F also go to the Editorial Committee. He said he would be perfectly happy with that, if his notes were wrong.

[The **proposal** was supported by **five seconders**.]

**Nakada**, as the proposer, wished to explain that his purpose was to clarify how to cite the authorship of the higher-ranked taxa, however that may be accomplished.

**Turland**, speaking to the proposal to refer to the Editorial Committee, said that the Editorial Committee would ensure that the Note was supported by whatever was explicit or implicit elsewhere in the provisions of the *Code*.

**Redhead** wished to comment that there were some basic errors in the proposal.

**Knapp** asked if he was alerting the Editorial Committee to watch it?

**Turland** assured him that the Editorial Committee always watched it. [*Laughter*]

**Greuter** said he was not persuaded that the Editorial Committee always watched it. [*Laughter*] He had been asking himself: were alternative family names descriptive names? The answer was no, because they had types. However, they could be used unchanged above the rank of family, in which case, they would have an author citation which differed from the author citation of the alternative name. He gave examples of *Leguminosae* or *Compositae*.

**Art. 46, Prop. F** was sent to the **Editorial Committee**.

**Art. 46, Prop. G** was **automatically** sent to the **Editorial Committee**.

**Art. 46, Prop. H** (3: 41: *20: 0) and **Prop. I** (2: 53: 9: 0)

**Turland** paraphrased from the Rapporteurs’ comments that Prop. H and Prop. I sought to address a perceived difficulty in distinguishing between ascription of a name to an author under the second sentence of Art. 46.2 and an indirect reference to a basionym, replaced synonym, or homonym, as mentioned in Art. 46.3. The proposers believed that citing the name of an author could be both an ascription and an indirect reference. This, in the opinion of the Rapporteurs, was contrary to Art. 46.3. The Rapporteurs found it hard to understand how the proposed solution, replacing the word “reference” with the phrase “a mere reference” in Art. 46.3 and adding two seemingly inconsistent Examples, would add to the clarity of Art. 46. Turland noted that Prop. H also removed the phrase “formal error” from Art. 46.3, which was unexplained in the *Code* until the Editorial Committee provided the current Art. 46 Ex. 19 in the *Melbourne Code*. Hence, Ex. 19 was invented for the phrase “formal error”, not the opposite as the proposers stated. Those who disagreed with Prop. H but agreed to the deletion of “formal error” had been invited to vote “ed.c.” in the mail vote.

**Greuter** moved that the proposal be sent to the Editorial Committee on the understanding that it acted in accordance with the Rapporteurs’ comments: deleting the unexplained, and in many ways inexplicable, last phrase. He recalled that this was brought in following a memorable session in Gea Zijlstra’s presence, trying to bring some sense into Art. 46, and it had had some success. He went on to say, however, that no one afterwards could explain why this phrase was put in and no one ever made a reasonable case for placing any concrete Example under it. In fact, the Editorial Committee for the *Melbourne Code* made a search for a suitable Example and added something, now Ex. 19, without much conviction that it was appropriate.

[The **proposal** was supported by **five seconders**.]

**Art. 46, Prop. H** was sent to the **Editorial Committee**.

**Art. 46, Prop. I** (2: 53: 9: 0) was **rejected** based on the **mail vote**.

**Knapp** closed the session by telling everyone they were excused. She reminded everyone of where to find the General Committee reports. She noted that the next morning’s discussions would start with the proposals from the Special Committee on Registration, which were Art. 42 Prop. B, Prop. C, Prop. D and Div. III Prop. A.

### Thursday, 20^th^ July 2017, Morning Session

**Knapp** welcomed delegates back to day four, noting that the Section was more than halfway finished, but that there was still a lot of business to get through and she wanted to ensure there was time to deliberate properly on any proposals that came from the floor of the Section on the final day. She reminded delegates that the deadline for proposals from the floor was the end of today and that they should be submitted to the Bureau in neat handwriting or electronically. She stressed that the end of the day meant 6 o’clock Shenzhen time because the Bureau would have to meet that evening to decide how to order the proposals. Five seconders would be required for each proposal submitted.

#### Registration of algal and plant names (Article 42 and Division III)

**Art. 42, Prop. B** (40: 25: 2: 1), **Prop. C** (40: 27: 1: 1), **Prop. D** (34: 32: 1: 2) and **Div. III, Prop. A** (49: 19: 1: 0)

**Turland** explained that this group of four proposals concerned the mandatory registration of names of organisms treated under “our *Code*”, in particular, those organisms not currently treated under Art. 42: algae and plants. The intent was to define a flexible framework within which a system of voluntary regulations could be developed for various categories of organisms. Div. III Prop. A was concerned with establishing a Permanent Committee on Registration. This would be an eighth Permanent Nomenclature Committee. This Committee was referred to in the other proposals so it made sense to deal with it first to find out whether the Section would approve the establishment of a Registration Committee. Art. 42 Prop. B established the mechanism for nomenclatural repositories to become authorized to register names. Art. 42 Prop. C clarified that registration may take three forms: proactive and/or synchronous and/or retrospective. What was currently in place for fungi was proactive registration where a potential name was first registered and was then validly published and the identifier from the nomenclatural repository had to be cited in the protologue. Art. 42 Prop. D established a mechanism whereby registration may be made a requirement for valid publication in between IBCs. It gave the General Committee the power, under specific circumstances, and after specific criteria had been met, to authorize registration as a requirement of valid publication. This authorization from the General Committee was subject to ratification of a subsequent IBC. The Rapporteurs had requested opinions from the General Committee on the proposals. Regarding Div. III Prop. A, the General Committee supported it by 21 “yes” votes to 2 “no” votes, with 2 abstentions. The General Committee also supported its proposed role in recognizing nomenclatural repositories and suspending or revoking such recognition, and its proposed role as described in Prop. D, where it had the power to make registration mandatory before the next IBC. He finished by stating that the General Committee was generally positive about all the registration proposals.

**Barkworth** thought that Turland had summarized the proposals very well. The Committee had hoped that by the time this Section was in session there would have been an opportunity for all groups to have had experience in registering names, but it was much more difficult to set up a nomenclatural repository than had been anticipated. Institutional support, a combination of financial support and support for staff time, was required and there was nothing on record yet to endorse the concept of registration going forward other than the formation of a Special Committee. Barkworth stated that this was why Div. III Prop. A was critical: it would put on record the fact that the Section wanted to implement registration. It could not be done immediately, but the Section would want to implement it going forward. This would help institutions in trying to obtain the support they needed to set it up. Barkworth noted that Div. III was a general statement designed to bring together the representatives of nomenclatural repositories, of the professional organizations in the different groups, and members of the General Committee, because they would need to interact and communicate with each other, as would any databases, so this was the overarching proposal.

Prop. B described what a nomenclatural repository would do, registering nomenclatural acts as well as new names. This was what a repository would be responsible for. The proposal laid out the groundwork for what kind of qualifications would be necessary and how institutions might put themselves forward to be a recognized nomenclatural repository; they must have been active for a while and been used by the community.

Prop. C said that it was up to the repositories to figure out how they wanted to be proactive.

Prop. D was, Barkworth thought, the most controversial and would give the General Committee the power to make registration mandatory for a group of organisms, subject to certain conditions. This proposal could also stop the process if, for example, a nomenclatural repository stopped functioning or there were complaints about it. It could also rule that registration was no longer mandatory and that could be done between Congresses, although it would have to be ratified at a Congress. The important thing was to provide a framework that would allow registration to move forward. She stated that the community needed registration and should not be adding to the backlog of names and name changes that were not known about because they were not in a database.

**Knapp** opened the discussion on **Div. III, Prop. A**, warning that she would be very strict about not allowing discussion of conceptual things in the other proposals while each was discussed individually.

**Paton** stated that he was strongly in support of the proposal but noticed that the Chair of the Committee would be specified, whereas in the other governance proposals to be discussed under Div. III, the Committee would have the power to choose officers within it. He felt that this was inconsistent. He had nothing against the current General Secretary of the IAPT, but it seemed strange to put it in a permanent piece of legislation. The Committee might work better if it had, as for other Committees, the power to elect its own officers. He proposed an amendment either to remove the sentence or to say, “The Committee has the power...”, taking exactly the same wording from paragraph 7.2 of the governance proposal [Div. III, Prop. B].

**Knapp** asked Paton to confirm his wish to strike the phrase, “It is chaired by the Secretary-General of the International Association for Plant Taxonomy or his/her deputy…”

**Paton** agreed, noting that the wording of the other proposal was, “The committees have power to elect officers as desired, to fill vacancies, and to establish temporary subcommittees…”

**Knapp**, from the point of view of the President, thought there should be an addition stating that the IAPT would be an ex-officio member of the Committee. She noted that, as not all members of the Special Committee were present, all **amendments** would have to be **unfriendly**.

[The **amendment** was supported by **five seconders**.]

**Thiele** stated that he strongly supported the amendment but asked the Committee to explain their thinking in specifying the Secretary-General as the Chair.

**Barkworth** replied that it was because it came under the role of the whole of the IAPT and the Secretary or deputy, but she did not object to the amendment.

**Greuter** wished to state that one of the ideas behind involving the IAPT at a high level was that the IAPT had always been viewed as a possible source for funding or facilitating matters related to registration. The IAPT had funded fully a trial run for registration preceding the St. Louis Congress [1999]. The IAPT had always shown remarkable commitment to nomenclatural causes and was a rich association. He added that during his tenure as the Secretary-General it had more or less quadrupled its assets.

[The amendment was **accepted**.]

**Funk** had an unfriendly amendment: she thought the Committee should be a Special-purpose Committee, not a permanent one, because there was currently no registration in the *Code* except for fungi. She thought it was premature to establish a Permanent Nomenclature Committee on something that was not currently in the *Code*. In addition, she thought the ideas were not developed enough to put them in the *Code*.

[The **amendment** was supported by **five seconders**.]

**Barrie** stated that the problem in returning this to a Special-purpose Committee was that it was not going to have the authority and weight it needed to get the institutions to back it. He thought there ought to be something in the *Code* that said the community was serious about doing this. The Committee could end up having nothing to do, but that would not be a problem. He thought there needed to be a Permanent Committee looking at registration so it could be monitored, so he opposed the amendment.

**Knapp** asked for further comment but warned that she would only allow a couple of comments from any delegate and then they would be cut off.

**Greuter** wished to draw the attention of the Section to the differences between Special Committees and Permanent Committees. A Special Committee, if it was mentioned in the *Code*, would presumably not have to be debated and decided again every Congress; this was no different to a Permanent Committee. However, a Permanent Nomenclature Committee was elected by the Section on the slate of future membership drawn up by the Nominating Committee, whereas a Special Committee was nominated by the General Committee and set up at the discretion of the General Committee.

**Watson** asked Funk what the remit of the Special-purpose Committee would be, because it seemed to him that many of the things in the proposal would be beyond the remit of Special-purpose Committees. He asked what a new Special-purpose Committee would be doing beyond what the existing Special Committee on Registration had already done.

**Funk** replied that she was not convinced that the Special Committee had done their job very well. She did not think their ideas were well developed. When the [Melbourne, 2011] Section debated this for fungi, their Committee came to the table with a well-developed proposal, which they had spent years on, and she had been very impressed with it. They convinced her that they knew what they were doing, they had a plan that was working, everybody was happy with it and they were moving forward. Funk did not get that sense with this proposal and she felt that the Committee had not finished their job and that was why she wanted another Special[-purpose] Committee.

**Knapp** moved to a vote on Funk’s amendment.

[The **amendment** was **rejected**.]

**Freire-Fierro** proposed an amendment to add the IAPT to the new Registration Committee, as it was not currently represented.

[The **amendment** was supported by **five seconders** and was **accepted**.]

**Cantrill** wanted to ask a member of the Special Committee about the list of representatives. They had the International Organisation of Palaeobotany but did not have the International Federation of Palynological Societies, which represented a large community dealing with fossil spores and pollen and probably had more taxa in their remit than palaeobotanists. He wanted clarification on why they were not represented or how they might have been engaged in this discussion.

**Barkworth** replied that it was a failure on the part of the Committee to be aware of the group and suggested that an amendment to include it would be appropriate.

[The **amendment** was supported by **five seconders**.]

**Redhead** asked if this was an umbrella organization that also covered the International Organisation of Palaeobotany.

**Herendeen** stated that they worked closely together on some things but were distinct organizations and he was pleased that Cantrill had asked the question.

[The **amendment** was **accepted**.]

**Hawksworth** noted that the proposal had said, “at least 5 members appointed by the Nomenclature Section” followed by “and representatives from”. He asked whether the people representing those bodies would be selected by the executives of those bodies or by people who happened to be at a Nomenclature Section. He was unsure who would be deciding on the representatives and believed it should be the organizations themselves.

**Watson** replied that the idea was to have the representatives as additional to the members appointed by the Nomenclature Section. He proposed an amendment to add: “and nominated representatives from”.

**Turland** suggested rewording the amendment to “representatives nominated by”.

**An unidentified delegate** asked if this would be an editorial change.

**Turland** explained that it was important because it was not clear as it was written and could be ambiguous, therefore it was useful, not just editorial.

[The **amendment** was supported by **five seconders** and was **accepted**.]

**Knapp** asked delegates to read all the amendments and reflect on the changes that had been made to the original proposal before making further comment.

**Nakada** proposed an amendment that a footnote stating “successors of the organizations” be added, as it was a Permanent Committee and the name of an association might be changed.

[The **amendment** was not supported by **five seconders** and was **rejected**.]

**Gereau** stated that the proposal authorized the creation of a new Permanent Nomenclature Committee, with a mandate to create a new mechanism of unknown impact and expense. He did not think that it would be safe to extrapolate from the experience of fungal registration. He thought it would establish a new requirement for valid publication that would further slow the work of publishing scientific names and it should not be passed.

**Thiele** replied that unless the Section approved one of the other proposals, the Committee would not have the power to do what Gereau had said. It had the power to assist the design and implementation of repositories for new names and nomenclatural acts. Thiele therefore thought that the Committee was appropriate and did not have as much power as had just been represented.

**Div. III, Prop. A** was **accepted as amended**.

**Knapp** immediately opened the floor for debate on **Art. 42, Prop. B**.

**Applequist** noted that if the Section did not approve Art. 42 Prop. D, then Art. 42 Prop. B did not serve any purpose. Any institution, such as the Royal Botanic Gardens, Kew, wishing to set up a voluntary registration system, could do so. They did not require the permission of the General Committee. She felt that the only purpose for adding this to the *Code* was to allow registration of an unknown form to be imposed between Congresses, therefore she opposed it.

**Paton** disagreed. In his opinion the proposal set out a possible framework that would take the community forward towards registration. In the absence of this it would be difficult to get people who were not *au fait* with the current state of nomenclature to outline a vision of where registration was heading. He thought the proposal was important for helping to build the way towards registration. Whether Prop. D was passed or not, this proposal still served a function for clarifying what the vision was.

**Dorr** was curious about the timeframe for the proposal. He felt it was left open-ended and suggested that somebody could apply at any given time. He asked for clarification from the authors of the proposal whether there was some time element involved as to when institutions could apply to be repositories.

**Barkworth** replied that there was no specific timeframe in the proposal. Anybody could set up a repository, but they would need to be recognized as such. On the other hand, the proposal did say that there would have to be a public trial run of at least one year. People would have to have known about the repository and have tried it out so that they had evidence when asking to be recognized.

**Herendeen** added that another reason to keep it open-ended was that one existing repository could run out of funding, or the one person running it could die, or something else could happen and another repository would need to come into existence. He thought it needed to be left open for the possibility that some other organization needed or wanted to jump in later.

**Schori** wished to point out that if there was only one repository and the one person who was running it died, and registration was required, there would be a period of at least a year when nobody could publish any new names because they there would be no repository to register them in.

**Paton** replied that this would only be the case if registration was mandatory and it was not. He reiterated his earlier point that this was about setting up and allowing for possibilities to explore the area. If something were to fail, it would fail, and people would have learned in the process. The proposal allowed things to be tried.

**Barrie**, at the risk of burning through his allotted comments, wanted to respond to what Applequist had said regarding how things would be sent to the General Committee in the proposed new Art. 42.0*bis.* This would be analogous to the relationship between the General Committee and the other Permanent Nomenclature Committees. Everything would be sent to the General Committee but would end up going to the Registration Committee to be reviewed.

**Hawksworth** wanted an amendment stating that fungi should be excluded from this because they had already ruled that the decisions about what repositories were recognized were approved by an IMC and not by the General Committee. He suggested that “except for fungi” be added after “General Committee”. He went on to suggest that he would delete altogether the phrase: “The General Committee has the power to suspend or revoke a granted recognition”.

**Knapp** stated that Hawksworth had set out two amendments and they would be discussed one at a time. She asked for comments on his first amendment: including the phrase “except for fungi” after “General Committee” in the first sentence of Art. 42.0*bis*.

[The **amendment** was supported by **five seconders**.]

**Applequist** pointed out that the Section had just set up a Permanent Committee for registration that would include a representative of an international mycological organization. If the mycologists were not going to participate in an apparently forthcoming system of registration, she asked why they should have any voice in it.

**May** asked that one of the screens display Art. 42.3, regarding the procedure for the appointment of repositories to handle fungi, as he felt it would be helpful for delegates to see it.

[Hawksworth’s **first amendment** was **accepted**.]

**Knapp** moved on to Hawksworth’s second amendment: to strike the last sentence “The General Committee has the power to suspend and revoke a granted recognition”.

[The **amendment** was supported by **five seconders**.]

**Wilson** was not in favour of the amendment because she thought there must be a mechanism for dealing with a situation in which a repository was not functioning, and people should not have to wait until the next IBC to sort it out.

**Watson** noted that the “struck-off sentence” seemed parallel to what was already in the *Code* for fungi, Art. 42.3 clause (*2*): “cancel such appointment at its discretion”. He asked the proposer why he wanted to strike it out.

**Hawksworth** replied that he did not want to discourage people from experimenting. It would largely be carried out voluntarily by organizations, and the fact that they would have a big sledgehammer hanging over them might put people off from starting and continuing to operate such systems.

[The **amendment** was **rejected**.]

**Dorr** commented that, as written, the proposal implied that there would be a single institution recognized as a repository and suggested the proposal be rephrased to state “an institution or institutions” or even “consortium of institutions”.

**Knapp** asked Dorr whether he thought the Editorial Committee could be trusted to deal with that editorially or whether he was making an amendment.

**Dorr** replied that he was a trusting person.

**Knapp** moved to a vote on Art. 42 Prop. B as amended, with the proviso that the Editorial Committee would look at the first sentence of Art. 42.0 and ensure that the intent to allow more than one institution as a repository was clear.

**Art. 42, Prop. B** was **accepted as amended**.

**Knapp** moved on to discussion of **Art. 42, Prop. C**.

**Applequist** thought the proposal showed how premature the discussion was. She could imagine the problems of retrospective registration but if someone was publishing a book, she did not know what synchronous registration would look like. She thought this “completely nebulous statement” showed that the Committee was trying to get something into the *Code* when no one had a clue what it was.

**Greuter** was in favour of the proposal because he thought it illustrated ways in which registration could operate and did not preclude ways that might become more popular or easily acceptable in the long run. If registration were to become mandatory in the future, it would be as a service to the community, both to publishing authors and to users of names. He did not think the question of synchronous registration was as nebulous as it seemed. It already functioned and would probably be the way forward when journals, directly upon publication, linked into systems like IPNI. This was already the case for journals like *PhytoKeys*. He went on to say that names would be registered the moment they were published. There would be no delay between publication of the journal and registration because there would be automation inbuilt in an exchange between the publisher of the journal and the registration centre. He invited someone from IPNI to comment.

Regarding retrospective publication, Greuter said that what was available now was a kind of registration. Names were indexed after they were published, and he thought this would still be possible. He noted that the fungal community had gone the other way, registering before a name was published. This had advantages and drawbacks. One drawback was that if on publication the registration requirement was not fulfilled, the name had to be published again completely, starting from zero. The other traditional system from *Index Kewensis* and IPNI and existing indices permitted registration of names that had been published but not registered, after they had been published. Registration, he thought, would avoid the creation of names that were not validly published. He wanted to raise these points because the “fungal experiment” had taken a different direction and there was a perceived bias in favour of the fungal system, which he thought would be a pity.

**Lindon**, a content editor for IPNI, explained that the *PhytoKeys* system was not perfect, but they did submit the names to IPNI before publication, as did *Kew Bulletin*. The publishers of the *European Journal of Taxonomy* and of *PLoS ONE* had also approached IPNI about this. She noted that publishers were approaching IPNI and asking to register names with the database before publishing. They did not always get it right, but they were willing to work with IPNI to improve the process. Lindon had observed that she did not know how they found out about registration, but journals were interested in setting up this system before it was required, and they seemed to want it.

**Govaerts**, in reply to Greuter, noted that fungi also had synchronous registration. He had discussed this with Kirk. On *Index Fungorum*: a new combination could be published and instantly registered, so they had two types of registration.

**Art. 42, Prop. C** was **accepted**.

**Knapp** asked for comments on **Art. 42, Prop. D**.

**Gereau** stated that the proposal granted unprecedented new powers to the General Committee to modify the *Code* between IBCs, a precedent that he believed should not be established. He thought changes should require the approval of the Nomenclature Section at the time of the change. Furthermore, the proposal granted the power to impose a requirement that would have a disproportionate impact on taxonomists in developing countries. He felt that the proposal should not be approved under any circumstances.

**Marhold** wanted to hear from somebody in a developing country because, as far as he understood, electronic connection was the form of communication they relied on most; they did not have rich libraries for printed material.

**Fortunato** said it depended on the circumstances. In South America each country had differences in their access to the internet. There were also differences within countries, for example the access in Buenos Aires differed from the rest of Argentina. This was also true for institutions editing taxonomic journals.

**Applequist** pointed out that the General Committee certainly did not universally support this proposal. As a member, she strongly opposed it because it had never been their remit to impose new conditions for valid publication, and they were not a particularly democratic or representative body: they were not competitively elected. There were 25 members on the General Committee, 21 of whom were from western nations. Large swathes of the world were unrepresented, and it was not possible to know what burden a particular system might place on someone from Iran or Turkey or Cambodia. Although she opposed the proposal, she thought many delegates would vote for it. Therefore, she proposed an amendment to limit the ability of the General Committee to approve things that would be highly damaging. After the first sentence she wished to add: “Any mechanism approved by the General Committee must be accessible to individuals from all nations and include a means of registering names without direct internet access”. She added that if delegates found this outrageous, then there was a problem.

[The **amendment** was supported by **five seconders**.]

**Watson** could not see how anything could be accessible to all individuals from all nations and thought it completely unworkable.

**Schori** supported the spirit of the amendment, having spent a year in the Philippines where she was at a college of forestry that had no internet access for the 10 months she was there. She thought it would pose a serious barrier to botanists in many countries because electricity was not guaranteed on a regular basis. Even places like Pakistan, which had great internet access to all kinds of journals for its students, had people who could not do lab work because they did not have regular electricity.

**Freire-Fierro** said it was the same in Latin America.

**Struwe** stated that if people had no electricity, then the internet should not be the only way of registering names and this was the whole point of the amendment. She thought there must be other ways to register names. The amendment would allow a way in which people could work without power, without internet, and still be botanists, still publish names and still be active in discovering and describing new species. She added that the wording might not necessarily need to be “must”.

**Knapp** clarified that as an Article it would have to be “must”.

**Barrie** thought there was no way the General Committee could ever verify whether all nations could access things. He also pointed out that the author need not be the one to register. If someone was submitting papers to be published, obviously they had access to a source to publish their names. The publisher could register the names.

**May** noted that for fungal mandatory registration, which relied on internet access, they were not aware of anyone who had not been able to register a name. Should that be the case, it should be possible for them to write a letter to one of the repository curators who would be happy to carry out the registration for them. Very few names of fungi published since registration had been introduced were not registered.

**Paton** wished to propose an amendment to the amendment. He liked the second clause because he thought it would help people allay certain fears, but the first part–“must be accessible to individuals of all nations”–would be impossible to monitor. He proposed that this first part be removed, but the second part kept.

**Applequist** asked if Paton would accept the modification: “must not refuse registrations from individuals from any nation”.

**Paton** did not accept the modification, noting that it raised something that questioned everything else in the *Code*: that the *Code* was open to everyone.

[The **amendment** to the **amendment** was supported by **five seconders** and was **accepted**.]

**Greuter** thought that some of what had been discussed pointed to a “difficulty of principle” that the Section had in its relationship with the General Committee. The Section should stop considering the General Committee as some kind of small, ugly dwarf generated by itself and without any function that involved decisionary power. The General Committee represented the Section between the Congresses. It was not a by-product generated by the Section but had a sistership relation to it. Nomenclature in many cases needed representation between Congresses and the Section would be well advised in taking the role of the General Committee seriously as a democratic institution, which was elected at the Section meetings.

Greuter felt that in a rapidly changing world the need for registration would become even greater. He supported the amendment as far as its second half was concerned. Names handed in by mail or by courier or whatever could be registered, and this had been the case in the trial registration run in Berlin preceding the St. Louis Congress [1999] and would hopefully be the case in registration centres in future. In the future he believed that almost 100% of registration would occur via the internet and people would forget the “Stone Age” approach.

**Kirk** noted that a proxy service already existed in fungal registration. None of the repositories allowed bulk uploading of names to receive a series of identifiers, so he offered an unofficial service: if somebody sent him a spreadsheet with 100 new names, he would instantly assign 100 identifiers and return the updated spreadsheet within a couple of minutes. The system they operated was not rocket science, it was very simple.

**Thiele** called the question on Applequist’s amendment.

[The Section voted **to vote**.]

[The **amendment** was **accepted as amended**.]

**Levin** wanted to address the concern that had been expressed regarding the burden that registration might put on people from developing countries. He noted that researchers in developed countries needed access to the work carried out in developing countries and having a mechanism of registration would increase the impact of that work because it allowed it to be seen more readily.

**Knapp** noted that having a mechanism of registration had already been discussed.

**Levin** apologized for jumping ahead a bit and recognizing the implications of adopting the proposal.

**Hawksworth** proposed an amendment to add at the beginning: “subject to approval by the appropriate international organization at the next IBC”, and at the end “by subsequent International Botanical or other appropriate Congress”. He thought that if the phycologists wanted to adopt registration, they should be allowed to do it without any reference to the next IBC.

[The **amendment** was supported by **five seconders**.]

**Barrie** opposed the amendment, stating that it was not appropriate to give other organizations the authority to change the rules of the *Code* as determined by the Nomenclature Section of an IBC.

**Watson** added that both the General Committee and the Registration Committee had representatives of the international committees mentioned and this amendment was not required.

**Cantrill** asked to hold the proceedings as he wished to confer with Herendeen who was seated next to him.

**Herendeen**, having conferred with Cantrill, spoke against the amendment. They thought it could open the door for the “European Palaeobotanical Organization”, for example, to make such a decision. There was nothing in it that would regulate the scope or universality of the specialist international congress. A small group could make a decision for all of palaeobotany and palynology.

[The **amendment** was **rejected**.]

**Saarela** asked the proposers to clarify whether Art. 42 Prop. D meant that if the General Committee approved registration as a requirement for valid publication it would need to be ratified by the next IBC, so the earliest it could come into effect would be 2023.

**Watson** replied that if the General Committee proclaimed registration as being mandatory for a particular group, then that would be a requirement for valid publication from the time the General Committee report was published. A subsequent IBC could overturn this. It would have no effect on the names registered, because they would still be validly published.

**Barkworth** added that the General Committee would also have the power to suspend the mandatory requirement, so it would work both ways. It would not do this until after it had gone through the Registration Committee, so it would not be done on a whim.

**Middleton** was puzzled as to the need for unseemly haste and thought it was a bad idea to allow the General Committee to allow mandatory registration between Congresses. He thought the system functioned fine as it was, and voluntary registration seemed to be a good idea for new names and nomenclatural acts. However, he could not see why the Section would wish to institute something between Congresses when it could just as easily be done after six years of testing. This was not a particularly long time in the history of taxonomic botany, mycology, phycology etc. and it seemed to him a poor idea to rush a system through. It could be mandated in six years’ time if people thought it was a good idea.

**Govaerts** pointed out that six years represented 50,000 vascular plant names.

**Funk** felt that giving up the right to make decisions on changing the *Code* to anyone else was a “slippery slope”. She spoke as a member of the General Committee and, while she did not think they were a bunch of terrible people sitting in the corner, she did think that they were a select group. Election to the Committee was not contested. Funk referred to zoological nomenclature, which was decided by committee and did not have broad-based support from the zoological community. The botanical *Code* on the other hand always had strong support from the community because the community made the decisions. The community gathered every six years to make decisions and this was a critical part of how botanical nomenclature was done. Funk added that rejecting the proposal would not prevent people from registering 50,000 names in six years. It would allow and even recommend that people register names where possible, so she thought Govaerts’s comment was unfair.

**Kirk** objected to Funk referring to the “botanical *Code*” and wished to point out the disproportionate representation in the Section of delegates who worked on organisms other than plants. This was also true of geographical representation. He wanted to respond to the delegate who said that the IPNI system was working and asked how much that delegate paid the Royal Botanic Gardens, Kew for the service. He stated that IPNI was supported by Kew and working because of Kew, but the politics in the UK could result in the disappearance of Kew before the next IBC. He wondered what the botanists would do if that happened, and urged that registration be set up as soon as possible, as six years was “a lifetime” in the 21^st^ century.

**Wilson**, speaking as Secretary of the current General Committee, did not see a problem with accepting the proposal, as she did not think the community would be ready for registration in six years. Most groups would take at least six years to come up with a coherent plan on how they would do the registration and put registration centres in place. In practice, therefore, she did not think it would be an important consideration. She agreed that the ultimate authority for final approval had to be the Nomenclature Section.

**Knapp** asked if someone from the mycological community could tell the Section how long voluntary registration had been in place before it was made mandatory.

**May** replied that it was exactly four years.

**Middleton** stated that most of the 50,000 species that may be described over the next six years would be described from the tropics: developing countries that would be most burdened by mandatory registration. He also asked what would happen if the mechanism for a repository stopped working and was wound up by the General Committee: there might be a period where no one could register new taxa. He asked if someone from the Special Committee could explain what would happen in this circumstance.

**Barrie**, who was on both the General Committee and the Special Committee, said there was no possibility of this happening. The General Committee would not approve a system that was not robust enough and if one system failed there would have to be another to take its place. There would not be a single registration repository and there would have to be a back-up so that people were not inconvenienced or put in a position where they would be unable to register names once it became mandatory. There would be an experimental test period to assess problems and work out how to address them.

**Freire-Fierro**, as an Ecuadorian representative of the Latin American Botanical Society, opposed the proposal for exactly the reason Kirk had given: if funding for a repository disappeared, what would happen with this “forced requirement”? She thought it was working fine as it was and did not think there should be a requirement for registration in order to validly publish a name.

**Gandhi** wished to point out that IPNI was not solely supported by Kew and that since 1999 there had been active support from Harvard University. In addition, he said that most of the 50,000 names that would be registered from developing countries would be authored in association with researchers in the West. Cacti and orchids from developing countries were authored by westerners.

**Marhold** noted that there had been comments as to whether it was technically possible to set up registration and yet the mycological community had already shown that it worked.

**Knapp** reminded the Section that the discussion was about the proposal, not registration in general.

**Saarela** asked if it would be possible for the community to ratify a decision of the General Committee between Congresses.

**Knapp** drew attention to the rules laid out in Div. III as was currently in the *Code*: everyone who turned up at a Nomenclature Section was eligible to cast a vote. There was no membership *per se*: it was a body that was convened, and all the Permanent Nomenclature Committees, including the General Committee, represented the Section and worked on its behalf between Congresses.

**Turland** noted that the Section had no formal authority after the meeting was closed.

**Knapp** joked that the Section had no formal authority after the plenary session of the IBC either: “we all disappear…into the dust, like into an orchestra pit, and it closes over the top of us.”

**Saarela**: “So the answer is no?” [*Laughter*]

**Cantrill** called the question and the Section agreed to vote on the proposal.

[The Section voted **to vote**.]

[The vote on the proposal by show of hands did not clearly reach the 60% majority required and a **card vote** was requested.]

**Knapp** expected 100% correct cards this time: if not, there would be no tea!

**Turland** warned delegates not to put a number other than 7 in the box: “If you do, we’ll track your DNA and we’ll…Well, you don’t want to know.” [*Laughter*]

**Art. 42, Prop. D** was **rejected** based on the **card vote** (306 yes: 239 no; 56.1% yes)

[*A short informal discussion ensued*.]

**Redhead** asked at what point the Congress itself approved the recommendations of the Nomenclature Session.

**Knapp** explained that a resolution was put to the [closing] plenary session of the IBC, which could overturn all the work that Section had done if people were so frivolous as to do that.

**Redhead** said he was not suggesting they would be so frivolous as to do that but wished to point out the technicalities of the whole process.

#### Article 46 (continued)

**Art. 46, Prop. J** (49: 7: 7: 0) and **Prop. K** (1: 41: 23: 0)

**Turland** explained that these proposals had different authors so, technically, did not form a set but they addressed a similar issue and the Rapporteurs had discussed them together. They both addressed a situation found especially in 19^th^ century protologues, where the accepted name first appeared without author ascription and then appeared again with ascription in a list of synonyms or in a synonym position. Prop. J solved the problem by amending Art. 46.3 so that an author citation associated with a synonym would not constitute ascription of the accepted name. On the other hand, Prop. K was more drastic and would remove this notion entirely, considering it redundant, although the Rapporteurs were not quite certain that it would be redundant.

**Art. 46, Prop. J** was **accepted** and **Art. 46, Prop. K** was **rejected**.

**Knapp** suggested that the Section break for tea and asked that anyone wishing to serve on the Permanent Registration Committee should see Funk, Secretary of the Nominating Committee.

[*The Section broke for morning tea*.]

**Art. 46, Prop. L** (12: 44: 6: 0), **Prop. M** (12: 34: 17: 0) and **Prop. N** (11: 42: 11: 0)

**Turland** stated that there was a certain amount of interdependence among these proposals. Prop. L would be redundant unless both Prop. M and Prop. N were rejected, but it had received the highest number of “no” votes in the mail vote. Prop. M and Prop. N could be discussed first if the Section felt there was any value in retaining Prop. L as an alternative. The intention of Prop. L was to extend the application of Art. 46.4, which currently only applied to binary designations (species names).

**Nakada**, speaking as the author of the proposal, felt that all provisions for binary combinations should also be applied to ternary combinations if there was no concrete reason not to do so.

**McNeill** agreed with the principle and thought the negative votes in each case were because people were unhappy with the precise wording. He proposed an amendment that, in principle, the provisions of Art. 46.4 be applied to all combinations and that the Editorial Committee be required to look at the best wording to implement this.

**Turland** decided that there was some confusion and, as he and the Vice-rapporteur had understood it, the proposal was to extend Art. 46.4 so that it did, indeed, apply to all combinations and designations at all those ranks, which was the same as the principle that McNeill was proposing. He asked McNeill if the amendment was necessary.

**McNeill** replied that he proposed the amendment due to the high negative vote, which he attributed not to distaste for the principle, which he thought was an excellent one, but to concern that the wording was not the best. He was suggesting that Section approve the suggestion in principle and refer the wording to the Editorial Committee.

**Turland** assured McNeill that if the Section liked the proposal it should be accepted as it was on the understanding that the Editorial Committee would, as always, make the wording beautiful and perfect.

**McNeill**, on that assurance from the Rapporteur-général, withdrew his amendment.

**Gereau** thought the effect of the proposal was desirable and that it could be properly edited. However, he thought that Prop. M and Prop. N covered the same ground more thoroughly and clearly and should be preferred, rather than trying to edit Prop. L.

**Art. 46, Prop. L** was **accepted**.

**Turland** pointed out that **Art. 46 Prop. M** went further than Prop. L and there were five Examples associated with it, which would be handled by the Editorial Committee. However, the core of the proposal was not purely editorial: it applied the rule to uninomials and combinations, and replaced the word “attributed” with “credited”. The Section had seen an example of this wording earlier because the proposers preferred to reserve the term attribution for the authorship that was treated as correct under the rules for a name.

**Knapp** asked how Section had dealt with that proposal.

**Turland** stated that the earlier case was Rec. 23A Prop. A.

**Monro** confirmed that Rec. 23A Prop. A had been sent to the Editorial Committee.

**Turland** suggested that the Section should do the same thing with this proposal with respect to “credited” versus “attributed” and treat it editorially. Having accepted Prop. L, the question now was whether to go further and apply the rule to uninomials as well as combinations.

**Greuter** supported the proposal, noting that it reflected current practice.

**Soreng** pointed out that one of the Examples [regarding *Andropogon drummondii*] was incorrect because Nees was now understood to have been the author of some names within Steudel’s publication. Steudel accepted Nees’s manuscript names, and acknowledged Nees in the introduction [*Synopsis plantarum glumacearum* 1: vii. 1853].

**Turland** asked that Soreng send him an e-mail regarding this for the attention of the Editorial Committee.

[Steudel (Syn. Pl. Glumac. 1: 393. 1854) published the name *Andropogon drummondii*, whereas Nees’s manuscript contained “*Sorghum drummondii*”, hence the Example in the *Shenzhen Code*, Art. 46 Ex. 27, accords with Art. 46.4.]

**Art. 46, Prop. M** was **accepted**.

**Turland** introduced **Art. 46 Prop. N**, noting that it went even further than Prop. M in ruling that orthographical corrections were to be disregarded.

**Gereau** was not sure this was necessary, but felt it was useful to point out that disregarding minor orthographical corrections when validating names not previously validly published should be permitted, so he supported the proposal.

**Greuter** was not sure this proposal reflected current practice and it was not clear what “orthographic corrections being disregarded” should mean: whether a correction should be disregarded so that the original spelling was used, or whether the fact that there had been a correction should be disregarded so that the corrected spelling was used. Either way, he felt it was too restrictive and did more harm than good. He opposed the proposal.

**Herendeen** agreed with Greuter but wondered whether the proposer meant “orthographical errors being disregarded”, which might be clearer.

**Sennikov**, as one of the authors of the proposal, noted that “errors” were not the same in these situations as “corrections”. The Example gave two orthographical variants, both of which were correct, and no one could say that either was an error. The idea behind the proposal was that it did not matter if the validating author had made any corrections of the spelling, so “orthographic” could perhaps be replaced by “corrections in spelling” or something similar.

**McNeill** noted that now he had realized the two names were correct, he was not happy with the proposal. One would adopt a different name, correct, from the one that was orthographically correct but not validly published. He did not think this was a good idea and felt it dealt with a very specialist situation. He doubted that it reflected current practice and was inclined to vote against it.

**Gandhi** provided a comparable example: he had come across the genus name “*Eritrichum*” that was not validly published and later it was validated as “*Eritrichium*”. If this proposal were to be followed, only the validating authorship would be cited, whereas in current practice “ex” authorship was used.

**Barrie** called the question.

[The Section voted **to vote**.]

**Art. 46, Prop. N** was **rejected**.

**Art. 46, Prop. O** (3: 4: 58: 0), **Prop. P** (2: 8: 55: 0) and **Prop. Q** (2: 8: 53: 2) were **automatically** sent to the **Editorial Committee**.

**Art. 46, Prop. R** (5: 54: 5: 0) and **Prop. S** (3: 52: 9: 0) were **rejected** based on the **mail vote**.

**Art. 46, Prop. T** (19: 35: 10: 0)

**Turland** noted that this proposal sought to make explicit what was already implicit in Art. 46: namely, if authors cited other persons’ names followed by “ex” to precede theirs, and both Art. 46.2 or Art. 46.5 ruled that the correct author citation was different, Art. 46.10 would not override those other two Articles. This raised the question as to whether Art. 46.10 had any function as a rule. If the proposal was accepted the Editorial Committee would consider whether Art. 46.10 should be a Note or Recommendation rather than an Article.

**Art. 46, Prop. T** was **rejected**.

#### Recommendation 46A

**Rec. 46A, Prop. A** (13: 49: 3: 0) and **Prop. B** (2: 60: 3: 0) were **rejected** based on the **mail vote**.

**Rec. 46A, Prop. C** (7: 44: 13: 0) was **automatically** sent to the **Editorial Committee**.

**Rec. 46A, Prop. D** (11: 44: 9: 0)

**Turland** noted that this proposal would change the typography of author citations in Rec. 46A Ex. 3 and throughout the *Code* to match that of the standard forms given by the International Plant Names Index (IPNI), i.e. without spaces. The mention of IPNI in Note 1 would be retained, but that of *Index Fungorum* would be deleted, because that index provided standard forms containing spaces. The same considerations for Art. 46A Prop. A and Prop. B applied and those had been rejected in the mail vote. This proposal was a revision of Rec. 46A Note 1 to remove *Index Fungorum*. The implication was that IPNI would provide these abbreviations, but *Index Fungorum* would not.

**Greuter** proposed an amendment to leave the wording as it stood but add after the “IPNI”, in parentheses, “but disregarding the elimination of spaces”.

[The **amendment** was supported by **five seconders** and was **accepted**.]

**Garland** asked what the effect of this Note was; it merely seemed to be informative.

**Knapp** replied that Notes *were* informative, and that the Section was discussing a note in a Recommendation that did not have to be followed anyway.

**Garland** asked, therefore, why the Section was debating such fine points of wording.

**Thiele** called the question.

[The Section voted **to vote**.]

**Rec. 46A, Prop. D** was **rejected**.

#### Recommendation 46C

**Rec. 46C, Prop. A** (17: 41: 6: 0) and **Prop. B** (13: 45: 4: 2)

**Turland** explained that these two proposals sought to standardize author citations, so that when citing a name jointly published by two authors, only the ampersand symbol [&], not the word “et”, should be used between the authors. On the other hand, when citing a name jointly published by more than two authors, the first author should be followed only by “et al.”, not “& al.”. The *Code* currently recommended use of either “et” or the ampersand in both cases. The proposers used the two methods to differentiate between citing two authors and citing more than two authors, but they did not explain why such differentiation may be desirable. Turland went on to note that if Prop. B was accepted, the *Code* would have to follow its own recommendation and replace “& al.” with “et al.” throughout. The Rapporteurs had noted that, considering that the ampersand is essentially a form of the word “et”, this seemed, like the use of spaces and author citations, to be merely a matter of typography.

**Schori** noted that this was not a problem for people who were fluent readers of English and knew some Latin, but she had come across cases of manuscripts written by non-native English speakers where people thought that “et” and “al.” indicated people’s names.

**Groom** noted that for anyone who had ever tried to combine lists on computers, it made much more sense to have one standard way of doing stuff instead of multiple standards.

**Gereau** stated that this was only a Recommendation, which gave two perfectly reasonable choices and which people were free to use. He felt it was silly to be adjusting it and it should be left as it was.

**Geltman** opposed the proposal because he thought that in several countries and cultures the ampersand was less well known.

**Funk** asked if she could call the question.

**Knapp** refused, saying she would have to recognize Funk and she was about to invite de Lange.

**De Lange** called the question. [*Laughter*]

**Knapp** reminded delegates that they could not just shout, “Call the question!”; they had to raise their hand and be invited to speak.

[The Section voted **to vote**.]

**Rec. 46C, Prop. A** and **Prop. B** were **rejected**.

#### Recommendation 46D

**Rec. 46D, Prop. A** (6: 57: 1: 0) and **Prop. B** (3: 56: 5: 0) were **rejected** based on the **mail vote**.

#### Article 48

**Art. 48, Prop. A** (2: 35: *29: 0)

**Turland** said the Rapporteurs had thought this proposal unnecessary because the proposed new Note did not add anything that was not already clear from Art. 48.1. However, the Example could be helpful. As this was a Note, he did not want to automatically refer it to the Editorial Committee.

**Gereau** proposed that the proposal be sent to the Editorial Committee and the Example considered for inclusion under Art. 48.1.

[The **proposal** was supported by **five seconders**.]

**Turland** added that delegates should understand that if the Editorial Committee determined the Note not to be useful, it would not be included in the *Code*.

**Art. 48, Prop. A** was sent to the **Editorial Committee**.

#### Recommendation 50E

**Rec. 50E, Prop. A** (6: 52: 3: 0) and **Prop. B** (2: 50: 8: 0) were **rejected** based on the **mail vote**.

[These **proposals** were **reintroduced** for discussion upon a proposal from **May**, who was supported by **five seconders**.]

**May**, speaking as an individual and not representing the Nomenclature Committee for Fungi, found that the use of the recommended colon [:] in the author citation of a sanctioned name was extremely confusing. This occurred across nomenclature databases, especially when there was a mixture of different biota in one database. He thought nobody outside of mycology understood the citation and many within mycology did not understand it either. For other forms of names that were protected under the *Code*, for example through conservation, that act was attached to the name that had been conserved. In the case described under this proposal, the sanctioned name was the one that was meant to be designated, but if Fries or Persoon was naming a new combination there was confusion as to whether the colon was “attached” to the basionym or not. The proposer had pointed out that there was still a practice of citing the sanctioning work as the original place of publication and not the original work. This proposal sought to recognize that the name had been sanctioned but bring it into line with the use of “nom. cons.” and use the abbreviation “nom. sanct.” to indicate that a name appearing in the sanctioning works had been sanctioned.

**Schori**, speaking on behalf of her mycological colleagues at the USDA, said that they were not in favour of this. They thought the indications of Fries and Persoon names were well understood within the relevant community and they were not in favour of using “nom. sanct.”

**Hawksworth** noted that there was a major problem with the situation as it currently stood. The people at USDA might understand it but, as an editor of journals, many mycologists did not understand it. Although it went into the *Code* in 1981 it was not used in a lot of databases. It was not used in the *Dictionary of Fungi* or *Index Fungorum* because of the confusion it caused. He felt the proposal was a logical way to retain the protection and eliminate confusion.

**Greuter** was not against the proposal, but was uneasy with the Latin translation *nomen sanctum*, “holy name”, for sanctioned name. [*Laughter*] He proposed a friendly amendment to delete the portion in parentheses, because there was no reason to have a Latin equivalent. [The **amendment** was **accepted** as **friendly**.] Greuter went on to ask what the Committee for Fungi thought of the proposal.

**Turland** replied that the Nomenclature Committee for Fungi did not support either Prop. A or Prop. B and voted 6 “yes” to 11 “no” with one abstention on both proposals.

**McNeill**, speaking as a non-mycologist who had tried to understand the principle behind the colon citation and had found fundamental illogicalities in it, was concerned that the proposal did not have the universal support of the mycological community. He proposed an amendment to provide two alternative ways of indicating a sanctioned name, either by the use of the colon citation or via the abbreviation “nom. sanct.”.

[The amendment was **accepted** as **friendly**.]

**Rec. 50E, Prop. A** was **accepted as amended** and **Prop. B** was **automatically** sent to the **Editorial Committee**.

#### Article 52

**Art. 52, Prop. A** (53: 7: 5: 0), **Prop. B** (49: 5: 10: 0) and **Prop. C** (50: 9: 6: 0)

**Turland** explained that these proposals sought to clarify what constituted “citation of the name itself”. **Prop. A** would exclude *pro parte* citations of the name.

**Mabberley** felt that this was a welcome improvement.

**Art. 52, Prop. A** was **accepted**.

**Turland** stated that **Prop. B** would add another note to Art. 52 allowing citation of a name to be effected by unambiguous reference to it. The Rapporteurs were concerned whether this should be a Note or an Article, because there was a distinction in the *Code* between unambiguously referring to something and actually citing something. It was possible to indicate something without citing it, for example a type or a basionym. The Rapporteurs had suggested that converting the Note to an Article could allay such concerns. The proposed provision was desirable because it would prevent many names currently regarded as illegitimate from being reinterpreted as legitimate. Turland proposed a friendly amendment to convert the Note to an Article.

[The **amendment** was **accepted** as **friendly**.]

**McNeill** said that as currently worded the Article could be open to misuse. He proposed a friendly amendment to add the word “exact” before “diagnostic phrase name” because it was quite common for the diagnostic phrase name to be adopted and then some small addition made, which could imply that it was not a direct citation of it.

[The **amendment** was **accepted** as **friendly**.]

**Wiersema** pointed out that if the meaning of “citation” in Art. 52.2 was changed, it should not be used if it had another meaning elsewhere in the *Code*. He suggested the word “citation” may have to be changed in Art. 52.2.

**Sennikov** proposed that a simpler solution would be to incorporate the words “unambiguous reference” straight into the text of Art. 52.2(e). The Note would then stand as an explanation of how this unambiguous reference may be effected. He also had a concern about the word “exact” that had just been added, because these examples were not infrequent: when early post-Linnaean authors cited Linnaean diagnostic phrase names, often they were not reproduced exactly, so a number of situations could fall outside the wording of the provision.

**Turland** noted that he and the Vice-rapporteur thought that if the amended wording were to be accepted, then Sennikov’s suggestion would be editorial.

**Sennikov** was satisfied with this.

**Turland**, having reassessed the amendments made to the proposal, noted that he had some concerns. He wondered if the “unambiguous reference” could be an indirect or even cryptic reference, and if so, there could be some unwanted consequences: connection could be made between names that had not been imagined or intended.

**McNeill** agreed with Turland’s concerns and felt the wording should be quite tight, for example “effected by unambiguous reference…”, “…by citation of its original number or exact diagnostic phrase”. He thought a word stronger than “mention” might meet the concerns of the Rapporteur-général.

[*There followed some discussion about the best word to use.*]

**Greuter** interrupted the discussion to point out that Section were now doing editorial work.

**McNeill** reiterated that it should be clear that it was not a matter of indication but of unambiguous reference.

**Greuter** suggested that if the proposal was approved, it should be sent to the Editorial Committee with the instruction to seek a wording that would alleviate the concerns.

**Barrie** called the question.

[The Section voted **to vote**.]

**Art. 52, Prop. B** was **accepted as amended**.

**Turland** moved on to **Prop. C**, noting that it applied when a later isonym was cited. It provided some flexibility and guidance in determining whether the isonym was equivalent to the name itself or was used in the sense of a later author. He described this as a “somewhat complex” Note, but it had an explanatory function.

**Art. 52, Prop. C** was **accepted**.

**Art. 52, Prop. D** (6: 47: 12: 0)

**Turland** explained that this proposal removed what had been regarded as overkill in specifying “a legitimate generic name” in Art. 52.3: the name of a family or subdivision of a family that was nomenclaturally superfluous when published and based on the stem of an illegitimate generic name was also illegitimate under Art. 18.3 or 19.6. The Rapporteurs considered the proposer to be technically correct but were concerned about deleting the word “legitimate” because it could imply that superfluous names based on the stem of a generic name were legitimate, which was not necessarily the case, although the phrase “on account of its superfluity” would make it clear that other causes of illegitimacy may apply: those in Art. 18.3, Art. 19.6 and Art. 53 on homonyms. If the Section accepted the proposal the Editorial Committee might consider adding a Note to clarify this.

**Redhead** said that although it may be technically correct to remove the word “legitimate”, some redundancy in the *Code* for the sake of clarity was helpful so that the whole *Code* did not have to be studied again and again. If this Article was picked up and read it would lead to the correct answer. He thought that leaving the word “legitimate” made it a “crisper understanding”.

**Gereau** stated that the use of the word “legitimate” in Art. 52.3 was not redundant. It limited the application of the article to the cases that it covered, and its removal would change the meaning. He felt the proposition should be rejected.

**Art. 52, Prop. D** was **rejected**.

**Art. 52, Prop. E** (28: 21: 14: 1)

**Turland** explained that this proposal noted that the words “stem of” in Art. 52.3 were redundant, provided that the words “based on” were correctly understood to mean a name of a family or subdivision of a family formed from a generic name under Art. 18.1 or Art. 19.1, rather than the name of a subdivision of a genus that had a generic name as its basionym or replaced synonym. General Proposal Prop. J would replace “based on” with “formed from” and avoid such confusion. If Art. 52 Prop. E was accepted, the Editorial Committee would make it clear that the name formed from a generic name was the name of a family or subdivision of a family. He proposed, therefore, to refer the proposal to the Editorial Committee.

[The **proposal** was supported by **five seconders**.]

**Art. 52, Prop. E** was sent to the **Editorial Committee**.

#### Article 53

**Art. 53, Prop. A** (14: 37: 13: 0) and **Prop. B** (12: 34: 17: 0)

**Turland** noted that these were linked proposals. Prop. A added a new paragraph to Art. 53 and Prop. B added a new Note and two Examples, which would be sent to the Editorial Committee if the proposal was accepted. These proposals were not contingent on Art. 6 Prop. E but, because that proposal had been rejected, these would be more difficult to accept.

**Greuter** saw the proposal as “potentially dangerous.” There were cases in which very little was said in the protologue. He referred to Walter’s *Flora Caroliniana* [1788], in which Walter adopted many old, well-known epithets for species briefly described by him from the Carolinas, which were now known to be something else. He obviously never intended these to be names of new taxa, i.e. later homonyms. He did not cite any material for them and there were no syntypes. However, under this proposal these names would have to be regarded as later homonyms, introducing unnecessary, completely useless names that were never meant to have been published.

**Sennikov** explained that these proposals arose from the situation where it was totally impossible to decide if a certain name used in a given publication was a later homonym, a reuse, or an isonym of the name that had already been validly published. There had been quite a hot debate around this, hence his attempt to devise some procedure that would deal with the situation. Sennikov was surprised about the concern over Walter’s names because Prop. B included the *Catalpa* example from Walter, which was deemed to be reuse rather than a later homonym.

**Art. 53, Prop. A** was **rejected**.

**Turland** noted that **Prop. B** was not contingent on the just-rejected Prop. A and should be considered separately.

**Gandhi** noticed that in the proposed Ex. 7*bis* the authorship of *Allium globosum* was given as Candolle. He thought this was likely based on external evidence but, going by the title page, the authorship was Redouté.

**Applequist** pointed out that the question as to whether two names with no original material in common were synonyms or not was a taxonomic judgement, not a nomenclatural one. She thought it would be very disruptive to be able to declare names not to have been published merely because they were believed to be taxonomic synonyms.

**Art. 53, Prop. B** was **rejected**.

**Art. 53, Prop. C** (50: 12: 3: 0)

**Wiersema** explained that this was related to some of the other proposals that put a date on nomenclatural actions. In this case, it was the binding decision that two names were sufficiently alike to be treated as homonyms. The proposal would make the effect retroactive so that if a name was declared to be sufficiently like another name, it would be deemed to have been illegitimate when published.

**Art. 53, Prop. C** was **accepted**.

**Art. 53, Prop. D** (6: 56: 2: 0) was **rejected** based on the **mail vote**.

**Art. 53, Prop. E** (8: 9: *47: 0)

**Turland** noted that this proposal sought to make explicit what was implied in Art. 53.1 and Art. 53.6: that homonyms with equal priority, in other words simultaneously published homonyms, may be legitimate. However, the proposed additional wording seemed somewhat awkward and unnecessarily repetitive of what was already in Art. 53.1. The Rapporteurs had suggested that a simpler solution might be to insert a new Note after Art. 53.1. Turland, therefore, proposed an amendment to send this to the Editorial Committee.

**Govaerts** considered the **amendment unfriendly** because the wording reflected that in Art. 52.3, which was similar but dealt with superfluous names.

[The **amendment** was supported by **five seconders**.]

**Turland** confirmed that his proposal was to insert a new Note after Art. 53.1 and to amend the first clause of Art. 53.6.

**Barrie** asked if the proposal included striking out the first sentence that would have been added under the original proposal.

**Knapp** confirmed that it would.

[The **amendment** was **accepted**.]

**Dorr** was concerned about the double action of explicitly having to reject the other name. He noted that there were other instances where one name was adopted and used, and the other was not explicitly rejected.

**Art. 53, Prop. E** was **accepted as amended**.

[*The Section broke for the official photograph (Figure 1) and lunch*.]

### Thursday, 20^th^ July 2017, Afternoon Session

**Knapp** welcomed everyone back from lunch and announced that the fourth edition of *Mabberley’s Plant-Book* was being launched at the IBC. She provided details of the celebration that would be taking place at the main conference the following week. Knapp also announced that there would be a General Committee meeting during the afternoon tea-break today. She noted that everyone had been given URLs to access the General Committee reports. She was keen for people to contact Funk if they wished to serve on any of the new Committees that were being set up. Knapp wished to broaden the membership to avoid having the same people always serve on the Committees.

#### Article 54 and Recommendation 54A

**Art. 54, Prop. A** (10: 50: 2: 0) and **Rec. 54A, Prop. A** (11: 49: 4: 0) were **rejected** based on the **mail vote**.

**Turland** noted that these two proposals were linked and had been rejected in the mail vote.

**Redhead** wished to **reintroduce** the **proposals** and was supported by **five seconders** to do so. He had noted that the Nomenclature Committee for Algae had not favoured Art. 54 Prop. A, whereas the Nomenclature Committee for Fungi did. He wished, therefore, to propose an amendment to remove the words “an alga or” and leave “a fungus”.

[This was **accepted** as a **friendly amendment**.]

**Hawksworth** explained that the basis of the proposal was to bring the *Code* into alignment with what was already in the *International Code of Nomenclature of Prokaryotes*.

**Art. 54, Prop. A** was **accepted as amended**.

**Knapp** invited comments on **Rec. 54A, Prop. A**.

**Hawksworth** pointed out that having accepted the amendment in Art. 54 Prop. A, this proposal should be amended to read “algal and plant taxa”.

[This was a **friendly amendment** from the proposer.]

**Applequist** stated that this proposal had received a 77% negative vote in the mail ballot. If at least 51% of mycologists were likely to favour the former, then it was botanical and phycological colleagues who were voting against and who did not want to mess around with the zoological *Code*. She thought the Section should respect that.

**Nakada** thought “alga and plant taxa” was not adequate because there were some non-photosynthetic organisms related to algae covered in the *Code*. He proposed an amendment to add “new taxa other than fungi”.

[This was **accepted** as a **friendly amendment**.]

**Herendeen** had two questions: first, “taxa” and “names” were not precise, and he wondered if this applied across all levels. Second, as an editor of a journal who tried to follow recommendations as well as rules, he asked how this would be implemented: would he have to search zoological names to make sure an author had not used one? He did not think this was practical.

**Mabberley** thought that, the Section having just passed Art. 54 Prop. A, it would just be a matter of “editorial tinkering” with the existing Rec. 54A.

**Greuter** said that in his humble opinion this was just a political statement to the non-botanical world: a declaration of intent and of goodwill not to create other trans-kingdom homonyms that, in practice, in botany, no one would take notice of and no one would follow. While it did not do any harm and may even be followed as an example by zoologists, he thought it was completely useless.

**Levin** spoke in favour of the proposal. He noted that in today’s extensive use of electronic databases, Google searches etc., having unique names for plants and animals was beneficial.

**McNeill** stated that all discussion of this proposal had been out of order. Delegates had been talking about the existing Rec. 54A, and what was being proposed was editorial. It would be necessary to adjust the existing Recommendation to accommodate the fact that the fungi were no longer covered under it. He agreed with Greuter that the Recommendation was more political than practical and if delegates were opposed to it, they should recommend its deletion.

**Rec. 54A, Prop. A** was **rejected**.

**Art. 54, Prop. B** (30: 31: 2: 0) and **Rec. 54A, Prop. B** (34: 27: 3: 0)

**Turland** noted that these proposals formed a pair similar to the previous two proposals that had just been accepted and rejected.

**Wiersema** commented that this was moving the date from 2019 to 2025.

**Turland** confirmed that these proposals were essentially the same as the previous two but with a later starting date of 2025. Art. 54 Prop. B applied to all organisms treated under the *Code*, extending “a bacterial or protozoan name” to “a name available under either the prokaryote or the zoological *Code*”. The Nomenclature Committee for Fungi had only 50% support for it, whereas the Nomenclature Committee for Algae supported it. As for Rec. 54A Prop. B, this would become necessary upon acceptance of Art. 54 Prop. B, but would only apply as a Recommendation when the new rule took effect on 1 January 2025. The Nomenclature Committee for Fungi only had 50% support for Rec. 54B Prop. B, whereas the Nomenclature Committee for Algae supported it.

**Schori** asked if this meant that in order to know whether a name was legitimately published, one would have to find out whether there were names that were validly published and, therefore, available under another *Code*. This would mean one would have to work through the literature and interpret the *Code* for other organisms in order to determine whether the name proposed was legitimate under our *Code*.

**Hawksworth** replied that one could already find out what names were in use by consulting the *Catalogue of Life*, which was produced each year and was available online and on CD [compact disc]. There were unofficial lists of zoological names that he hoped would become generally available prior to the date of the introduction of the rule. The function of this proposal was to put a marker in for the next Congress. It would also be a gesture to the Zoological Commission that they should include something similar in their *Code*, which they would be revising during this period. If by 2023 it was not possible to check zoological names easily, the Articles could be deleted or changed.

**Watson** pointed out that the first line of Principle 1 was “The nomenclature of algae, fungi, and plants is independent of zoological and bacteriological nomenclature” and asked whether, if these proposals were passed, changing Principle 1 would be merely editorial?

**Turland** agreed that there would be a potential conflict and did not think that changing the Principles would be editorial.

**Applequist** did not believe that the *Code* ever could or should be subsumed into a global BioCode. The community did not want or need this, and it would create problems for those people who were working in biodiverse countries. She asked the Section to consider the genus *Cecropia*, which was both a tree and a moth. If someone wanted to publish a new combination or species in *Cecropia*, they would now have to check entomological databases. Hawksworth apparently had these on a CD, but people working in a tropical herbarium were unlikely to have access to that CD. She opposed the proposal.

**May** noted that if anyone wanted a CD from *Catalogue of Life*, they just had to e-mail Leiden and they would send it out to them.

**Thiele** called the question.

[The Section voted **to vote**.]

**Art. 54, Prop. B** was **rejected**.

**Knapp** asked for comments on **Rec. 54A, Prop. B**.

**Groom** was disappointed that Art. 54 Prop. B had been rejected. He pointed out that unique names for unique concepts should be provided for use by the whole biological research community. He felt that this was not being done and the least the Section could do was to pass this proposal.

**Schori** proposed an amendment to remove the date from the proposal.

[This was **accepted** as a **friendly amendment**.]

**Barrie** asked if the amended proposal now had the same wording as the existing Recommendation [Rec. 54A.1].

**Turland** agreed that the wording was the same with the addition of “prokaryote” [in place of “bacteriological”].

**Knapp** called for the vote to be taken and confirmed that delegates would be voting on Rec. 54A Prop. B, as amended: the only change being the use of the word “prokaryote” in place of “bacteriological”. She pointed out that this was merely an editorial change.

**Rec. 54A, Prop. B** was **accepted as amended**.

**Art. 54, Prop. C** (56: 5: 2: 0)

**Turland** noted that this proposal would add a new clause to Art. 54.1 allowing homonymy between generic names validly published under the *Code* and intergeneric graft hybrid names published under the *International Code of Nomenclature for Cultivated Plants* (*ICNCP*). The authors argued that because names of intergeneric graft hybrids were comparable with generic and nothogeneric names governed by the *Code*, precluding duplication between the two *Codes* was desirable. The authors noted that the proposal could be likened to the provisions in Art. 16.3 and others that prevented confusion between names governed by the *Code* and names of viruses, where names terminating in -*virus* and similar terminations were precluded. The proposal contained an enumeration of all ten graft hybrid names known to have been established under the *ICNCP*.

**Art. 54, Prop. C** was **accepted**.

**Art. 54, Prop. D** (2: 62: 2: 0), **Prop. E** (2: 59: 4: 0) and **Prop. F** (4: 56: 4: 0) were **rejected** based on the **mail vote**.

#### Article 55

**Art. 55, Prop. A** (9: 2: 55: 0)

**Turland** noted that this proposal was essentially editorial. It pointed out that the word “originally” in Art. 55.1 and Art. 55.2 was redundant. These provisions dated back to the Stockholm *Code* of 1952, where the phrase was “originally published under” in both provisions. This became “originally combined with” and “originally placed under”, respectively, in the Sydney *Code* [1983] and then both “originally placed under” in the *Tokyo Code* [1994]. Turland proposed that it be sent to the Editorial Committee.

[The **proposal** was supported by **five seconders**.]

**Art. 55, Prop. A** was sent to the **Editorial Committee**.

**Art. 55, Prop. B** (12: 1: 53: 0)

**Turland** explained that this proposal would add a Note, based on Art. 14.10, explaining how a name, as indicated in Art. 55.1 and Art. 55.2, may be used or not used. The Rapporteurs felt it was a useful addition and could be sent to the Editorial Committee. Turland thought it reflected what was explicit or implicit elsewhere in the *Code*, but rather than simply refer it to the Editorial Committee, it would be better if the Section discussed it.

**Gereau** stated that, although editorial, he felt this proposal clarified a situation that was frequently misunderstood. It had merit in itself and should be approved.

**Art. 55, Prop. B** was **accepted**.

**Art. 55, Prop. C** (3: 2: 59: 2) was **automatically** sent to the **Editorial Committee**.

**Art. 55, Prop. D** (43: 13: 10: 0) and **Prop. E** (6: 14: 46: 0)

**Turland** explained that these were linked proposals: Prop. E concerned only three Examples, which would automatically be sent to the Editorial Committee if Prop. D was accepted; Prop. D would add a provision to Art. 55 to explicitly allow a combination with a generic or species name that was a later homonym to be combined with the corresponding earlier legitimate homonym, without a change of authorship or date. It was implicit that this was anyway possible because the combination in either position was spelled the same and had the same type.

**Gereau** stated that this was a perfectly logical parallel to Art. 55.3 and it seemed like it belonged in this part of the *Code*.

**Art. 55, Prop. D** was **accepted**.

**Art. 55, Prop. E** was **automatically** sent to the **Editorial Committee**.

#### Article 56 and Recommendation 56A

**Art. 56, Prop. A** (62: 1: 2: 0)

**Turland** said that the Rapporteurs had noted that this proposal would be useful to dispel any doubts as to whether a rejected name could serve as the type of a higher-ranked name. It currently could. The second part of the Note, clarifying that a combination with a species name or generic name that was rejected was unavailable for use but may be legitimate, made the same point that Art. 55 Prop. B made with regard to Art. 55.1 and Art. 55.2, which had just been accepted. If accepted, the Example within the proposal would automatically be sent to the Editorial Committee.

**Art. 56, Prop. A** was **accepted**.

**Art. 56, Prop. B** (5: 57: 2: 0) was **rejected** based on the **mail vote**.

**Art. 56, Prop. C** (42: 16: 4: 0), **Prop. D** (47: 13: 3: 0), **Prop. F** (42: 14: 4: 0) and **Rec. 56A, Prop. A** (45: 14: 3: 0)

**Turland** explained that this group of proposals concerned terminology: replacing the word “rejected” with “suppressed” in Art. 56.3. There were then consequences in Art. 56.4 and Rec. 56A.1. Turland suggested **Prop. C** should be discussed first, and the two linked proposals would be more or less automatic depending on the outcome.

The idea of Prop. C was to propose a standard and less ambiguous label for the lists of fungal names created under Art. 56.3. The term “suppressed names” was currently in use for the entries in App. V of the *Code* and this would require a new title. The current wording of Art. 56.3, “to be treated as rejected under Art. 56.1”, would include rejection of all names for which a listed name was the basionym. The standing of the names in the current App. V and those of the lists generated under Art. 56.3 would be identical, the difference being in the process by which the respective entries were generated, with those in the current App. V resulting from the procedures outlined in Art. 56.2. Turland invited Wiersema to explain the proposal more eloquently.

**Wiersema** noted that, if the names were treated as rejected, and any name based on them was also rejected, there was no difference between these names and those in the current App. V, other than the process by which they got there. No lists of names had yet been proposed under Art. 56.3, so that issue was to be considered, together with the fact that “suppressed” was already used in App. VI for suppressed works.

**May** asked if the proposer could explain the distinction in terminology introduced by the current proposal, in the context of the paired set of proposals [Art. 14 Prop. H and Rec. 14A Prop. A] where “accepted” names had been changed to “protected” names and there was a specified difference between “protected” and “conserved” names. If there was no difference between “rejected” and “suppressed” names, he wondered why a different term should be used. He wanted delegates to consider that because the names to be treated as rejected could be generated through lists, there could be a lot of them. However, if they were the same status, he questioned why a different term would be needed.

**Hawksworth** replied that he was not sure any suppressed lists would be needed given what had been decided regarding protected lists already. The idea of the distinction was that these would be – apart from going through a different process – suppressed in favour of all names, not just in favour of names they might compete with. Whereas rejected names in the list of conserved names, for example, were rejected only in favour of the names that were conserved against them.

**Wiersema** pointed out that these names were akin to those in App. V that were rejected against all names and not to be used.

**Hawksworth** replied that there were two senses: the names that were rejected in favour of something [i.e. names were conserved against them], and then the list of rejected names [App. V].

**Wiersema** argued that the first sense would be dealt with under Art. 14. However, the names currently under discussion were under Art. 56.

**Hawksworth** said he was happy that they did not have any suppressed lists, because he did not think they were needed.

**Redhead** noted that both Art. 56.3 and the pertinent Art. 14.13 were added in Melbourne [2011] because it had been anticipated that, as fungal systematics shifted, there would be lists produced by the various committees and subcommittees. These lists, which had not yet been submitted, were at the generic level and did not make it all the way down to the species level in many cases. To clean up fungal taxonomy, Redhead still anticipated that large numbers of obscure names may be dumped.

**Knapp** asked if “dump” meant to throw into outer darkness?

**Redhead** agreed; he still thought this was a good Article and the differentiation between “suppressed” and “rejected” here, and “conserved” and “protected”, were a useful pair of terminology changes.

**Applequist** stated that no one had yet explained what the difference was between “rejected” and “suppressed”, noting that in either case it would be a long list. She said that the same proposer previously tried to convince the Section to use disruptive name changes to harmonize the *Code* with the zoological *Code*. Now he wanted the Section to de-harmonize the “MycoCode” and the *Code*, a process that she strongly opposed.

**McNeill** thought the proposer was thinking in terms of the App. IV and App. III situations, rather than the situation in App. V. However, he thought the word “suppressed” had merit in both cases. He explained that the *Code* distinguished names that were rejected under Art. 56 and therefore appeared in App. V as being rejected “utique”. In removing the Latin headings of the Appendices to the *Code*, App. V was referred to as a list of suppressed names to distinguish it from those names rejected under Art. 14. McNeill thought that an English language term should perhaps be sought to make clear what type of rejection was used. If rejected “utique” was dropped, a replacement was needed and “suppressed” might be the right one. He went on to say that he thought this was largely editorial.

**Art. 56, Prop. C** was **rejected**.

Turland asked if the proposal was “rejected” or “suppressed” [*Laughter and groans from the floor*.]

**Turland** explained that **Art. 56 Prop. D** was analogous to Art. 14 Prop. J concerning Art. 14.13, which had the same exclusion for lichen-forming fungi and those fungi traditionally associated with them taxonomically, for example *Mycocaliciaceae*. The Section had accepted that proposal so it would seem logical to accept this one also.

**Hawksworth** proposed that this be sent to the **Editorial Committee**.

[The **proposal** was supported by **five seconders**.]

**Applequist** argued that this was not necessarily editorial and that the lichenologists might accept no longer being exempted from the first provision, but not from the second provision.

**Barrie** noted that the lichenologists at the Field Museum supported this deletion, so whether it was dealt with editorially or voted upon they would be in favour of it.

**Turland** added that the Nomenclature Committee for Fungi supported Prop. D, but both lichenologists on the Committee opposed it. However, the Council of the International Association for Lichenology strongly supported it 9 votes to 0, with 2 abstentions.

**Art. 56, Prop. D** was sent to the **Editorial Committee**.

**Art. 56, Prop. E** (8: 54: 2: 0) was **rejected** based on the **mail vote**.

**Turland** advised that **Art. 56 Prop. F** and **Rec. 56A Prop. A** were necessary editorial adjustments of their respective provisions contingent on acceptance of Art. 56 Prop. C, which had just been rejected.

**Art. 56, Prop. F** and **Rec. 56A, Prop. A** were **automatically rejected**.

**Art. 56, Prop. G** (55: 6: 3: 0)

**Wiersema** explained that this proposal put a date on a nomenclatural act. This time it was an act under Art. 56 to reject a name “utique”. In this case the rejection would take effect, as with the conservation, on the date of effective publication of the General Committee’s approval, which as the Article already stated, would be authorized subject to the decision of a later IBC.

**Knapp** reminded delegates that there was an emendation not to have the hyperlink but to refer to the [Appendices] database.

**Art. 56, Prop. G** was **accepted**.

#### Article 57

**Art. 57, Prop. A** (60: 1: 2: 0)

**Turland** noted that this proposal sought to delete Art. 57.2 and associated Examples, Ex. 2 and Ex. 3. It was strongly supported by the Nomenclature Committee for Fungi. The proposal eliminated the provision that first appeared in the *Melbourne Code* when dual nomenclature for fungi was abandoned, and gave preference to teleomorph-typified names when competing with anamorph-typified names for the same taxon. Apparently nearly all mycologists favoured its removal, which would then allow priority to operate normally between such names.

**May** said that this Article had caused enormous difficulties in the Committee for Fungi: they called it the “Monty Python Article”. They were requested to reject a proposal. In the first round of voting in the Committee people accepted a proposal that the proposer wanted them to reject. He thought it would be “fantastic” if the Article were to be removed, although it had created great mirth at times in the Committee.

**Art. 57, Prop. A** was **accepted**.

**Art. 57, Prop. B** (47: 14: 2: 0)

**Turland** noted that this was contingent on rejection of the proposal [Art. 57 Prop. A] that had just been accepted.

**Hawksworth** wished to withdraw the proposal.

**Art. 57, Prop. B** was **withdrawn**.

#### Article 58

**Art. 58, Prop. A** (1: 1: 61: 1) was **automatically** sent to the **Editorial Committee**.

#### Article 59

**Art. 59, Prop. A** (7: 27: 3: 26)

**Turland** thought what was being proposed was obvious and the proposers believed that the gains from adopting this provision, in preserving familiar names or epithets, would outweigh any unintended consequences from misapplication of a combination to a different taxon. He noted that other mechanisms under Art. 14.13 also existed to resolve the underlying issue. However, the Nomenclature Committee for Fungi did not support Prop. A, with 2 “yes” votes to 6 “no” votes and 2 abstentions; 8 members of the Committee voted for a Special[-purpose] Committee to examine the matter. Turland went on to say that if the proposal was referred to a Special-purpose Committee, by 2023 the matter might be resolved anyway under Art. 14.13. The mail vote recorded 26 votes for a Special[-purpose] Committee, but Turland repeated his comment about the matter possibly being resolved by the next IBC.

**May** thought this was an issue that genuinely needed to be resolved because of the move to “one fungus, one name”. There were situations where there was a later name in a genus that was blocking the transfer of the earlier anamorph or teleomorph name that was introduced in another genus when there was another name that was heterotypic that had been introduced between the two of them. May was very supportive of the move to resolve this issue and some of the fungi involved were important plant pathogens. If the proposal was accepted, then somebody would have to go through the nomenclatural databases and amend all the author citations and change the second taxa that were described either as anamorphs or teleomorphs and make them into new combinations. If someone were to do that there would be greater nomenclatural stability and he suggested that a group of people might convene to draw up such a list; otherwise, if the proposal were accepted, the changes would dribble in over many years as people discovered situations.

May added that people introducing that second name under the rules as they applied would have typified that material on a different type. They were going against something that was fundamental by disregarding that second typification, and there could be circumstances where the second name so typified was the one that was in general use: it could be a teleomorph that was widely understood and the earlier anamorph name, which had a different type, might be found not to be conspecific. So, there could be unintended consequences. He reiterated the importance of having a group of people draw up a list so that these issues were easily identified. May did not know whether such a list would be put through the provisions of Art. 14.13 or not, but he pointed out that there would be an unknown number of situations. May asked the proposer [Hawksworth] what his thoughts were on these two issues, the large number of names that could be changed at one time rather than letting them dribble in and the possibility of non-synonymous types.

**Hawksworth** thought it would be relatively easy for the first problem to be addressed using databases. Regarding the issue of types, these would still be typified by the original name. For example, *Penicillium brefeldianum* would stay, so he did not think this would be a problem. It would be the same as the situation with illegitimate names and superfluous names: they would be typified by the name that should have been taken up and he did not see any problems with that. The types that had been made redundant in some cases might be useful for designation as epitypes if they had contained the stage not present on the original type.

**Redhead** did not think that the situation was as straightforward as Hawksworth had suggested. The two types, even though they may be similar taxa phylogenetically, could be slightly different. It was not just a matter of resolving the two types or reverting back to the earlier type for the earlier name, because they could turn out to be two different taxa. The rusts, some of which were economically important, were particularly complex because the asexual and sexual states had different names. The rules for pleomorphic fungi had changed through the various *Codes* and these rules had been applied but were sometimes interpreted to say that an author had created a new name when they had not explicitly done that. Sometimes, the author had explicitly said it, so it got very complex. The members of the Committee for Fungi were uncomfortable giving *carte blanche* to this conversion or were approaching it with caution. They thought that it was a clever idea but were apprehensive and could not fully explain the consequences, which was why they had voted to refer it to a Special[-purpose] Committee. Redhead formally proposed, therefore, to send this proposal to a Special-purpose Committee.

[The **proposal** was supported by **five seconders**.]

**Wiersema** wondered how this would play out because this Special-purpose Committee would depend on what happened with the Div. III ideas governing Articles of the *Code* that pertain solely to fungi.

**Knapp** thought that if a Special-purpose Committee were to be established to investigate this proposal, then it would be established. It was important that the issue was resolved in a way that delegates felt was aiding the science, which the Section hoped to facilitate.

**Art. 59, Prop. A** was sent to the newly established **Special-purpose Committee on Pleomorphic Fungi**.

**Knapp** proposed that this should be called the “Special-purpose Committee on Rust”. [*Laughter*]

#### Article 60

**Turland** said he remembered that when they were editing the *Melbourne Code*, Barrie had said that reaching Art. 60 was a bit like crossing into Mordor. [*Laughter*]

[*Mordor, with Mount Doom, elves and orcs mentioned below, are references to J. R. R. Tolkien’s Middle-Earth fantasy writings, epitomized in the Lord of the Rings trilogy: https://en.wikipedia.org/wiki/The_Lord_of_the_Rings*]

**Knapp** noted that it was almost teatime and proposed that the Section should break for tea early and have an extra 15 minutes for tea. [*Cries of “yes” from the floor; the proposal was accepted.*] Knapp stated that Section would break now and would cross into Mordor at four o’clock.

[*The Section broke for afternoon tea*.]

**Knapp**, while waiting for the Section to reconvene after the break, reminded delegates about the deadline for handing in proposals from the floor. She also wished to state, for the record, that she had looked in on the General Committee meeting and they were not gnome-like, nor were they sitting in leather armchairs smoking cigars, they were just normal people. [*Laughter*]

**Knapp** made an announcement regarding transport arrangements for delegates who would be attending the IBC, before reconvening the meeting and entering Mordor.

**Art. 60, Prop. A** (2: 58: 4: 0) was **rejected** based on the **mail vote**.

**Art. 60, Prop. B** (59: 12: 4: 0)

**Turland** noted that this proposal, together with Art. 60 Prop. C, would modify Art. 60.5. There was also a related proposal, Art. 60 Prop. D. These three proposals would be treated separately. Prop. B specified that the “modern practices” referred to in Art. 60.5 were only typographical ones. It would standardize all transcription of the Greek diphthong *ey* [ευ] (*epsilon-upsilon*) to *eu.* The use of *ev* instead of *eu* would be a correctable error.

**Gereau** said the addition of “typographical” was clarifying, but consistently correcting *epsilon-upsilon* to *eu* instead of *ev* could lead to some erroneous conclusions. The relatively small number of cases, such as *Mezonevron*, which was in the discussion with the proposal, could and should be handled by conservation and rejection. Gereau thought that the last part of the proposal was overkill and should not be accepted.

**Applequist** thought there were problems with both suggested emendations. There were some names that were traditionally spelt with an *ev* and it would be disruptive to force them to change. The addition of “typographical” was not merely a clarification, but a restriction: the sentence concluded with “in conformity with modern nomenclatural usage”, which could simply mean the custom of the modern era and not only typographical practices.

**McNeill** agreed with Applequist that “typographical practices” changed the meaning and was restrictive but thought that the origin of the Article was entirely related to typography. There had been strong controversy as to how it should be interpreted: whether it was only applicable to works in which those letters were used interchangeably, or whether it also applied to works where those letters had been used in any other way incompatible with modern practices. He explained that this was the reason for Art. 60 Prop. D, because if Prop. B was accepted then it would deal with a specific situation where there had been interchangeable use of those letters. The hole created by inserting “typographical” would then be plugged by Prop. D, assuming it would all be put together appropriately by the Editorial Committee. He supported this proposal but only if Prop. D was also supported.

**Greuter** wished to explain why changing *ey* to *eu* had been singled out as a special case. Transcription in conformity with modern nomenclatural usage was not in this case univocal. There was a strong French tradition, which English-speaking persons ignored [*Laughter*], to transcribe Greek words with *ey* to *ev*, such as *névrose* for neurosis, or *Névroptères* for the *Neuroptera*. If relying on modern nomenclatural transcription usage, the *ey* diphthong had to be singled out and ruled specifically. If this was not the case, it could result in non-correctable and perhaps undesirable names and epithets: for example, the now-conserved *Evonymus*, which had used non-traditional *ev* transcription.

**Art. 60, Prop. B** was **accepted**.

**Art. 60, Prop. C** (47: 12: 5: 0)

**Turland** noted that Prop. C would further expand Art. 60.5, standardizing usage of the letter *i* as a semi-vowel in Latin-derived words to the letter *j.* The proposers were spelling out what had traditionally been taken for granted for Latin-derived names and epithets, although no consistent tradition existed for Greek, hence the explicit exclusion of names and epithets of Greek origin.

**Gereau** stated that the example invoked in the explanation of the proposal was unnecessary. In the protologue of *Brachypodium japonicum* the *i* and *j* were used interchangeably, no differentiation was made between them, therefore this was already correctable. The number of cases of *i* being used as a semi-vowel followed by another vowel was extremely low and easily covered by conservation and rejection. He did not think a new rule was needed to cover this.

**Garland** said that there were no Latin diphthongs that started with the letter *i*, so he did not understand the part of the proposal where it said, “another vowel to form a diphthong”. He proposed an amendment to eliminate the words “to form a diphthong”.

[The **amendment** was considered **unfriendly** and was not supported by **five seconders**].

**Garland** went on to say that his understanding was that there were Latin diphthongs ending with the letter *i* but not beginning with the letter *i.* For instance, *ei* would be a Latin diphthong, as in “*Buddleia*” [*sic*]: the diphthong would be the *ei*, not the *ia.* These were two separate syllables: *ei*, *a*, not *e*, *ia*.

**Greuter** replied that when speaking of cases of *i* used as a semi-vowel it was equivalent to it taking the place of a consonant, and the consonant formed a syllable with the vowel that followed, as in *maior*, “major” and *Ianus*, “Janus”. According to his understanding of phonetics, it was quite correct.

**Art. 60, Prop. C** was **accepted**.

**Art. 60, Prop. D** (33: 16: 15: 0)

**Turland** explained that Prop. D was independent of the success or otherwise of the previous two proposals, but was only critical if these were accepted, which they had been. The thrust of Prop. D was to remove the “any way incompatible with modern practices”, entirely out of the context of a work where the letters *u*, *v* or *i*, *j* were used interchangeably. Even if Prop. B and Prop. C failed, which they had not, it could no longer be argued that Art. 60.5 applied only to works with interchangeable use of these letters. If all three proposals were accepted the Editorial Committee would integrate them appropriately.

**Gereau** stated that in Art. 60 Prop. B the phrase “in any way incompatible with modern practices” was deemed overly vague and had to have “typographical” added to it. With this proposal the same phrase was purported to be some advance. Gereau thought it was a vague phrase that was nearly impossible to interpret. It was not a useful phrase in the *Code* as it could not be defined.

**McNeill** replied that the reason for the insertion of “typographical” had been to ensure that that article related only to works in which *u* and *v*, and *i* and *j* were used interchangeably, whereas Prop. D reflected what Applequist had referred to earlier, namely the fact that putting in “typographical” narrowed the scope of the Article. This proposal would restore the full scope in an unequivocal manner. Previously he thought it had been somewhat equivocal whether this could be applied generally or only in a case where these letters had been used interchangeably.

**Greuter** felt that this proposal would widen unduly the scope of application of the Article. *Hieronyma*, for example, which had been spelt in various ways [e.g. “*Hieronima*”, “*Hyeronima*”], but not always in conformity with modern transcription, would be an *i* for the first *i* and a *y* for the second one. He asked McNeill if these would now be correctable.

**McNeill** replied that any such cases should be standardized in conformity with modern nomenclatural usage, because the community did not want to change what they were currently doing. The difficulty arose when different cultures used different things, but they were a minority.

**Art. 60, Prop. D** was **accepted**.

**Art. 60, Prop. E** (7: 0: 57: 0) was **automatically** sent to the **Editorial Committee**.

**Art. 60, Prop. F** (60: 1: 3: 0), **Prop. G** (59: 1: 4: 0), **Prop. H** (50: 0: 13: 1) and **Art. 20, Prop. B** (44: 4: 14: 0) [*Deferred*]

**Turland** explained that this set of proposals were from the Nomenclature Committee on Fossils. Prop. F was the core proposal and, if accepted, Prop. G and Art. 20 Prop. B would be editorial. Prop. H was an Example, so would be automatically sent to the Editorial Committee if Prop. F passed. All four proposals were written by and unanimously supported by the Nomenclature Committee on Fossils.

**Herendeen** noted that [Alexander] Doweld had made the Committee aware that there were numerous generic names that were hyphenated when originally published. They were not so used, in that the hyphen had been lost, so nobody was aware that these hyphens had even existed originally. Doweld proposed conservation of all these names [with conserved spelling] without the hyphen, but some of these names had other problems. The Committee had been made aware of more hyphenated generic names than Doweld had mentioned in his original proposal and they were concerned that there were even more still to be found. Rather than conserve all these names one by one, the proposed solution was to regard the hyphen in the generic names as an orthographical error. This would be much easier to deal with and if individual generic names needed attention, this could be done separately.

**Gereau** proposed an amendment to remove “fossil-genus” and say, “The use of a hyphen in the name of a genus is in all cases to be treated as an error to be corrected”.

**Herendeen** could not comment on how common this might be in the names in genera of living plants, fungi or algae, but it was a serious problem in fossils. This amendment might result in the whole proposal going down in flames, so he considered the **amendment unfriendly**.

**Gereau** withdrew his amendment: “Thinking it through, I don’t want to go there!” [*Laughter*]

**Turland**: “We are in Mordor already!”

**Middleton** reproposed Gereau’s withdrawn amendment as an unfriendly amendment.

[The **amendment** was supported by **five seconders**.]

**Redhead** said he was looking at Art. 20.3 regarding the hyphen in a generic name. He stated that Art. 20.3 would have to be removed if this proposal were to be accepted.

**Applequist** raised a point of order: after five seconders for this amendment had been obtained, the Section should have voted on the amendment.

**Knapp** apologized and asked for comments on the amendment to leave off the word “fossil”, which was germane to Redhead’s comment.

**Applequist** did not think that many names would be affected but would be uncomfortable voting on it without knowing the facts.

**Schori** noted that Art. 60 Note 3 specified that Art. 60.9, which referred to hyphens in compound epithets, “refers only to epithets (in combinations), not to names of genera or taxa in higher ranks; a generic name published with a hyphen can be changed only by conservation”. She thought this Note would also be affected.

**Turland** pointed out that this was covered in Prop. G.

**Knapp** agreed that this comment was not germane to leaving the word “fossil” out; it was discussing the entire proposal.

**Cantrill** stated that the Committee had discussed this and their reason for leaving “fossil” in was because of some of the other issues it would affect in the *Code*. The best approach was to keep the word “fossil” and constrain it to those names that were related to fossils. He was against deleting the word “fossil”.

**Lindon** said she had done a very quick survey in IPNI up to the letter *N* and at least 96 names of genera contained hyphens This was offered to give delegates an idea of the number of genera that would be affected.

**McNeill**, speaking against the amendment, had long thought it anomalous that it was possible to correct the spelling of an epithet but not the spelling of a generic name. However, the correction of the spelling of an epithet that had a hyphen in it was quite carefully controlled. To require the deletion of the hyphen in all generic names without the same sort of provisions as there were for specific epithets would be very undesirable.

**Kusber** pointed out that the genus *Pseudo-nitzschia* with a hyphen had been highly discussed in the phycological community, so he warned that the Section had to be careful about what they were doing.

[The **amendment** was **rejected**.]

**Herendeen** wished to address the issue with Art. 20.3. The Committee was aware of this and had discussed it with McNeill. Art. 20.3 said that a name of a genus might not consist of two words. In the names that came up in the Doweld proposal none of them consisted of two freestanding words. For example, *Cicatricosi-sporites* would not fall under Art. 20.3.

**Art. 60, Prop. F, Prop. G** and **Art. 20, Prop. B** were **accepted**.

**Art. 60, Prop. H** (50: 0: 13: 1) was **automatically** sent to the **Editorial Committee**.

**Art. 60, Prop. I** (9: 0: 55: 0) and **Prop. J** (4: 17: 43: 0) were **automatically** sent to the **Editorial Committee**.

**Art. 60, Prop. K** (34: 24: 6: 0) and **Prop. L** (11: 46: 7: 0)

**Knapp** noted that Art. 60 Prop. K suggested the addition of a new voted Example, so the Section would discuss it rather than automatically referring it to the Editorial Committee.

**Turland** explained that Art. 60 Prop. K and Prop. L were by the same authors and essentially were two alternatives. They concerned voted Examples, which as he had mentioned on day one, functioned as a rule to govern a specific case. These examples would govern when a hyphen was to be omitted or maintained in epithets formed from names containing a preposition or a definite article: whether the hyphen in a preposition or definite article was to be omitted or not inserted on the one hand, or maintained or inserted on the other. According to the proposers, if Prop. K were to be accepted (hyphen to be omitted or not inserted) 139 and 209 records would require correction in IPNI and the *World Checklist of Selected Plant Families* respectively. If Prop. L was approved (hyphen to be maintained or inserted) the proposers noted that these records could not easily be found via a search. Therefore, the number of epithets affected was unknown and would have to be dealt with when they came to light.

**Greuter** had one concern about the proposal, which he thought was otherwise perfect: the use of the asterisk. In his opinion, the removal of the hyphen in these epithets was fully covered by the current rule, Art. 60.9. If the Section approved this as a voted Example it appeared to say that it was not covered by the current rule, which could affect other epithets. The current rule, as usually interpreted, was that two independent words must stand independently side by side in order to permit the hyphen. Whereas La Sierre and Le Testu could stand side by side in a normal phrase, *lasierrana* and *letestui* could not, which meant that he supported the proposal, but proposed deletion of the asterisk.

**Turland** noted that he had some comments to add. He had received an analysis of these two proposals from Luis Parra Sánchez, who had extracted data from IPNI and had come up with some figures on the impact of accepting either one of these proposals as a voted Example. He asked the President if the Section was still considering these proposals as voted Examples.

**Knapp** confirmed that Greuter had indeed proposed an amendment to delete the asterisk from Prop. K.

**Govaerts**, as a co-author of the proposal, said Greuter’s **amendment** would be considered **unfriendly**. As the Rapporteur-général had just explained, a voted Example would rule how an Article should be interpreted. Although it was true that Art. 60.9 ruled that words had to stand independently, the question was if “le, la, les, von, van” could stand independently. Some people said they could not, because it was always “le Testui” or “la fille” or “les enfants”. But it was also possible to make sentences where these words were independent. No one over the last ten years had been able to give him a definite answer regarding what to do. This was why the proposers wanted a voted Example.

[The **amendment** was supported by **five seconders**.]

**Knapp** confirmed that Section was now debating whether to delete the asterisk from Prop. K to change it from a voted Example to a regular Example.

**Barrie**, having had experience when dealing with mechanical typification of fighting a voted Example that people had tried to get rid of for 60 years, was in favour of deleting the asterisk. He noted that this proposal added a voted Example for something that was already explicit in Art. 60.9. Voted Examples were ones used for specific exceptions to the other rules, behaving as another ruling in and of themselves. In this case he did not think this was necessary because the examples in Prop. K could simply be added to Art. 60 Ex. 24, as Greuter had pointed out.

**Sennikov**, having asked the opinion of other users, was under the impression that Art. 60.9 could be interpreted differently by different people. One interpretation was that “words that usually stand independently” meant the words that appeared wholly in the epithet. The second interpretation, which was not uncommon, was that people thought the wording applied to the original words from which the epithet was derived. He thought the proposal was a good idea, as it would make clear what was implied in Art. 60.9, but he thought the Example should be a normal, non-voted one.

**Govaerts** asked a question regarding procedure. He wondered if the Example would go straight to the Editorial Committee if it were to be changed to a normal Example. If that were the case, he felt that there would be no point in the proposal because the Editorial Committee would then decide which of the two Examples were correct.

**Knapp** ruled that, given that there would have to be a decision about which of the examples would be sent to the Editorial Committee, she would put it to the Section rather than let the Editorial Committee decide, adding that this was probably totally out of order, but that it was late in the day.

[The **amendment** to delete the asterisk in Prop. K was **accepted**.]

**Turland** noted that the Section had now created a proposal that consisted of only an Example, which should automatically be sent to the Editorial Committee. However, the Section could now move on to Prop. L and, if this Example were to be treated in the same way, he did not see any reason why the Section could not then vote on the two Examples as alternatives and as a message to the Editorial Committee as to which Example was more appropriate. He asked Knapp if this would be in order and sensible.

**Knapp** ruled that this was in order and asked the original proposers if this would achieve their purpose.

[The proposers agreed.]

**Wiersema** added that if one of the Examples turned out to be counter to the Article, the Editorial Committee would not be empowered to put it in.

**Knapp** decided that the Section would therefore need to discuss the relative merits of the Examples and decide which to agree on. She proposed to delete the asterisk from Prop. L, to make it parallel with Prop. K.

[The **amendment** was supported by **five seconders**.]

**McNeill** said that according to the advice delegates had been given it was Prop. K that was in accordance with the *Code*. He suggested rejecting the amendment to remove the asterisk from Prop. L and then proceeding to a vote to defeat the proposal outright. This would answer the question completely.

**Greuter** asked the Rapporteur-général to share what Luis Parra Sánchez had said about Prop. L.

**Turland** noted that in the Excel table he had been sent there were 789 names containing the particles mentioned in the two proposals, this could be verified in IPNI. Of these, 662 names (84%) were written with a single word and 127 (16%) were written with hyphenated particles, in both cases irrespective of the spelling in the original work. If Prop. L were to be accepted, 662 records would have to be changed, while if Prop. K were accepted, only 127 records would be changed. Parra had verified that of the 131 plant names and four fungal names dedicated to Le Testu, 121 were published with a space between the two elements of the epithet and 14 with joined elements where there was no space or hyphen. No epithets were published with a hyphen. Parra had also commented that Prop. K was in accordance with Rec. 60C.5(c), which was what McNeill had been alluding to. Turland thought, after calculating the effect of Prop. L in IPNI, it would be much worse than Prop. K in terms of nomenclatural stability.

**Govaerts** disagreed with the numbers. Of the [more than] 600 epithets that did not have a hyphen, not all would need to be corrected to have a hyphen, so he thought the comments of Parra were erroneous. Regarding the reference to Rec. 60C.5(c), which was in the original proposal, this had nothing to do with hyphens: it merely said that the preposition should be maintained within an epithet and did not say whether or not it had to have a hyphen. He thought it was irrelevant to this Article.

**Knapp** said that if she had correctly followed the silken thread through the Minotaur’s cave of hyphens, the Section was now returning to the decision about whether to delete the asterisk from Prop. L.

[The **amendment** to delete the asterisk in Prop. L was **accepted**.]

**Knapp** then suggested that Prop. K and Prop. L had been made equivalent and the Section would vote between them. Delegates would now discuss the two options as alternatives. There would be a vote to accept one of the Examples, which would be determined by a simple majority. The chosen Example would then be sent to the Editorial Committee with the understanding that they would add it to the *Code*. Knapp cautioned delegates to think about whether either of these proposals was contrary to something else in the *Code*. She suggested they be discussed rather than immediately voted upon.

**Applequist** pointed out that others had already mentioned the greater impact of Prop. L, which had received a stronger negative mail vote than Prop. K. This indicated that there was a strong feeling among voters that they did not want to be forced to insert hyphens into epithets that started with *le* or *la*.

**Gereau** stated that Prop. L did not suggest that anyone was forced to insert a hyphen. The epithet in question was published with a hyphen in 1950 as “*la*-*sierrana*”; those were two words that usually stood separately, so this was perfectly in accordance with Art. 60.9. In the case of “*le testui*”, it was published as two separate words [separated by a space, not by a hyphen], in which case a hyphen would be inserted. Both examples were in accordance with the Article. If an epithet was published without a hyphen [as a single compound word], there was no provision for having to insert one. Art. 60 Prop. L was in accordance with Art. 60.9 and was the preferable one as it did not force anything else.

**Govaerts** agreed with Gereau.

**Geltman** was worried about what would happen with the epithet *decandollei* because it would be necessary to investigate the original spelling to see if a hyphen had been used. The same epithet could appear in two ways.

**Govaerts** wished to make a clarification about the corrections necessary for Prop. L: IPNI had always recorded the names as published so, in principle, there would not be any changes in IPNI. However, IPNI was not perfect and there could be mistakes. He did not think that the number of changes was relevant and there would be no *decandollei* that would have to be changed; he was not aware of this epithet ever having been published with a hyphen.

**Garland** stated that there was some implied usage that people followed that was not spelled out in the *Code*, for example, the epithet *costaricensis*. “Costa” was a separate word in the name of the country Costa Rica, but a hyphen was supposed to be removed in *costaricensis*. The first word was not inflected, it was the basic form of the word, or in Latin the nominative case. The second part of the word had an adjectival ending on it, *ricensis*, so the implied method that people used with hyphens seemed to him to be that if the first word was the completely separate word in its basic form and the second word was an inflected form, an adjectival form or a genitive form, then the hyphen was removed. However, this was not explained anywhere in the *Code*. He thought that whatever Example was chosen would be helpful. His personal preference was Prop. K because he thought that was more akin to current practice: hyphens were removed rather than inserted or maintained.

**Greuter** agreed with Garland that the words “cannot stand independently” were not very satisfactory, and the only way to interpret it would be through the Examples now given. To make the *Code* quite clear he thought he should now propose to set up a Special-purpose Committee to deal with it. But, he said, he would not do that! [*Laughter*]

**Knapp**: “Mount Doom.”

**Greuter** went on to say that words were treated as able to stand independently when they could stand side by side in a phrase that made sense, which was not the case of *costa* and *ricensis*. In Ex. 24, *costaricensis* had no hyphen, whereas all those compound epithets in Ex. 26 showed that these words could stay jointly in a phrase that made sense: of Prince Roland [*rolandii-principis*], grape of Mount Ida [*Vitis-idaea*], aquatic *Anagallis* [*anagallis-aquatica*]. If Prop. K and Prop. L were sent to the Editorial Committee and the Editorial Committee followed the criteria set out in Ex. 24, it would have to adopt Prop. K and not Prop. L, because the asterisks had been removed.

**Paton** quipped that he was trying to keep a happy thought in his head and was thinking about the road out of Mordor. He went on to say that Prop. K meant that anything named after Le Testu would end up consistently as *letestui* whereas Prop. L would result in a mixture. So, for those people who did not love orthography, Prop. K would result in easier standardization than Prop. L would. He would vote for Prop. K.

**Knapp**, having received no further comments, thanked Paton for ending on a high, with his suit of elven chain mail issued to him as he left the doors of Kew. Knapp then asked the Section to vote between the two alternatives: Prop. K and Prop. L. This would be a simple majority vote.

**Art. 60, Prop. K** was **accepted as amended**.

**Art. 60, Prop. L** was **rejected**.

**Art. 60, Prop. M** (56: 4: 4: 0)

**Turland** noted that this was the Scottish proposal.

**Knapp**: “Like the Scottish play.” She explained for those who were not familiar with it, that the Scottish play was Shakespeare’s *Macbeth*, where everybody dies.

**Turland** noted that it is considered inauspicious to utter the play’s true name.

**McNeill** explained that the proposal had arisen from an IPNI entry for the epithet named after “McKen”, in which a reverse quotation mark [‘] and then “*Ken*” followed the initial *M* in the original publication. When added to IPNI the epithet had been joined together as “*mkenii*”, which made no sense because the original work acknowledged the services of McKen. The joining of the two parts of the epithet was contrary to the *Code*, because the original work used a reverse quotation mark, or “6-quote”, not an apostrophe [’]. Strictly speaking, this mark should have been left in the epithet. The 6-quote was commonly used in place of a superscript *c* [*^c^*] and came about because a superscript *c* was not available in 19^th^ century typesetting. This proposal would ensure that people whose names began with *M* followed by a 6-quote should simply have them transcribed as *Mc.* He proposed to use the word quotation mark because it was not an apostrophe. It was, he thought, a very minor matter, but avoided rather silly-looking epithets like “*mkenii*”.

**Art. 60, Prop. M** was **accepted**.

**Art. 60, Prop. N** (4: 36: *24: 0) was **automatically** sent to the **Editorial Committee**.

**Art. 60, Prop. O** (40: 2: 21: 0)

**Turland** stated that this proposal was editorial. The idea behind it was that terminations formed in accordance with Rec. 60C.2 were presumably correct and could hardly be corrected. The proposal was to replace the wording “are not to be corrected” with “are to be accepted as correct”. Turland proposed that this be sent to the Editorial Committee.

[The **proposal** was supported by **five seconders**.]

**Art. 60, Prop. O** was sent to the **Editorial Committee**.

**Art. 60, Prop. P** (4: 44: 15: 0), **Prop. Q** (5: 45: 13: 0) and **Rec. 60C, Prop. A** (8: 42: 13: 0)

**Turland** noted that the second and third of these three proposals were contingent on the first being accepted. The proposals sought to permit epithets such as that of *Syringa josikaea* to be accepted as correct, whereas otherwise they would be correctable under Art. 60.12, but without the correct form being apparent. The Rapporteurs had noted that these cases were very rare and perhaps it was better just to tolerate them rather than explicitly permit an indefinite number of new names with a new kind of epithet derived from a personal name. One of the Examples under Prop. P, *Cacalia kleinia*, could be considered by the Editorial Committee as a possible Example under Art. 60.12, because the epithet was a pre-1753 generic name used in apposition, to which one could argue that Art. 60.12 and Rec. 60C.1 did not apply.

**Art. 60, Prop. P** was **rejected**, meaning that **Prop. Q** and **Rec. 60C, Prop. A** were **automatically rejected**.

**Art. 60, Prop. R** (19: 34: 8: 3)

**Turland** said that this proposal ventured where angels feared to tread. It proposed splitting Art. 60, while promoting Rec. 60C.1 and Rec. 60G to rules: one Article on original spelling, one on allowable characters, one on personal names, and one on compounds. This would result in a major overhaul of Art. 60, splitting it into four Articles and would promote the so-called backdoor rules of Rec. 60C.1 and Rec. 60G to Articles. This would be purely editorial and could be carried out by the Editorial Committee, otherwise a “no” vote would avoid a lot of time-consuming and needless restructuring. [*Laughter*] Turland reiterated that this could be sent to the Editorial Committee and, if that Committee was feeling particularly bold, they might consider doing the restructuring. However, he thought the Editorial Committee would appreciate the Nomenclature Section’s guidance and he asked if the Section could vote on it.

**McNeill** welcomed the possibility of the *Code* not having backdoor rules in Recommendations. If the Editorial Committee could achieve this, it would be a great advantage. He supported the suggestion, not the requirement, that the Editorial Committee look at this.

**Wilson** agreed with McNeill that looking at this was long overdue, but she thought that the Editorial Committee should do it, not just look into it.

**Greuter** commented that he missed “our old friend” Vincent Demoulin very much during these sessions, but were Demoulin to be here, he would not make the proposal he was about to make: that there should be a Special-purpose Committee on Orthography. [*Laughter, groans*] Greuter could not see the Editorial Committee, which was a very competent body, coping with this alone in an editorial manner unless it got advice from a competent Special-purpose Committee delving into the quite funny questions of orthography.

[The **proposal** was supported by **five seconders**.]

**Gereau** stated that if the re-establishment of a Special-purpose Committee on Orthography was going to “shove this down the road another six years” when the Section had in front of them a good proposal for a long-overdue restructuring to end the backdoor status of Rec. 60C.1, removal of a major inconsistency and source of confusion from the *Code*, and making it understandable to novice and even intermediate users of the *Code*, then he was very much against it.

[The proposal was **rejected**.]

**Turland** proposed to refer Prop. R to the Editorial Committee.

[The **proposal** was supported by **five seconders**.]

**Wilson** asked the Editorial Committee to clarify whether this proposal meant they were going to do the work or not do the work. [*Laughter*]

**Knapp** invited Turland to speak as the Editorial Committee Chair, saying that he had brought this on himself. [*Laughter*]

**Turland** said he did not think that the Editorial Committee could escape from this proposal unless the Section voted no, which he did not think they would do. He thought converting the rules into Articles would be relatively straightforward editorially. Splitting Art. 60 would also be straightforward, but he did not know if this was a useful thing to do. The Section would have to trust the Editorial Committee to decide whether that made a more useful section on orthography. He had noted the desire from delegates for restructuring, particularly for the backdoor rules to be eliminated.

**Art. 60, Prop. R** was sent to the **Editorial Committee**.

#### Recommendation 60C

**Rec. 60C, Prop. A** was discussed under **Art. 60, Prop. P** and **Prop. Q**.

**Rec. 60C, Prop. B** (47: 4: 13: 0)

**Turland** noted that this proposal introduced a little more latitude into Rec. 60C.5(a) by recommending that variants of the Scottish and Irish patronymic prefix, when used in epithets, could be spelled not only as *mac*, but alternatively as *mc.* The epithet *mcneillii*, for example, would not then be contrary to the recommendation. [*Laughter*]

**Rec. 60C, Prop. B** was **accepted**.

#### Recommendation 60E

**Rec. 60E, Prop. A** (4: 55: 5: 0) was **rejected** based on the **mail vote**.

#### Recommendation 60G

**Rec. 60G, Prop. A** (1: 1: 62: 0) was **automatically** sent to the **Editorial Committee**.

#### Recommendation 60H

**Rec. 60H, Prop. A** (4: 16: *44: 0)

**Turland** explained that this Proposal would amend Rec. 60H.1 to include also the epithets of replacement names. This was perfectly logical, but the wording “new epithets” might obscure the point that the new epithet was new because it was in the name of a new taxon or replacement name and not in a new combination or a name at new rank. This was somewhat parallel to Rec. 60E Prop. A, which was ruled as rejected in the mail vote. The Rapporteurs had advised that those who wished to add the epithets of replacement names to Rec. 60H.1, without the other proposed changes, should vote “ed.c.”. Turland stated that this was not an editorial proposal. It would add the element of the epithets of replacement names to the Recommendation, but this could be done in a slightly less obscure way. Turland proposed an amendment to do this, by rewording the proposal as: “The etymology of new generic names or of epithets in the names of new taxa or replacement names should be explicitly stated, especially when the meaning is not obvious.”

[The **amendment** was supported by **five seconders** and was **accepted**.]

**Rec. 60H, Prop. A** was **accepted as amended**.

**Rec. 60H, Prop. B** (17: 41: 6: 0)

**Turland** explained that Prop. B concerned etymological practice when forming compound adjectival epithets in which the genitive form of a generic name appeared in a non-final position. The proposed Recommendation as currently worded was not easily interpreted, but the supporting text in the proposal was clearer. It recommended effective publication of a proposed genitive form of a generic name. If this were a rule it would be parallel to the formal nomenclatural acts of Art. 61.3 and Art. 62.3. The Section had to decide whether this additional guidance was useful and required.

**Gereau** thought that the practice being recommended was desirable, but the statement was so unclear that he did not think it could be editorially fixed. On that basis, he was against the proposal.

**Greuter**, speaking as someone who had great respect for nomenclatural tradition, did not like the words “at least” that preceded “nomenclatural tradition” in this proposal. As worded, it appeared to say that in the first instance classical usage should be followed and nomenclatural tradition should only be considered secondarily. He proposed an [unfriendly] amendment to delete the words “at least”.

[The **amendment** was supported by **five seconders** and was **accepted**.]

**Rec. 60H, Prop. B** was **rejected**.

**Knapp** announced that the Section had reached the end of Art. 60 [*Applause*] noting that everyone had survived, and no one had been eaten by orcs. As President, Knapp proposed that the Section would not deal with issues of governance 15 minutes before the end of the session. The Rapporteur-général had suggested that the Section continue by debating App. I. She proposed to start with the Div. III proposals the next morning when everyone was fresh.

#### Appendix I

**App. I, Prop. A** (12: 3: *50: 0)

**Turland** noted that this was an editorial proposal, but it was a rather bold one, possibly bolder than the proposal to restructure Art. 60, if such a thing was possible. [*Laughter*] The proposal was to move App. I, the Appendix on names of hybrids, into the main body of the *Code* as a new chapter: Chapter X. The proposal specified that the Articles should be renumbered, so that Art. H.1 to Art. H.12 would become Art. 63 to Art. 74. This would, of course, depend on any renumbering in the preceding Chapters of the *Code*. Recommendations would be renumbered accordingly. The Appendices would also be renumbered and relevant cross-references throughout the *Code* would be adjusted. This seemed to be a perfectly logical adjustment, especially if App. II to App. VIII were separate from the main body of the *Code*. However, the Rapporteurs considered that it would be clearer and less disruptive to retain the current numbering of Articles and Recommendations from Art. H.1 to H.12, simply following on from Art. 62. There was no logical reason to change the current numbering. The renumbering of App. II–VIII could also be achieved with minimal disturbance by renumbering App. IIA and IIB as App. I and II, respectively.

[This was **accepted** as a **friendly amendment**.]

Turland confirmed that the proposal now was to move App. I into the main body of the *Code*, retaining the current numbering of the Articles and Recommendations within the hybrid Appendix, and to renumber App. II–VIII, which would only require changing the numbering of App. IIA and IIB as App. I and II, respectively. There would be minimal renumbering, but the hybrid Appendix would cease to be App. I and would become a chapter of the *Code*. He proposed that App. I Prop. A be sent to the Editorial Committee.

**Knapp** ruled that Section should vote on the proposal, because the current principle was that anything sent to the Editorial Committee may or may not happen. She thought that it would be better to find out what the will of the Section was and whether they wanted this to happen or not.

**Greuter** agreed with the Rapporteur-général that it was perfectly logical to include the hybrid Appendix in the body of the *Code*, because that was where it belonged. Such a move had been carried out at an earlier Congress regarding the guide to typification, which had also been moved to the main body of the *Code*. He did not think, however, that the hybrid Appendix should just be placed all together at the end. He proposed that the amendment be modified so as not to make the numbering and position of the present Articles explicit, leaving the choice to the Editorial Committee. For example, provisions on nothospecies might sit better in the section relating to species names, and nothogenera might be best placed in the generic name section.

**Knapp** clarified that the first [friendly] amendment was to delete the phrase “Renumber the Articles”, and Greuter’s amendment would be to delete the words “as Chapter X”.

[Greuter’s **unfriendly amendment** was supported by **five seconders** and was **accepted**.]

**May** thought the proposal was eminently sensible but wanted to put the idea to the Editorial Committee that calling it Chapter H in line with the suggestion for a Chapter F, for fungi, could be useful.

**Applequist** wished to propose an unfriendly amendment regarding renumbering of the Appendices, but immediately apologized, realizing she was wrong.

**Knapp** thought this was perfectly understandable as it was getting to the end of the day. She moved to a vote on App. I Prop. A, to move App. I into the main body of the *Code*, renumber the Appendices and editorially adjust things.

**App. I, Prop. A** was **accepted as amended**.

**Knapp** closed the meeting for the day, noting that the deadline for proposals from the floor had passed and reminding delegates to fill in the sign-up sheets for buses going to the main Congress venue.

### Friday, 21^st^ July 2017, Morning Session

**Knapp** welcomed everybody back, congratulating delegates for having made it through four days so far. She noted that the previous day’s discussion ended after App. I, and that the discussion of Div. III, provisions for governance of the *Code*, had been deferred until this session.

#### Division III

**Div. III, Prop. A** was discussed under **Art. 42, Prop. B, Prop. C** and **Prop. D** [*see beginning of Thursday morning session*].

**Div. III, Prop. B** (59: 3: 1: 1)

**Turland** wished everyone a good morning. He reminded the Section that Div. III Prop. A, concerning the Registration Committee, had been discussed in the morning session the previous day. This session would, therefore, begin with Div. III Prop. B, from the Special Committee on By-laws for the Nomenclature Section, to replace Division III of the *Code* with a new version. The General Committee almost unanimously supported the general principle of the new Div. III, supported the proposed new paragraphs that affected the General Committee, and supported the collective name “specialist committees”. The specialist committees in the proposed new Div. III were the five committees for algae, fungi, fossils, vascular plants and bryophytes. The General Committee did not support certain details of Rec. 7A, Prov. [Provision] 7.11, and Prov. 7.12 [in the proposed new Div. III] and had proposed amendments. These amendments were considered by the Special Committee on By-laws, which voted to accept the proposed amendment to Rec. 7A as friendly. The Special Committee did not consider the other two proposed changes in Prov. 7.11 and Prov. 7.12 to be friendly.

**Knapp** interjected that the Special Committee did not consider them friendly because half the Committee felt they were friendly, and half did not.

**Turland** added that, because several people were not present on Tuesday morning for the general discussion about governance issues, he would invite Herendeen to introduce the proposal on behalf of the Special Committee on By-laws.

**Herendeen** explained that Prop. 286 [Prop. B] to replace Div. III, and the accompanying Prop. 362 and 363 [Prop. C and D] by the fungal subcommittee [Special Subcommittee on Governance of the *Code* with Respect to Fungi] were an outcome of the Melbourne Nomenclature Section [2011], when it became clear that there was a requirement to formalize the procedures in writing. Until then, the process had operated on institutional memory from one Section meeting to the next.

The need came about because of the controversy surrounding *Acacia*, over 12 years ago. One outcome from this was that more detail was written down, and the work of the Special Committee resulted in these documents. There had been a lot of discussion over quite some time to get to this point. Herendeen noted that what was about to be discussed in detail was the result of that Special Committee and the fungal Subcommittee’s discussions. Herendeen added that Knapp had chaired the Special Committee, which was why she had not introduced the subject.

**Knapp** noted that Prop. 286, Div. III of the *Code* [Div. III Prop. B], would be addressed separately from the fungal proposals, which would come later.

**Alford** had several questions and a couple of proposals. He asked if delegates could take each point in turn, i.e. section one, section two [Prov. 1, Prov. 2] etc.

**Knapp** agreed to take them section by section but not bit by bit.

**Herendeen** expressed the need to be expeditious in the discussions and asked the Section to try to be efficient.

**Knapp** reminded everyone that the Section would not be wordsmithing pieces of Div. III and only substantial changes to meaning would be discussed.

**Herendeen** pointed out that the meaning of every word in the document had been debated, so a lot of discussion had already taken place.

**Alford** said he presumed it was still acceptable for him to take these one at a time. He started with Prov. 2, saying he had submitted proposals for conservation, rejection, etc. but not to amend the *Code*. It was not clear to him in Prov. 2 if a proposal to amend the *Code* could be rejected if it did not fit the size limitation. It seemed like a proposal may be rejected if it did not fit the formatting, but regarding content, he asked if a proposal to amend the *Code* could ever be rejected outright.

**Turland** spoke for the Rapporteurs saying, in general, no. If somebody submitted a proposal to amend the *Code* it went to the Bureau of Nomenclature, which, when proposals to amend the *Code* opened, normally consisted of the Rapporteur-général only. Once they came to the Rapporteurs, they could not be rejected. If the proposal was particularly flawed, the Rapporteurs, or whoever was editing the proposals for *Taxon*, could explain the flaws to the proposer and these could be corrected, or the proposer could withdraw their proposal, but it could not be rejected.

**Thiele** suggested two proposals to amend Div. III. The first one he thought was straightforward: to move an item from Prov. 5.2.

**Knapp** interrupted, noting that this was in Prov. 5, “Procedure and voting at the Nomenclature Section”, which was essentially what everyone had been doing for the past four days.

**Thiele** noted that for referring items to the Editorial Committee a simple majority was required. He proposed that it should require a qualified majority [i.e. Prov. 5.2 clause (5) should be moved to Prov. 5.1].

[The **amendment** was supported by **five seconders**.]

**Thiele** went on to explain that one of the reasons for overhauling Div. III was to bring clarity to the procedures of these meetings and he thought that in this meeting there had been some lack of clarity. He thought that referring an item directly to the Editorial Committee as the Section had been doing may require or may allow the Editorial Committee to make an amendment to the *Code*. Just as with other amendments, he thought this should require a qualified majority.

**Knapp** wished to add that this was the procedure that she had instituted in this Section: to use a qualified majority for this kind of vote.

[The **amendment** was **accepted**.]

**Watson** had another amendment for Prov. 5 but recommended that if the wording of the proposed new Div. III had already been changed the Section should first be shown all the changes, because otherwise it was difficult to comment.

**Turland** said there were about three or four other minor changes, which were not editorial. He suggested the Section go through these first.

**Knapp** suggested to the Rapporteur-général that the Section look at the friendly amendment. She explained that this was a change to Rec. 7A, where the General Committee had suggested that the second sentence, “In the General Committee and specialist committees, the number of members entitled to vote should be a multiple of 5”, was probably impractical. The Special Committee had voted on this, agreed and accepted it as a friendly amendment.

**Turland** acknowledged that the Special Committee had agreed and deleted the disputed sentence. The next amendment was in Prov. 2.4. The phrase “specialist committees” had been replaced with “Permanent Nomenclature Committees”. This was when the Rapporteur-général and Vice-rapporteur might ask for committee opinions to include in the synopsis of proposals. “Specialist committees” would exclude the General Committee, so McNeill proposed putting “Permanent Nomenclature Committees” to include the General Committee, which may also give comments on proposals to put in the synopsis. Turland noted that this amendment must be approved if it were to be added.

[The **amendment** was supported by five **seconders**.]

**Funk** spoke in support of the change, noting that two new Permanent Nomenclature Committees were being added and they may have to consult for various items such as registration and institutional votes.

**Applequist** asked if McNeill would accept a friendly amendment to change “specialist committee opinions” in Prov. 2.6 to “Permanent Nomenclature Committee opinions”.

**Knapp** pointed out that this was a separate issue because there was a proposal to change the names of some of the Permanent Nomenclature Committees to specialist committees.

[The **amendment** was **accepted**.]

**Turland** introduced the next change, in Prov. 4.11, which came from the Bureau of Nomenclature. The original text read: “The Nominating Committee comprises members who must be unavailable to serve on the Permanent Nomenclature Committees or as Rapporteur-général”. The Bureau attempted to put together a Nominating Committee and found it extremely difficult, given the number of people at the Section and the number of people who would be likely to serve on Committees or who wanted to be available to serve on Committees. They decided that making this a rule using the word “must” was impractical, and proposed that it should be, “should preferably be unavailable”.

[The **amendment** was supported by **five seconders**.]

**Paton** asked for clarification of the term “unavailable to serve”. He wondered what constituted being unavailable to serve.

**Turland** explained that somebody who was unavailable to serve on one of the Committees was somebody who would not be nominated as a member of one of those Committees. Ideally the members of the Nominating Committee should not be nominating themselves for the Permanent Nomenclature Committees. It was to prevent the Nominating Committee from nominating some of their own members, or all their own members, to Permanent Nomenclature Committees. It aimed to create as much impartiality as possible.

[The **amendment** was **accepted**.]

**Turland** said the next amendment was in Prov. 5.6. He began to explain the rationale behind deleting the word “Notes”.

**Knapp** asked Turland to wait, pointing out that he had to propose the amendment, and she had to get five seconders for it before he could discuss the rationale. She explained this was an unfriendly amendment because it came from him. [*Laughter*]

**Knapp** backtracked, explaining it was not unfriendly just because it came from Turland, but that the amendment in fact came from the Bureau of Nomenclature and was not a friendly amendment because it had not been discussed with the entire Special Committee.

[The **amendment** to delete the word “Notes” was supported by **five seconders**.]

**Turland** explained that at the beginning of the Section on Monday morning delegates voted to automatically refer proposals to amend the *Code* that concerned only Examples or Glossary items to the Editorial Committee. Notes were not included in that vote. The reason for deleting the word “Notes” was that they were not always editorial, so this Provision should be restricted to purely editorial items such as Examples and the Glossary. The *Code* already stated that the Editorial Committee had the power to introduce new Examples into the *Code*, to delete Examples, and to move or edit Examples as it saw fit. It would be perfectly in order for this paragraph to stand with Examples and Glossary items. Glossary items must not contradict anything in the *Code*, so they were also purely editorial. Notes could go beyond the editorial and Turland thought it a little dangerous to have “Notes” in Prov. 5.6 and preferred that it was kept out.

**Greuter** noted that on the previous afternoon, there had been a couple of items that were purely within the Editorial Committee’s competence and had been accordingly sent to the Editorial Committee, but which could be mentioned at this point. He moved that “Notes” should instead read “placement” or “numbering”.

**Knapp** pointed out that first the Section had to finish discussing deleting “Notes” before any discussion on replacing it with something else.

[The **amendment** to delete the word “Notes” was **accepted**.]

**Turland** moved to the next amendment, in Rec. 7A, after Prov. 7.6. This was the friendly amendment that had already been accepted by the Special Committee, so it did not need to be discussed.

**Watson** commented on the composition of the Editorial Committee in Prov. 7.4. There was an implication that only individuals who had been at the Nomenclature Section of the previous IBC could serve on the Editorial Committee. He thought this was laudable but could see situations where individuals were not able to attend for a particular reason, maybe transport or temporary health issues. He thought it would be a shame not to be able to include such individuals on Editorial Committees if they wished to serve on one. He proposed an amendment to change the wording of Prov. 7.4 from “who were present” to “who should preferably have been present” at the Nomenclature Section.

[The **amendment** was supported by **five seconders**.]

**Barrie** explained that traditionally it was felt to be advantageous for the Editorial Committee to be comprised of people who had participated in the Nomenclature Section, because they would be the ones who made the decisions for editing the *Code.* They should have some memory of the Section, hopefully a nice memory, which would help them make decisions in complicated situations so that the intent of the Section was in the *Code* and nothing was contrary to it. He agreed that there were people who would be missed, for example Vincent Demoulin, and thought it unfortunate that under the current rules he could not be on the Editorial Committee. However, in the long run he thought it more important to have people who participated in the Nomenclature Section on the Committee.

**Struwe** spoke in favour of this amendment because she thought it easy to exclude people that could not travel for various reasons, for a meeting that occurred only once every six years. There were also people that could not afford to go to these meetings. In future the Sections may be held in a format where people could call in from outside, which may change the meaning of participation. Struwe asked if someone had to be physically present or whether they could be present by some other means of electronic communication.

**Redhead** also supported the change, adding that if the amendment was not accepted, he proposed to change the word “the previous” to “a previous” International Botanical Congress. In this way, if someone could not be present at the Nomenclature Section but still had vast experience, they would still be eligible to serve on the Editorial Committee.

**Watson** said he agreed with Barrie’s sentiment, but noted there was no definition of how long someone needed to be present at the Nomenclature Section. He asked if it was okay just to turn up for the first hour, the first day, or did one need to be there the whole time? [*Laughter*]

**Knapp** suggested that nobody would dare turn up for only the first hour.

**Freire-Fierro** agreed with Struwe, saying that maybe in the future there would be online participation and suggested changing the proposal to read, “who would have participated in person or virtually”.

[The **amendment** to the **amendment** was supported by **five seconders**.]

**Wiltshire-Hawksworth** asked if participation by proxy would be considered if the Section was going to go online.

**Gereau** suggested that delegates were wordsmithing and the amendment to the amendment was unnecessary. The Section had not yet defined what “present” was and proposals were coming up for the next Congress to expand participation in various ways, so that people could participate remotely. He thought it unnecessary to add the words “in person or virtually” at this time.

**Price** thought “should preferably” had been added because “the previous International Botanical Congress” was confusing. It meant the one for which the *Code* was being edited, not Melbourne [2011] in this case.

**Knapp** pointed out it was not relevant to the discussion of the “in person or online” part.

**Price** agreed to hold her comment until the discussion on the amendment to “should preferably have been”.

[The **amendment** to the **amendment** was **rejected**.]

**Price** felt that there was a conceptual issue with “at the previous International Botanical Congress” with respect to “should preferably have been”. What delegates were talking about was that persons wishing to serve on the Editorial Committee for the *Shenzhen Code* should have been present at the Shenzhen Congress [2017], not at Melbourne [2011]. She thought this was what was causing confusion with respect to the presence of persons at a Congress and she thought the phrase should, perhaps, be clarified.

**Watson** asked if it would help to change “previous” to “relevant”?

**Knapp** accepted this as a **friendly amendment** to Watson’s **amendment**.

**Hawksworth** risked being accused of wordsmithing by saying it might help if “normally” was added before “comprises” and then delete “should preferably have been”.

**Watson** considered the **amendment unfriendly**.

**Knapp** cautioned against wordsmithing.

**McNeill** disagreed with the President that putting in “normally” was wordsmithing. He thought there was a difference between “normally comprises individuals” as opposed to saying, “who should preferably have been”. He thought “normally” meant that it really was only if they missed their flight, if something terrible went wrong, whereas “should preferably have been” was rather more open. He agreed with Barrie that the whole ethos of the Section meeting was very important if someone was going to serve effectively on the Editorial Committee.

**Knapp** clarified that the amendment to the amendment read: “normally comprises individuals who were present at the Nomenclature Section of the relevant International Botanical Congress”.

[The **amendment** to the **amendment** was supported by **five seconders**.]

**Levin** said he did not interpret “normally” in this case the same way as the person who had made the amendment. It did not refer to the travel plans of the person, it referred to how the Editorial Committee was put together. This would say that, except under abnormal circumstances, most Editorial Committees would consist of people who had been present at the Section meeting, but it could be different.

[The **amendment** to the **amendment** was **rejected**; the phrase now stood at “The Editorial Committee comprises individuals who should preferably have been present at the Nomenclature Section of the relevant International Botanical Congress…”.]

**Groom** called the question.

[The Section voted **to vote** on the amendment, and the **amendment** was **accepted as amended.**]

**Alford** requested moving to Prov. 5 and proposed to add a Prov. 5.8*bis* or something that stated that, “A proposal to modify the *Code* may be ignored (see Prov. 5.5), voted yes or no, tabled, automatically sent to the Editorial Committee (see Prov. 5.6), sent to a Special[-purpose] Committee, or sent to the Editorial Committee. If the Section sends a proposal to the Editorial Committee, this gives the Editorial Committee the liberty not only to adjust the wording, format, placement, etc., but also to make the decision whether to include or to exclude the content or spirit of the proposal (see Prov. 7.9)”.

**Knapp** prevented Alford from speaking further until the amendment had been typed out, seconded, and voted upon.

**Watson** sought clarification regarding the procedure for introducing a proposal. He noted that if you introduced a proposal [from the floor], you could chat about it first and then make a proposal, but if you stated the wording of the proposal first you could not retrospectively provide the background and reasoning for it.

**Knapp** agreed, but said that delegates should have chatted about an amendment that they wanted to make from the floor before they made it. Amendments from the floor had to be well thought-out as the Section had discovered over the last four days; amendments from the floor that were made without thinking them out often did not work very well.

**Funk** thought all proposals had to be in by six o’clock the previous evening.

**Knapp** agreed that this was a large change but pointed out that, technically, it was amendments that were being discussed, and proposed concentrating on the spirit of the changes rather than wordsmithing them to death.

[The **amendment** was supported by **five seconders**.]

**Alford** explained the point of the amendment was to clarify what happened when the Section voted to send something to the Editorial Committee. He voted against sending many things to the Editorial Committee because he thought it was the Section’s responsibility to give guidance as to what the Editorial Committee did, yes or no. He thought it made it clear what their responsibilities would be based on the Section’s vote.

**Schori** said the point of a governance document or something that was effectively by-laws was to set forth the procedures. If these were procedures that the Section followed, it made sense to include them.

**Applequist** did not agree that the document could possibly incorporate all the procedures. Those who were simply reading the *Code* had no need to know the details of the procedure and it would be needlessly cumbersome to enumerate everything that was done.

**Barrie** suggested referring this to the Editorial Committee. [*Laughter*]

**Knapp** asked if that would be considered a friendly amendment by the amender. [*It was not.*] She reiterated that it was important that delegates should consider the spirit of this, rather than the exact wording.

**Barkworth** asked if the first sentence could be deleted so that it would begin: “If the Section sends…”, which would clarify the statement.

**Alford** accepted this as a **friendly amendment** to his **amendment**.

**Greuter** thought the moved amendment superfluous because it was covered fully by Prov. 7.9.

**Thiele** thought there was one respect in which the proposed amendment extended beyond Prov. 7.9, and that was specifically to say that the Editorial Committee was given the power to essentially ignore the proposal, which he did not think was in the Article.

[The **amendment** was **rejected**.]

**Thiele** moved to add a new item to Prov. 5.1, which stated “(x) rejecting a singled-out recommendation from the General Committee (see Prov. 5.4)”, under the provisions for a qualified majority.

[The **amendment** was supported by **five seconders**.]

**Thiele** wished to explain without going into the full background of the Div. III rewrite, which had arisen out of the Melbourne Congress [2011] as a result of the *Acacia* controversy. If a specialist committee needed a supermajority to recommend conservation, rejection, etc., the General Committee needed a supermajority to recommend that to the Section. He believed that it should not be easy and straightforward for the Section to overturn such a recommendation from the General Committee.

Further, Art. 14.16 of the *Code* specified that once the General Committee had published its decision with respect to a request to conserve, reject, etc., that decision would go into nomenclatural effect pending ratification at the next Congress. It was entirely appropriate that the Section should be able to overturn a decision of the General Committee, but he believed that a supermajority should be required to do that. These voting procedures were in effect at Vienna [2005]. It was extensively discussed between Vienna and Melbourne in the literature and had become a focus of the discussions around *Acacia* at Melbourne. At Melbourne, the same procedure was adopted, to require a qualified majority to reject a recommendation of the General Committee. Subsequently, the *Acacia* provision was voted on and was soundly accepted. He believed this to be an important part of a check and balance system between the General Committee and the Section.

**Knapp** clarified that *Acacia* was not voted upon at Melbourne. [The *Vienna Code*, the Appendices of which included the conservation of *Acacia* resulting from the Vienna Congress of 2005, was ratified as the basis for discussion at the Melbourne Nomenclature Section; see Flann & al. in PhytoKeys 41: 17. 2014].

**Gereau** said the wording of Art. 14.16 was parallel to that of Art. 34.2, for suppression of a publication; Art. 38.4, for a binding decision on descriptions; Art. 53.5, for a binding decision on confusable names; and Art. 56.4, for rejection of a name. The specialist committee approved a proposal to conserve a name by a qualified majority of 60%. The General Committee approved that recommendation by a qualified majority. At a later IBC, through its Nomenclature Section, a decision would be made whether to approve the recommendation of the General Committee.

In the case of an individual recommendation of the General Committee singled out for reconsideration by the Nomenclature Section under the present proposal, the most logically consistent procedure would be to require a qualified majority of 60% for the Nomenclature Section to approve the General Committee recommendation, just as in the first and second steps of the process.

Out of deference to the experience and expertise of the specialist committees and the General Committee, the Special Committee on By-laws lowered the voting percentage to approve, and thus also to reject, the General Committee recommendation to a simple majority of 50%. This still constituted a decision on the part of the Nomenclature Section, as mandated by the relevant articles of the *Code*. Requiring a qualified majority of the Nomenclature Section to reject a General Committee recommendation, as in the proposed amendment, effectively treated the General Committee recommendation as a decision, contrary to all five relevant articles.

Only an IBC, through its Nomenclature Section, had the mandate to decide these matters and, unless all relevant articles were amended to provide otherwise, the Nomenclature Section would reach this decision by at least a simple majority. Therefore, he was fully against the amendment.

**Applequist** thought the amendment introduced a contradiction, because Prov. 5.2 clause (8) said that a more than 50% majority was required to accept recommendations of the General Committee, which she would support because a 40% minority would be profoundly undemocratic. If the Section indeed should object to only one recommendation out of the several hundred in the General Committee report, they might have no alternative but to vote to reject the entire report and throw hundreds of proposals into chaos.

**Greuter** explained what Art. 14.16 really meant. “Retention of that name is authorized” meant, in dealing with conservation and rejection proposals, that what was authorized was setting apart the provisions of the *Code* for a given name. That given name could not be used unless it was conserved. By decision of the General Committee that name was conserved and the Section should be able to de-conserve a conserved name by rejecting a singled-out General Committee recommendation. For instance, if the General Committee decision was reached five years in advance of the next Congress, botanists throughout the world should have followed this authorization for five years. The practice of five years, legal under the *Code*, could then be reversed by the Section rejecting the General Committee decision. He did not think it logically correct to say that approving the General Committee recommendation added something to the *Code*. In fact, it was already virtually in the *Code*: in the spirit, if not yet in the printed version. The names approved by the General Committee were treated as if they were in the Appendices, and in the future, with the advent of electronic Appendices, they would appear in the Appendices between Congresses. Therefore, he was in favour of the amendment.

**McNeill**, in response to Applequist, said that these two provisions should be in agreement. If this amendment was accepted, then it should also apply to all the recommendations from the General Committee, not simply those that were singled out. They all concerned names that *de facto* were already conserved or already rejected and therefore, for this to be changed by the Nomenclature Section, should require a supermajority.

**Knapp** reiterated that the vote was on introducing a provision for a 60% majority to reject something that was singled out from the General Committee report. She wished it to be clear because there was confusion introduced about this at Vienna, which caused dissension in the botanical community.

[The **amendment** was **accepted**.]

**McNeill** suggested that the implication of the change was that if it applied to those recommendations that were singled out, it should also apply to all the recommendations of the General Committee. He proposed adding the words “not included in 5.1(*x*)” to the end of Prov. 5.2 clause (8) and to amend Thiele’s addition to Prov. 5.1 to make it apply to rejecting all recommendations of the General Committee instead of merely “singled-out” recommendations. He qualified this further by saying that it should only apply to rejecting General Committee recommendations on the conservation and rejection of names, suppression of works, and binding decisions. He explained that, currently, the Section had only covered the singled-out recommendations, and Applequist’s point was that there might be a situation in which you would have to move it back in [with the other recommendations and vote on them together]; they should all be treated the same.

[The **amendment** was supported by **five seconders**.]

**Barrie** thought the issue was that if the Section only accepted Thiele’s amendment, without McNeill’s amendment, because of Prov. 5.2, which only required a 50% majority to accept the recommendations of the General Committee, people who opposed a single recommendation need not single it out. They could just vote for rejecting all the recommendations of the General Committee and it would affect every proposal that the General Committee had submitted, but it would also get rid of the one they opposed. Barrie thought it would be dangerous to set it up without McNeill’s amendment.

**Wilson** suggested adding “one or more” to “recommendations of the General Committee” would cover the singled-out case or the whole case.

[**McNeill** accepted the **amendment** to his **amendment** as **friendly.**]

**Thiele** pointed out that if the amendment was accepted, the Editorial Committee would have the power to deal with the resulting superfluity of the previous amendment by Thiele.

[The **amendment** was **accepted as amended**.]

**Middleton** referred to the last sentence of Prov. 5.9, “No single person will be allowed more than 15 votes, including personal vote and institutional votes.” He asked if the relevant committees considered limiting the number of institutions from which a single person could carry the votes, in addition to the total number of votes that could be carried.

**Turland** said it was not considered, but logically the maximum number would be 14 because 15 votes could be carried, and one of those would be a personal vote.

**Middleton** proposed an amendment to state that “No single person will be allowed more than 15 votes, including personal votes and institutional votes from no more than three institutions”.

[The **amendment** was not supported by **five seconders**.]

**Schori** asked about Rec. 7A, saying if “geographically balanced” was added, there should also be language to indicate that a gender balance should be sought.

**Knapp**, not speaking as the President, thought this was a great idea.

**Schori** noted that it may not always be practicable.

[The **amendment** was supported by **five seconders**.]

**Applequist** said at this point, that her primary biological qualification for committee membership was a heartbeat, and that she would consider a vampire if they had published on nomenclature. [*Laughter*]

[The **amendment** was **accepted**.]

**Wilson** wished to raise a couple of matters on behalf of the General Committee. In Prov. 7.11, the figures suggested voting three times if a specialist committee was unable to make a recommendation. The feeling in the General Committee was that this should not be specified, because each vote took effort and time. If the specialist committee had problems making a recommendation after two votes, in general they were not going to change their minds. She noted that one Secretary had said to her, as Secretary of the General Committee, “Don’t send it back to us, because we will not agree still”. To avoid this, Wilson proposed the more general statement, “If a specialist committee is unable to make a recommendation after voting at least twice…”.

[The **amendment** was supported by **five seconders**.]

**Sennikov** proposed an amendment to just say “twice”, otherwise there was an implication that things could be voted upon indefinitely.

[The **amendment** to the **amendment** was considered **unfriendly** and was not supported by **five seconders**.]

**Thiele** supported the spirit of Wilson’s amendment, but thought it introduced a logical problem in the sentence. The important part of the sentence was the last part: the committee was considered to have recommended against the proposal, and there were an indefinite number of votes after which the committee was considered to have recommended against.

[The **amendment** was **accepted**.]

**McNeill** stated that there was a serious conflict in Prov. 7.11, beyond the editorial matter that had just been raised by Thiele. The first part, “the committee is considered to have recommended against the proposal”, was fine, but he asked what “or against making a binding decision” meant. In the case of Art. 53.5 proposals, it meant that the words were taken literally: if in fact there was one letter difference between the names involved, then those names were not homonyms. In the case of binding decisions under Art. 38.4, not making a decision was ducking the issue, and did not solve the problem. There was a default situation with Art. 53.5: if there was a single-letter difference, those names were not homonyms. But in the case of Art. 38.4, where one was considering whether or not there was a validating description, there was no default situation.

**Barrie** said he also had a problem with that part, but he did not think his solution would solve the issue raised by McNeill. One of the problems was that it said the committee had made a decision. Taking into account the way the committees had been functioning over the last six years, there were many times when the specialist committees made no decision, and they would dump it in the lap of the General Committee. He had been going to propose changing “against making” to “not to have made” but felt this was a neutral change so he would not pursue it.

**Turland** wished to respond to McNeill’s concern about not making a decision, saying it was deliberate in the proposal to require the specialist committees first to vote on whether they would make a binding decision or not. Otherwise, if a request for a binding decision on homonymy or on valid publication was submitted and received by the committees, then a decision had to be made and had to appear in one of the relevant Appendices.

In some cases a request for a decision may be clear-cut: when it was obvious if a name was validly published or was a homonym, the column editor of *Taxon* would communicate with the individual(s) who had requested the binding decision. An agreement could be reached, and the authors might withdraw the request. Otherwise, a decision had to be made and listed in the relevant Appendix of the *Code*. The idea was to enable the committees to decide whether a case was uncertain and required a decision.

**Greuter** thought McNeill’s concern was a pseudo-concern. The initial assumption was that the phrase being discussed was dealing with binding decisions, but it was dealing with whether to issue a binding decision. No supermajority was required to declare if two names should be treated as homonyms or were validly published. In fact, the closer the vote in the committee, the more urgent it was to have a binding decision. Binding decisions took a simple majority and were only non-conclusive if they ended in a tie. The indefinite number of rounds of voting would not change this situation. Instead Greuter proposed to amend the phrase, “the committee is considered to have recommended against a proposal or against making a binding decision” to “the proposal goes to the General Committee without a recommendation from the specialist committee”.

**Barrie** agreed that this was also his intent and it reflected current practice.

[The **amendment** was supported by **five seconders** and was **accepted**.]

**Wilson** now proposed an amendment to Prov. 7.12 that aimed to give the General Committee the same option to vote at least twice to approve a recommendation, rather than just having to approve a recommendation or send it back. Currently there were three alternatives: approve a recommendation in the General Committee from one of the specialist committees, reject the recommendation of the specialist committee, or send it back. The proposed wording only allowed the General Committee to approve it or to send it back. In some cases, there were reasons to reject a proposal, particularly those that had come with no recommendation from the specialist committee, where there would have been no point sending it back to the specialist committee because they were so strongly divided.

The amended wording for Prov. 7.12 was as follows (new text in italic, deleted text in strikethrough): “The General Committee may approve *or not approve* a recommendation of a specialist committee provided that a qualified majority (at least 60%) of the General Committee members supports the recommendation. In this *either* case, the General Committee makes its own recommendation, which is subject to the decision of a later International Botanical Congress (see also Art. 14.16, 34.2 and 56.4). If the required majority is not achieved *after voting twice*, *the General Committee is considered to have recommended against the proposal or against making a binding decision. The General Committee may also decide to refer* the matter is referred back to the specialist committee for further consideration.”

[The **amendment** was supported by **five seconders**.]

**Wilson** emphasized that this reflected current practice, which had been working well over the last few years. If every proposal or binding decision had to be sent back to a specialist committee there would be a revolt, so having the option to not approve was important.

**Turland** asked what Wilson meant by “not approve”: to overturn or just not approve?

**Wilson** said she had originally suggested “overturn”, but it was suggested by members of the General Committee that “not approve” was better.

**Turland** explained that the General Committee required a 60% majority to approve or reject a recommendation from one of the specialist committees. The phrase “not approve” could mean a limbo state in which it was not approved with a 60% majority but was not rejected with a 60% majority.

**Wilson** said she would be happy to see “overturn” instead of “not approve”.

[This was **accepted** as a **friendly amendment** to the **amendment**.]

**Greuter** did not see why this should not be parallel to the last sentence of the previous paragraph, and suggested as a friendly amendment adding the words “at least”, i.e. “If the required majority is not achieved after voting at least twice”.

[This was **accepted** as a **friendly amendment** to the **amendment**.]

**Greuter** went on to suggest adding “The proposal goes to the Nomenclature Section without a recommendation from the General Committee”.

[The **amendment** to the **amendment** was considered **unfriendly** and was not supported by **five seconders**.]

**Barrie** added that he approved of the change because it reflected how the committees had been working.

[The **amendment** was **accepted as amended**.]

**Price** brought up the renaming of Permanent Nomenclature Committees to “specialist committees” in Prov. 7.1 versus “Special Committee” [which would be renamed to “Special-purpose Committee”]. She asked for an explanation from the Special Committee on By-laws on the choice and the reasoning behind the change. She foresaw confusion as these terms were very similar.

**Turland** explained that there was not an official collective term for the five committees that dealt with taxonomic groups and fossils. They had been informally referred to as “group committees”, but it was pointed out that “group committee” was a meaningless phrase, because a committee was a group of people, and it was not clear what “group” meant in this context. It referred to “taxonomic group”, but the phrase “group committee” was not self-explanatory in any way. There was a suggestion “taxonomic group committees” could be used, but fossils were not a taxonomic group. The existing Special Committees should therefore be renamed to Special-purpose Committees. It may seem slightly confusing because the existing term was changing, but, in looking to the future, the Special Committee members felt that “Special-purpose Committee” described the kind of committee that was established by one Congress to report back to the next on a particular issue; “Special-purpose” here had an inherent meaning. “Specialist committees” reflected specialization in taxonomic groups or fossils.

**Funk** asked what was wrong with Permanent Nomenclature Committee.

**Turland** said there was nothing wrong with it, and it was also in the proposed Div. III as the umbrella term for all the Committees, including the General Committee, the Editorial Committee and the new Committee on Registration.

**Funk** noted that there would now be two more permanent committees, which were not nomenclature committees. She suggested “Permanent Committees”, and then underneath that, “Permanent Nomenclature Committees”.

**Turland** explained that the reason for “specialist committees” was that there was a collective term for all the Permanent Nomenclature Committees, but a collective term was also required for the five committees that look at algae, vascular plants, bryophytes, fungi, and fossils.

**Dorr** risked being accused of wordsmithing but suggested changing it to “including five committees with special remits”. He explained that the Committees had a special purpose but were not specialist committees.

**Herendeen** said if there was a sentiment to go back to taxonomic committees, the fossil Committee could simply be called Nomenclature Committee for Fossil-Taxa. That might take care of the problem. He pointed out that one of his Committee members had a cow, to use a technical term, about “Nomenclature Committee for Fossils”, because she thought it implied that the members of the Committee were fossils. [*Laughter*]

**Wilson** pointed out there were currently eight Committees, but the Registration Committee would soon be added as a Permanent Committee. She proposed an amendment to change the title of the whole group to “Permanent Committees”, and then refer to five “specialist nomenclature committees”.

**Knapp** clarified, saying the new phrasing of Prov. 7.1 would be “There are 8 Permanent Committees, including 5 specialist nomenclature committees” noting that including the Registration Committee would be completely editorial, because that was something decided before the Special Committee on By-laws created their proposal.

[The **amendment** was supported by **five seconders**.]

**Turland** wished to note, before the Section began discussing the amendment, that if the proposed Div. III was passed with this amendment, then throughout the *Code*, “Permanent Nomenclature Committees” would be referred to as “Permanent Committees”, and wherever “Permanent Nomenclature Committees” were mentioned, instead of referring to “specialist committees”, they would be referred to as “specialist nomenclature committees”.

**Wilson** thought Herendeen was also suggesting rather than “specialist nomenclature committees” the term “taxonomic committees”.

**Turland** agreed that Herendeen’s suggestion of “taxonomic committees” would be self-explanatory if Herendeen was happy with fossil-taxa coming under that umbrella group.

[The **amendment** to the **amendment** was accepted as **friendly**.]

**Kirk** said that this would mean that the mycologists would have to change their acronym from NCF to TCF.

**Hawksworth** stated that his understanding was that the *Code* was not to do with taxonomy at all, so this was a rather interesting change, and he strongly opposed it.

**Price** explained that her original comment was due to the proximity of the words “Special-purpose” and “specialist”. She suggested taking off the word “specialist” and just having “nomenclature committees” and then taking off “taxonomic”. This would put it back to the original “nomenclature committees”. They had “Permanent Committees” for the nine that would be formed. The word “specialist” could be dropped because that was obvious. It could go back to “nomenclature committees”, and there would be no confusion during the proceedings between “specialist nomenclature committees” and “Special-purpose Committees”.

[The **amendment** to the **amendment** was accepted as **friendly**.]

**Miller** asked if committees four through eight [Prov. 7.1 clauses (4) to (8)] were to be called “Nomenclature Committees” and asked how they would be distinguished from the General Committee and the Editorial Committee, which were also about nomenclature. They were looking for an encompassing term for specialized groups of organisms.

**Turland** said it was a general term, and that the Section might be in danger of covering the same ground covered in the Special Committee on By-laws. This had been discussed at great length, and it seemed that the Section was going over it all again here. He thought that some significantly new ideas were required, or perhaps consideration of what the Special Committee on By-laws originally suggested, which had been arrived at after a lot of discussion.

**Marhold** said he did not like “Permanent Committees” because outside the context of the *Code* there were permanent committees in IAPT, in some other places, and permanent nomenclature committees. These were all dealing with nomenclature.

**McNeill** said he had initially shared the concerns regarding confusion between “specialist committee” and “Special Committee”. “Special-purpose Committee” had been an improvement and reduced the conflict. After discussions with Turland, he concluded that there was no ready solution. The name that he always used for the committees dealing with particular [taxonomic] groups was the “Permanent Nomenclature Committees for particular groups”, but this was far too long when they were being discussed generically. He felt that the original wording of the proposal, “specialist committee” and “Special-purpose Committee”, was the best solution and he was thus speaking against the amendment.

[The **amendment** was **rejected**.]

**Funk** announced, before the morning break, that it was the last chance to be considered for any of the Permanent Nomenclature Committees.

[*The Section broke for morning tea.*]

**Knapp** welcomed everyone back from tea, noting that someone had pointed out to her that she had broken her own rule and had actually expressed an opinion, for which she apologized.

**Wilson** asked for an explanation from the Special Committee on By-laws why the term “supermajority” had been changed to “qualified majority”, because she thought “qualified majority” was less explanatory than “supermajority”.

**Knapp** proffered the answer, as the procedural expert in the room: because that was the correct term under most sets of procedural rules.

**Cantrill** called the question.

[The Section voted **to vote**.]

**Div. III, Prop. B** was **accepted as amended**.

**Div. III, Prop. C** (21: 42: 2: 1)

**Turland** informed the Section that this proposal was to amend Division III of the *Code* so that proposals on matters relating solely to names of organisms treated as fungi were dealt with by the Fungal Nomenclature Session of an International Mycological Congress [IMC]. The General Committee did not support the general principle of Prop. C, in particular certain paragraphs that affected the General Committee. However, the Nomenclature Committee for Fungi supported it. In the Tuesday morning session there had been a general discussion on fungal governance and there had been a paper published in *IMA Fungus* [Miller & al. in IMA Fungus 8: (9)–(11). 2017 https://doi.org/10.1007/BF03449429], in which there was a proposal for an amendment to Prop. C.

**May** reminded the Section that he had presented the case for this proposal in the Tuesday morning session. He wanted to emphasize that the mycologists wanted to take up the collegial spirit in which these Sections were held and replicate that at a Mycological Congress to deal with all sorts of issues, under a clear set of procedures, and in the spirit of improving the *Code*.

He quoted, with great respect to the President, a comment Knapp had made the other day when discussing some matters specific to fungi, “I didn’t understand any of what you were talking about”. From time to time there were matters in the *Code* that were specific to fungi, which arose from their different fundamental biology and different state of taxonomic understanding. These matters required differences in the *Code*. A number of those differences were in the *Code*, some long-established, others more recently introduced. It would only be these matters that would be dealt with. One of the amendments that would shortly be moved would clarify this concept of moving the fungi-specific matter into what could be called Chapter F, and that would be what mycologists were dealing with, not the rest of the *Code*.

Apart from that, regarding the General Committee vote on Prop. C, he thought they were perhaps concerned with some of the more technical aspects of the involvement of the General Committee in the procedures that had been outlined. He welcomed discussion about these technical aspects, and if there was any way that the proposal could be improved, the Special Subcommittee [on Governance of the *Code* with Respect to Fungi] that had generated the proposal would be very glad to hear suggestions.

Finally, in preparing this proposal, the wording of Div. III Prop. B had been exactly mirrored, and of course, if any of that wording had changed as a result of the previous discussions, it would be changed accordingly in this proposal. He had not been able to find anything in the amendments discussed for Div. III Prop. B that changed the current proposal. May emphasized that everything in the proposal regarding procedures replicated what was in Div. III Prop. B. There were two things that they had done differently: there were no institutional votes, and they had changed the names of the officers. There was a Fungal Nomenclature Bureau, but the names of the officers had changed, so that there was no confusion between a Secretary of a Fungal Nomenclature Session and a Rapporteur-général of a Nomenclature Section, but these were matters that could be easily changed if people so wished.

**Wilson** apologized if the General Committee vote had unduly influenced the general voting, because the Committee was divided. The vote was 8 for, and 14 against, with 3 abstentions. There was a simple majority against the fungal governance proposal, but there was a strong minority that did support it, and it was the question of complexities that certain Committee members were worried about.

**Schori** added that her mycological colleagues at the USDA were strongly against this proposal because they saw it as going down the road to having a separate *Code* for fungi, which they did not support.

**De Beer** wished to point out two facts. The first was that at this meeting, they had taken a photograph of the 16 mycologists present, some of whom were students. They had debated and discussed very complex issues relating to fungal nomenclature, of which most people in the audience had absolutely no working experience. What the mycologists were asking for was to take only these issues to a group of people who had that working experience. The second point he wished to make was that the Articles to be dealt with at the IMC took up less than three pages of the *Code*. All the rest, and any issues pertaining to those, would be dealt with at the IBC.

**Applequist** noted that if all the fungal-only material from the *Code* was segregated into a chapter, it would include many things that did not necessarily pertain only to fungi, such as mandatory registration, and effectively “names in current use”. Now it included the requirement that to be legitimate, a name must not be a [later] homonym of a zoological or a prokaryote name, which contradicted one of the fundamental principles of the *Code*, but at least it had been voted on by the Section. There was a mention of an amendment that would prevent other such changes to the fundamentals of the *Code* from being placed into the fungal chapter and she would like to see the details.

**Knapp** clarified that such an amendment had not yet been proposed.

**Wiltshire-Hawksworth** said there was no doubt that fungi were very different from plants. Speaking as a botanist with a foot firmly in the mycological camp she understood the terminology and the differences between plants and fungi.

**Knapp** “Unlike me” [*Laughs*]

**Wiltshire-Hawksworth** continued that there were many botanists at the Section who did not have a clue about fungi. She cited the article from *IMA Fungus*, and proposed a friendly amendment: “the Section instructs the Editorial Committee to bring together all material relating only to fungi into a separate section or chapter within the *Code*, and that this section be subject to modification only by the International Mycological Congresses operating as proposed by the Special Subcommittee on the Governance of the *Code* with Respect to Fungi.”

[Because not all members of the Subcommittee were present, the **amendment** was considered **unfriendly**; it was supported by **five seconders**.]

**May** suggested that an appropriate place for that amendment was in Art. 8.1, after “For proposals relating solely to names of organisms treated as fungi…” and, to assuage some of the concerns that had been introduced, with “but excluding any other material” added in parentheses. Then the two groups would run on to the same procedures.

[May’s suggestion was treated as a **friendly amendment** to the **unfriendly amendment**].

**Thiele** asked for an opinion of the Rapporteur-général as to whether this was a straightforward thing to do.

**Turland** said the short answer was, yes.

**Knapp** told him that short answers were good. [*Laughter*]

**Turland** stated that the Vice-rapporteur, the President and himself had looked into exactly which provisions of the *Code* would need to be moved into Chapter F, and it was straightforward to identify those that pertained solely to fungi. At least 90% of the provisions of the *Code* pertained to algae, fungi, and plants, so this proposal only pertained to the provisions that related solely to fungi, and it was simple to move them.

**Knapp** asked for clarification from the proposer if Articles outside Chapter F could be modified at an IMC.

**May** confirmed that the answer was no. Articles that were outside Chapter F, containing material that mycologists wished to propose a different way of dealing with things i.e. solely to do with fungi, could be moved into Chapter F. For example, this week the Section had introduced new rules for the registration of later typification acts for fungi. That, said May, was what they wished to do at an IMC: taking an existing procedure and modifying it for fungi. He thought everyone would agree that this was an innovative and useful thing to do. They would not be able to make any changes to the rest of the *Code*, as it pertained to all the organisms that were treated under the *Code*.

**Turland** clarified that in a situation such as May described, the rule pertaining to registration of later typifications of names of fungi would be in Chapter F. An existing rule that formerly applied to algae, fungi, and plants would, of course, remain in the main body of the *Code*, but would no longer apply to fungi.

**Applequist** said May acknowledged that the IMC would have the power within the fungal chapter to add any new requirement whatsoever or suspend any principle of the *Code* whatsoever, if it applied only to fungi. She thought that would effectively create a “MycoCode” within the *Code*. If their values and interests diverged from the rest of the community, that chapter would become increasingly divergent from the rest of the *Code*, and the community would end up with a “*Code* and a half”. It gave the mycologists great power, since there would be no way to try and steer their chapter back towards the principles of the *Code*, whereas they could continue to move the *Code* toward the “MycoCode”.

**May** insisted there would be no “MycoCode”, adding that if it would assist the Section in retaining the confidence of mycologists, direct reference in the Preamble to the inability of an IMC Nomenclature Section to alter anything could indicate that mycologists did not want to diverge from the overall spirit of the *Code*.

**Greuter** asked the authors of the amendment if anything would preclude retaining the functional integrity of the *Code* by duplicating fungal provisions both in the body of the *Code* and in Chapter F.

**May** answered that cross-references would be expected when material was in two different places, as relevant. Should new material appear in Chapter F that related to other existing Articles of the *Code* before an IBC Nomenclature Section, cross-references in the online *Code* would be useful, but would disappear after each Nomenclature Section of an IBC. He wished to assure everyone that the *Code* would not gradually accumulate cross-references into Chapter F, because the existing Articles would just be rewritten editorially to indicate what was covered.

**Schori** asked for some clarification regarding a situation in which, for example, there was something only in the chapter dealing with fungi, such as registration, and the next Congress voted for registration for all groups treated under the *Code*. She wondered whether the portion in Chapter F that said registration was only for fungi would remain there until the next IMC decided to alter that chapter. Would the Editorial Committees for that chapter and the rest of the *Code* work between Congresses to keep up with changes?

**Turland** said the Editorial Committee was able, willing and ready to work between the IBC and IMC to ensure that the *Code* remained fully up to date. The print edition of the *Code* would contain an indication for mycologists to check the online version for any updates that might appear as a result of an IMC.

**Wiltshire-Hawksworth** said that Applequist seemed worried that a separation of fungi might affect the *Code* as it existed. However, there had been an admission that botanists did not understand the issues and the complexity involved in the mycological disciplines. She did not understand, therefore, how they could vote either way on any issue that involved fungi.

**Herendeen** called the question.

[The Section voted **to vote** on the amendment, and the **amendment** was **accepted as amended**.]

**Applequist** wished to respond to Wiltshire-Hawksworth’s comment that botanists did not understand the life cycle of fungi and so forth. While that was frequently true, the life cycle of fungi was not going to change much in the future. There were provisions in the *Code*, which the mycologists had asked for and had been given, without problems. She felt the botanists were as capable as the mycologists of understanding the issues involved in registration or harmonization with the zoological *Code*, or redefinition of types. As such, botanists should have some say in what went into their *Code*; otherwise there would be two *Codes*, not one.

**Knapp** apologized, saying she had inadvertently started this entire line of discussion by admitting she did not know something. She thought it was better to admit when you didn’t know something rather than to pretend that you knew.

**Redhead** spoke for the members of the Nomenclature Committee for Fungi saying they had no desire to start a “MycoCode”. This proposal was not a slippery slope, but rather a barrier towards that. He added that these committees would have to continue attending the IBC because the bulk of the *Code* would not be just restricted to fungi and they would want a say in how the rest of the Articles and proposals took place. He reassured the Section that they could expect to see a small expert group of mycologists continuing to attend the IBC to have input and feedback. This change was just to facilitate being more democratic as far as the numbers of specialists in mycology dealing with mycological issues.

He continued by noting that earlier in the week there had been a discussion that implied that somebody might have to read the zoological *Code* and there had been a great reluctance to do that. As an expert in nomenclature for mycology, he had been forced to learn how to use the zoological and bacteriological *Codes*, so that he could see all the pitfalls and traps and things that overlapped between them. Mycology was such a special area because they had organisms that had drifted in from these so-called kingdoms.

**Groom** freely admitted to not understanding fungal nomenclature and that was why he went to all the mycologists in his institution to get their opinion on this proposition. They did not support this position, and while he did not fully understand why not, he would cast their vote accordingly.

**Gereau** said that the Section was being asked to create a new Chapter of the *Code* to contain all matters pertaining to one group of organisms, which may be workable. There was also the issue of the botanical and mycological Congresses not being coordinated, which caused a lot of complication for the Editorial Committee, but the Editorial Committee had indicated it may be workable. The Section had been given the assurance from May that even with this *Code*-within-a-*Code* the mycological community fully intended to observe the spirit of the rest of the IBC. He asked about the willingness of the mycological community to observe the letter of the rest of the *Code*. For example, during the discussion of Art. 8 Prop. O [to allow DNA sequence data to serve as the type of a fungal name], at least one individual apparently intended to disregard the results of that vote, because six years was too long to wait. He asked what assurances could be given that this sentiment was not widely shared among the rest of the community.

**Knapp** clarified Gereau’s comment, noting that he had said “all matters pertaining to fungi”, but it should be matters “solely related” to fungi.

**Hawksworth** drew attention to the fact that these issues had been debated for a long time, starting in 1971 at the first IMC, at the last two IBCs, and at several Mycological Congresses. These were firm proposals backed by the International Commission on the Taxonomy of Fungi and the International Mycological Association. He cautioned that, if the community did not want a “MycoCode”, they had to approve this proposal.

**May** addressed the comments from Gereau, wishing to indicate the seriousness with which the Special Subcommittee had treated this in terms of replicating the procedures. The proposal had a Bureau of Nomenclature [the Fungal Nomenclature Bureau], it would also engage the General Committee at several points in the consultation. For example, when deciding what matters were solely related to fungi, there would be consultation with the General Committee. There would also be consultation under “Election and role of Chair of the Fungal Nomenclature Session”. When the Fungal Nomenclature Session authorized Special-purpose Committees, this would be in consultation with the General Committee. The Chair of the Fungal Nomenclature Session would be elected by the Nomenclature Committee for Fungi in consultation with the General Committee. There were anchoring points that meant that the significant office bearers and the procedures were in the collegial spirit that existed at this Section. Similarly, under the “Election and role of Deputy Secretary”, there would be consultation.

Just as in the Botanical Congress Nomenclature Section, the Rapporteur [i.e. Secretary] would be elected by the preceding Congress. They would not have a preceding Congress for the next Mycological Congress [2018], and in the absence of any other procedure, he suggested that, should there be a Nomenclature Session of the IMC, its Secretary should be elected by this [Shenzhen Nomenclature] Section. That would anchor the procedures and provide continuity. Within the Nomenclature Committee for Fungi, within the IAPT, within the IMA, there was a wish to replicate an IBC at an IMC. The overwhelming wish was to take the fantastic way that the botanical community have been able to modify and improve the *Code* and reproduce that. He hoped that next year they would be sitting around in Puerto Rico and the room would look the same and have the same spirit.

**Geltman** shared his exchange of opinions with mycologists of the Komarov Botanical Institute: there were several opinions, but generally they were against mycological separatism. They thought that if what was being discussed now were to be implemented, it would be the first step to a separate “MycoCode”.

**Gandhi** noted that despite his not being a mycologist, he addressed the fungal problems for his department. He supported the proposal but was concerned that the mycologists would have a say in the general part of the *Code*, whereas botanists would not have a say in the fungal part.

**Knapp** asked if botanists would be welcome at Fungal Nomenclature Sessions. [*Laughter*]

**May** said they absolutely were and pointed out that the proposed changes included cordially inviting the Rapporteur-général in office, or their delegate, to attend the Mycological Nomenclature Session. In fact, Turland had attended the last International Mycological Congress.

**Watson** noted that the General Committee decided which proposals dealt solely with fungi. He asked if provisions resulting from such proposals could be brought back into the main part of the *Code* if, at some point in the future, they no longer dealt solely with fungi. He mentioned, for example, registration.

**Turland** explained that this would be editorial. If there was a provision in Chapter F that related solely to fungi, and it were modified in such a way by the Nomenclature Section that it no longer only applied to fungi, but to other organisms, then it would come back into the main body of the *Code*.

**Greuter** suggested that the functional integrity of the *Code* should be preserved. He thought the Editorial Committee might consider duplicating some provisions that were needed in the context of the body of the *Code* for a fuller understanding. He understood that the Editorial Committee would be perfectly authorized to do this. He wanted to ask, however, about the involvement of structures of the botanical *Code* in the new additional tasks of fungal nomenclature. He asked if IMA or IUMS would fund additional Editorial Committee meetings or attendances of the Rapporteur-général at IMCs.

**Knapp**, while appreciating the sentiment, said that this was off the topic that was being debated, which was whether the Section would accept Div. III Prop. C.

**Herendeen** called the question.

[The Section voted **to vote**.]

**De Lange** requested this be a **card vote** [instead of a show of hands], so that the views of institutions could be represented.

**Knapp** allowed card vote number 8, reminding attendees that any cards with the wrong number on them would be discarded.

**Div. III, Prop. C** was **accepted as amended** based on the **card vote** (346 yes: 180 no; 65.8% yes).

[*Discussion moved to Art. H.5 while the results of the card vote were being tallied*.]

#### Article H.5

**Art. H.5, Prop. A** (10: 0: 48: 6) was **automatically** sent to the **Editorial Committee**.

#### Recommendation H.5B (new)

**Rec. H.5B (new), Prop. A** (28: 12: 15: 6)

**Turland** explained this was, until recently, the hybrid appendix but, as he had pointed out to Knapp yesterday evening, the *Code* had had its appendix out. [*Laughter*]

**Turland** explained that this proposal addressed a problem that could arise when only one nothotaxon between two species was known and at least one of the parent taxa was at an infraspecific rank. In a case like this a nothospecific name for the nothotaxon would be inappropriate to its hybrid formula and therefore incorrect in relation to that hybrid formula.

The proposed Recommendation was sound advice and accorded well with the advice that was already given in Rec. H.10B.1. The current wording of the proposal did not allow for the parent taxa to be at the same infraspecific rank, but this would be something that would be resolved by the Editorial Committee if the proposal were accepted.

**Greuter** opined that the proposal was inappropriate because it recommended against giving a name to a recognized taxon. He did not think the *Code* should advise against giving names to taxa that were recognized and accepted.

**Gandhi** said he sometimes came across new hybrid names based on parents of unequal rank. While such names were incorrect, it was not clear how to proceed, and he was against the proposal.

**Rec. H.5B (new), Prop. A** was **rejected**.

#### Article H.6

**Art. H.6, Prop. A** (27: 24: 4: 5)

**Turland** said the purpose of this amendment was to bring Art. H.6.2 in line with Art. H.6.3 and Art. H.6.4, so that all nothogeneric names would be restricted to a maximum of eight syllables.

**Applequist** pointed out this only applied to two existing names, which were not particularly long in terms of alphabet and easy enough to pronounce. She asked why new names should be published for them when they were not causing problems.

**Art. H.6, Prop. A** was **rejected**.

**Art. H.6, Prop. B** (34: 4: 17: 6)

**Turland** explained that this proposal sought to permit nothogeneric names of bigeneric hybrids that included a hyphen to be validly published, with the hyphen to be deleted. The Rapporteurs noted that the wording should be somewhat adjusted because some of these names were published with both a hyphen and a connecting vowel and they gave a few examples.

**Gandhi** said he came across such names with a hyphen and was confused as to whether to delete or to keep it. After discussing it with a few people, he decided to delete the hyphen, so he supported this proposal.

**Art. H.6, Prop. B** was **accepted**.

#### Glossary

**Glossary, Prop. A** was discussed under **Art. 36, Prop. D**.

**Glossary, Prop. B** (3: 8: 65: 0), **Prop. C** (10: 25: 40: 0), **Prop. D** (1: 3: 72: 0), **Prop. E** (1: 23: 51: 1), **Prop. F** (2: 2: 72: 0), **Prop. G** (2: 8: 66: 0) and **Prop. H** (2: 2: 72: 0)

**Knapp** reminded the Section that on the first day they had voted to refer all the proposals to amend the Glossary directly to the **Editorial Committee**.

[*At this point the results of the card vote on Div. III, Prop. C were received and discussion returned to Div. III*.]

#### Division III (continued)

**Div. III, Prop. D** (20: 44: 1: 1)

**Turland** noted that this proposal would amend Div. III of the *Code* so that the Nomenclature Committee for Fungi was elected by an IMC. The General Committee did not support the general principle, but the Nomenclature Committee for Fungi did.

**May** pointed out that the proposals that went to the NCF would continue to be handled through the existing procedures. They would go to the General Committee and then come to the Nomenclature Section of an IBC, because this would just be replicating the procedures across the other committees. The NCF would decide whether to recommend proposals. Their recommendations would go to the General Committee and because this was an overarching committee across all the specialist committees, their decisions would go to the Nomenclature Section of an IBC. Those decisions were not going to an IMC, but in terms of the composition of the NCF it would be appropriate for that body to be elected at an IMC.

May moved an amendment, saying that the very last bit of the amendment, that the Nomenclature Committee for Fungi, “includes the Secretary and the Deputy Secretary of the Fungal Nomenclature Bureau as non-voting ex-officio members”, would actually rule out those people as being able to serve on the Committee and, given the smaller pool of people that would be available to draw from, it would seem appropriate to add at the end of that sentence, “if they are not already members of the Nomenclature Committee for Fungi”.

[The **amendment** was supported by **five seconders** and was **accepted**.]

**Wilson** wanted to note that, contrary to Turland’s introductory verbal comment, the General Committee did not express the opinion that they did not support the proposed method by which the Nomenclature Committee for Fungi was appointed. The General Committee did not specifically vote on that issue; they voted generally on the whole principle of these fungal proposals.

**Turland** acknowledged the mistake.

**Hawksworth** said one of the motivations for the proposal was to get new, younger people and a better cross-section, both geographically and gender-wise, to come forward. They sometimes had great difficulty selecting people, and the fact that people could now make proposals at the IMCs could lead to the involvement of a broader spectrum of participants. They would not propose changing the agreed composition of the Committee next year, as they had only just been appointed. They would have to get the IMA Nomenclature Session to approve what was there, but perhaps take one or two extra people on, if the Committee thought that was desirable.

**Div. III, Prop. D** was **accepted as amended**.

#### Floor proposals

[See **Appendix A** for the texts of these proposals.]

**Knapp** moved on to the proposals made from the floor of the Nomenclature Section.

**Floor Prop. 1–3, Sennikov** [To convert the footnote of Art. 8.1 into an Article defining an illustration for the purpose of typification; the **proposal** was supported by **five seconders**.]

**Turland** introduced the first proposal from the floor by reminding the Section that on Monday, the first day of the Section, Art. 8 Prop. N by Sennikov was deferred and the proposer was asked to replace or reformulate it and come back with a proposal from the floor. Sennikov had withdrawn the original proposal and these proposals were the replacement for it. They were to add a new Article to Art. 8 and three Examples, which would be sent to the Editorial Committee if the proposal was accepted.

**Sennikov** stated that he had split his former proposal into three steps. The first step (Prop. 1) was the core of the proposal and offered the main definition of what was an illustration and what was supposed to be designated as the type. The second step (Prop. 2) offered the restriction to the wording, “If an illustration comprises more than one element…” and defined what kind of elements were acceptable. The third step (Prop. 3) was separate but complementary.

**Knapp**, having gained agreement from Sennikov, ruled that the three “steps” would be voted on as one proposal.

**Redhead** wished to know, before starting, how many proposals from the floor there were, and for which Articles, in case there were competing proposals for Art. 8.

**Knapp** stated that there were 15 proposals and there were no competing proposals for any Article.

**McNeill** was puzzled as to the total effect of Sennikov’s proposal. He wondered if it deleted the footnote to Art. 8.1, which defined an illustration, saying “Here and elsewhere in this *Code*, the term ‘illustration’ designates a work of art or a photograph depicting a feature or features of an organism, e.g. a picture of a herbarium specimen or a scanning electron micrograph.”

**Sennikov** clarified that the footnote would be deleted.

**Nakada** said he strongly supported the proposal because it was in conformity to the microalgal methods. He proposed a friendly amendment adding “excluding movies”. [*Laughter*]

**Sennikov** did not believe multimedia could fall into the definition of an illustration; he considered the **amendment unfriendly**.

[The **amendment** was not supported by **five seconders**.]

**Redhead** proposed an amendment to add “and anatomical” in additional to “morphological”.

[The amendment was **accepted** as **friendly**.]

**Gandhi** asked for the Rapporteurs’ comments on each of the proposals from the floor.

**Knapp** explained that because the proposals were only received at 6 o’clock the previous evening the Rapporteurs did not have a chance to craft comments.

**McNeill** said while there was a slight rewording to the previous footnote, the addition was a Recommendation and did not really belong in the Article.

**Turland** responded to Gandhi saying that, compared with the original wording of Art. 8 Prop. N, there was something new: “referable to a single taxon”. He wondered how a photograph of a single taxon could be taken, except from a super close-up. There could be a considerable amount of admixture.

**Sennikov** said that the addition of the words “and depict one or more morphological and anatomical features of an organism” may get around that. If the photograph was just something in the landscape, it did not qualify, because it was not intended to show any morphological features.

**Applequist** pointed out that if the statement that there should be a single plate was “should” rather than “must”, it was not mandatory, and two paintings on successive pages, or two plates of photographs, could be called an illustration.

**Gereau** could see no improvement to the existing footnote and felt that the extra specificity made it overly restrictive and hard to apply; it should be rejected.

**Thiele** drew attention to one significant restriction: the proposal specified that an illustration for typification must depict one or more morphological or anatomical features of an organism. The third example under the original proposal removed a loophole in the current *Code*, which had allowed some fungi to be typified on a picture of a DNA sequence, with the argument that this was an illustration representing a feature of an organism.

**Barrie** noted that the *Code* and the Glossary defined an illustration as an element, so an illustration could not be composed of elements.

**Floor Prop. 1** and **Prop. 2** were **rejected**; the dependent **Prop. 3** was **withdrawn**.

**Floor Prop. 4, McNeill** [To clarify the meaning of “used only one element” in Art. 9 Note 1; the **proposal** was supported by **five seconders**.]

**McNeill** said the reasoning behind the proposal to amend Art. 9 Note 1 was that the word “used” had been misinterpreted as implying “mentioned in the protologue”. The proposal intended to make clear that such an interpretation, which was hard to believe in terms of the meaning of the English words “to use”, was clearly disallowed. The words being inserted were “when preparing the account of the new taxon”, indicating what the original author was using the element for.

**Floor Prop. 4** was **accepted**.

**Floor Prop. 5, McNeill** [To show in Art. 9 Note 7 that a type supported by an epitype can be lost or destroyed instead of superseded; the **proposal** was supported by **five seconders**.]

**Turland** noted that this proposal would amend Art. 9 Note 7.

**McNeill** explained that this was a minor clarification that an epitype could support not only a lectotype or a neotype, but also a holotype. If the supported type was lost, destroyed or superseded, with supersession applicable generally to lectotypes or neotypes but not to holotypes, then the epitype had no standing. This was triggered by a discussion in Vienna [2005], where some people did not realize that an epitype also could support a holotype.

**Floor Prop. 5** was **accepted**.

**Floor Prop. 6, Wilson, McNeill, Mabberley, Barrie and Funk** [To clarify that Art. 14.3 does not preclude conservation or rejection of names of nothogenera, because a statement of parentage determines their application; the **proposal** was supported by **five additional seconders**.]

**Turland** introduced the proposal, which would add some words at the end of Art. 14.3: “Application of conserved and rejected names of nothogenera is determined by a statement of parentage (Art. H.9.1).”

**Wilson** explained that the General Committee had become aware of the issue of the status of names of nothogenera, and whether they could be conserved, when they were considering a hybrid name from the Committee for Vascular Plants. Art. 14.3 should not preclude conservation of names of nothogenera. Names of nothogenera could be rejected, but conservation was not possible because their application was determined by a statement of parentage rather than by typification.

**Applequist** expressed her hope that there would not be a lot of proposals to do this but asked if the proposers would accept as a friendly amendment “nothogenera or nothospecies”.

[The **amendment** was considered **unfriendly**.]

**Turland** explained that the amendment was unnecessary because names of nothospecies had types.

**Floor Prop. 6** was **accepted**.

**Floor Prop. 7, Funk, Greuter, McNeill, Malécot and Herendeen** [To define words not to be regarded as generic names by deleting Art. 20.2 and adding a new clause (c) to Art. 20.4; the **proposal** was supported by **five additional seconders**.]

**Turland** noted that this proposal was connected with Art. 20 Prop. A, discussed earlier in the week, regarding a Latin technical term used in morphology. It had been discussed at considerable length and rejected after a card vote. This proposal from the floor took a different approach, to delete Art. 20.2 and Ex. 2 to Ex. 6 and insert some text from the deleted examples into a new clause, Art. 20.4(c).

**Malécot** explained that the rationale of this proposal was to answer the question of whether a generic name could be based on a morphological term. The proposal removed Art. 20.2 and added Examples from Art. 20.2, regarding not validly published generic names, to Art. 20.4 to rule that any generic name that used these words would not be considered a generic name. The chosen words had already been used in the Examples, and a few more were introduced from pharmacopoeia terminology to prevent some uses that may occur in old literature.

**Schori** asked whether the words would be exactly as they appeared or whether they would be used in combination to form a generic name.

**Malécot** confirmed it would be the words exactly as written: for example, something like “*Floscuculi*” would be considered a generic name, but not “*Flos*” alone.

**Gereau** said that if Art. 20.2 were to be deleted without any starting date for the new provision, 105 years of names that had been declared not validly published would suddenly become validly published, and this created a much bigger problem than was solved by the proposal.

**Sennikov** proposed an amendment to vote separately on inserting this provision and retain Art. 20.2. The new wording could be inserted for the purposes of clarification on how to make Art. 20.2 workable.

[The **amendment** was supported by **five seconders**.]

**Barrie** asked for clarification that this change was to retain Art. 20.2 and delete the Examples.

**Knapp** confirmed that the amendment would retain Art. 20.2 and add words to Art. 20.4.

[The **amendment** was **rejected**.]

**Greuter** explained that the proposal sought to delete the paragraph because it was an awkward provision. Although he was not sure where it came from, it seemed that at some early stage of international nomenclature, there was a concern that several terms used in the pharmacopoeia generally, and used in designation of species of drugs in the form of Latin polynomials, might be considered to have become accidentally but validly published generic names.

There had been extensive literature in those early times about drugs referred to by their polynomials as used in the pharmacopoeia, for instance, “*Lignum*” so-and-so and “*Balsamum*” so-and-so. In a paper with such names, there were descriptive statements. The first word, which occupied, accidentally, the position of a generic name, might have been considered a generic name and was validly published by inclusion of the descriptive statement. It was this concern that had led to the Article.

Under the Article as it stood, it became clear that generic names, other than pharmacopoeial ones, were made illegal by it, such as “*Lanceolatus*” and “*Lobata*”. This was the portion that caused problems: not the pharmacopoeia rejection, but the application of the Article to the descriptive morphological terms that were thereby outlawed.

The best solution was to delete the provision and to list the names concerned, insofar as they were known, as inappropriate for generic names. This had been suggested during the discussion when the proposal was considered. The new proposal listed in clause (*c*) the two technical morphological terms that were specifically mentioned to be outlawed, “*Lanceolatus*” and “*Lobata*”, so that they did not become validly published through deletion of Art. 20.2. The proposal added a number of terms from pharmacopoeia. These were those in the earlier part of the enumeration, from “*Balsamum*” to “*Semen*”, in alphabetical order, so that they could not *post factum* become validly published generic names. Greuter concluded by saying that he would not have dared to associate himself with the proposal unless he saw no potential of destabilizing of existing names, in answer to Gereau’s concern.

**Garland** asked if the list was exhaustive or was merely a list of examples.

**Malécot** considered it an exhaustive list.

**Garland** asked if a name like “*Ovatus*” would be perfectly fine, but “*Lanceolatus*” was prohibited.

**Schori** suggested another source for the article: Stearn’s *Botanical Latin*, in which he gave the history of nomenclature and presented at one point the Linnaean canons. Included in that section of text was discouragement of using adjectives, descriptive terms or morphological terms as generic names. If Art. 20.2 was deleted, there would be nothing to prevent “*Lancifolium rotundifolium*” as a binomial.

**Hawksworth** said he was concerned about the words “some” and “widely used”, because this would mean that the case of “*Caeruleum*”, discussed earlier in the Congress, would now be acceptable.

**Gereau** called the question.

[The Section voted **to vote**.]

**Floor Prop. 7** was **rejected**.

**Knapp** announced that after lunch she would have sign-up sheets for the new Special-purpose Committees that had been established by the Section: on DNA Sequences as Types; on Typification; and on “Lists of Available Names”. She said there was also a Special-purpose Committee on Rusts [rather, on Pleomorphic Fungi], so a rusty special committee sign-up sheet was also required.

[*The Section broke for lunch*.]

### Friday, 21^st^ July 2017, Afternoon Session

#### Floor proposals (continued)

[See **Appendix A** for the texts of these proposals.]

**Knapp** stressed to delegates the importance of finishing all the business by the end of the day. She urged everyone to be brief, concise and not to repeat things that other people had said during discussions.

**Floor Prop. 8, Turland and Knapp** [To delete the words “and format [layout]” from Art. 30.2 as previously amended by the Section under Art. 30 Prop. D; the **proposal** was supported by **five seconders**.]

**Knapp** pointed out that under the rules of procedure this was what was known as a “Proposal to Reconsider”. The Section had already voted on the proposal, but she and the Rapporteur-général wanted to put this back on the floor to reconsider.

**Turland** explained that this concerned Art. 30 Prop. D, which had been accepted as amended [during the morning session on 19 July]. The amendment had been accepted by the President and the Rapporteur-général as friendly, adding the words “and format” or “and layout”, with the exact wording to be determined by the Editorial Committee, in the phrase “…its content and format [layout] is merely a preliminary…”.

At the time this was considered to be a friendly amendment, but having thought about it further, the proposers had serious concerns that it could cause problems with so-called online-first or issue-in-progress articles. What was important was that the content must not be preliminary; the content must be what the publisher considered final. There were some cases where these online-first articles had slight changes in format between the online-first version, with preliminary pagination, and the final version with the final pagination that appeared in the compiled journal issue or volume. The change might just be something in the header, or the way that the article history was presented, or the way the page numbers were shown. It could amount to a cover sheet or logos or just some minor format change. They were concerned that people may look at the amendment and say, “there was a small change in format from when it was first issued, and even if it was a Version of Record it is not effectively published.” They therefore proposed deletion of “and format”. The member of the Section who proposed the friendly amendment was concerned about the current Example in Art. 30 (Ex. 5) concerning “*Dracula trigonopetala*”. The Example said, “However, the paper is not presented in a format suited for publication in the *OrchideenJournal* and was evidently not intended for inclusion in that journal”.

Even if “and format” was taken out of Art. 30.2, this Example could still stand. One could say that it was the content of the paper that was not presented in a format suited for the journal, because this preliminary publication had been in English, and then it was later translated into German to appear in the German-language journal. It was the *content* that was not suited to publication in the journal.

**Herendeen** agreed that this was potentially confusing, and should be struck.

**Floor Prop. 8** was **accepted**.

**Floor Prop. 9, Miller** [To disallow, on or after 1 January 2019, effective publication through distribution of unpublished printed matter without an ISBN or ISSN; the **proposal** was supported by **five seconders**.]

**Turland** introduced this proposal to insert a new Article into Art. 30, following Art. 30.7 or wherever the Editorial Committee deemed most appropriate.

**Miller** said the proposal sought to place a limitation on a loophole in the definition of effectively published that still allowed effective publication with very minimal distribution of a limited number of personally printed copies. When the first definition of effective publication was adopted, one of the real pillars of the *Code*, it was left intentionally open because at that time getting printed material distributed was much more difficult. This was not the case in the modern world. The proposal intended to place a higher bar and limit the inappropriate publication of names by distributing a very limited number of copies by deposition directly into libraries.

**Applequist** asked Miller to confirm that he did not intend to imply that something that lacked an ISBN was not published.

**Miller** confirmed that he did not intend to imply that.

**Applequist** pointed out that the proposal needed to be reformatted, and that it would be banning a lot of stuff from foreign countries that the Section had previously agreed they did not want to ban.

**Miller** pointed out it did not retrospectively ban anything but established a new date where it would become a requirement.

**Applequist** pointed out that, as had been discussed earlier, such a change would be very hard for people in some countries.

**Turland** pointed out that the proposal said “unpublished printed matter without an ISBN or ISSN”, meaning that *published* printed matter without an ISBN or ISSN would not be included in this provision. It only referred to unpublished material.

**Lindon** wished to highlight potential arguments that might be made. She asked how the proposal would address preprints: for example, if a preprint was from a journal that had an ISBN, would that preprint be, by definition, unpublished.

**Miller** agreed that preprints and proofs were not published.

**Kirk** asked for a definition of the word “published”, arguing that “published” meant to make public.

**Greuter** thought the proposal was dangerous because it was too vague and contained circular reasoning. If unpublished printed matter was not effectively published, the proposal was saying that such material was not effectively published because it was unpublished.

**Miller** agreed.

**Tong** asked how a publication could be unpublished but still have an ISBN or ISSN.

**Knapp** asked Turland to explain the circumstances.

**Turland** offered a few words regarding the circumstances out of which the proposal arose. There had been situations where authors had been depositing proof copies or preprints or non-final printed manuscripts in more than one botanical library or in a botanical institution’s library. This, under the current rules, constituted effective publication and, in some cases, pre-empted the same article that was later to appear in the journal to which it was submitted and which was in the process of publication. Sometimes it would be a completely standalone publication that was just printed twice: [for example] one copy sent to the Royal Botanic Gardens, Kew and another sent to the Natural History Museum, London. The concern was that this was poor practice but was perfectly acceptable for the purposes of effective publication under the *Code.* This proposal was an attempt to try to stop such practices.

**Gandhi** said he knew of a few cases wherein the final versions from competing authors had been distributed as printed matter to a few libraries and thus had been effectively published, just in order to establish priority. It was not only a problem relating to preliminary versions.

**McNeill** was puzzled as to whether this banned all printed matter without an ISBN or ISSN. This seemed to be the only sensible meaning it had. Otherwise, anything was published once it had been distributed.

**Middleton** said the clause “without an ISBN or ISSN” was redundant, because if it had one it would not be unpublished. He thought that maybe this was the confusing element of the proposal and it should just be deleted.

[The **amendment** was considered **friendly**.]

**Hawksworth** asked if it was deliberate that the proposal did not apply to mycological institutions and their libraries.

**Knapp** said she thought it was not.

**Floor Prop. 9** was **rejected**.

**Knapp**, speaking as President, said that everyone should discourage the bad practice outlined in the previous proposal. No longer speaking as President, she asked those more versed in nomenclatural matters, what it would mean if something was given to a library but not accessioned. [*Laughter*] She asked everyone to think about this as a community. She then stated that she would resume her role as President [*More laughter*].

**Turland** commented that Knapp had created quite a frisson, and that the Section was obviously thinking about it.

**Floor Prop. 10, Geltman** [To include with theses the separately distributed abstracts thereof as not effectively published under Art. 30.8; the **proposal** was supported by **five seconders**.]

**Turland** introduced this as a small amendment to Art. 30.8, to add after the word “thesis” the phrase “(including the separately distributed abstract of thesis or similar material)”, and asked the proposer to say a few words.

**Geltman** explained that there was a situation regarding dissertations or theses in Russia and some neighbouring countries. A thesis existed only as two copies: one in the national library, and one in the institution’s library. However, it was mandatory to distribute around 100 copies of an abstract of the thesis. This proposal said that an abstract should be equivalent to the thesis, and the appearance of a name in this abstract was not effectively published.

**Sennikov** did not think this solved any problems, because the Russian abstracts had always been widely distributed, thus qualifying for effective publication. They all contained a statement from the printer and often the publisher, and therefore they qualified for effective publication and did not fall under this Article, even with the proposed amendment.

**Schori** thought it might still be important in certain cases. Sometimes a summary of papers constituted a thesis, especially in countries like Sweden. In one such summary, published before the papers, it was stated that a combination was going to be made in one of the papers. That statement had in fact made the combination there and then, creating a circular citation, which was not particularly helpful.

**Greuter** did not support the proposal, on the grounds that it was retroactive and the effects had not been duly examined. He explained that the current wording of the Article came about at the Vienna Congress [2005], with the same date of 1953, and the *Vienna Code* was published in 2006. It was widely retroactive and had rendered not validly published dozens of names that had been indexed, and used, but had referred to such dissertations without any proper screening. Now what was being proposed was that these dissertations made, in anticipation of future versions of the *Code*, a declaration of effective publication under rules that were not in existence at that time. This was not realistic and was widely unfair. Greuter thought that scores of names could be lost that had been validly published, without any proper search having been made regarding the retroactive effect of the proposal.

**Floor Prop. 10** was **rejected**.

**Floor Prop. 11, Funk, Greuter, McNeill, Malécot and Herendeen** [To widen the coverage of Art. 36.2 to pairs of alternative names that are not both new; the **proposal** was supported by **five seconders**.]

**Turland** invited one of the proposers to introduce their proposal.

**Greuter** spoke to the proposal, which concerned Art. 36.2, noting that the concept of alternative names had been traditionally applied to cases in which names were technically not alternative names. If both names were not validly published or first proposed in the same relevant publication, or only one of them was, they were alternative names, but not as defined in the *Code*. This could be circumvented by replacing “proposed” by “used”: alternative names used in the same publication and which need not both be new.

**Paton** asked for clarification: if an author was making a new combination and therefore using two names with the same type, would the new combination be not validly published? He felt that, under the proposed wording, if he were to take a previously published name of which he was the author and move it into another genus, the intended new combination would not be validly published because he would have used two names both authored by him and based on the same type.

**McNeill** suggested to Paton that this situation would be covered by the second sentence of the existing Art. 36.2.

**Govaerts** asked what would happen if a book was published by two authors and the name was used in the book, then, later, one of the authors made a new combination. How would one know if this applied? Because the new combination was made by one of the authors, was the previous use of the alternative name by both authors or was it only used by one of the authors? Govaerts wanted to know how this would be established.

**Redhead** said they had already run into such a situation where two lichenologists published names, co-authored a publication and then proposed new names in the publication where they disagreed on the taxonomy. They authored each name separately so were simultaneously publishing them, but, because they were different authors even though they were in the same publication, the names were considered to be validly published.

**Govaerts** replied that this was not what he meant. He outlined a case where one name already existed and was used in a co-authored publication, but it was not clear which one of the authors had used this existing name, and then a new combination was made in another genus by one of the authors. Was the previous use of the existing name by the author of the new combination or was it by both authors of the original publication? If that was not specified, how could one determine whether it was an alternative name or not?

**Gandhi** knew of one example in the *Apiaceae*, which would be used for *Flora of North America*, in which an author mentioned two names. One was a previously published name and the other was alternatively proposed as a new name. Until now, the newly proposed name had been treated as not validly published because a previously existing name was also cited. But following this proposal that would be wrong: the newly proposed name would become validly published.

**Paton**, returning to his previous comment, did not think the [second] sentence [of Art. 36.2] involving combinations applied if one was moving a species into a new genus, in which case the names were not at different ranks, but at the same rank.

**Applequist** tried to summarize the concerns expressed. She gave an example of an introductory portion of a revisionary paper where she referred to a species in a genus. Later, she might say she would move it into another genus and publish that new combination. But in the introductory material when talking about the prior literature one must use the name that had previously been used, therefore two names were being used in the same paper. She wished to propose a friendly amendment to change “used” to “accepted”, which might avoid that problem.

**Greuter** accepted the **friendly amendment**, saying he had been going to propose it himself. He replied to points raised by Gandhi and an earlier speaker. If the authors were different – and in the cases mentioned by Gandhi, they were – then even before this amendment, the new name was validly published because these were not alternative names because they had different authors.

**Gandhi** elaborated by saying that the names in question were by the same author.

**Hawksworth** wondered if saying “simultaneously used in different positions” or “different ranks” would get around the problem of the combinations.

**McNeill** responded that the inclusion of “accepted” rather than “used” made it clear that it was not a problem. It was only the new combination that was accepted, not the basionym.

**Schori** stated that a name had to be accepted by the author in order to be validly published. She was therefore not sure that the proposal was accomplishing anything new.

**Floor Prop. 11** was **accepted as amended**.

**Floor Prop. 12, Rambold, Bensch, Kirk, Yao, Robert, Sanz and Triebel** [To recommend in a new Rec. 46E that an identifier issued by a recognized repository may be used in place of an author citation; the **proposal** was supported by **five seconders**.]

**Bensch** explained that this proposal would add a new Recommendation to Art. 46, with an Example. It came about because the length of author name citations had significantly increased in the last decades, and there was more ambiguity in the citations due to variations in their abbreviations and incomplete or incorrect citations. The proposed new Recommendation would replace the author citation in publications subsequent to the protologue by the identifier that was issued for that name by a recognized repository.

The Example showed that it would increase the readability within the text. Authors preparing manuscripts would no longer need to check for the correctness of the author citation. As an additional advantage, the identifier could be linked with the website of one of the repositories so that you could access all the data available for an author. Bensch said this was comparable to what had already been done with references that were linked in online publications and would be a similar way to link the names with the websites of the repositories. Some of the Pensoft journals, like *MycoKeys*, already linked the name with a database or with an online database. The correctness of the identifier could easily be checked by the editors in using appropriate IT tools, by cross-checking, parsing and so on, and it would be less time-consuming than checking the correctness of the author citation.

**Middleton** asked why this perceived problem could not be overcome by Rec. 46C.2, which said that after a name published jointly by more than two authors the citation should be restricted to the first author followed by “et al.”

**Bensch** pointed out that, in the Example, if you shortened it to the first author plus “et al.”, then you still have a long string instead of six digits if an identifier was cited. There would also be the advantage of the linking.

**Wilson** expressed sympathy with the proposal, but agreed that “et al.” would be more user friendly, and if it just had a URL not everyone was going to be able to get through on that URL. As it was only a Recommendation it might be okay, but she was wary of it.

**Groom** agreed there were great advantages in being linked but the linking was not put into the Recommendation and he wished to suggest an amendment. The fact that it had to be linked and it had to be an electronic publication should be in the Recommendation. If it was not an electronic publication, then the number would mean nothing to anybody.

[The **amendment** was **accepted** as **friendly.**]

**Hawksworth** added that, as only fungi have repositories, “of fungal names” should be inserted after “repository”. He said that as an editor he generally deleted all author citations and this seemed a reasonable compromise. These things were easily accessible through the online systems from pretty well anywhere in the world.

[The **amendment** was **accepted** as **friendly.**]

**Redhead** spoke to those debating whether to use the first author plus “et al.”, saying there was a part in brackets where you would have to say “M. F. Landell et al.”, followed by “A. M. Yurkov et al.” Both the basionym authors and the publishing authors would have to be reduced if this method was used.

**Watson** said that because this would now be only applicable to fungal names and would be in the new Chapter, it could be left for the next IMC to sort out. He called the question.

[The Section voted **to vote**.]

**Floor Prop. 12** was **rejected**.

**Floor Prop. 13, McNeill** [To rule as illegitimate in Art. 54.1 a name of an algae or fungus originally assigned to another Code under which that name is unavailable for use; the **proposal** was supported by **five seconders**.]

**McNeill** said this proposal would only be added to the portion of the *Code* where there was an interaction with other *Codes*: it concerned Art. 54. There were situations where a taxon was described and published under another *Code*. In the Example, the name of an organism that was now considered to be an alga was published under the zoological *Code*. Under the zoological *Code*, there was an earlier homonym. A replacement name was then published, and that name was the correct name under the zoological *Code*. When transferred to the jurisdiction of our *Code*, the replaced synonym was available. The intent of this proposal was to plug that gap. There was one specific case, but there was no reason to suppose there would not be others. It would not be a flood, but there would undoubtedly be the odd occasion where this also applied.

The arrangement of the proposal was that a name that was illegitimate under the zoological *Code* or the prokaryote *Code*, if then found to apply to an organism falling under our *Code*, was also illegitimate.

**Watson** asked the proposer if this would introduce a second Article that contravened the first line of Principle I, that the *Codes* were independent?

**McNeill** said that there were a number of small items in the *Code* that were not independent when there was a name for an organism that was published under one *Code* and was then treated under another. This was no different from what was already in the *Code* in that respect. It was an exception, so the Preamble and the Principles would still apply.

**Floor Prop. 13** was **accepted**.

**Floor Prop. 14, Schori and Wiersema** [To specify in Art. 60.9 under what circumstances a hyphen may be inserted into a compound epithet; the **proposal** was supported by **five seconders**.]

**Schori** explained that this proposal, which would amend Art. 60.9, was designed to deal with instances where there were two vowels side by side. Art. 60 Ex. 25 suggested that the hyphen was to be maintained in the epithets *austro-occidentale* and *pseudo-oblongum*. In those cases, they were published with hyphens, which should be maintained. There was nothing in this Article that said if an epithet was not published with a hyphen then it was permissible to insert one; often having a hyphen increased readability. Currently, for indexing purposes, having *austro-occidentale* or *pseudo-oblongum* with and without a hyphen meant there were two different versions.

The original proposal was modified to exclude cases where there might be a connecting vowel, like an -*i*- in *opuntiifolia* or *tiliifolia*. As far as was known, this only affected compounds where the first part of the compound ended with an -*o* and the second part of the compound began with an *o*-. Schori had asked Lindon and Hartley, editors of IPNI, to check for other instances in IPNI.

**Lindon** stated that she had only found *pseudo*-, *austro*- and *neo*-. There was nothing with consonants or other vowels. There were about 60 records that had hyphens and about 20 that did not.

**Applequist** pointed out that many of these compounds involved the term *pseudo*-. The deletion of “or” and the addition of the period after “independently” said that the hyphen was only permitted when the epithet was formed of words that usually stood independently, and she questioned whether *pseudo*- usually did. She thought this might threaten some cases where the hyphen was currently used.

**Gereau** said there was no precedent for mandating the insertion of a punctuation mark in an epithet that was published without it, and there was no reason to start now. The presence of a hyphen made electronic searches more difficult because the hyphen had to be typed in. He thought the community should be striving to eliminate extraneous pieces of punctuation. There had been specious discussion at the Melbourne Nomenclature Section [2011] about eliminating or not eliminating the diaeresis. He asked to get punctuation out of the epithets, not start inserting it where it never was.

**Garland** thought the proposal did not make sense as written, because when the vowels before and after the hyphen were the same, if the hyphen was missing it implied it was to be inserted. If there was no hyphen, how would you determine which were the vowels before and after the hyphen.

**Floor Prop. 14** was **rejected**.

**Floor Prop. 15, de Lange** [To add a Note under Art. H.2.1 to show how hybrid formulae are expressed; the **proposal** was supported by **five seconders**.]

**De Lange** explained that this proposal, to add a new Note under Art. H.2.1, sought to add clarity regarding how hybrid formulae are expressed, because there was confusion in the literature and in various websites and databases. [The aim of the proposal was to discourage expressions such as “Kunzea linearis × robusta”, in which the generic name or its abbreviation is omitted from names following the multiplication sign.]

**Floor Prop. 15** was **accepted**.

**Floor Prop. 16, Schori, Redhead, Malécot, Paton, Wilson, Lindon, Groom, Kusber and Hartley** [To permit in Art. 40.5 the type of a name of a microscopic alga or any fungus to be a specimen that consists of more than one gathering, with certain conditions; the **proposal** was supported by **five seconders**.]

**Schori** noted that this started off as an attempt to deal with the situation of herbarium specimens made from cultivated material and wild gatherings, originally proposed as a modification to Art. 8.2. However, now that a definition of a gathering had been accepted as part of the *Code*, the proposers realized that this would affect Art. 40.5. For fungi and microscopic algae, multiple cultured material on different substrates (slides) could be put onto one sheet and treated as one gathering. There were various attempts to get around the issues of deciding whether this would be a single or multiple gathering by saying that the date on the sheet would be what made it a gathering. Even if specimens were prepared or collected at different times, as long as they were put on a sheet on the same date, it was not considered to be a different gathering.

This proposal sought to prevent having to publish subsequent lectotypifications of fungal names based on specimens that had been prepared in this way and recognized that, given the biology of these organisms, material from different cultures or on different substrates was often required to get enough data to confirm that it was a new species. The Note would make it clear that multiple collections of cultivated material could not be put together and considered as one gathering.

**Redhead** expanded the discussion by saying that this was common practice in mycology in the past. One could isolate a fungus from soil or air and obtain a colony. This would be grown out on various substrates, or put under UV light and various results obtained. They all came from one source but might be dried over months or days and put on a sheet. The proposers did not want to restrict this to organisms collected on the same day in the laboratory, because it was only after growing them under these different regimes that mycologists finally realized that they had a new species or genus.

There were other cases where a mycologist would take two different isolates from two different sources and cross them via a sexual event and then, suddenly, they would have the teleomorphs produced and get excited and then declare the whole thing a type. That was why there was a second part to the proposal regarding an isolate derived from a single sexual cross. The proposal would restrict this so that the type did not, for example, come from two different countries. The wording was to cover these two kinds of situations and prevent a type from being different isolates from different time periods. There was a similar situation for microscopic algae. If you had a small alga floating somewhere and isolated it, you may have a single cell that you could then use to generate a culture and fix it in some way to make a specimen, but it would have to be generated in the laboratory.

**Nakada** said as a microphycologist he hesitated to support this, because the phycological custom was different. Because he could not understand the proposal fully, he felt it should be more deeply considered. He proposed to refer it to the Special-purpose Committee on Typification.

[The **proposal** was supported by **five seconders**.]

**Applequist** pointed out that fungi were not now the problem of this Section since mycologists could do what they wanted at an IMC. It seemed to her that there was not the expertise in the Section to answer the microalgal question at this time, and this was the sort of thing that the new Special-purpose Committee had been set up to examine.

**Marhold** said he had advised a mycologist that if it was already established mycological practice, the proposal should be separated out and approved at this Section; the algae situation could be left for the Special-purpose Committee.

**Schori** pointed out that the proposers consulted with someone who worked on microalgae and whose name was on the proposal, so they had considered the point of view of someone from the phycology world.

**Sennikov** called the question.

[The Section voted **to vote**.]

**Floor Prop. 16** was sent to the **Special-purpose Committee on Typification**.

**Knapp** announced that while the deadline to submit proposals to amend the *Code* from the floor had passed, the same deadline did not apply to proposals to establish Special-purpose Committees and there was a proposal from the floor to establish a Special-purpose Committee.

**Freire-Fierro** explained that she and some colleagues had submitted an article in *Taxon* proposing to establish a Special-purpose Committee on virtual participation in Nomenclature Sections of future Congresses. That would be a good opportunity for many botanists to participate in the meetings.

[The **proposal** was supported by **five seconders**.]

**Fortunato**, as an author of the proposal, felt it was important to say that in the future it was likely that virtual and internet contact and voting would be easier than it was now.

The **proposal** to establish a **Special-purpose Committee on Virtual Participation in the Nomenclature Section** was **accepted**.

#### Appendices II–VIII

**Wiersema** introduced a presentation to bring up issues pertaining to the Appendices and get some feedback from the Section. In 2015, around the time that the [*Melbourne Code*] Appendices came out, a paper appeared in *Taxon*, partly an analysis of the history of proposals, but also showing how the text of the *Code* had grown in relation to the size of the Appendices, especially in recent years [Wiersema & al. in Taxon 64: 1021–1027. 2015 https://doi.org/10.12705/645.11].

The Appendices to the *Shenzhen Code* would have an additional 375 entries added, which was why at Melbourne [2011] the Editorial Committee was empowered to publish the text and the Appendices separately, and to consider whether the Appendices might be published online. The Appendices were published in hardcopy [2015] but there was now an online database thanks to the Smithsonian National Museum of Natural History [https://naturalhistory2.si.edu/botany/codes-proposals/]. Dan Nicolson, a long-time nomenclature editor of *Taxon* and President of the Vienna Nomenclature Section [2005], had originally created two databases: one dealing with confusable names, the other with proposals to conserve or reject names. The content of those two databases fed into the present database, which also brought in the data from the Appendices that resulted from these proposals.

Two types of reports could be generated from searching the present database. One type of report would tell you the history of a proposal, when it was proposed, how the specialist committees and the General Committee recommended on it and, if it ended up getting into the *Code*, then the text of the relevant Appendix entry for a particular name or work would be displayed. It was also possible to select the names in the Appendices that fit a set of criteria, for example, those dealing with species conservation. If you wanted to retrieve the entire Appendices, it was a simple matter of leaving everything blank and selecting the Appendices report.

Some issues regarding the Appendices had been published in a recent paper in *Taxon* [Wiersema & al. in Taxon 66: 772–775. 2017 https://doi.org/10.12705/663.38] that raised the question of whether the Appendices should continue to be published in hardcopy. If so, should the hardcopy of the Appendices be published at the same six-year interval as the main text? If the Appendices were not published in hardcopy, would there be a need to archive a snapshot at some point in time, for example every six years, as a Version of Record? With the online version it was now possible to make corrections, and these were constantly being fed in. The corrections could be made and flagged, using the cross-out feature for things that were deleted, or the underscore for things that were inserted. Another question was, therefore, whether such corrections should continue being made on a regular basis or whether it was better to wait and include them in the official “Version of Record”. This would be different from what had been done prior to having the online resource.

If there was a hardcopy version and an electronic version of the Appendices that was static, or if there was a dynamic version in which corrections could be made, Wiersema asked which one of these would then become the Version of Record. He wished to discuss these questions and gauge what the Section felt about some of the issues.

**Turland** said that at the Melbourne Congress it was agreed by a vote of the Section that the Appendices did not have to be published together with the *Code*; they could be published separately and in electronic form only. The Appendices to the *Melbourne Code* were, indeed, published separately in both print form and electronic form. He invited the Section to give feedback to the Vice-rapporteur and himself, and to the Editorial Committee, about whether people were happy with what had been done up to this point and to bear in mind the questions that the Vice-rapporteur had just put before the Section.

**Redhead** congratulated the Rapporteurs for what they had accomplished in adding all the information to the database. Secondly, with regard to the increase in the number of names that were listed, he anticipated for mycology that the lists would shortly have thousands, not hundreds, of names added. He liked the idea of the snapshot to preserve it at one time, and asked delegates to indicate if they had physically seen a hardcopy of the Appendices.

[*There was a show of hands.*]

**Wiersema** added that there were additional issues related to fungal Appendices that he wanted May to speak about.

**Kirk** asked whether there was a URL for a clickable link that he could add to generic names in *Index Fungorum* that were cited as “nom. cons.” to take users to the information on conservation of that name on the Appendices website.

**Knapp** suggested that Kirk was getting into specific matters, when they were trying to get the feeling of the Section.

**Kirk** argued that his question was about whether the website was fit for purpose in the 21^st^ century.

**Wiersema** suggested it would be best to link to the proposal report that related to the name.

**Kirk** said the proposal reports were long text discussions and he just wanted the little bit that said “nom. cons.”

**Wiersema** explained that the first of the two reports he had demonstrated was the history of a proposal, which included the header from the paper as it went into the Appendices, which was what Kirk wanted.

**Wilson** thought that hardcopy would soon become inappropriate. She supported the move to electronic but thought a snapshot should be archived as a record, perhaps when each new *Code* came out. She said that this was one problem with electronic floras or lists, that there was no record of what had been done at a certain time. She thought it may also be useful to archive the database once a year, but at least every six years. However, changing the *Code* after fungal conferences [i.e. IMCs] would complicate it.

**May** said the Appendices would not be changed after the IMC, only after the IBC. Ordinary proposals to conserve and reject names were treated exactly the same way for fungi as for anything else. They would appear in the General Committee reports in the same manner as a proposal to conserve the name of any other group.

**Wiersema** added that there was the possibility of another Appendix, dealing with protected names.

**May** elaborated that the specific issues to do with the fungal names were under Art. 14.13, added since the Melbourne Congress. These were names that were protected, and their status was different to conserved names. There were 51 of these names, and the question would be where to put them. On the one hand it would be useful to insert them in the lists of genera, species and so on, with an indication that their status was different. On the other hand, because the status was different it could be useful to put them in separate Appendices.

A second issue was that there was the potential under Art. 14.13 and Art. 56.3 to generate large lists of potentially thousands of names. That would influence the decision to print the Appendices. Kirk had indicated that in *Index Fungorum* he flagged conserved names. If large lists were being generated to be submitted under Art. 14.13, they would come out of the nomenclatural indices in the first place, because that was how the list would be generated. If thousands of names were being dealt with, it would make sense to have tight integration between the nomenclatural indices and the lists of the names that were protected under the *Code*.

**Saarela** thought in addition to the online database it would be most useful to have a nicely formatted Appendix published as a paper in *Taxon*, for example, and the same for the *Code* to make that content more accessible.

**Paton** thought it was great to have a database. He thought that what was missing was a reference in the printed *Code* or *Code* website, which referred to Appendices I to VIII. Since he was not particularly *au fait* with the Appendices, it was difficult to find a list of what they were.

**Wiersema** thought there was a link from the *Code* itself to the Appendices.

**Paton** said he could not find it.

**Wiersema** suggested it was on the nomenclature page [of the IAPT website].

**Knapp** agreed that the list of the Appendices had not gone into the electronic copy of the *Code* and thought it was a very nice suggestion.

**Nakada** expressed his preference for effective publication in conformity with the *Code* (i.e. PDF) every six years.

**Schori** said when hosting Appendices in a database, there should be some redundancy so that if the Smithsonian, for example, was subject to US government shutdowns and their web pages were not available, this information would still be available elsewhere. If there were going to be lots and lots and lots of fungal names that needed to be entered, it would be appropriate to have more than one person who was responsible.

**Smith** recalled that in Melbourne there was a comment that in many parts of the developing world it was easier and more affordable to access these documents online. However, that was not yet applicable throughout the developing world so, for the moment, there was a place for both hardcopy and internet content.

**Wiersema** asked if anyone had brought their copy of the Appendices to the Section.

**Knapp** commented that no one seemed to have brought one, likely because of its size. It was already 2 cm thick, before all the names went in from this [*Shenzhen*] *Code*.

**Herendeen** noted that it was about twice as thick as the 2012 *Melbourne Code*.

**Govaerts** suggested rather than republish or reprint the entire Appendix, just the changes to the previous Appendix could be printed.

**Knapp** suggested everyone could go talk about the Appendices over tea. She also reminded the Section there was a new Special-purpose Committee on Virtual Participation in the Nomenclature Section and she had made two sign-up sheets for interested people.

[*The Section broke for afternoon tea.*]

**Knapp** welcomed everyone back to the final session, noting that everyone had done well in getting through all the sessions extremely efficiently, quickly, and in incredibly good humour. The last item of business was the reports of the various committees, beginning with the report of the Nominating Committee.

#### Report of the Nominating Committee

**Funk** explained that the Permanent Nomenclature Committees were elected at the end of this Section, every six years, and were run by a Chair and a Secretary. Traditionally, at the beginning of the Section meeting, the Secretaries presented their list of the people that they would like to have on their committee for the next six years, barring unforeseen circumstances. The report was actually very short, just the list of names that followed. Most of the committees were well balanced geographically, and the Nominating Committee made a few additions to make sure they had a geographical and gender balance.

They had found the gender balance to be somewhat lacking but with the cooperation of the Secretaries of the committees, they had added several people to some of the committees to improve gender balance, with a goal of increasing the number of female members to 20%. They were still taking volunteers for the two new committees that had been set up, and for those committees 50% or more of the volunteers were female. Funk finished by thanking the Nominating Committee for their work.

**Knapp** interjected that the Nominating Committee also proposes the Rapporteur-général for the next IBC as part of their slate.

**Funk** agreed, saying they now had the Rapporteur-général, and the Secretary of the Fungal Nomenclature Bureau. After this Congress the latter would be taken care of at the IMC, but they were jump-starting it here.

The General Committee had two kinds of members: regular members, and ex-officio members who were the Secretaries of the various specialist committees. She asked the Recorders’ Assistant to scroll through the list so that members of the Section could have a look at them. Funk said that some of the committees had done an excellent job in achieving balance, pointing out that the Committee on Fossils, whether its name referred to the age of the participants or to the taxa they were studying, was very well balanced. She continued that the Committees for Fungi and Bryophytes were doing well also.

Most of the committees comprised around 20 people. Some of them were a little smaller, some a little larger, for various reasons. The optimal number for getting a 60% vote was about 20. Several people had asked how they could become a member of one of these committees, and Funk had suggested that if they had not been on a committee before, they should volunteer for one of the Special-purpose Committees; if they did the work and contributed to the reporting on such a committee they would be picked up for something else. She urged delegates not to worry: like the vampires of nomenclature on Applequist’s committee, anyone with a heartbeat could do it.

**Knapp** pointed out that the Editorial Committee should be composed of people who had been present at the Nomenclature Section, and it should also represent the taxonomic spread of the *Code*.

**Turland** added that it had to have at least one representative from each of the five specialist committees, which it did.

**Funk** spoke about the newly passed Committee on Institutional Votes, where the Rapporteur-général served as the Chair. The Registration Committee had some people who had volunteered and some prescribed members. All five specialist committees had a representative, as did IPNI and TROPICOS as potential registration providers.

**Barkworth** added that if Funk was listing places that were along the line to be registration centres, Berlin had PhycoBank coming, and there was one for fossil names. These had not been included in the report because it came in late.

**Knapp** suggested voting on the report of the Nominating Committee, which would then establish the committees with the membership as shown to the Section on the screen.

The **report of the Nominating Committee** was **received and approved unanimously**.

#### Reports of the Permanent Nomenclature Committees (including the specialist committees)

##### Nomenclature Committee for Algae

**Nakada** delivered the message from the Secretary of the Committee [Willem Prud’homme van Reine], saying the NCA had always been a small Committee, usually with 15 members. However, they had lost two devoted members, the former Committee Chair, Paul Silva, Berkeley, USA, who had died on 12 June 2014, as well as a former Committee Secretary and a specialist in freshwater algae, Pierre Compère, who had died on 28 April 2016.

The Committee Secretary was, for some years, unable to work on Committee activities, because his archives had been temporarily lost after one of the successive removals to other rooms and even buildings for the Naturalis Biodiversity Center. The new Chair, Bill Woelkerling, managed, with Prud’homme van Reine, to prepare many notices on recommendations about proposals to conserve or reject names, as well as three notices on binding decisions on the application of the *Code*. These actions, and the preparation of 17 internal discussion papers, communications and 12 proposals to amend the *Code*, produced a large amount of correspondence. He warmly thanked the members of the NCA for their help and for their timely answering of the many e-mails he had sent to them, especially the Chairman.

On 1 July 2017, Bob Andersen took over as Secretary of the NCA and the new membership was nominated.

##### Nomenclature Committee for Bryophytes

**Price** informed the Section that over the last six years some people had resigned or retired and were no longer on the Committee. These included David Long, from Edinburgh; Bill Buck, from New York; and David Glenny, from New Zealand. They also lost a long-serving member, Benito Tan, who had died at the end of 2016.

The Committee Chair was David Long, although he had resigned from the role of Chair during 2016, so there was not much activity during that period. They did, however, deal with a number of proposals and requests and forwarded their recommendations to the General Committee in mid-2016 and early 2017. The Secretary [Niels Klazenga] was unable to be at the Section, and the previous Chair had stepped down, so they were in hiatus.

##### Nomenclature Committee on Fossils

**Herendeen** reminded the Section that they were also known as the Fossil Committee – to the annoyance of some members. They had dealt with 80 proposals in the last six years, more than usual. The majority were proposals for conservation, with a couple of requests for decisions on confusable names. All were resolved and sent on to the General Committee. They started in 2011 with 15 members; after about one year, one person stepped down because it “wasn’t their cup of tea”, and someone else resigned after about four years. They finished up with 13 members. In addition to trying to have gender balance and a good geographical distribution, Herendeen also tried to get career-stage diversity in the membership. He also had to balance expertise in Palaeozoic, Mesozoic and Cenozoic time periods, as well as macrofossils and microfossils.

##### Nomenclature Committee for Fungi

**May** informed the Section that he took over as Secretary of the NCF from Lorelei Norvell late in 2014. Lorelei remained an active member of the Committee, and he paid tribute to her long and distinguished service to the NCF. Around 120 matters had been sent to the NCF since the last Congress [Melbourne, 2011], of which 63 had been finalized and reported on. The NCF also dealt with the establishment of working groups under Art. 14.13, in consultation with the International Commission on the Taxonomy of Fungi. Six lists from those working groups, comprising a total of 51 names, had been approved. In addition, the NCF had confirmed three repositories for registration of names of fungi under Art. 42.3.

##### Nomenclature Committee for Vascular Plants

**Applequist** reported that between the Melbourne Congress [July 2011] and February 2017, the Committee for Vascular Plants dealt with 354 proposals; she quipped that the other committees did not know what a workload was. The largest categories were 217 proposals dealing with conservation or rejection of species names, and 63 dealing with conservation or rejection of generic names. In addition, there were 20 requests for binding decisions on the adequacy of a description, and 23 requests for treatment of similar names as homonyms.

Throughout this last six-year period they had always had 18 members. When one was lost, they would then try to replace them, and there had been considerable turnover during this period. Two members who had passed away had already been mentioned: Dick Brummitt, after his retirement from the Committee, and Gill Perry. In addition, there was Koos Roux. The Committee had agreed to increase membership to 20 and try that out. That left two slots for a little more geographical diversity, and hopefully it would make 60% an easier voting majority to achieve.

##### Editorial Committee

**Turland** reported that the Editorial Committee comprised 14 members, chaired by John McNeill with Turland as the Secretary. The Committee worked extensively online by e-mail, but also met for a week in London in December 2011, at the Natural History Museum. The result of all this work was the *Melbourne Code*. It existed in hard copy, published in December 2012. The online version followed shortly afterwards, on the IAPT website. The Appendices of the *Code* were published as hardcopy in 2015.

He recognized the service of three former members of the Committee. These included Willem Prud’homme van Reine, the algologist on the Committee, and Bill Buck, the bryologist. In particular, he wished to recognize Vincent Demoulin, who was not able to attend this Nomenclature Section. He stated that Demoulin was missed, but hastened to add that he was fine, and still alive [*Laughter*], just not present at the meeting. Turland believed that Demoulin held the record, possibly an equal record, of having served on more Editorial Committees than any other person. He had served on the Editorial Committee for the last seven *Codes*, since the Leningrad *Code* back in the 1970s [1978]. He remained on the General Committee and was still available to help the new Editorial Committee with any mycological or lichenological questions they might have.

##### General Committee

**Wilson** noted that the General Committee started out with 24 members immediately after the [Melbourne] Congress in 2011. Ghillean Prance immediately resigned, leaving 23. Tony Orchard retired in 2014/2015; he had been a member since 1981, like Werner Greuter, who was still on the Committee. Wilson hastened to add that Orchard was also still alive and kicking. In 2016, they elected three new members to fill gaps and broaden the range of people serving, so the Committee now had 25 members.

Wilson showed the proportions of proposals, requests for binding decisions, suppression of works and other matters that the Committees had dealt with. This would give an idea of the total number dealt with by all the Committees. The Committee for Vascular Plants, as Applequist had said, had an incredible workload compared to the Committee for Bryophytes with their 13 proposals and one confusability request.

Wilson said the Secretaries had done a terrific job with their Committees, and that it was good to have such dedicated people. The proportions were the important thing: the actual figures varied somewhat, because they included a few old requests that had long since been through the different specialist committees.

In 2011, after the [Melbourne] Congress, the General Committee was responsible for approving and setting up five Special Committees. These went into a report immediately after the Congress [Wilson in Taxon 61: 878–879. 2012 https://doi.org/10.1002/tax.614015]. They had published revised guidelines for all the nomenclature Committees’ procedures in a report in *Taxon*. The only real change was that they decided it would be more appropriate to have a 60% qualified majority, or supermajority, for all proposals and all requests. In the past, the binding decision requests only required a 50% figure, but they felt that it was more appropriate to make them all 60%. They had published nine reports in *Taxon*, the most recent of which was Report 20, which had appeared online during the current week. Reports 12 to 20 had come out since the last IBC.

Wilson expressed her appreciation for the work of the members in the General Committee, as well as those in all the other specialist committees. She appreciated greatly the support of people like John McNeill and Franz Stadler, in terms of editing the proposals. They did a great job behind the scenes in making sure the proposals that went into *Taxon* and their reports were presented very well. [*Applause*]

**Knapp** asked for the Section to vote to confirm that it had received all seven Committee reports.

**Turland** pointed out that the vote had nothing to do with ultimate acceptance or otherwise of proposals to conserve or reject. It was just to record that the Section had received and heard the reports in the Section.

The **reports of the Permanent Nomenclature Committees** were **received unanimously**.

**Knapp** moved on to the next item of business, which was to accept the recommendations of the General Committee reports. These were the things that went into the Appendices. Based on earlier agreement, there was no percentage with which to *accept* the General Committee reports. She explained that the Section was voting to *reject* the General Committee reports. If there was a 60% majority to reject the General Committee reports, they would be rejected in their entirety. If there was not a 60% majority to reject the General Committee reports, they would be accepted.

[*Unanimous show of hands against rejecting the General Committee reports; laughter.*]

The **recommendations** of the **General Committee reports** were **accepted**.

[*The recommendations were published in reports 13–20 of the General Committee, listed below. Report 12, also mentioned by Wilson, concerned only Special Committees and contained no recommendations*.]

13: Wilson in Taxon 65: 380–381. 2016 https://doi.org/10.12705/652.17

14: Wilson in Taxon 65: 878–879. 2016 https://doi.org/10.12705/654.15

15: Wilson in Taxon 65: 1150–1151. 2016 https://doi.org/10.12705/655.14

16: Wilson in Taxon 66: 189–190. 2017 https://doi.org/10.12705/661.15

17: Wilson in Taxon 66: 478–480. 2017 https://doi.org/10.12705/662.13

18: Wilson in Taxon 66: 742–744. 2017 https://doi.org/10.12705/663.15

19: Wilson in Taxon 66: 980. 2017 https://doi.org/10.12705/664.14

20: Wilson in Taxon 66: 981. 2017 https://doi.org/10.12705/664.15

**Knapp** reiterated that this was a consequence of what had been voted on in Div. III Prop. B. The Section had now accepted the General Committee reports, but this would have to be done in subsequent Nomenclature Sections, because of what had been accepted in Div. III earlier.

#### Resolution

**Turland** announced there was a final but very important item of business to attend to, otherwise, all the work the Section had done over the last week would be ineffective. He moved that the Nomenclature Section instruct the President and the Rapporteurs to present a resolution to the Resolutions Committee of the XIX IBC, to the effect that the Congress approved the decisions and appointments of the Nomenclature Section. The text had not yet been finalized, but he read a draft to give an idea of what a resolution sounded like:

“*The XIX International Botanical Congress resolves that the decisions of its Nomenclature Section with respect to the International Code of Nomenclature for algae, fungi, and plants, as well as the appointment of the Rapporteur-général and the Fungal Secretary, and officers and members of the Permanent Nomenclature Committees made by that Section during its meeting 17–21 July 2017 be accepted, noting with interest a framework for the mandatory registration of algal and plant names, provisions for improved clarity in the governance of the Code, and extension of governance of nomenclature that solely relates to fungi to the International Mycological Congress.*”

The **motion** was **accepted unanimously**.

#### Conclusion of business

**Knapp** noted that this concluded the formal business of the meeting. She wished to express her thanks to Li Zhang and the local organizing team for providing a seamless set of tea breaks, for transporting delegates to and from the venue, and organizing the buses from the hotel to the main Congress venues. She told them that delegates owed them a huge vote of thanks. [*Applause*]

Knapp went on to thank the Botanical Society of China, who had supported this Nomenclature Section, and the International Association for Plant Taxonomy, who likewise supported the meeting. She also wished to thank the Shenzhen City Government, who generously helped with the planning and preparation for this Section. Last of all, she wished to thank all the attendees for conducting business in an open, friendly and collegial manner, which she thought spoke to the fact that the Section was celebrating the 150^th^ anniversary of the very first Laws of Botanical Nomenclature, written by de Candolle, in which he said that we needed to establish a framework that would facilitate the science that it aimed to support. She thought this had been achieved over the last five days. A framework had been established, and the Section had enabled it to facilitate the science that the community did every day. [*Applause*]

**McNeill**, on behalf of the members of the Section, thanked the Bureau for the excellent way they had conducted the business of the Section so expeditiously, but most particularly he wished to thank the President for her quite outstanding chairmanship, and for ensuring that business had been completed not only on time, but with a great deal of humour and enjoyment. [*Applause*]

**Knapp** thanked McNeill and said that when he first asked her to do this before Melbourne, she said, “John, but I don’t know anything about nomenclature”. McNeill had said, “You don’t need to know anything. All you need to do is be able to keep order”. So with that, our business is concluded!

The end.

## Appendix A

Proposals made from the floor of the Nomenclature Section

The following 16 proposals were submitted to the Bureau of Nomenclature from the floor of the Nomenclature Section before the deadline, set by the Bureau, of the end of the afternoon session on Thursday, 20^th^ July. They were discussed and voted on during the morning and afternoon sessions on Friday, 21^st^ July. The text of the proposals was in some cases amended (friendly or unfriendly amendments), as described in the main proceedings. When the proposers provided explanatory text, this has been included here, following the heading “*Explanation*”.

**Floor Prop. 1**, proposed by A. Sennikov (rejected)

[Floor Prop. 1–3 were reformulated from Art. 8, Prop. N, which was also proposed by Sennikov and withdrawn by him.]

Replace the footnote to Art. 8.1 with a new Article and add three Examples:

“*8.3bis.* For the purpose of typification an illustration comprises one or more drawings, paintings, photographs or other graphical works that are referable to a single taxon and depict one or more morphological features of an organism.”

“*Ex. 5bis.* The illustration of *Gladiolus fistulosus* Jacq. (Pl. Hort. Schoenbr. 1: t. 16. 1797) shows two plants, of which one (incomplete plant) is more typical of the taxon and the other (complete plant with three separate fragments) may be a hybrid. These two figures were apparently drawn from different plants, which may be taxonomically different; Goldblatt & al. (in Bothalia 43: 134. 2013) designated a single element, the left-hand plant on the illustration, as the lectotype of this name.”

“*Ex. 5ter.* The lectotype of *Chaetanthera pinnatifida* Humb. & Bonpl. (Pl. Aequinoct. 2(17): 170, t. 136. 1817), designated by Vuilleumier (in Contr. Gray Herb. 199: 140. 1969), is the illustration published in the protologue, which consists of drawings of a complete plant with an analysis of eight details that were presumably drawn from the same plant.”

“*Ex. 5quater. Lawreymyces bogotensis* Lücking & Moncada (in Fungal Diversity 84: 133, t. 7A. 2017) and seven other taxa were typified solely on diagrams published in the protologue that represent diagnostic ITS sequences. These are not depicting characters of morphology, and cannot be used for typification.”

**Floor Prop. 2**, proposed by A. Sennikov (rejected)

Add a sentence to the new Article of Floor Prop. 1 (new text in bold):

“*8.3bis.* For the purpose of typification an illustration comprises one or more drawings, paintings, photographs or other graphical works that are referable to a single taxon and depict one or more morphological features of an organism. **If an illustration comprises more than one element, its elements should be organized in a single plate or its equivalent.**”

**Floor Prop. 3**, proposed by A. Sennikov (withdrawn)

Add a sentence to the new Article of Floor Prop. 1 (new text in bold):

“*8.3bis.* For the purpose of typification an illustration comprises one or more drawings, paintings, photographs or other graphical works that are referable to a single taxon and depict one or more morphological features of an organism. **Unpublished sketches[, which are not ready and presumably were not intended for publication,] cannot be designated as types.**”

**Floor Prop. 4**, proposed by J. McNeill (accepted)

Amend Art. 9 Note 1 to read (new text in bold) [text in square brackets already accepted from Art. 9, Prop. C]:

“*Note 1.* Any designation made by the original author, if definitely expressed at the time of the original publication of the name of the taxon, is final (but see Art. 9.11 and 9.15). If the author used only one element [(i.e. specimen or illustration)] **when preparing the account of the new taxon**, it must be accepted as the holotype. If a name of a new taxon is validly published solely by reference to a previously published description or diagnosis, the same considerations apply to material used by the author of that description or diagnosis (see Art. 7.7; but see Art. 7.8).”

*Explanation* – Some authors have misinterpreted the verb “used” (deployed as a means of accomplishing a purpose) in this Note to mean “mentioned in the protologue”. The added words will make clearer that it is [i.e. means] use in preparing the content of the protologue. As it is a small modification to a Note, it is probably just editorial, but as the confusion has been troubling for diatomists and others, it seems worth having it approved by the Section.

**Floor Prop. 5**, proposed by J. McNeill (accepted)

Amend Art. 9 Note 7 to read (new text in bold):

“*Note 7*. An epitype supports only the type to which it is linked by the typifying author. If the supported type is **lost, destroyed, or** superseded, the epitype has no standing with respect to the replacement type.”

*Explanation* – There was extensive discussion in Vienna [2005] on a proposal that suggested that “supported type” in the second sentence of this Note be replaced by “lectotype or neotype”. The proposal was eventually, and correctly, defeated because an epitype may also support a holotype. But as this is the case, the word “superseded” is misleading because the destruction or other loss of a holotype is not generally thought of as its supersession. The suggested additional words would clarify this and avoid the confusion engendered by the proposal in Vienna.

**Floor Prop. 6**, proposed by K. Wilson, J. McNeill, D. Mabberley, F. Barrie & V. Funk (accepted)

Add to the end of Art. 14.3: “Application of conserved and rejected names of nothogenera is determined by a statement of parentage (Art. H.9.1).”

*Explanation* – The intent of the Article is to establish that a name may not be conserved or rejected under Art. 14 unless its application is established explicitly. For all names except those of nothogenera (and of hybrids between subdivisions of genera), this is established by designation of a type. This proposal aims to clarify that Art. 14.3 does NOT preclude conservation of a nothogeneric name, despite such names being defined by parentage and not by a type.

The General Committee [GC] became aware of this lack of clarity in Art. 14.3 when it was considering a case to reject a nothogenus. Rejection of a nothogenus is, of course, allowed by the *Code*, and the GC considered that conservation of a nothogenus should also be possible since it is defined by the names of its parents.

**Floor Prop. 7**, proposed by V. Funk, W. Greuter, J. McNeill, V. Malécot & P. Herendeen (rejected)

[Following from Art. 20, Prop. A, which was rejected, and an unsuccessful amendment proposed to it.]

Delete Art. 20.2 and Ex. 2–6; insert a new clause in Art. 20.4 (shown in bold):

“*20.4.* The following are not to be regarded as generic names:

[…]

***(c)* Some words that have been widely used in the pharmacopoeia or as descriptive morphological terms: *Balsamum, Bulbus, Cortex, Caulis, Flos, Folium, Fructus, Herba, Lignum, Oleum, Radix, Rhizoma, Spina, Semen, э, and Lobata*.**”

*Explanation* – The underlined items are transferred from the former Ex. 4 and 6 (proposed for deletion). All non-underlined items in clause (*c*), not now in the *Code*, are from a list of frequent pharmacopoeia designations ferreted out by Valéry [Malécot].

The rationale as heading clause (*c*) reflects the presumed origin of current Art. 20.2 plus Ex. 6; pharmacopoeia items were often used in the form of apparent Latin polynomials and, if associated with descriptive matter, might be construed to be validly published generic names. To avoid inadvertent revalidation of such “names” through deletion of Art. 20.2, likely candidates not now explicitly mentioned in Ex. 6 were therefore included in the clause (*c*) enumeration.

Acceptance of this proposal makes valid[ly published] the recently proposed name *Solitaria* (McNeill) Sadeghian & Zarre (in Bot. J. Linn. Soc. 178: 667. 14 Jul 2015), based on Arenaria subg. Solitariae (‘*Solitaria*’) McNeill (in Notes Roy. Bot. Gard. Edinburgh 24: 128. 1962) (data provided by K. Gandhi).

**Floor Prop. 8**, proposed by N. Turland & S. Knapp (accepted)

Amend Art. 30.2, as already amended by Art. 30, Prop. D (i.e. as worded here), as follows (deleted text in strikethrough):

“*30.2*. An electronic publication is not effectively published if there is evidence within or associated with the publication that its content and format [layout] is merely preliminary and was, or is to be, replaced by content that the publisher considers final, in which case only the version with that final content is effectively published.”

**Floor Prop. 9**, proposed by J. Miller (rejected)

Insert a new Article following Art. 30.7:

“*30.7bis*. The distribution on or after 1 January 2019 of unpublished printed matter, without an ISBN or ISSN, to botanical institutions and their libraries, does not in itself constitute effective publication.”

**Floor Prop. 10**, proposed by D. Geltman (rejected)

Amend Art. 30.8 as follows (new text in bold):

“*30.8.* Publication on or after 1 January 1953 of an independent non-serial work stated to be a thesis **(including the separately distributed abstract of thesis or similar material)** submitted to a university or other institute of education for the purpose of obtaining a degree does not constitute effective publication unless the work includes an explicit statement (referring to the requirements of the *Code* for effective publication) or other internal evidence that it is regarded as an effective publication by its author or publisher.”

*Explanation* – This amendment will partly solve concern about effective publication of abstracts of dissertations (usually distributed within [a] country only), which is important for Russia and several other countries.

**Floor Prop. 11**, proposed by V. Funk, W. Greuter, J. McNeill, V. Malécot & P. Herendeen (accepted as amended)

Amend Art. 36.2 to read as follows (new text in bold, deleted text in strikethrough) [text in square brackets already accepted from Art. 36, Prop. D]:

“*36.2*. When, on or after 1 January 1953, two or more different names based on the same type are proposed **used** simultaneously for the same taxon by the same author [and accepted as alternatives by that author in the same publication] (so-called alternative names), none of them, **if new**, is validly published. This rule does not apply in those cases where the same combination is simultaneously used at different ranks, either for infraspecific taxa within a species or for subdivisions of a genus within a genus (see Rec. 22A.1, 22A.2, and 26A.1–3), nor to names provided for in Art. 59.1.”

*Explanation* – The proposed rewording widens the coverage of the provision to pairs of alternative names that are not both new. It reflects the meaning that in the past has often been inappropriately given to the provision, to cover a situation not presently covered in the *Code*.

**Floor Prop. 12**, proposed by G. Rambold, K. Bensch, P. Kirk, Y.-J. Yao, V. Robert, V. Sanz & D. Triebel (rejected)

Add a new Recommendation to Art. 46 with an Example:

“*46E.1*. Once an identifier issued by a recognized repository (see Art. 42.2; see also Art. 22.1 and 26.1) is available, it may be used in place of an author citation for a name subsequent to the valid publication of that name.”

“*Ex. 1. Carlosrosaeavrieseae* (M.F. Landell, L.R. Brandão, S.V. Safar, F.C. Gomes, C.R. Félix, A.R. Santos, D.M. Pagani, J.P. Ramos, L. Broetto, T. Mott, M.H. Vainstein, P. Valente & C.A. Rosa) A.M. Yurkov, X.Z. Liu, F.Y. Bai, M. Groenew. & Boekhout may be cited as *Carlosrosaeavrieseae* #814757.”

**Floor Prop. 13**, proposed by J. McNeill (accepted)

Add a new clause (*c*), a footnote, and an Example to Art. 54.1:

“(*c*) A name of a taxon treated as belonging to the algae or fungi but originally assigned to a group not covered by this *Code* and that is unavailable for use under the provisions of the other *Code*^1^, usually because of homonymy, is illegitimate under this *Code*.”

[footnote] “^1^ Such names are termed “objectively invalid” in the *International Code of Zoological Nomenclature* and “illegitimate” in the *International Code of Nomenclature of Prokaryotes* (formerly the *International Code of Nomenclature of Bacteria*).”

“*Ex. 1*. *Cribrosphaerella* Deflandre ex Górka (in Acta Palaeontol. Polon. 2: 239, 260, 280. 5 Sept 1957) was published under the provisions of the *International Code of Zoological Nomenclature* for the Cretaceous coccolith algae, previously known as *Cribrosphaera* Arkhang. (in Mater. Geol. Rossii 25: 411. 1912), an objectively invalid (equivalent to illegitimate) name under that *Code*, being a later homonym of *Cribrosphaera* Popofsky (in Ergebn. Plankton-Exped. 3(L.f.β): 22, 32, 63. 1906) a genus of *Radiolaria*. Although *Cribrosphaera* Arkhang. is not a later homonym under this *Code*, it is illegitimate as it is not available for use according to the provisions of the *Code* under which it was published; consequently *Cribrosphaerella* is the correct name for the genus under both *Codes*.”

*Explanation* – It has been suggested that the new provision [clause (*c*) above] could be incorporated into the current wording of Art. 54.1(b) (see below) and that should be considered by the Editorial Committee, but I [McNeill] believe that the separate presentation above makes the purpose clearer for the members of the Nomenclature Section.

“(*b*) A name originally published for a taxon other than an alga, fungus, or plant, even if validly published under this *Code* (Art. 32–45), is illegitimate if it ***(i)*** becomes a homonym of an algal, fungal, or plant name when the taxon to which it applies is first treated as an alga, fungus, or plant (see also Art. 45.1) or ***(ii)* is unavailable for use under the provisions of the other *Code*^1^, usually because of homonymy**.”

**Floor Prop. 14**, proposed by M. Schori & J. Wiersema (rejected)

Amend Art. 60.9 as follows (new text in bold, deleted text in strikethrough):

“*60.9*. The use of a hyphen in a compound epithet is treated as an error to be corrected by deletion of the hyphen. A hyphen is permitted only when the epithet is formed of words that usually stand independently, or when the letters before and after the hyphen are the same (see also Art. 23.1 and 23.3); **in these instances, a missing hyphen is treated as an error to be corrected by insertion of a hyphen**.”

*Explanation* – Article 60.9 includes an Example (Ex. 26) that states “Hyphen to be inserted”, but the Article itself does not include this provision. The added text would clearly state that a missing hyphen should be added and would remove any ambiguity introduced by “permitted”, which indicates that a hyphen is allowed but not that a missing hyphen should be treated as an error.

**Floor Prop. 15**, proposed by P. de Lange (accepted)

Add a new Note under Art. H.2.1:

“*Note 1*. Because a species name is a binary combination (Art. 23.1) hybrid formulae are expressed in the following manner: *Kunzea linearis* (Kirk) de Lange × *Kunzea robusta* de Lange & Toelken or *Kunzea linearis* (Kirk) de Lange × *K. robusta* de Lange & Toelken, not as Kunzea linearis (Kirk) de Lange × robusta de Lange & Toelken.”

*Explanation* – The current *Code* consistently implies, but does not make implicit [*sic*; presumably “explicit”], that hybrid formulae should be expressed in the following manner: *Kunzea linearis* (Kirk) de Lange × *Kunzea robusta* de Lange & Toelken or *Kunzea linearis* (Kirk) de Lange × *K. robusta* de Lange & Toelken (see, e.g., Art. H.2 Ex. 1 and Art. H.3 Ex. 1). However, there appears to be no clear ruling on how hybrid formulae should be expressed. The lack of stated preference has sometimes resulted in hybrids being expressed as [e.g.] Kunzea linearis (Kirk) de Lange × robusta de Lange & Toelken. This is incorrect because Art. 23.1 states “The name of a species is a binary combination consisting of the [name of a] genus followed by a single specific epithet”.

Because no clear stated preference is evident in the *Code*, this has meant some authors and international websites e.g., DYNTAXA (https://www.slu.se/en/collaborative-centres-and-projects/dyntaxa/), iNaturalist (https://www.inaturalist.org), NatureWatch NZ (http://naturewatch.org.nz/), and USDA Plants Database (https://plants.usda.gov/core/profile?symbol=QUAL3) have expressed hybrid formulae contrary to how it is implied in the *Code*, and they have argued that, because the *Code* makes no rulings, they are entitled to do so. In the absence of a clear ruling, this has necessitated some journals to indicate their preference (following what is implied in the *Code*) in their instructions to authors.

For consistency and clarity, it is recommended that the *Code* spells out exactly how hybrid formulae should be expressed.

**Floor Prop. 16**, proposed by M. Schori, S. Redhead, V. Malécot, A. Paton, K. Wilson, H. Lindon, Q. Groom, W.-H. Kusber & H. Hartley (sent to the Special-purpose Committee on Typification)

Amend 40.5 and add a Note:

“*40.5*. For the purpose of Art. 40, the type of a name of a new species or infraspecific taxon of microscopic algae or microfungi (fossils excepted: see Art. 8.5) may be an effectively published illustration if there are technical difficulties of preservation or if it is impossible to preserve a specimen that would show the features attributed to the taxon by the author of the name. **For microscopic algae and all fungi, it may be a specimen that consists of more than one gathering as long as they represent the same isolate from a single source or an isolate derived from a single sexual cross.**”

“*Note 1*. Type designations for taxa other than fungi and microscopic algae that include citation of more than one gathering (such as a wild gathering and a cultivated gathering or multiple cultivated gatherings) are by definition separate gatherings and do not meet the provisions of Art. 8.1 and 8.2; the proposed names are not validly published.”

## Appendix B

Institutional votes

A list of the institutions entitled to vote at the Nomenclature Section of the XIX International Botanical Congress (IBC), Shenzhen, was published by Funk & Turland (in Taxon 65: 1449–1454. 2016 https://doi.org/10.12705/656.33), as part of the report of the Special Committee on Institutional Votes, which was established by XVIII IBC, Melbourne in 2011. The General Committee had approved the list (Funk & Turland l.c.: 1450; Wilson in Taxon 66: 189–190. 2017 https://doi.org/10.12705/661.15) and later approved two additional requests for institutional votes (one vote for BSD, two for KH; Wilson in Taxon 66: 744. 2017 https://doi.org/10.12705/663.15). At the Nomenclature Section, two further institutions (GDGM and HK) applied for one institutional vote each, and these were added to the list.

The list below includes all institutions that were entitled to vote at the Shenzhen IBC. The format is as follows: herbarium code according to *Index herbariorum* (http://sweetgum.nybg.org/science/ih/); allocated number of votes; “ * ” indicating that the institution exercised its institutional vote(s) at the Shenzhen IBC; “new” indicating that the institution was new to the list for Shenzhen; “ 1↑ ” indicating that the allocated votes were increased by one vote since Melbourne. The “ + ” symbol is used between herbarium codes when two or more institutions were treated together, e.g. CAN + CANA + CANL + CANM were treated as a single institution with five votes. The total number of institutions on the list is 546; the total number of institutional votes that could potentially have been exercised is 931. At the Shenzhen IBC a total of 166 institutions exercised 427 institutional votes.

**Table:**

Herbarium code	Votes	Notes
AA	1	
AAS	1	
AAU	4	*
ABD	1	
ACAD	1	
ACOR	2	* new
AD	4	*
AK	2	*
ALA	1	
ALTA	1	
AMH + AHMA	1	
AMNH	1	
ANK	2	*
ANSM	1	
ANSP	1	
APP	1	new
AQUI	1	
ARIZ	1	
ASH	1	
ASSAM	2	* ↑1
ASU	1	*
ATCC	1	
ATHU	2	
AVE	1	
B	7	*
BA	3	*
BAA	1	
BAB	1	*
BAF	2	
BAFC	1	*
BAG	1	
BAK	1	
BAMU	2	* new
BARC	1	*
BAS	1	
BC	2	
BCN	2	
BEO	1	
BERN	1	
BG	2	
BH	3	
BHU	1	
BHUPM	1	
BI	1	
BILAS	1	
BIRM	1	
BISH	2	
BKF	2	*
BLAT	1	
BM	7	*
BNRH	1	
BO	4	
BOL	2	
BOLO	1	
BONN	1	
BORD	1	new
BP	5	
BPI	3	*
BR	6	*
BRA	1	*
BRG	1	
BRI	4	
BRIT	3	
BRLU	2	new
BRNM	1	
BRNU	1	
BRVU	1	
BRY	1	
BSA	1	*
BSD	1	* new
BSI	1	*
BUCA + BUCM	2	
BUF	1	
BURD	1	
C	7	*
CAF	1	
CAHUP	1	
CAI	2	
CAIM	1	
CAL	5	*
CAME	1	
CAN + CANA + CANL + CANM	5	*
CANB	6	*
CANU	1	
CAS	4	
CAT	1	
CAY	1	*
CBS	4	*
CCNU	1	
CDBI	2	
CFMR	1	
CFSHB	1	
CGE	1	
CGG	1	
CHAPA	1	
CHEP	1	new
CHIS	1	
CHR	3	*
CHRB	1	* new
CICY	1	
CIIDIR	1	*
CL	1	
CLF	1	*
CM	2	*
CMMI	1	
CNR	1	new
CNS	2	*
COI	3	
COL	3	*
COLO	1	
CONC	1	
CONN	1	
CORD	2	*
CR	1	
CSH	1	* new
CTES	1	
CUHK	1	*
CUP	1	
CUZ	1	new
DACB	1	
DAO + DAOM	6	*
DAR	1	*
DAV	1	
DAVFP	1	
DBN	1	
DD	2	
DNA	1	
DNZ	1	new
DR	1	new
DU	1	new
DUKE	2	*
E	6	*
EA	3	↑1
EAP	1	
EDTU	1	new
EGE	1	*
EIF	1	new
ENCB	3	
ERE	3	*
ETH	2	↑1
F	5	*
FCAB	1	new
FCME	1	
FCO	1	
FCQ	1	
FHI	1	
FHO	1	
FI + FT	4	*
FLAS	2	
FNU	1	
FR	1	*
FSU	1	
FTG	2	
FUS	1	
G	7	*
GA	1	
GAS	1	new
GAT	2	*
GAUBA	1	* new
GB	4	
GC	1	
GCU	1	new
GDA	1	
GDGM	1	* new
GE	1	* new
GENT	1	
GH	6	*
GI	1	
GJO	1	
GLM	1	* new
GMUF	1	
GNNU	1	new
GOET	2	* ↑1
GRA	1	
GUADA	1	
GUAM	1	
GZU	2	*
H	7	*
HA	1	* new
HAC	2	
HAJB	3	*
HAJU	1	* new
HAL	2	*
HAMAB	1	* new
HAS	1	
HAST	1	
HB	1	
HBG	3	
HBI	1	
HBNU	1	* new
HCIO	1	
HEID	1	new
HGAS	1	
HGU	1	new
HIB	1	
HIRO	2	
HK	1	* new
HMAS	5	*
HN	2	
HNM	1	new
HNNU	1	
HNU	1	
HNWP	2	
HO	1	
HSC	1	
HSNU	1	*
HSS	1	* new
HUA	1	
HUB	2	*
HUJ	2	
HUQ	1	new
HUT	1	
HUTI	1	* new
HWA	1	
HZU	1	
I	1	
IAN	1	
IB	1	
IBGE	1	
IBK	2	
IBSC	4	*
IBUG	2	
ICEL	1	
ICN	1	
ID	1	
IEB	1	
IFP	2	
IJ	1	
ILL	1	
ILLS	1	*
IND	1	
INH	1	* new
INPA	1	
IRAN	2	
ISC	2	
ISL	1	
ISMAR	1	new
ISTE	2	*
ISTF	1	
J	1	*
JACA	1	
JBSD	1	
JCT	1	
JE	3	
K	7	*
KANA	1	
KANU	1	
KATH	2	↑1
KB	3	new
KEP	1	
KFBG	1	new
KH	2	* new
KLU	1	
KMG	1	
KOR	1	*
KPABG	1	* new
KPBG	1	*
KR	1	
KRA	2	*
KRAM	3	*
KSC	1	
KUH	2	
KUN	5	*
KW	5	*
KWHU	1	new
KYO	2	*
L	7	*
LA	1	
LAE	1	
LATV	1	
LAU	1	
LBL	1	
LD	6	*
LDS	1	
LE	7	*
LECB	1	*
LG	2	
LI	1	
LIL	3	
LISC	2	
LISE	1	
LISI	1	
LISU	2	
LIV	2	
LJF	1	new
LJU	1	
LMA	1	
LMU	1	
LOD	1	
LP	1	*
LPB	1	
LPS	1	
LSU + LSUM	2	
LTB	1	
LTR	1	
LUX	1	
LW	1	
LWG	2	
LWI	1	new
LYD	1	*
LZ	1	
M	5	*
MA	4	*
MAF	1	*
MAG	1	*
MAK	2	
MAL	1	
MANCH	1	
MARSSJ	1	
MARY	1	
MASS	1	
MBM	1	*
MCNS	1	new
MEL	6	*
MELU	2	
MEXU	5	*
MG	1	
MGC	1	
MH	2	*
MHA	2	*
MHU	1	
MICH	4	
MIL	1	
MIN	2	
MJG	1	
MO	7	*
MONTU	1	
MOR	1	
MPU	2	*
MSB	1	*
MSC	2	
MSK	1	*
MSM	1	
MT	2	
MU	1	
MUB	1	
MUCL	1	
MVM	1	
MW	3	* ↑1
MY	1	
N	1	
NA	1	
NAI	1	
NAP	1	
NAS	2	
NBG	2	*
NCSC	1	
NCU	2	
NE	1	
NEFI	1	
NEU	1	
NF	1	
NH	1	*
NICH	2	
NMW	1	
NR	1	new
NRRL	2	
NS	1	*
NSK	1	*
NSW	6	*
NT	1	
NTUF	1	
NU	1	*
NWTC	1	
NY	7	*
NYS	1	
NZFRI	1	
O	5	
OAC	1	
OKL	1	
OLD	1	*
OS	2	
OSC	1	
OULU	1	
OWU	1	
OXF	1	
P + PC	7	* ↑1
PAC	1	
PACA	1	
PAD	2	*
PAL	3	*
PAV	2	
PDA	1	
PDD	1	*
PE	7	*
PERTH	3	*
PERU	1	
PG	1	new
PH	2	*
PI	2	
PMA	1	
PNH	1	
PO	1	
PORT	1	
POZ + POZG	2	*
PR + PRM	2	
PRC	3	*
PRE	6	*
PREM	2	*
PRU	1	*
PTBG	1	*
PUC	1	*
PYU	1	
QCA	1	
QCNE	1	
QFA	1	
R	2	
RAB	1	
RAW	1	
RB	6	* ↑1
REG	2	
RHT	1	
RIG	1	
RM	1	
RNG	2	
RO	1	
ROV	1	new
RSA	2	*
RUBL	1	
S	6	*
SAN	1	
SAP	1	
SAPS	1	new
SAR	1	
SARA	1	
SASK	1	
SAV	1	*
SBBG	1	
SBT	1	
SD	1	
SEL	1	new
SEV	2	
SFSU	1	
SGN	1	new
SGO	1	
SHIN	1	
SHM	1	
SI	3	*
SING	3	* ↑1
SIU	1	
SLO	2	*
SM	1	
SNU	2	
SOA	1	
SOM	3	
SP	4	*
SPF	2	*
SRGH	2	
SS	1	
STA	1	*
STU	2	
SUVA	1	
SVER	1	*
SWFC	1	
SYD	1	
SYS	1	*
SZ	1	
TAA	2	*
TAES	1	
TAI	1	
TAIF	1	
TAN	1	
TARI	1	
TASH	2	
TAU	1	
TBI	2	
TCD	1	*
TELA	1	
TENN	1	
TEX	4	*
TFC	1	
TI	4	
TK	1	*
TL	1	*
TMI	1	
TNS	5	
TO	1	
TRH	2	
TRIN	1	*
TRN	1	*
TRT	1	*
TRTC	1	*
TSB	2	
TSH	1	new
TU	2	*
TUB	1	
TUR	2	*
TUS	1	
UAMH	1	
UAMIZ	1	
UB	1	
UBA	1	
UBC	2	
UC	5	
UCJ	1	
UCR	1	
UDW	1	
UEC	2	*
UGDA	1	* new
UKMB	1	
ULT	1	
UME	2	
UNL	1	
UNM	1	
UPA	2	
UPS	4	*
URM	1	
US	7	*
USF	1	*
USM	2	
USMS	1	* new
UT	1	*
UTC	1	*
UWO	1	*
UWSP	1	*
V	1	
VAL	2	
VEN	2	
VLA	1	
VPI	1	
VT	1	
W	3	
WA	3	*
WELT	2	↑1
WH	1	
WIND	1	
WIR	2	* ↑1
WIS	2	
WRSL	1	*
WS	1	
WSP	1	
WSY	1	*
WTU	2	
WU	5	*
WUK	2	*
WVA	1	
XAL	2	*
XJA	1	
XJBI	1	
XJU	1	
YA	1	
YU	1	*
YUKU	1	
Z + ZT	3	
ZA	1	
ZULU	1	

## Appendix C

List of registered members of the Nomenclature Section

Names are cited in the format: Family name(s), Given name(s). Names and initials are those provided by the members upon registration. Initials have not been added if they were not provided. Chinese and Korean given names consisting of two syllables have been standardized in the format (e.g.) De-Zhu (rather than De Zhu, De-zhu, or Dezhu).

Alford, Mac (USA)

Aninha, Maria (India)

Aoki, Takayuki (Japan)

Appelhans, Marc (Germany)

Applequist, Wendy (USA)

Attullil, Anna Ancy Antony (India)

Bai, Lin (China)

Barkworth, Mary (USA)

Barrie, Fred (USA)

Bensch, Konstanze (Netherlands)

Bhagat, Rani (India)

Cai, Wen-He (China)

Callmander, Martin (Switzerland)

Cantrill, David (Australia)

Cantuária, Patrick (Brazil)

Cárdenas Ramírez, Glenda Gabriela (Finland)

Chandore, Arun (India)

Chang, Chin-Sung (South Korea)

Chang, Kae-Sun (South Korea)

Chen, Jiong-Shan (China)

De Beer, Zacharias Wilhelm (South Africa)

De Lange, Peter (New Zealand)

Deng, Hui (China)

Deng, Wang-Qiu (China)

Deng, Yun-Fei (China)

Dhabe, Arvind (India)

Do Nascimento Moura, Carlos Wallace (Brazil)

Dönmez, Ali A. (Turkey)

Dorr, Laurence (USA)

Du, Cheng (China)

Du, Jia-Mu (China)

Duretto, Marco (Australia)

Feng, Shu-Cheng (China)

Fonseca, Maria A. A. R. (India)

Fortunato, Renée (Argentina)

Freire-Fierro, Alina (Ecuador)

Funk, Vicki A. (USA)

Gandhi, Kanchi (USA)

Garland, Mark (USA)

Ge, Bin-Jie (China)

Geltman, Dmitry (Russia)

Gereau, Roy (USA)

Gernandt, David (Mexico)

Gillespie, Lynn (Canada)

Govaerts, Rafael (UK)

Greuter, Werner (Germany)

Groom, Quentin (Belgium)

Gürdal, Bahar (Turkey)

Han, Sang-Kuk (South Korea)

Hartley, Helen (UK)

Hawksworth, David (UK)

He, Xiao-Lan (China)

Herendeen, Patrick (USA)

Hirai, Regina Yoshie (Brazil)

Huang, Ti-Ran (China)

Ilechukwu, Ejike (Nigeria)

Jadhav, Neeta (India)

James, Shelley (Australia)

Kambale, Sharad (India)

Kim, Hui (South Korea)

Kim, Hye-Won (South Korea)

Kirk, Paul (UK)

Klopper, Ronell R. (South Africa)

Knapp, Sandra (UK)

Kolte, Rutuja (India)

Kusber, Wolf-Henning (Germany)

Kwan, Cheung-Ting (China)

Lau, Tai-Wai David (China)

Lee, Suah (South Korea)

Lehtonen, Samuli (Finland)

Levin, Geoffrey (USA)

Li, Bo (China)

Li, De-Zhu (China)

Li, Tai-Hui (China)

Li, Wen-Jun (China)

Liao, Shuai (China)

Liao, Wen-Bo (China)

Lin, Zheng-Mei (China)

Lindon, Heather (UK)

Lindsay, Stuart (Singapore)

Liu, Ho-Yih (Taiwan, China)

Liu, Pei-Liang (China)

Liu, Yea-Chen (Taiwan, China)

Loizeau, Pierre-André (Switzerland)

Luong, Thientam (Finland)

Lyon, Stephanie (USA)

Ma, Jin-Shuang (China)

Mabberley, David (Australia)

Malécot, Valéry (France)

Marhold, Karol (Slovakia)

Mascarenhas, Maria Emilia (India)

May, Tom (Australia)

McNeill, John (UK)

Middleton, David (Singapore)

Miller, James S. (USA)

Monro, Anna (Australia)

Nagamasu, Hidetoshi (Japan)

Nakada, Takashi (Japan)

Nge, Francis (Australia)

Pang, Kuen-Shum (China)

Paton, Alan (UK)

Peterson, Paul M. (USA)

Pokle, Dileep (India)

Prado, Jefferson (Brazil)

Pranee, Nangngam (Thailand)

Prasad, Suresh Hendre (Kenya)

Price, Michelle (Switzerland)

Proćków, Jarosław (Poland)

Qin, Hai-Ning (China)

Qin, Xin-Sheng (China)

Redhead, Scott (Canada)

Reier, Ülle (Estonia)

Renner, Susanne Sabine (Germany)

Resmi, S. (India)

Romaschenko, Konstantin (USA)

Saarela, Jeffery (Canada)

Schori, Melanie (USA)

Schuettpelz, Eric (USA)

Sennikov, Alexander (Finland)

Seregin, Alexey P. (Russia)

Shen, Mineya (Taiwan, China)

Shi, Shuo (China)

Shimizu, Gustavo (Brazil)

Shimpale, Vinod (India)

Singh, Paramjit (India)

Smith, Gideon (South Africa)

Söderström, Lars (Norway)

Song, Zhen (China)

Soreng, Robert (USA)

Struwe, Lena (USA)

Suddee, Somran (Thailand)

Sun, Xuan-Ting (China)

Thiele, Kevin (Australia)

Tong, Yi-Hua (China)

Turland, Nicholas (Germany)

Ugbogu, Omokafe Alaba (Nigeria)

Wang, Chao-Qun (China)

Wang, Ke (China)

Wang, Ya-Ning (China)

Watson, Mark (UK)

Wei, Tie-Zheng (China)

Wiersema, John (USA)

Wilson, Karen L. (Australia)

Wiltshire-Hawksworth, Patricia (UK)

Xia, Nian-He (China)

Yadav, Shrirang Ramchandra (India)

Yadav, Usha (India)

Yang, Tuo (China)

Yang, Yong (China)

Yang, Yu-Chang (China)

Zeng, Si-Jin (China)

Zhang, Ming (China)

Zhang, Qiang (China)

Zhu, Rui-Liang (China)

Zhu, Xiang-Yun (China)

